# Photocatalytic Decarboxylative Coupling of Aliphatic N‐hydroxyphthalimide Esters with Polyfluoroaryl Nucleophiles

**DOI:** 10.1002/anie.202108465

**Published:** 2021-09-28

**Authors:** Xiangli Yi, Runze Mao, Lara Lavrencic, Xile Hu

**Affiliations:** ^1^ Laboratory of Inorganic Synthesis and Catalysis Institute of Chemical Sciences and Engineering Ecole Polytechnique Fédérale de Lausanne (EPFL) ISIC-LSCI, BCH 3305 1015 Lausanne Switzerland

**Keywords:** aliphatic acid, copper, photocatalysis, polyfluoroarylation, reaction mechanisms

## Abstract

Polyfluoroarenes are an important class of compounds in medical and material chemistry. The synthesis of alkylated polyfluoroarenes remains challenging. Here we describe a decarboxylative coupling reaction of N‐hydroxyphthalimide esters of aliphatic carboxylic acids with polyfluoroaryl zinc reagents (Zn‐Ar_F_) via synergetic photoredox and copper catalysis. This method readily converts primary and secondary alkyl carboxylic acids into the corresponding polyfluoroaryl compounds, which could have a wide range of F‐content (2F‐5F) and variable F‐substitution patterns on the aryl groups. Broad scope and good functional group compatibility were achieved, including on substrates derived from natural products and pharmaceuticals. Mechanistic study revealed that a [Cu‐(Ar_F_)_2_] species could be responsible for the transfer of polyfluoroaryl groups to the alkyl radicals.

## Graphical abstract

Decarboxylative coupling of aliphatic N‐hydroxyphthalimide esters with polyfluoroaryl zinc reagents was achieved by dual photoredox and copper catalysis. This method allows the installation of alkyl groups on polyfluoroaryls with a wide range of F‐content (2F‐5F) and variable F‐substitution patterns.

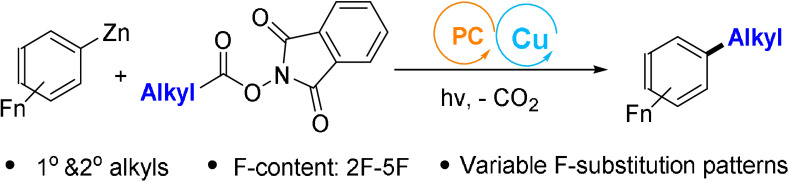

Polyfluoroarenes can form special intermolecular interactions,^[1]^ such as π–π_F_ and anion–π_F_ interactions, which lead to widespread applications in pharmaceuticals^[2]^ and materials[^1a^, ^3^] (Scheme 1 a). The synthesis of polyfluoroaryl compounds from easily available simple polyfluoroarenes has drawn much recent attention. Strategies such as SN_Ar_ reactions^[4]^ on polyfluoroarenes, reactions via polyfluoroaryl radicals^[5]^ and radical addition to polyfluoroarenes^[6]^ have been reported. However, these strategies generally require highly electron‐deficient polyfluoroarenes, which makes them unsuitable for arenes with a lower F‐content.

**Scheme 1:**
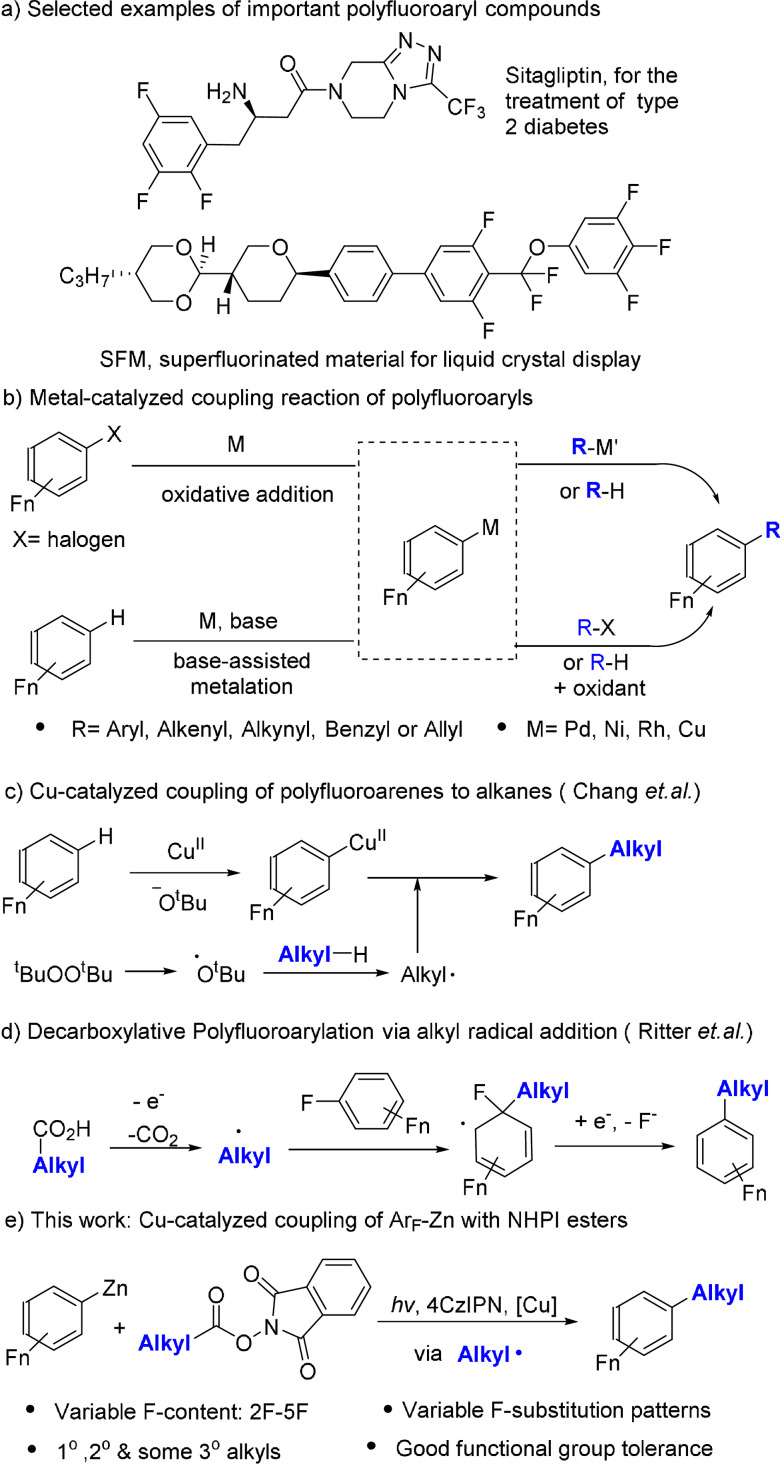
Examples of polyfluoroaryl compounds and their synthesis by the coupling reactions of polyfluoroaryl reagents.

Metal catalyzed C−C cross‐coupling of polyfluoroaryl reagents (X‐Ar_F_, H‐Ar_F_ and M‐Ar_F_, where X is a halide, M is a metal), on the other hand, provides a more general and versatile approach to the synthesis of polyfluoroaryl compounds (Scheme 1 b).^[7]^ Many examples of the coupling of polyfluoroaryls with aryl,^[8]^ alkenyl,^[9]^ alkynyl,^[10]^ benzyl^[11]^ and allyl^[12]^ groups have been reported. However, the coupling of polyfluoroaryls with unactivated alkyl groups^[13]^ remains challenging, possibly due to the difficulty in the reductive elimination step as a result of a strong M‐Ar_F_ bond^[14]^ and a facile β‐H elimination reaction from many M‐alkyl intermediates. In an important development, Chang and co‐workers reported a Cu‐catalyzed method for the oxidative coupling of polyfluoroarenes with alkanes (Scheme 1 c).^[13a]^ This reaction involves the *tert*‐butoxide‐assisted C−H metalation of a polyfluoroarene to form an Cu^II^‐Ar_F_ species, which captured an alkyl radical to effect the coupling. Despite the advance, arenes with a low F‐content (e.g. 2F and 3F) were not suitable substrates, likely because the Ar_F_−H bonds of the low‐F‐content arenes were less acidic and resistant to base‐assisted C−H metalation. The reaction also has relatively harsh conditions and suffers from the regioselectivity problem of C−H activation for many substrates.

The group of Ritter recently developed decarboxylative polyfluoroarylation of alkyl carboxylic acids based on radical addition to polyfluoroarenes followed by the elimination of ipso‐fluorine (Scheme 1 d).^[6]^ This novel method still has some limitations such as regioselectivity of radical addition. The method was less efficient for polyfluoroarenes with 4F and not suitable for those with 3F or less.

Here we describe a metallophotoredox approach^[15]^ for the coupling of Ar_F_‐Zn reagents with aliphatic NHPI esters synthesized from NHPI (N‐hydroxyphthalimide) and alkyl carboxylic acids (Scheme 1 e). Unlike their unstable lithium and Grignard analogues,^[16]^ the Zn‐Ar_F_ reagents^[17]^ are more stable and less reactive, leading to high functional group tolerance. Using these preformed reagents, we were able to install Ar_F_ groups with a wide range of F‐content (2F‐5F) and with varied F‐substitution patterns. Although the coupling reaction of NHPI esters is well established with many organometallic reagents,^[18]^ such coupling with weakly nucleophilic Ar_F_‐Zn reagents is hitherto unknown. Compared to the method of Ritter,^[6]^ our organometallic approach avoids the problem of regioselectivity and is suitable for polyfluoroaryls with low F‐content.

We started our exploration using (diglyme)Zn(C_6_F_5_)_2_ (**b1**) as the source of ‐Ar_F_, which could be obtained as a stable solid from a simple reaction of pentafluoroiodobenzene with diethylzinc. After a thorough screening of reaction conditions (Figure S1–3, Table S1,2, SI), we found that with 1 mol % of Ir(dfppy)_2_(ppy) as photocatalyst, 10 mol % of Cu(OTf)_2_ as catalyst and 20 mol % of dtbbpy (4,4′‐di‐*tert*‐butylbipyridine) as ligand, the NHPI ester of 1‐benzoylpiperidine‐4‐carboxylic acid (**a1**) was coupled with **b1** to give the desired product **c1** in excellent yield (95 %, Table 1, entry 1). Selected examples of reaction optimization highlighting the influence of key reaction parameters are described in Table 1. Fe(OTf)_2_ and NiCl_2_(DME) (DME=dimethoxyethane) were not effective metal catalysts (Entries 2 and 3, Table 1). L1, which was the best ligand in Chang's work,^[13a]^ was not efficient for this reaction (Entry 4, Table 1). When 1 equivalent of C_6_F_5_‐ZnCl was used as a source of ‐Ar_F_, the side product from chlorination (44 %) outweighed the coupling product **c1** (22 %, Entry 5, Table 1). To our delight, when we replaced Ir(dfppy)_2_(ppy) with 4CzIPN, an easily accessible and inexpensive organic photocatalyst,^[19]^ the coupling was highly efficient as well, with a yield of 93 % (Entry 6, Table 1). Thus, we used 4CzIPN as the final choice of photocatalyst. When light illumination or the photocatalyst was eliminated, no coupling occurred (Entry 7, Table 1), confirming the necessity of photocatalysis. When the copper catalyst was removed, the product could not be obtained neither (Entry 8, Table 1).

**Table 1:** Selected examples of reaction optimization highlighting the influence of key reaction parameters.^[a]^ 
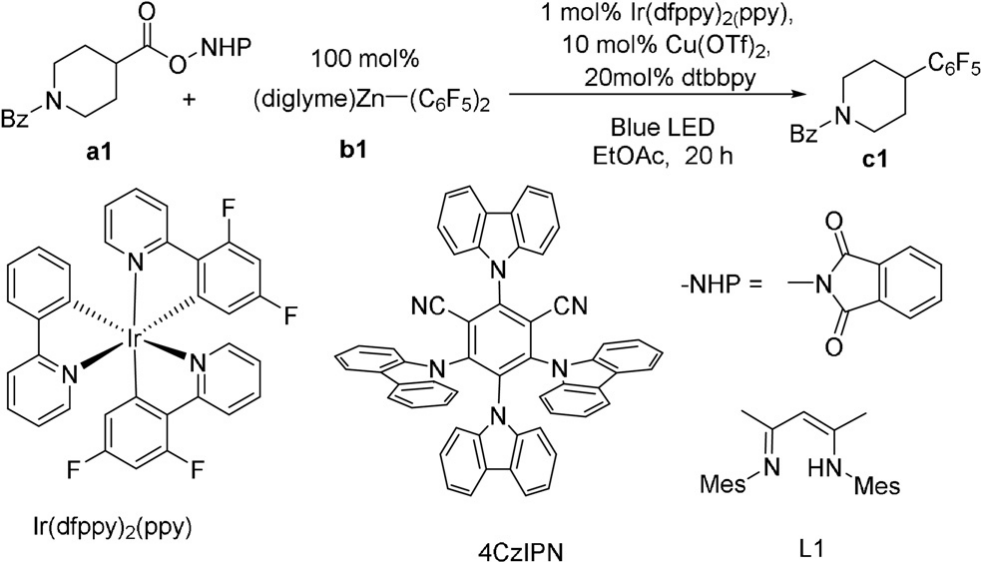

Entry	Variations	Yield^[b]^ [%]
1	–	95
2	Fe(OTf)_2_ instead of Cu(OTf)_2_	15
3	NiCl_2_(DME) instead of Cu(OTf)_2_	0
4	L1 instead of dtbbpy	22
5	C_6_F_5_‐ZnCl + 1 equiv. diglyme instead of (diglyme)Zn(C_6_F_5_)_2_	23
6	4CzIPN instead of Ir(dfppy)_2_(ppy)	93 (85^[c]^)
7	No radiation or no photocatalyst	0
8	4CzIPN instead of Ir(dfppy)_2_(ppy), no Cu(OTf)_2_	0

[a] Reaction conditions: 0.1 mmol **a1**, 0.1 mmol **b1** and other additives in 0.5 mL EtOAc. Reaction under Blue LED for 20 h. [b] GC yield with mesitylene as internal standard. [c] Isolated yield.

Based on the optimized conditions (Entry 6, Table 1), we evaluated the substrate scope of NHPI esters (Figure 1). For secondary alkyl NHPI esters, a large number of cyclic (**c1**–**c7**) and acyclic (**c8**–**c10**) alkyl groups, including those with considerable steric hindrance (e.g, **c10**) were coupled to polyfluoroaryls in good yields. Sulfonamide (**c2**), ether (**c3**), ketone (**c5**), and electron‐rich arene (**c9**) were compatible with the reaction conditions. Several pharmaceuticals, such as Ibuprofen, Ketoprofen and Naproxen, could be modified via this method into polyfluoroaryl compounds (**c11**–**c13**) in excellent yields. A substrate with an α‐oxyalkyl group was not suitable for the reaction, with a GC yield of less than 5 %, possibly due to facile direct oxidation of the α‐oxyalkyl radical^[20]^ in competition with the trapping by copper catalyst.

**Figure 1:**
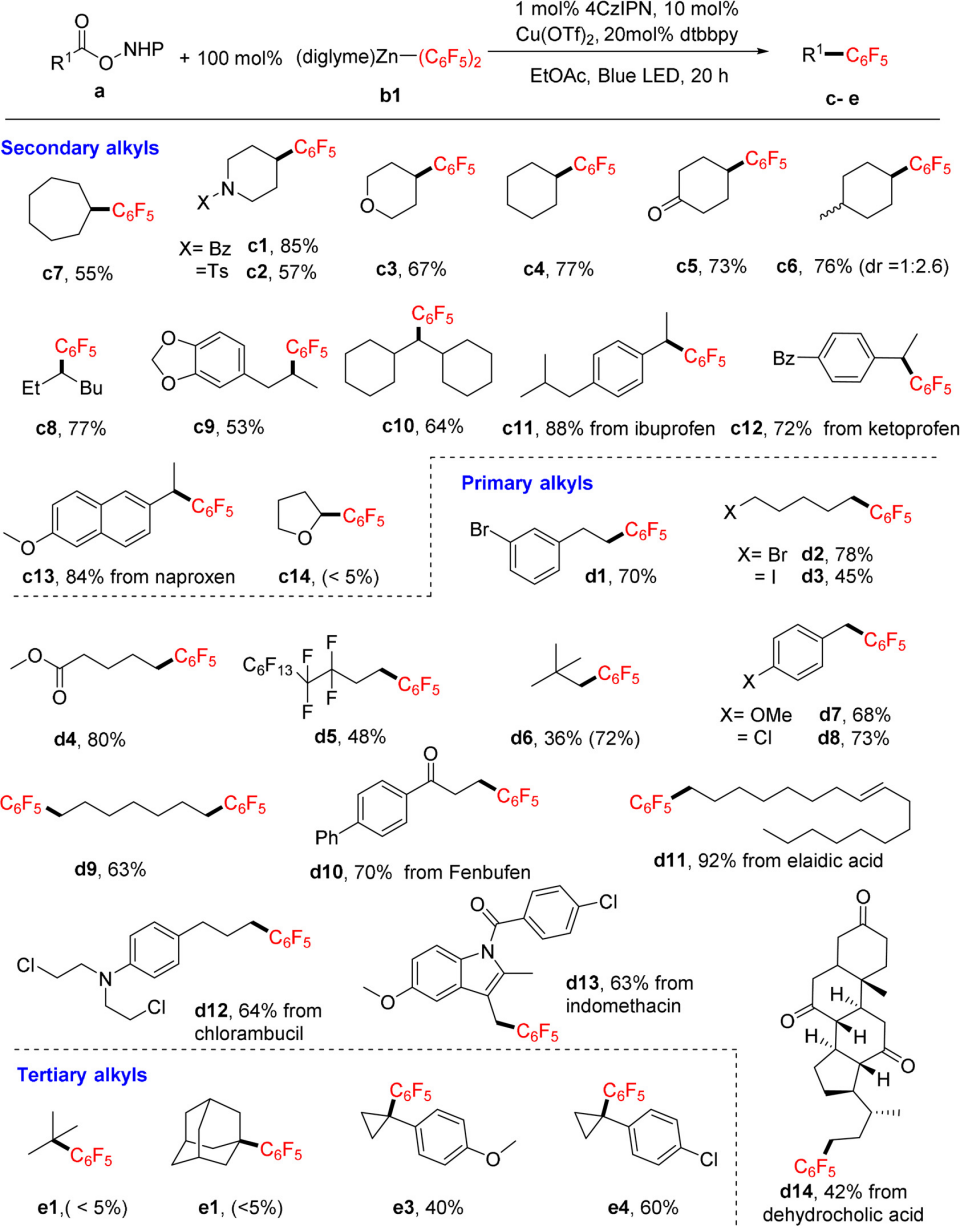
Scope of alkyl NHPI esters. Reaction conditions: 0.1 mmol **a**, 0.1 mmol **b1** and other additives in 0.5 mL EtOAc. Reaction under Blue LED for 20 h. Isolated yields and GC yields (in bracket) are shown.

A wide range of primary alkyl NHPI esters were also suitable substrates for this reaction (**d1**–**d14**, Figure 1). Notably, the arylbromide (**d1**), alkylbromide (**d2**) and alkyliodide (**d3**) groups, which serve as electrophiles in many cross‐coupling reactions,^[21]^ could be tolerated in this reaction, leaving a synthetic handle for further transformation. A hindered primary alkyl (**d6**) and primary benzyls (**d7**, **d8**) were also suitable for this transformation. Despite their high molecular weights, some polyfluoro products were volatile (**d5**, **d6**) and suffered from substantial loss of yields during separation (e.g. for **d6**, 72 % GC yield but 36 % isolated yield). Double polyfluoroarylation on the same substrate was demonstrated (**d9**). Reactions of NHPI esters derived from natural products and pharmaceuticals (**d10**–**d14**) also led to high coupling yields (**d10–d13**). The reaction of the NHPI ester of the structurally complicated dehydrocholic acid had a modest yield of 42 % (**d14**). For the coupling of tertiary alkyl NHPI esters, the reaction was generally not productive (e.g. **e1**, **e2**). Some specific substrates, e.g., those with a cyclopropyl ring, were coupled in modest to good yields (**e3**, **e4**).

We next explored the scope of polyfluoroaryl zinc reagents (Figure 2). Diaryl zinc reagents with different F‐content and F‐substitution patterns were prepared from polyfluoroaryl lithium reagents and used as solutions of ethyl acetate. We used two representative alkyl NHPI ester substrates, one with a primary alkyl and one with a secondary alkyl group, which upon coupling gave products **f** and **g**. The reactions of the 2,3,5,6‐tetrafluoro‐4‐iodophenyl zinc reagent gave **f1** and **g1** in moderate yields. On the *para* position, different groups including ‐CF_3_, ‐OPh, ^n^hexyl, phenyl, silyl (TIPS, triisopropylsilyl), alkynyl were all tolerated in the reaction, leading to the corresponding products (**f2**–**f7**, **g2**–**g7**) in good yields. Then, the coupling to a set of aryls with varied F‐content and F‐substitution patterns was probed. All three isomers of tetrafluorophenyl could be coupled to a primary alkyl (**f8**–**f10**) or a secondary alkyl (**g8**–**g10**) group in good yields. Trifluorophenyl zinc reagents were successfully applied to the reaction as well (**f11**–**f13**, **g11**–**g13**), so was the 2,6‐difluorophenyl zinc reagent (**f14**, **g14**). The coupling of 2,4‐difluorophenyl zinc was inefficient (30 % for **g15** and <5 % for **f15**). Likewise, the coupling of 2‐fluorophenyl or 4‐fluorophenyl zinc reagent was not successful (<5 % for **f16**, **f17**, **g16** and **g17**). In these cases, the relatively electron rich aryl zinc reagents were prone to homocoupling to give biaryls. The electron‐deficient tetrafluoropyridyl zinc reagent was not suited to this reaction as well (29 % for **g18** and 0 % for **f18**). Notably, 1 equivalent of diglyme could generally enhance the yields by 5–10 % (Figure S3), possibly because diglyme acted as a ligand to promote the aryl transfer process.

**Figure 2:**
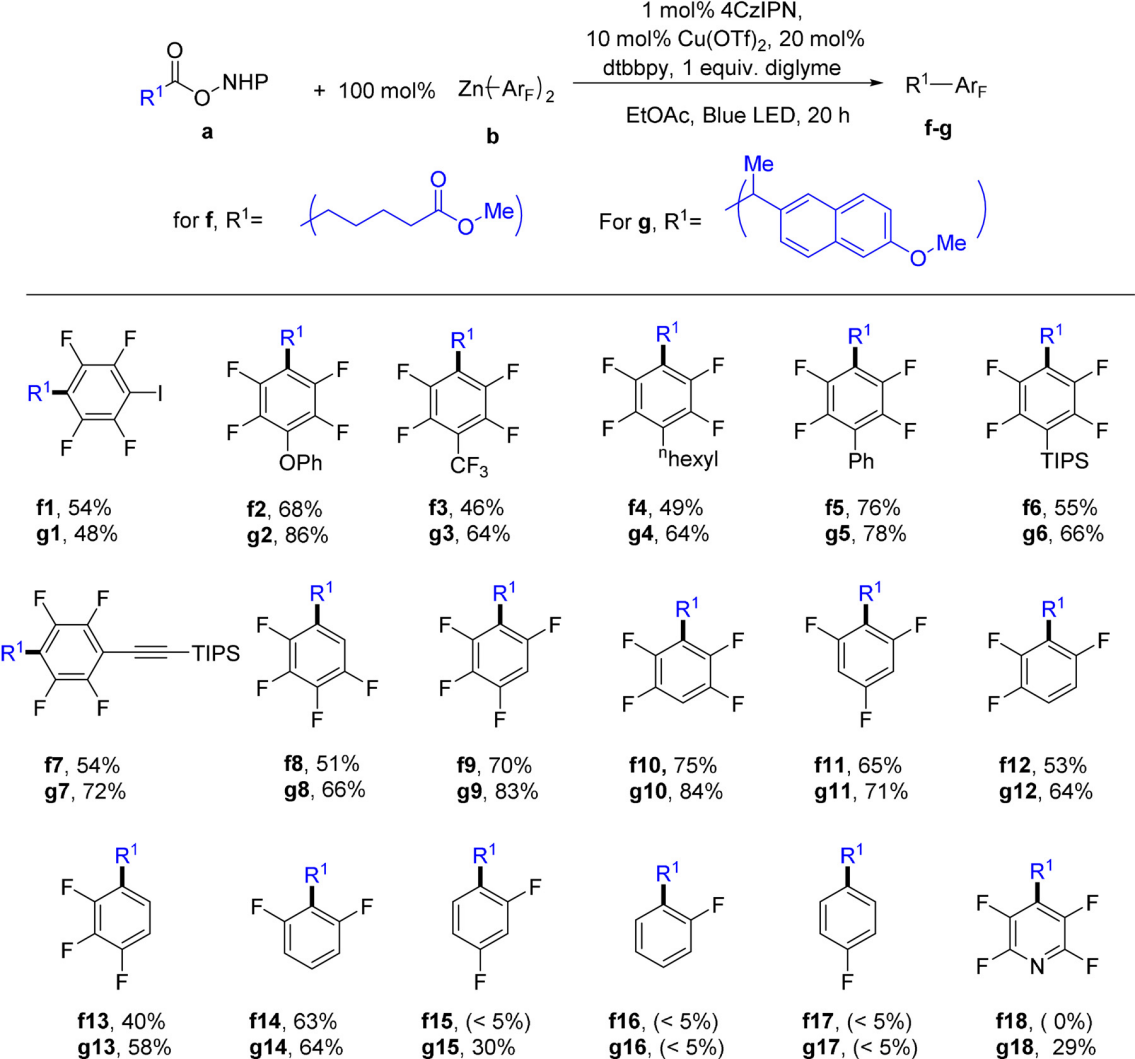
Scope of polyfluoroaryl zinc reagents. Reaction conditions: 0.1 mmol **a**, 0.1 mmol **b1** and other additives in 0.5 mL EtOAc. Reaction under Blue LED for 20 h. Isolated yields and GC yields (in bracket) are shown.

Several experiments were conducted to shed light on the mechanism of the coupling. When the NHPI ester derived from 6‐heptenoic acid (**a2**) was used as a substrate to couple with **b1** under the standard conditions [Eq. (1), Scheme 2], **h1**, a product formed via 5‐exo‐trig cyclization of 5‐hexenyl radical, was obtained as the only cross‐coupling product (60 % yield). This result is consistent with the intermediacy of an alkyl radical formed from the alkyl NHPI ester. When (dtbbpy)Cu(OTf)_2_ was mixed with1 equivalent of **b1**, the homocoupling product C_6_F_5_‐C_6_F_5_ was formed in 42 % yield [Eq. (2), Scheme 2). This result is consistent with previous reports that Cu^II^ species could oxidize Zn‐Ar_F_ to Ar_F_‐Ar_F_.^[22]^ Thus, the resting oxidation state of Cu in the catalytic system is likely Cu^I^. The Cu^I^ complex [(bpy)Cu(C_6_F_5_)] was synthesized and was found to be a competent catalyst as well [Eq. (3), Scheme 2]. However, the stoichiometric reaction of [(bpy)Cu(C_6_F_5_)] with **a1** under photochemical conditions didn't give any coupling product [Eq. (4), Scheme 2]. In a crossover experiment, **a1** was treated with 50 mol % of [(bpy)Cu(C_6_F_5_)] and 100 mol % of **b1** [Eq. (5), Scheme 2]. Coupling with both ‐C_6_F_5_ and ‐C_6_F_4_H occurred with a total yield of 52 % (‐C_6_F_4_H: ‐C_6_F_5_=1:0.18 in the products, =1:0.25 in the starting materials). These results indicate that [(bpy)Cu(C_6_F_5_)] could enter the catalytic cycle and transfer the ‐C_6_F_5_ group on the copper into the product. However, it cannot directly transfer the ‐C_6_F_5_ group without a further transformation.

**Scheme 2:**
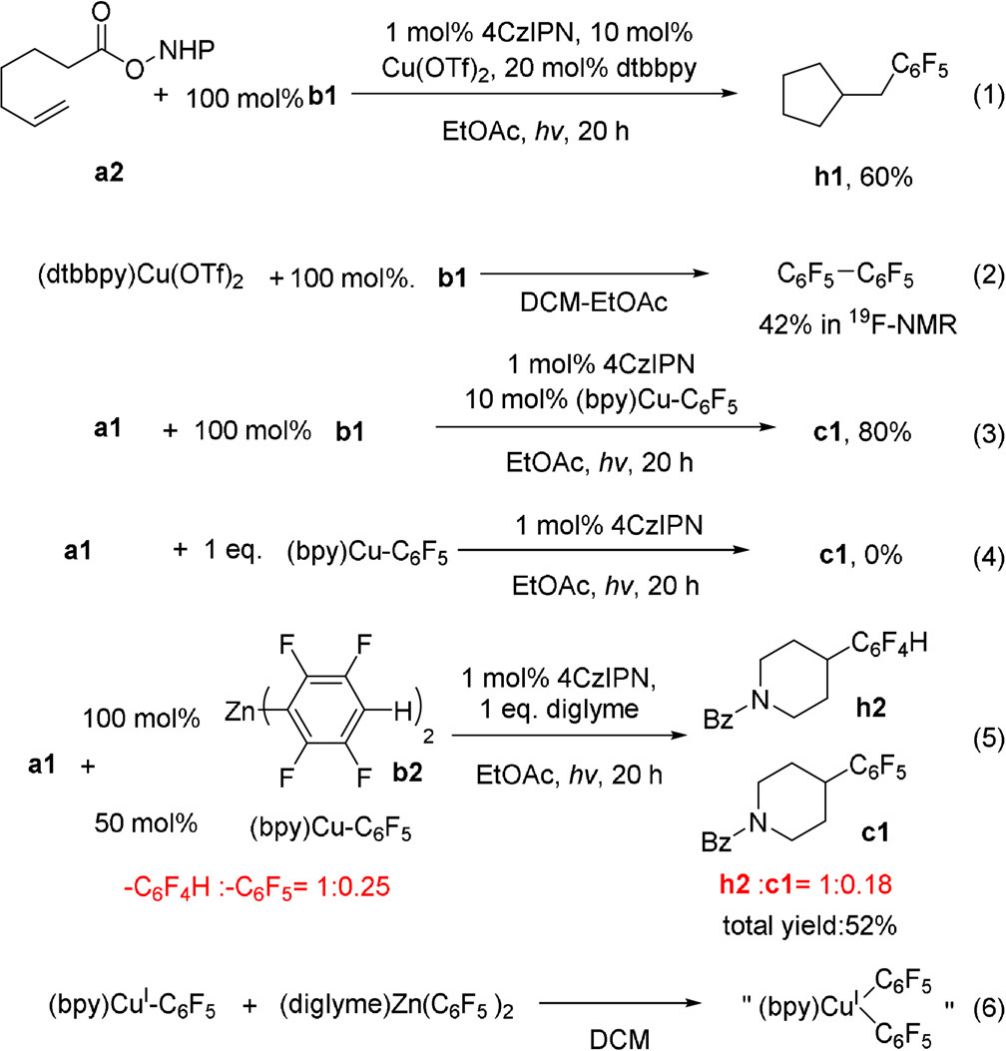
Mechanistic investigations.

When **b1** was added to a solution containing [(bpy)Cu(C_6_F_5_)], an instant color change from orange to light yellow was observed. With UV/Vis spectroscopy, we observed that increasing the ratio of **b1** to [(bpy)Cu(C_6_F_5_)] in dichloromethane led to a significant decrease of absorbance of [(bpy)Cu(C_6_F_5_)] (Figure S4), which could indicate a transmetallation process. Likewise, in the ^19^F‐NMR spectra of the mixture of **b1** and [(bpy)Cu(C_6_F_5_)], a new peak **A** was generated and the peak **B** in the original spectrum of [(bpy)Cu(C_6_F_5_)] disappeared (Figure 3 a). This suggests the conversion of [(bpy)Cu(C_6_F_5_)] to a new M‐C_6_F_5_ species with the addition of **b1**. In the cyclic voltammograms (Figure 3 b), a new oxidation peak emerged at −0.57 V vs. Fc^+^/Fc (Fc=ferrocene) when **b1** was added to [(bpy)Cu(C_6_F_5_)], which was 0.60 V lower than the oxidation peak of [(bpy)Cu(C_6_F_5_)]. These results could be attributed to the formation of a [(bpy)Cu(C_6_F_5_)_2_] species upon the addition of **b1** to [(bpy)Cu(C_6_F_5_)] [Eq. (6), Scheme 2], which could be the species responsible for transferring the ‐C_6_F_5_ group to alkyl radicals.

**Figure 3:**
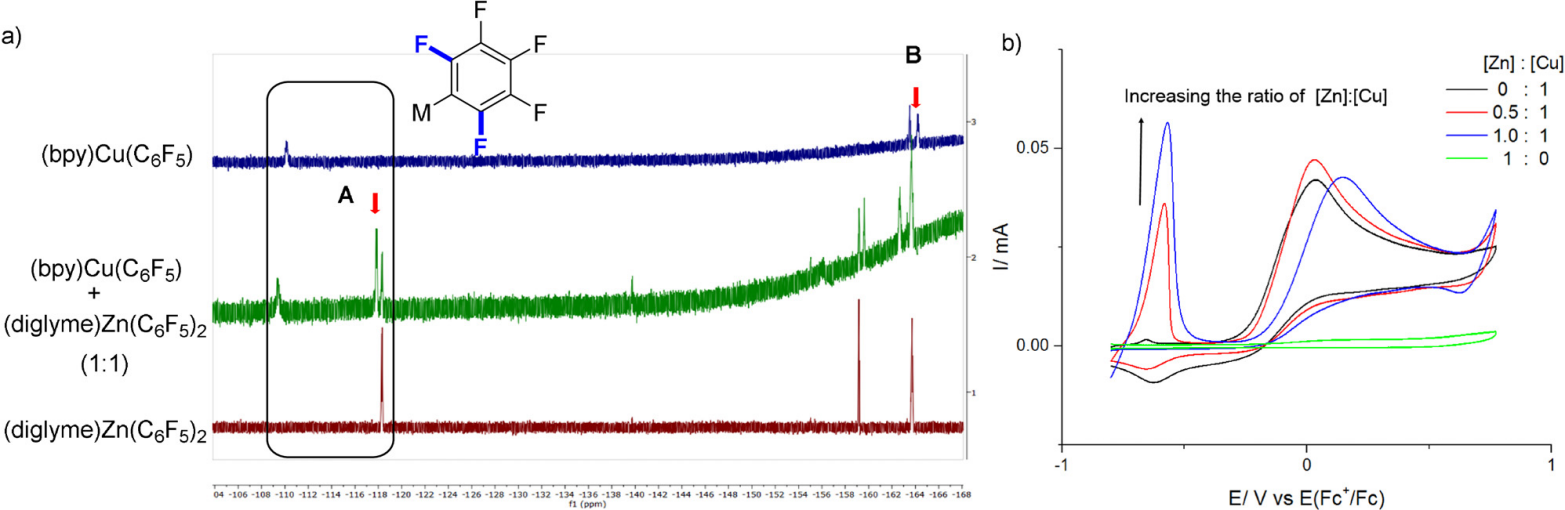
a) ^19^F‐NMR spectra of **b1**, [(bpy)Cu(C_6_F_5_)], and their mixtures. c(**b1**)=c[(bpy)Cu(C_6_F_5_)]=0.002 M in CH_2_Cl_2_ solution. b) Cyclic voltammograms of **b1**, [(bpy)Cu(C_6_F_5_)], and their mixtures in CH_2_Cl_2_‐CH_3_CN (2:1). c(**b1**) was 0.0027 M when alone. In all other samples, c[(bpy)Cu(C_6_F_5_)] was 0.0027 M and c(**b1**) was adjusted according to the given ratio. Conditions: Bu_4_NBF_4_ (0.03 M), glassy carbon disk as working electrode, Pt wire as counter electrode, Ag|AgCl, KCl(aq) as reference electrode (calibrated with Fc^+^/Fc). The scan rate: 100 mV s^−1^.

Stern–Volmer quenching was used to probe the interaction of the excited photocatalyst with different reagents (Figure S5–7, SI). NHPI ester **a1** and the Zn reagent **b1** did not quench the emission of the excited photocatalyst, yet both [(bpy)Cu(C_6_F_5_)] and the 1:1 mixture of **b1** with (bpy)CuC_6_F_5_ were effective quenchers. These data suggest that the reaction possibly starts by a reductive quenching of the excited 4CzIPN by the Cu^I^ species. Considering the large excess of **b1** to copper catalyst under the coupling conditions, the neutral [LCu(Ar_F_)] are expected to have a very low concentration and [LCu(Ar_F_)_2_]^−^ is more likely to be the quencher of the excited 4CzIPN.

Based on these results, we propose a plausible mechanism (Scheme 3). The reaction starts with the reductive quenching of the excited 4CzIPN by the [LCu^I^(Ar_F_)_2_] species **i** to form the reduced 4CzIPN and a LCu^II^(Ar_F_)_2_ species (**j**). The radical anion of 4CzIPN then reduces the NHPI ester **a** to give an alkyl radical after decarboxylation. The alkyl radical is captured by **j** to form a formal Cu^III^ intermediate **k**, which undergoes reductive elimination to give the coupling product and a LCu^I^(Ar_F_) species **l**. Although there is no precedent for a polyfluoroaryl alkyl reductive elimination from such a Cu^III^ complex, reductive elimination from an analogous [(bpy)Cu(CF_3_)_2_(CH_3_)] was reported to form CF_3_CH_3_ and [(bpy)Cu(CF_3_)].^[23]^ On the other hand, an out‐sphere ‐Ar_F_ transfer process (in dash square) could not be ruled out. [LCu^I^(Ar_F_)] undergoes transmetallation with the Zn‐Ar_F_ reagent to regenerate **i** and closes the catalytic cycle.

**Scheme 3:**
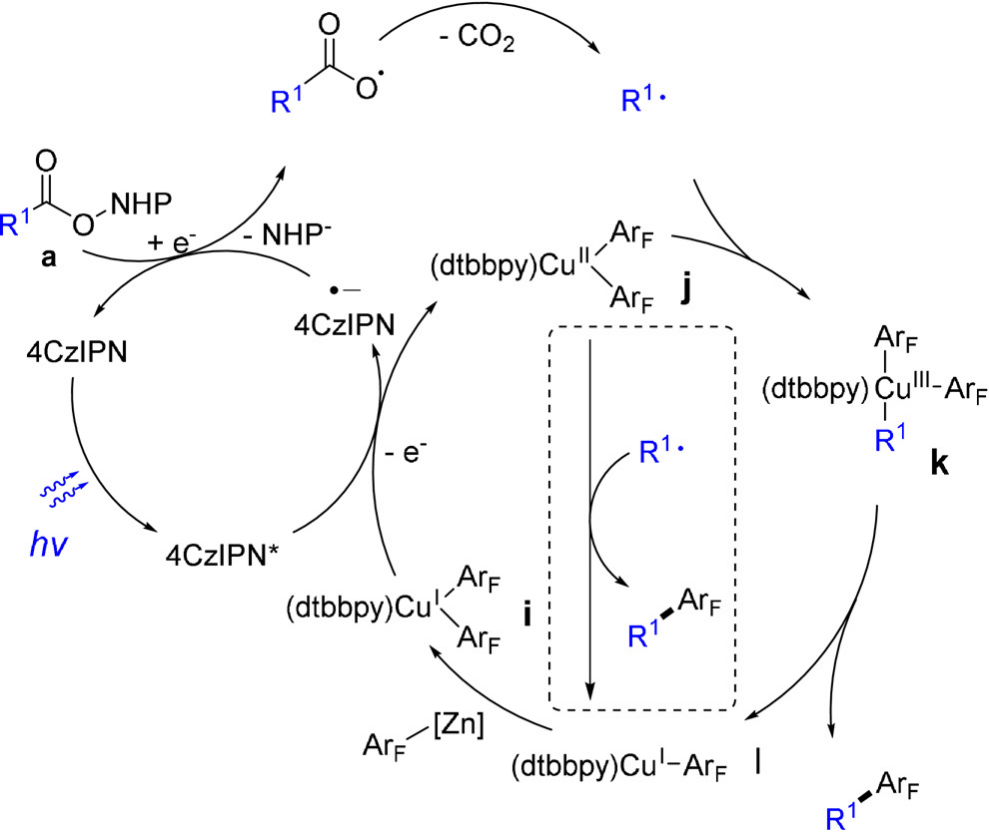
The proposed mechanism.

In summary, we have developed a dual photo‐ and Cu‐catalyzed method for the decarboxylative coupling of aliphatic acids with polyfluoroaryl zinc reagents. This method allows the installation of polyfluoroaryls with variable F‐content and F‐substitution patterns on a primary or secondary alkyl group, with good compatibility of functional groups. Our strategy might be extended to the coupling of Zn‐Ar_F_ reagents with alkyl radicals generated by other methods, leading to new methods in polyfluoroarylation.

## Conflict of interest

The authors declare no conflict of interest.

## Supporting information

As a service to our authors and readers, this journal provides supporting information supplied by the authors. Such materials are peer reviewed and may be re‐organized for online delivery, but are not copy‐edited or typeset. Technical support issues arising from supporting information (other than missing files) should be addressed to the authors.

Supporting Information
