# Supplementary material for: Photocatalytic Decarboxylative Coupling of Aliphatic N‐hydroxyphthalimide Esters with Polyfluoroaryl Nucleophiles
Source: Angew Chem Int Ed Engl. 2021 Sep 28;60(44):23557–63. doi: 10.1002/anie.202108465 (PMC8596744; doi:10.1002/anie.202108465)
Supplement: Supplementary file 1 — Supporting Information [file ANIE-60-23557-s001.pdf]

Angewandte Chemie
Eine Zeitschrift der Gesellschaft Deutscher Chemiker

Supporting Information

Photocatalytic Decarboxylative Coupling of Aliphatic N-hydroxyphthalimide Esters with Polyfluoroaryl Nucleophiles

*Xiangli Yi, Runze Mao, Lara Lavrencic, and Xile Hu**

anie_202108465_sm_miscellaneous_information.pdf

Table of Content

1. General information S2

2. Detailed optimizations of the reaction S3

3. Synthesis of substrates S8

4. Synthesis and characterizations of products S18

5. Mechanistic study S36

5.1 UV-Vis study S36

5.2 Cyclic voltammetry study of the reaction reagents S36

5.3 Stern-Volmer quenching S37

6. NMR spectra of products and selected substrates S39

References S162

1. General information

Anhydrous dichloromethane (DCM), tetrahydrofuran (THF) and acetonitrile were prepared with solvent drying system. Purchased anhydrous solvents [DMA (N,N-dimethylacetamide) and AcOEt] and commercially available reagents were used without purification. 15 mL Teflon-screw capped vials (d = 2 cm) were used for the photochemical reactions. Blue LEDs (From Kessil Co., Ltd., 40 W max., wavelength centered at 460 nm, product No. A160WE) was used as the radiation source and a cooling fan was used to keep the reaction at aound room temperature during photolysis. After the reaction, GC-MS (Aglilent Tech. 7890B-5977B) was used to to determine the yield of the products for reaction optimizaiton, with 1,3,5-trimethylbenzene as an internal standard. Silica gel columns were used for reaction separation, with hexane-AcOEt as eluent unless otherwise noted. NMR spectra [^{1}H, ^{19}F (^{1}H decoupled), ^{13}C (^{1}H-decoupled)] were recorded on Bruker Avance 400 MHz instrument. Chemical shifts are reported in ppm in reference to the residual signal of $CDCl_3$, ^{1}H (7.26 ppm) and ^{13}C (77.16 ppm). Descriptions of multiplicities are abbreviated as follows: s = singlet, d = doublet, t = triplet, q = quartet, m = multiplet, b = broad. Determinations of high resolution mass spectra (HRMS) of unknown compounds by ESI, APPI or EI ionization technique were performed with a Micro Mass QTOF Ultima spectrometer at the EPFL Mass Spectrometry Center or ETH MoBiAS center. Stern-Volmer quenching experiments were conducted with a Varian Cary Eclipse fluorescence spectrophotometer. UV-Vis spectra were recorded on Cary 60 UV-Vis (Agilent Tech.). Cyclic voltammograms (CV) were recorded on a BIO-LOGIC VSP.

2. Detailed optimizations of the reaction

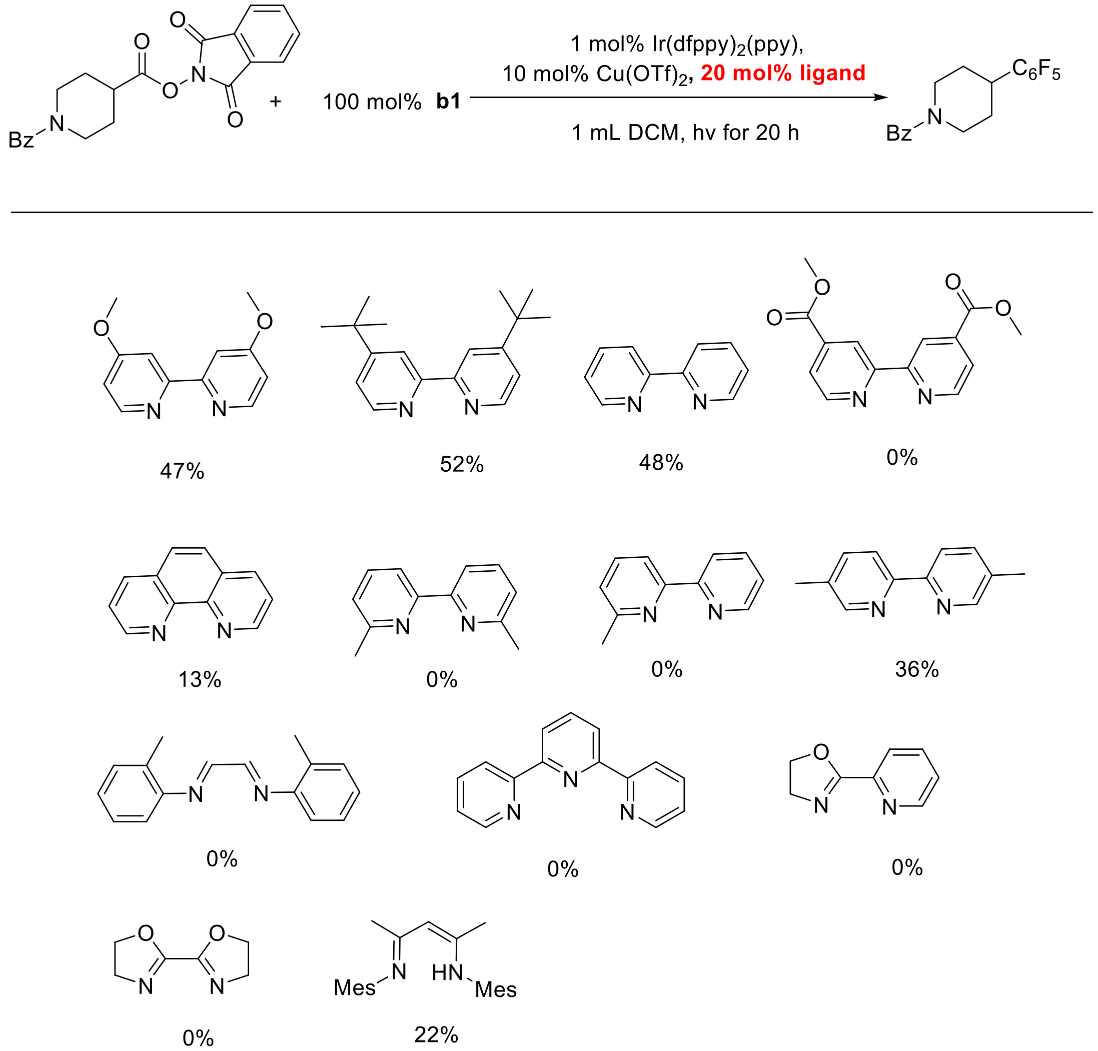

Figure S1. Screening of Ligands. Reaction conditions: 0.1 mmol **a1**, 0.1 mmol **b1** and other additives in 1 mL DCM. Reaction under Blue LED for 20 h.

Table S1. Screening of the type and volume of solvents

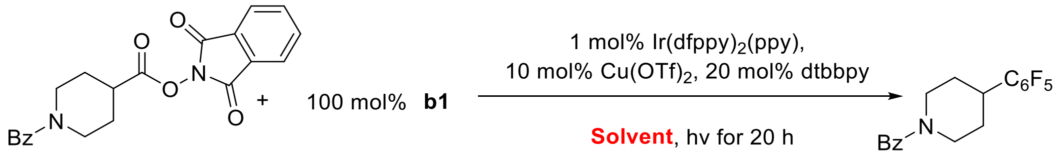

Entry	Solvent	Volume/ mL	Yield/ %
**1**	DCM	3	22
**2**	DCM	1	52
**3**	DCM	0.75	62
**4**	DCM	0.5	68
**5**	Acetone	0.5	84
**6**	PhCl	0.5	82
**7**	DCE	0.5	88
**8**	EtOAc	0.5	95
**9**	DMA	0.5	0

Reaction conditions: 0.1 mmol **a1**, 0.1 mmol **b1** and other additives in the given solvent. Reaction under Blue LED for 20 h.

Table S2. Screening of photocatalysts.

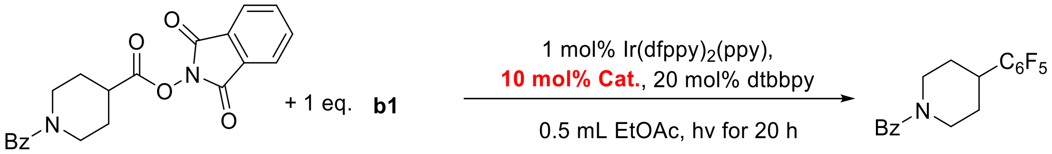

Entry	Metal Cat.	Yield/ %
**1**	Cu(OTf)$_2$	95
**2**	Cu(OPiv)$_2$	94
**3**	CuBr	75
**4**	Cu(CH$_3$CN)$_4$BF$_4$	83
**5**	Fe(OTf)$_2$	15
**6**	-	8
**7**	NiCl$_2$(DME)	0

Conditions: 0.1 mmol **a1**, 0.1 mmol **b1** and other additives in the given solvent. Reaction under Blue LED for 20 h.

For entry 6, the origin of the 8% yield of the product was not clear yet. A hypothesis was made below. The excited iridium photocatalyst reduces the NHPI ester to generate an alkyl radical after decarboxylation. The radical adds to the aryl of the zinc reagent to form an anionic radical, which is then oxidized by the photocatalyst to give the product. The excited state of 4CzIPN does not reduce NHPI ester directly (according to the Stern-Volmer quenching in Figure S5), which is consistent with the 0% yield in Entry 8, Table 1.

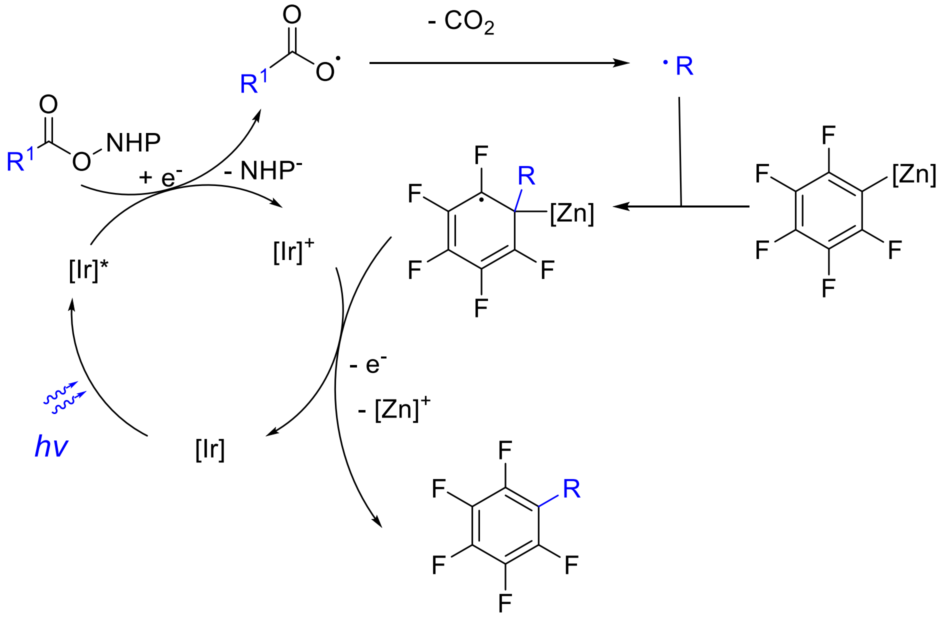

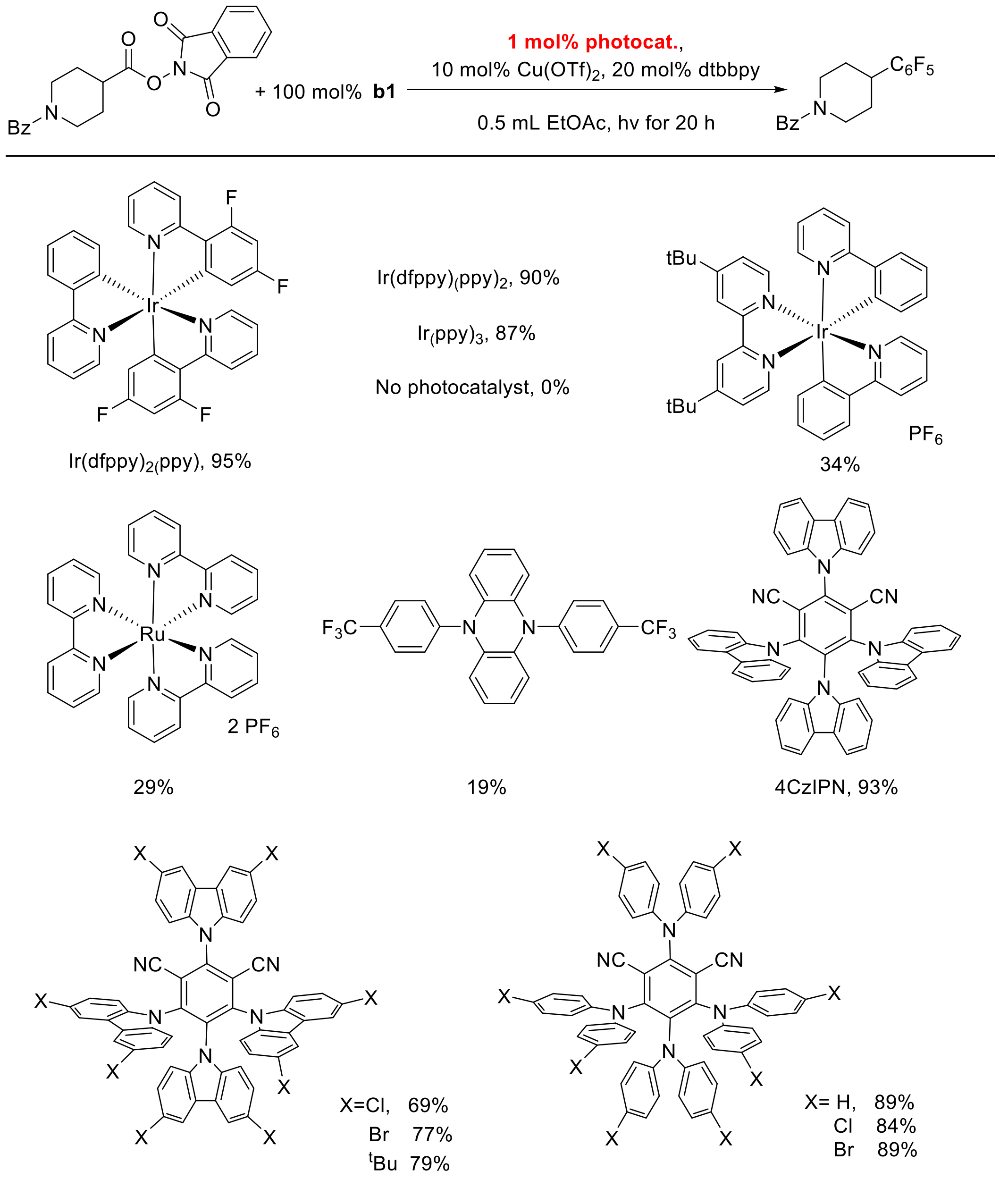

Figure S2. Screening of photocatalysts. Conditions: 0.1 mmol **a1**, 0.1 mmol **b1** and other additives in the given solvent. Reaction under Blue LED for 20 h.

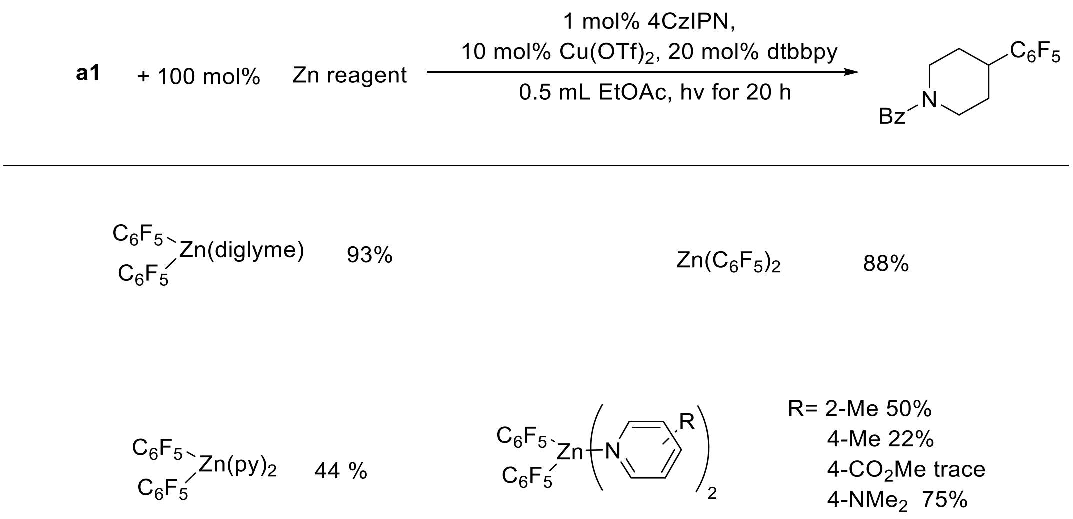

Figure S3. Optimization of the zinc reagents. Conditions: 0.1 mmol **a1**, 0.1 mmol zinc reagents and other additives in the given solvent. Reaction under Blue LED for 20 h.

3. Synthesis of the substrates.

Preparation of NHPI esters of carboxylic acids

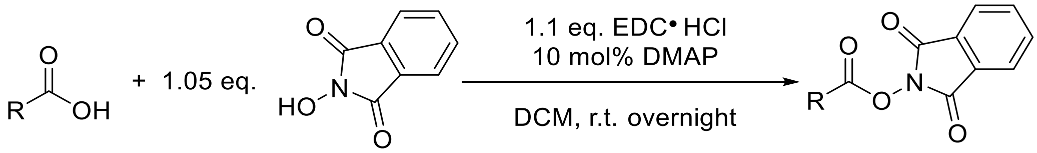

A round-bottom flask was charged with 5 mmol carboxylic acid, 1.05 eq. N-hydroxyphthalimide and 10 mol% of DMAP. 10 mL DCM was added and the stirring was started. Then, 1.1 eq. EDC·HCl (EDC= (1-ethyl-3-(3-dimethylaminopropyl)carbodiimide)dispersed in 5 mL DCM was added slowly to the flask. During the addition, the solid N-hydroxyphthalimide was dissolved gradually. The reaction was left overnight. Afterwards, the reaction mixture was diluted with DCM and washed 3 times with H_2O. The organic phase was collected, dried over Na_2SO_4 and concentrated using an evaporator. The residual was separated on a silica gel column with hexane-EtOAc as eluent. The NMR data for the following NHPI esters could be found in the corresponding literature.[1-5]

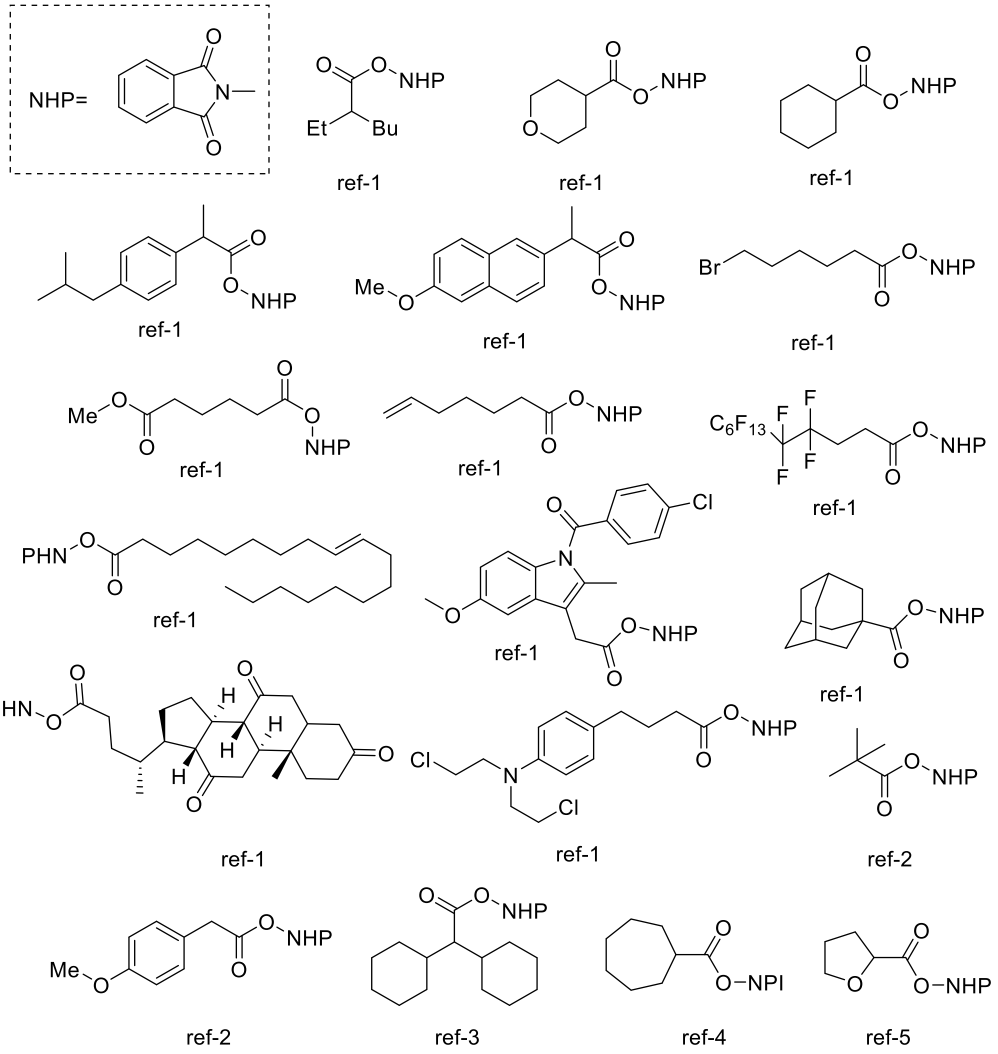

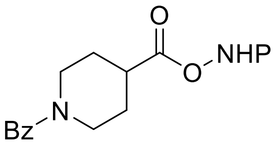

White solid. ^{1}H NMR (400 MHz, $CDCl_3$) δ 7.82 (m, 2H), 7.73 (m, 2H), 7.34 (m, 5H), 4.41 (b, 1H), 3.74 (m, 1H), 3.18 (ddd, *J* = 13.7, 10.3, 3.2 Hz, 2H), 2.98 (tt, *J* = 10.0, 4.2 Hz, 1H), 2.29 – 1.74 (m, 4H). ^{13}C NMR (101 MHz, $CDCl_3$) δ 170.7, 170.5, 162.0, 135.8, 135.0, 129.9, 129.0, 128.7, 127.0, 124.2, 38.6, 23.5.

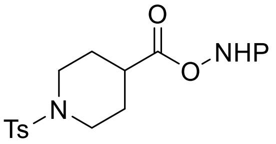

White solid. ^{1}H NMR (400 MHz, $CDCl_3$) δ 7.80 (dd, *J* = 5.5, 3.1 Hz, 1H), 7.75 – 7.70 (m, 1H), 7.65 – 7.55 (m, 1H), 7.27 (d, *J* = 8.0 Hz, 1H), 3.57 (dt, *J* = 12.1, 4.4 Hz, 1H), 2.72 – 2.53 (m, 2H), 2.37 (s, 2H), 2.09 (dq, *J* = 12.8, 3.8 Hz, 1H), 2.01 – 1.91 (m, 1H). ^{13}C NMR (101 MHz, $CDCl_3$) δ 170.3, 143.9, 135.0, 133.3, 129.9, 129.0, 127.8, 124.2, 45.0, 37.7, 27.4, 21.7.

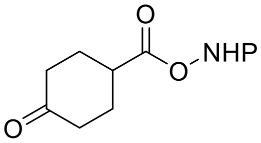

White solid. ^{1}H NMR (400 MHz, $CDCl_3$) δ 7.93 – 7.88 (m, 2H), 7.82 (m, 2H), 3.20 (tt, *J* = 8.3, 4.2 Hz, 1H), 2.63 (m, 2H), 2.50 – 2.22 (m, 5H). ^{13}C NMR (101 MHz, $CDCl_3$) δ 209.0, 170.6, 162.1, 135.0, 129.0, 124.2, 39.3, 38.1, 28.4.

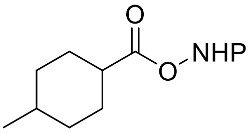

White solid. ^{1}H NMR (400 MHz, $CDCl_3$) δ 7.88 (dd, *J* = 5.5, 3.1 Hz, 2H), 7.78 (dd, *J* = 5.5, 3.1 Hz, 2H), 2.62 (tt, *J* = 12.2, 3.6 Hz, 1H), 2.22 – 2.12 (m, 2H), 1.93 – 1.78 (m, 2H), 1.62 (qd, *J* = 13.2, 3.5 Hz, 3H), 1.47 – 1.35 (m, 1H), 1.11 – 0.95 (m, 2H), 0.92 (d, *J* = 6.5 Hz, 3H). ^{13}C NMR (101 MHz, $CDCl_3$) δ 172.2, 162.2, 134.8, 129.2, 124.0, 40.7, 34.1, 31.9, 29.0, 22.5.

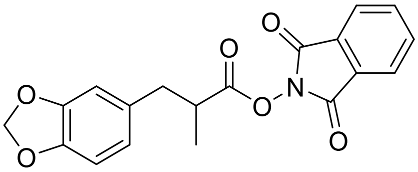

White solid. ^{1}H NMR (400 MHz, $CDCl_3$) δ 7.94 – 7.84 (m, 2H), 7.83 – 7.74 (m, 2H), 6.81 – 6.68 (m, 3H), 5.95 (s, 2H), 3.16 (dd, *J* = 13.7, 6.4 Hz, 1H), 3.09 – 3.01 (m, 1H), 2.77 (dd, *J* = 13.6, 7.7 Hz, 1H), 1.33 (d, *J* = 6.9 Hz, 3H). ^{13}C NMR (101 MHz, $CDCl_3$) δ 172.2, 162.1, 147.9, 146.5, 134.9, 131.7, 129.1, 124.1, 122.4, 109.6, 108.4, 101.1, 39.3, 39.1, 16.5.

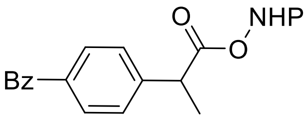

White solid. ^1H NMR (400 MHz, $CDCl_3$) δ 7.90 – 7.75 (m, 8H), 7.67 (dt, *J* = 7.8, 1.5 Hz, 1H), 7.63 – 7.57 (m, 1H), 7.56 – 7.47 (m, 3H), 4.20 (q, *J* = 7.1 Hz, 1H), 1.71 (d, *J* = 7.2 Hz, 3H). ^{13}C NMR (101 MHz, $CDCl_3$) δ 196.5, 170.6, 138.7, 138.4, 137.5, 134.9, 132.7, 131.7, 130.3, 129.8, 129.5, 129.1, 129.0, 128.5, 124.1, 43.0, 19.0.

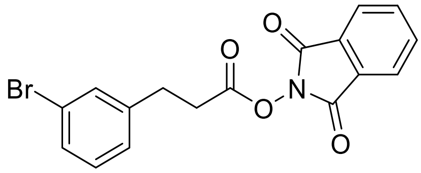

White solid. ^1H NMR (400 MHz, $CDCl_3$) δ 7.92 – 7.83 (m, 2H), 7.82 – 7.73 (m, 2H), 7.43 – 7.33 (m, 2H), 7.22 – 7.17 (m, 2H), 3.06 (t, *J* = 7.0 Hz, 2H), 2.97 (t, *J* = 7.7 Hz, 2H). ^{13}C NMR (101 MHz, $CDCl_3$) δ 168.7, 161.9, 141.5, 134.9, 131.5, 130.4, 130.0, 128.9, 127.1, 124.1, 122.8, 32.5, 30.2.

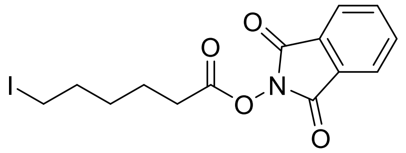

White solid. ^1H NMR (400 MHz, $CDCl_3$) δ 7.93 – 7.84 (m, 2H), 7.84 – 7.75 (m, 2H), 3.21 (t, *J* = 6.9, 2H), 2.69 (t, *J* = 7.4, 2H), 1.95 – 1.76 (m, 4H), 1.58 (m, 2H). ^{13}C NMR (101 MHz, $CDCl_3$) δ 169.5, 162.1, 134.9, 129.1, 124.1, 33.1, 30.9, 29.8, 23.8, 6.2.

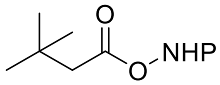

White solid. ^1H NMR (400 MHz, $CDCl_3$) δ 7.88 (dd, *J* = 5.5, 3.1 Hz, 2H), 7.83 – 7.74 (m, 2H), 2.53 (s, 2H), 1.16 (s, 9H). ^{13}C NMR (101 MHz, $CDCl_3$) δ 168.0, 162.2, 134.8, 129.1, 124.1, 44.7, 31.4, 29.6.

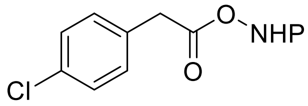

White solid. ^1H NMR (400 MHz, $CDCl_3$) δ 7.87 (m, 2H), 7.78 (m, 2H), 7.40 – 7.28 (m, 4H), 3.96 (s, 2H). ^{13}C NMR (101 MHz, $CDCl_3$) δ 167.4, 161.9, 135.0, 134.0, 130.8, 130.1, 129.2, 128.9, 124.1, 37.2.

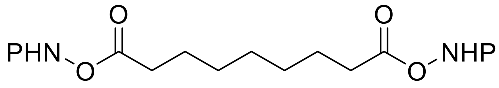

White solid. ^1H NMR (400 MHz, $CDCl_3$) δ 7.87 (m, 4H), 7.78 (m, 4H), 2.67 (td, *J* = 7.4 Hz, 4H), 1.80 (p, *J* = 7.4 Hz, 4H), 1.51 – 1.36 (m, 6H). ^{13}C NMR (101 MHz, $CDCl_3$) δ 169.7, 162.1, 134.8, 129.1, 124.1, 31.0, 28.7, 28.6, 24.7.

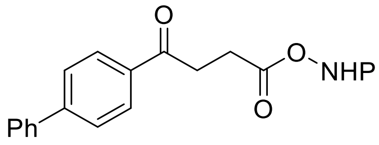

White solid. ^1H NMR (400 MHz, $CDCl_3$) δ 8.12 – 8.04 (m, 2H), 7.90 (m, 2H), 7.79 (m, 2H), 7.74 – 7.67 (m, 2H), 7.67 – 7.59 (m, 2H), 7.52 – 7.44 (m, 2H), 7.44 – 7.36 (m, 1H), 3.51 (t, *J* = 6.9 Hz, 2H), 3.18 (t, *J* = 6.9 Hz, 2H). ^{13}C NMR (101 MHz, $CDCl_3$) δ 196.2, 169.5, 162.0, 146.3, 139.9, 134.9, 129.1, 129.1, 128.9, 128.5, 127.5, 127.4, 124.1, 33.4, 25.6.

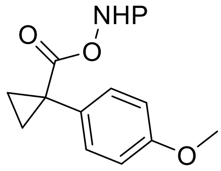

White solid. ^{1}H NMR (400 MHz, $CDCl_3$) δ 7.84 (m, 2H), 7.75 (m, 2H), 7.44 (d, *J* = 8.8 Hz, 2H), 6.89 (d, *J* = 8.7 Hz, 2H), 3.80 (s, 3H), 1.87 (q, *J* = 4.3 Hz, 2H), 1.45 (q, *J* = 4.4 Hz, 2H). ^{13}C NMR (101 MHz, $CDCl_3$) δ 171.4, 162.0, 159.3, 134.8, 131.8, 129.2, 129.1, 124.0, 114.0, 55.4, 26.7, 19.0.

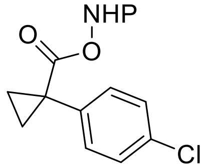

White solid. ^{1}H NMR (400 MHz, $CDCl_3$) δ 7.85 (m, 2H), 7.76 (m, 2H), 7.52 – 7.41 (m, 2H), 7.38 – 7.31 (m, 2H), 1.98 – 1.86 (m, 2H), 1.53 – 1.41 (m, 2H). ^{13}C NMR (101 MHz, $CDCl_3$) δ 170.8, 161.9, 135.6, 134.9, 134.0, 132.1, 129.0, 128.8, 124.0, 26.8, 18.9.

Preparation of (diglyme)Zn(C_6F_5)$_2$ (b1)

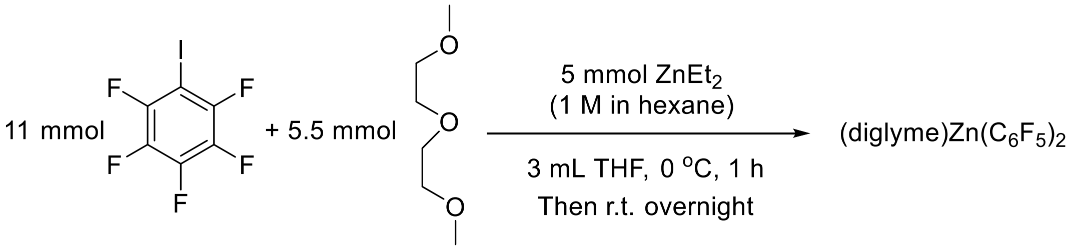

A 50-mL Schlenk Tube was charged with 11 mmol pentafluoroiodobenzene, 5.5 mmol diglyme and 3 mL THF under nitrogen. The reaction vessel was cooled to 0 °C, and diethyl zinc (1 M in hexane) was gradually added to the stirred solution. The reaction was maintained at 0 °C for 1 h, and then at room temperature overnight. A white precipitate was observed. The solvent was removed under vacuum, and the residual was washed with hexane and then dried again under vacuum to give fine white powder in 50-60 % yield. In case where there is some impurities observed on NMR spectrum, recrystallization in DCM-Et_2O could give pure crystals. ^{1}H NMR (400 MHz, $CDCl_3$) δ 3.94 (t, *J* = 4.9 Hz, 4H), 3.66 (t, *J* = 4.9Hz, 4H), 3.24 (s, 6H). ^{19}F NMR (376 MHz, $CDCl_3$) δ -117.6 (m), -157.3 (m), -162.13 (dt, *J* = 18.7, 10.6 Hz). ^{13}C NMR (101 MHz, $CDCl_3$) δ 149.1 (dd, *J* = 226.0, 26.9 Hz), 139.8 (d, *J* = 246.6 Hz), 136.6 (dd, *J* = 253.1, 31.8 Hz), 119.3, 70.31, 69.6, 58.5.

Preparation of LZn(C_6F_5)$_2$

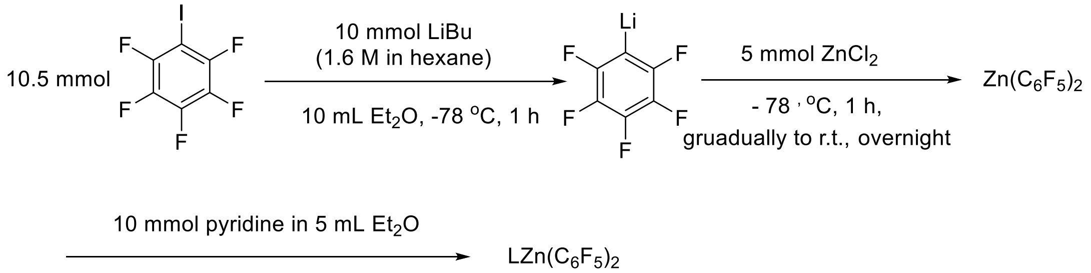

A 50-mL Schlenk Tube was charged with 10.5 mmol pentafluoroiodobenzene, 10 mL Et_2O under nitrogen. The reaction vessel was cooled to - 78 °C, and 6.25 mL Li^{n}Bu (1.6 M in hexane) was added to the stirred solution by a syringe pump in 10 min. The reaction was maintained at - 78 °C for 1 h. Then, 5 mmol $ZnCl_2$ was added to the solution, which was maintained at - 78 °C for another one hour. The reaction was allowed to gradually warm to room temperature overnight. A white precipitation was observed. Filtration under nitrogen removed the white solid to obtain clear solution. 10 mmol pyridine in 5 mL Et_2O was added to the solution. Both an amorphous white solid (possibly LiCl) and crystals (LZn(C_6F_5)$_2$) were formed. DCM was added to dissolve the crystals, and the amorphous precipitate was removed by filtration. The obtained solution was concentrated under vacuum to give yellowish solid, which can be recrystallized using DCM-Et_2O.

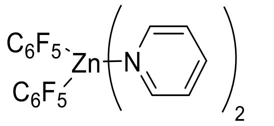

^{1}H NMR (400 MHz, $CDCl_3$) δ 8.52 (dt, *J* = 4.6, 1.6 Hz, 4H), 7.94 (tt, *J* = 7.7, 1.7 Hz, 2H), 7.54 – 7.46 (m, 4H). ^{19}F NMR (376 MHz, $CDCl_3$) δ -116.3 (m), -157.7 (t, *J* = 19.6 Hz), -162.0 (m). ^{13}C NMR (101 MHz, $CDCl_3$) δ 149.2, 139.4, 125.5.

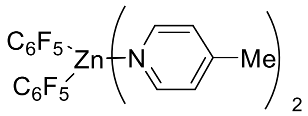

^{1}H NMR (400 MHz, $CDCl_3$) δ 8.35 (d, *J* = 5.3 Hz, 4H), 7.25 (d, *J* = 5.2 Hz, 4H), 2.43 (s, 6H). ^{19}F NMR (376 MHz, $CDCl_3$) δ -116.3 (m), -158.0 (t, *J* = 19.4 Hz), -162.2 (m). ^{13}C NMR (101 MHz, $CDCl_3$) δ 148.8, 126.1, 21.5.

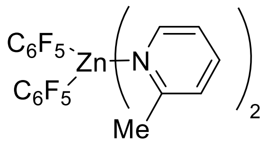

^{1}H NMR (400 MHz, $CDCl_3$) δ 8.44 (d, *J* = 5.4 Hz, 2H), 7.79 (t, *J* = 7.8 Hz, 2H), 7.31 (d, *J* = 7.9 Hz, 2H), 7.28 – 7.23 (m, 2H), 2.53 (s, 6H). ^{19}F NMR (376 MHz, $CDCl_3$) δ -117.4 (m), -158.1 (t, *J* = 19.4 Hz), -161.6 (m). ^{13}C NMR (101 MHz, $CDCl_3$) δ 159.3, 149.0, 139.1, 126.1, 122.2, 24.0.

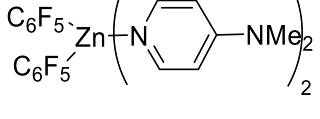

^{1}H NMR (400 MHz, $CDCl_3$) δ 8.03 (d, *J* = 6.2 Hz, 4H), 6.49 (d, *J* = 6.2 Hz, 4H), 3.03 (s, 12H). ^{19}F NMR (376 MHz, $CDCl_3$) δ -116.2 (m), -159.1 (t, *J* = 19.4 Hz), -162.6 (m). ^{13}C NMR (101 MHz, $CDCl_3$) δ 155.1, 148.7, 106.8, 39.2.

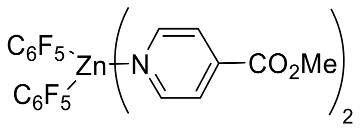

^{1}H NMR (400 MHz, $CDCl_3$) δ 8.80 – 8.62 (m, 4H), 8.14 – 7.97 (m, 4H), 4.00 (s, 6H). ^{19}F NMR (376 MHz, $CDCl_3$) δ -116.4 (m), -156.88 (t, *J* = 19.1 Hz), -161.6 (m). ^{13}C NMR (101 MHz, $CDCl_3$) δ 164.2, 150.0, 140.4, 124.9, 53.4.

Preparation of [(bpy)Cu(C_6F_5)]

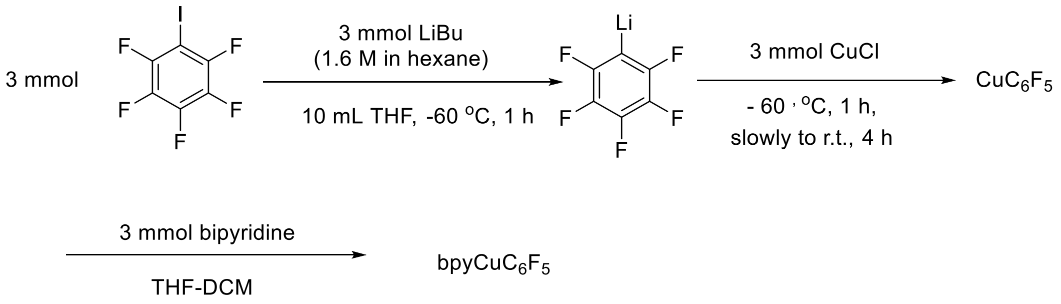

A 50-mL Schlenk tube was charged with 3 mmol pentafluoroiodobenzene and 10 mL THF under nitrogen. The reaction vessel was cooled to - 78 °C, and 1.9 mL Li^{n}Bu (1.6 M in hexane) was gradually added to the stirred solution by a syringe pump in 5 min. The reaction was maintained at - 60 °C for 1 h (using an acetone – dry ice bath). Then, 3 mmol CuCl was added to the solution, which was maintained at - 60 °C for another one hour. The reaction was allowed to slowly warm to room temperature in 4 h (the increase of temperature in acetone – dry ice bath can be pretty slow when the Dewar is covered with cotton layer). Then, the solution was filtered and concentrated under vacuum. The residual was dissolved in 3 mL DCM. 3 mmol bipyridine dissolved in 3 mL THF was added dropwise to the CuC_6F_5 solution, and a dark red solution was obtained. The solution was placed under – 20 °C for 2 h and red crystals formed. By decanting the upper solution, washing the solid with Et_2O and finally drying under vacuum, the product could be obtained. The NMR Data matched with the reported data.[6]
^{1}H NMR (400 MHz, CD_2Cl_2) δ 9.03 – 8.82 (m, 2H), 8.18 (d, *J* = 8.1 Hz, 2H), 8.05 (tt, *J* = 7.9, 1.6 Hz, 2H), 7.68 – 7.50 (m, 2H). ^{19}F NMR (376 MHz, CD_2Cl_2) δ -109.69 (d, *J* = 38.0 Hz), -162.97 (t, *J* = 20.0 Hz), -163.69 (m).

Preparation of Zn(Ar_F)$_2$ (as solution in EtOAc), method 1 (with Li^{n}Bu)

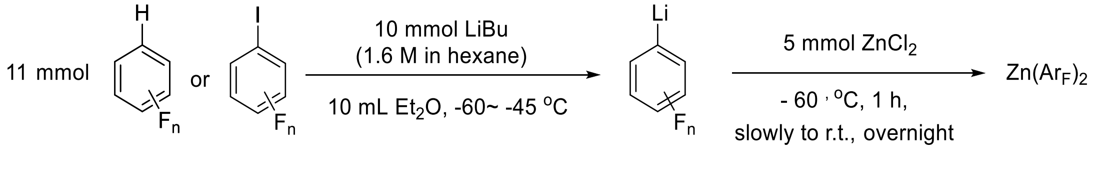

A 100-mL Schlenk tube was charged with 11 mmol polyfluoroarene and 20 mL THF under nitrogen. The reaction vessel was cooled to - 78 °C, and 6.25 mL Li^{n}Bu (1.6 M in hexane) was gradually added to the stirred solution by a syringe pump in 10 min. The reaction was maintained at - 60 ~ - 45 °C for a given period of time. Then, 5 mmol $ZnCl_2$ was added to the solution, which was maintained at - 60 °C for another one hour. The reaction was allowed to gradually warm to room temperature overnight. The solvent was removed under vacuum, and then the product was heated under vacuum for 2 h to remove the remaining solvent. A white or yellowish solid was obtained, which was dispersed in 10 mL DCM to give a suspension. Filtration was used to remove the solid and the solvent was again removed under vacuum. The residual was dissolved in 5 mL EtOAc and the concentration of $Zn(Ar_F)_2$ was determined using NMR, with pentafluorobenzene or 1,2,3,4-tetrafluorobenzene as internal standard.

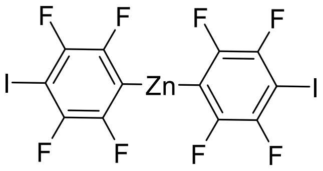

Prepared from 2,3,5,6 -tetrafluoro-1,4-diiodobenzene, 1 h for the first step. ^{19}F NMR (376 MHz, EtOAc-$CDCl_3$) δ -117.10 (m), -122.7 (m). $C(Ar_F)$ = 1.6 M.

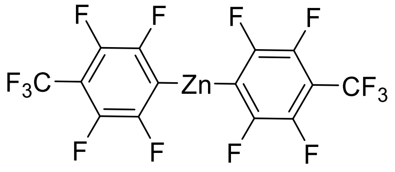

Prepared from 1,2,4,5-tetrafluoro-3-(trifluoromethyl)benzene, 2 h for the first step. ^{19}F NMR (376 MHz, EtOAc-$CDCl_3$) δ -56.4(m), -117.1(m), -143.8(m). $C(Ar_F)$ = 0.60 M.

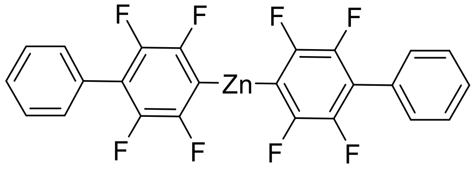

Prepared from 2,3,5,6-tetrafluoro-1,1'-biphenyl, 2 h for the first step. ^{19}F NMR (376 MHz, EtOAc-$CDCl_3$) δ -119.7(m), -145.5(m). $C(Ar_F)$ = 0.68 M.

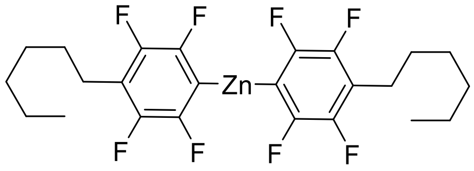

Prepared from 1,2,4,5-tetrafluoro-3-hexylbenzene, 3 h for the first step. ^{19}F NMR (376 MHz, EtOAc-$CDCl_3$) δ -120.9 (m), -144.9 (m). $C(Ar_F)$ = 0.60 M.

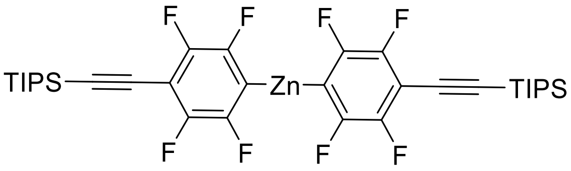

Prepared from triisopropyl((2,3,5,6-tetrafluorophenyl)ethynyl)silane, 3 h for the first step. ^{19}F NMR (376 MHz, EtOAc-$CDCl_3$) δ -119.7 (m), -137.7 (m). C(Ar_F) = 1.2 M.

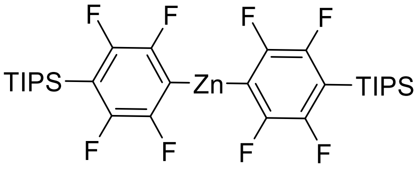

Prepared from triisopropyl((2,3,5,6-tetrafluorophenyl)ethynyl)silane, 3 h for the first step. ^{19}F NMR (376 MHz, EtOAc-$CDCl_3$) δ -119.3 (m), -128.4 (m). C(Ar_F) = 0.62 M.

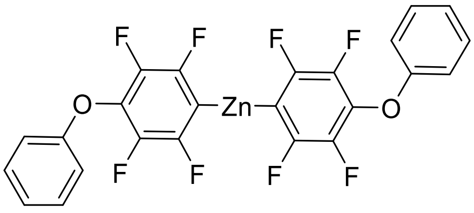

Prepared from triisopropyl((2,3,5,6-tetrafluorophenyl)ethynyl)silane, 3 h for the first step. ^{19}F NMR (376 MHz, EtOAc-$CDCl_3$) δ -118.0 (m), -156.3 (m). C(Ar_F) = 0.64 M.

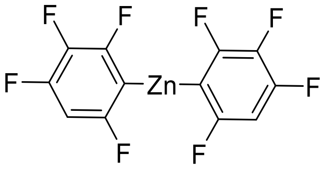

Prepared from 1,2,3,5-tetrafluorobenzene, 2 h for the first step. ^{1}H NMR (400 MHz, EtOAc-$CDCl_3$) δ 6.43 – 6.28 (m, 1H). ^{19}F NMR (376 MHz, EtOAc-$CDCl_3$) δ -93.6 (d, J = 13.9 Hz), -113.6 (d, J = 29.7 Hz), -137.9 (m), -169.0 (m). C(Ar_F) = 1.5 M.

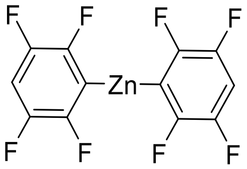

Prepared from 1,2,4,5-tetrafluorobenzene, 2 h for the first step. ^{1}H NMR (400 MHz, EtOAc-$CDCl_3$) δ 6.70 (m, 1H). ^{19}F NMR (376 MHz, EtOAc-$CDCl_3$) δ -120.5 (m), -140.8 (m). C(Ar_F) = 0.9 M.

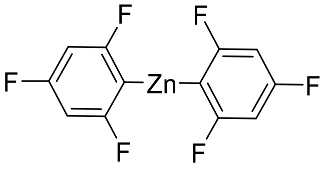

Prepared from 1,3,5-trifluorobenzene, 4 h for the first step. ^{1}H NMR (400 MHz, EtOAc-$CDCl_3$) δ 6.39 (dd, *J* = 9.5, 5.1 Hz, 1H). ^{19}F NMR (376 MHz, EtOAc-$CDCl_3$) δ -87.5 (m), -113.6 (m). C(Ar_F) = 1.9 M.

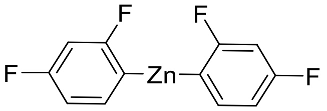

Prepared from 2,4-difluoro-1-iodobenzene, 2 h for the first step. ^{1}H NMR (400 MHz, EtOAc-$CDCl_3$) δ 7.33 (m, 1H), 6.68 (m, 1H), 6.55 (m, 1H). ^{19}F NMR (376 MHz, EtOAc-$CDCl_3$) δ -88.6, -114.7 (m). C(Ar_F) = 1.2 M.

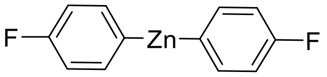

Prepared from 4-fluoro-1-iodobenzene, 2 h for the first step. ^{1}H NMR (400 MHz, EtOAc-$CDCl_3$) δ 7.51 (t, *J* = 7.5 Hz, 2H), 6.89 (m, 2H). ^{19}F NMR (376 MHz, EtOAc-$CDCl_3$) δ -115.38. C(Ar_F) = 1.2 M.

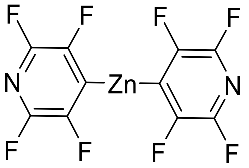

Prepared from 2,3,5,6 -tetrafluoropyridine with a different purification process (the product has poor solubility in DCM): After the reaction, the suspension was filtrated to get the solid. The solid was washed with THF and then DCM, and dried under vacuum. A white powder was obtained. Dissolving part of the powder in EtOAc led to a clear solution. ^{19}F NMR (376 MHz, EtOAc-$CDCl_3$) δ -98.39, -122.93. C(Ar_F) = 0.5 M.

Preparation of Zn(Ar_F)$_2$ (as solution in EtOAc), method 2 (with Li^{t}Bu)

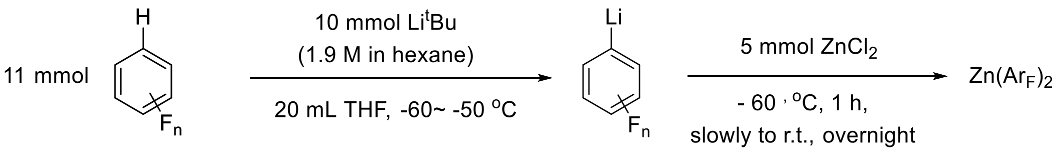

A 100-mL Schlenk tube was charged with 11 mmol polyfluoroarene and 20 mL THF under nitrogen. The reaction vessel was cooled to -100 °C, and 5.3 mL Li^{t}Bu (1.9 M in hexane) was added to the stirred solution by a syringe pump in 10 min. The reaction was maintained at -60 ~ - 50 °C for 3 h. Then, 5 mmol $ZnCl_2$ was added to the solution, which was maintained at -60 °C for another one hour. The reaction was allowed to gradually warm to room temperature overnight. A clear solution was obtained. The solvent was removed under vacuum, and then the product was heated under vacuum for 2 h to remove the remaining solvent. A white or yellowish solid was obtained, which was dispersed in 10 mL DCM to give a suspension. Filtration was used to remove the solid and the solvent was again removed under vacuum. The

residual was dissolved in 5 mL EtOAc and the concentration of $Zn(Ar_F)_2$ was determined using NMR, with pentafluorobenzene or 1,2,3,4-tetrafluorobenzene as internal standard.

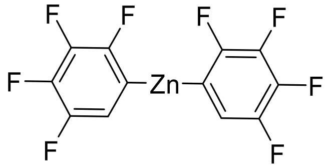

Prepared from 1,2,3,4 -tetrafluorobenzene. ^{1}H NMR (400 MHz, EtOAc-$CDCl_3$) δ 6.88 (m, 1H). ^{19}F NMR (376 MHz, EtOAc-$CDCl_3$) δ -118.31 (m), -142.25 (m), -158.5 (m), -160.74 (m). C(Ar_F) = 1.5 M.

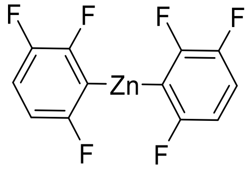

Prepared from 1,2,4-trifluorobenzene. ^{1}H NMR (400 MHz, EtOAc-$CDCl_3$) δ 6.79 – 6.72 (m, 1H), 6.49 (m, 1H). ^{19}F NMR (376 MHz, EtOAc-$CDCl_3$) δ -95.8, -114.6, -145.9. C(Ar_F) = 1.6 M.

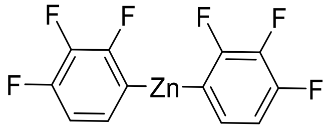

Prepared from 1,2,3-trifluorobenzene. ^{1}H NMR (400 MHz, EtOAc-$CDCl_3$) δ 7.05 (m, 1H), 6.75 (m, 1H). ^{19}F NMR (376 MHz, EtOAc-$CDCl_3$) δ -115.0 (dd, *J* = 31.1, 7.1 Hz), -141.0 (dd, *J* = 18.4, 7.1 Hz), -165.1 (dd, *J* = 31.1, 18.4 Hz). C(Ar_F) = 1.5 M.

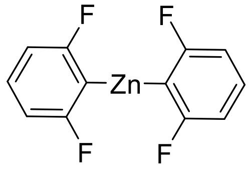

Prepared from 1, 3-difluorobenzene. ^{1}H NMR (400 MHz, EtOAc-$CDCl_3$) δ 7.04 (tt, *J* = 7.7, 6.6 Hz, 1H), 6.62 (m, 2H). ^{19}F NMR (376 MHz, EtOAc-$CDCl_3$) δ -89.2. C(Ar_F) = 1.8 M.

.

4. Synthesis and characterizations of products.

General procedure 1 (GP1):

0.1 mmol NHPI ester (**a**), 0.02 mmol dtbbpy and 0.001 mmol 4CzIPN were added to a glass vial (d=2 cm, 15 mL), which was transferred to the glovebox. 0.01 mmol $Cu(OTf)_2$ and 0.5 mL EtOAc were added and a green solution was obtained. Then, 0.1 mmol **b1** was added to the solution, which instantly turned yellow. The vial was properly sealed and placed in the photochemical setup (shown in the picture below). After 20 h, the reaction was stopped. To the reaction solution were added ethyl acetate (4 mL), water (1 mL) and 3 drops of 15% aqueous ammonia for workup. The organic phase obtained by extraction was concentrated. The residue was subsequently analyzed by GC-MS&FID to assess the reaction yield and was separated by chromatography on silica gel to give the final product.

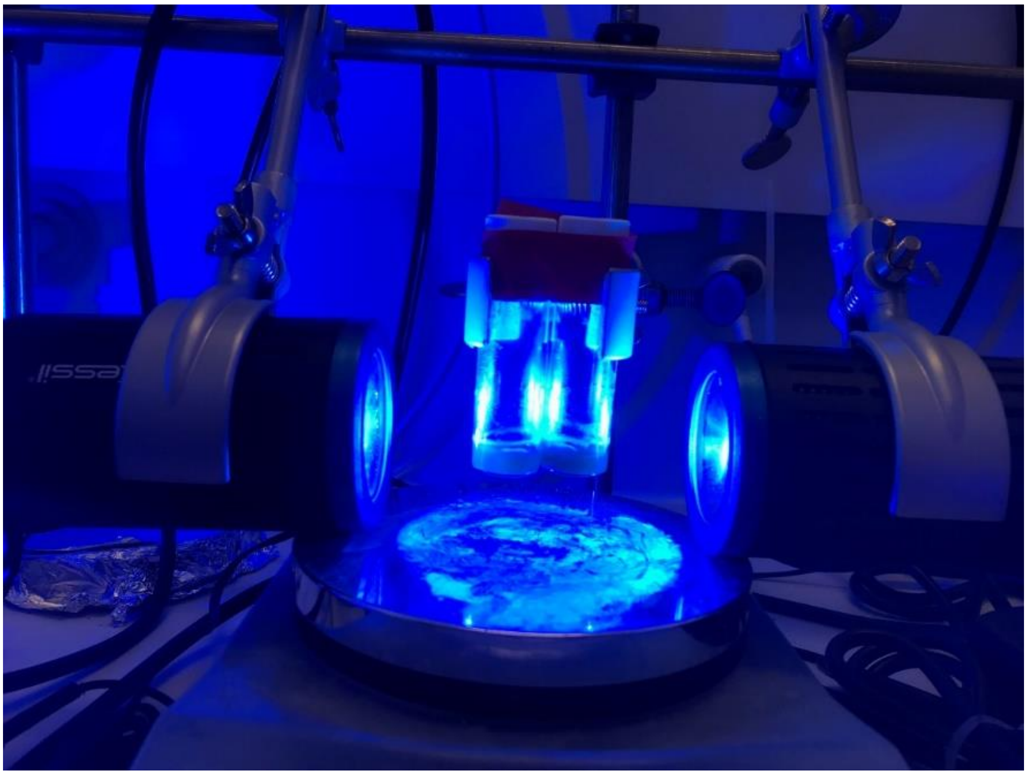

General procedure 2 (GP2):

0.1 mmol NHPI ester (**a**), 0.02 mmol dtbbpy and 0.001 mmol 4CzIPN were added to a glass vial (d=2 cm, 12 mL), which was transferred to the glovebox. 0.01 mmol $Cu(OTf)_2$, 0.1 mmol diglyme and (0.5-x) mL EtOAc were added and a green solution was obtained (x is the volume of $Zn(Ar_F)_2$ solution in EtOAc). Then, x mL of $Zn(Ar_F)_2$ solution (0.1 mmol) was added to the vial, and the mixture turned yellow. The vial was properly sealed and placed in the photochemical setup. After 20 h, the reaction was stopped. To the reaction solution were added ethyl acetate (4 mL), water (1 mL) and 3 drops of 15% aqueous ammonia for workup. The organic phase obtained by extraction was concentrated. The residue was subsequently analyzed by GC-MS&FID to assess the reaction yield and was separated by chromatography on silica gel to give the final product.

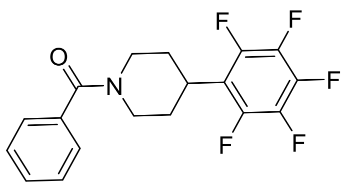

c1

(4-(Perfluorophenyl)piperidin-1-yl)(phenyl)methanone, synthesized via GP1, colorless crystal, m.p. 117-118 °C , 30.1 mg (Yield = 85%). ^{1}H NMR (400 MHz, $CDCl_3$) δ 7.42 (m, 5H), 4.91 (b, 1H), 3.90 (b, 1H), 3.26 (tt, *J* = 12.5, 3.7 Hz, 1H), 3.08 (b, 1H), 2.84 (b, 1H), 2.04 (b, 2H), 1.89 – 1.63 (b, 2H). ^{19}F NMR (376 MHz, $CDCl_3$) δ -142.7 (m), -156.7 (t, *J* = 20.7 Hz), -162.0 (td, *J* = 22.0, 7.8 Hz). ^{13}C NMR (101 MHz, $CDCl_3$) δ 170.6, 146.4(m), 144.0(m), 141.2(m), 138.9(m), 136.5(m), 136.0, 129.8, 128.7, 126.9, 117.3(m), 48.3, 42.9, 33.6, 33.6, 30.5. HRMS (ESI/QTOF) m/z: $[M + H]^+$ Calcd for $C_{18}H_{15}F_5NO^+$ 356.1068; Found 356.1065.

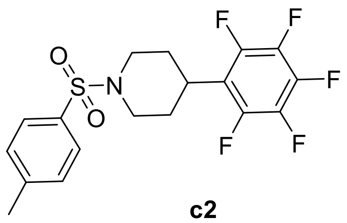

c2

4-(Perfluorophenyl)-1-tosylpiperidine, synthesized via GP1, colorless crystal, m.p. 147-148 °C , 23.1 mg (Yield = 57%). ^{1}H NMR (400 MHz, $CDCl_3$) δ 7.67 (d, *J* = 8.0 Hz, 2H), 7.35 (d, *J* = 8.0 Hz, 2H), 3.96 (d, *J* = 11.6, 2H), 2.88 (tt, *J* = 12.4, 3.6 Hz, 1H), 2.45 (s, 3H), 2.39 – 2.28 (m, 2H), 2.25 – 2.10 (m, 2H), 1.76 (d, *J* = 12.2 Hz, 2H). ^{19}F NMR (376 MHz, $CDCl_3$) δ -142.5 (m), -156.5 (t, *J* = 20.9 Hz), -161.9 (td, *J* = 21.6, 7.7 Hz). ^{13}C NMR (101 MHz, $CDCl_3$) δ 146.4(m), 144.1(m), 143.9, 141.2(m), 139.0(m), 136.5(m), 133.2, 129.9, 127.9, 117.1(m), 46.9, 32.8, 29.5, 29.5, 29.4, 21.7. GC-MS: 405. HRMS (ESI/QTOF) m/z: $[M + H]^+$ Calcd for $C_{18}H_{17}F_5NO_2S^+$ 406.0895; Found 406.0895.

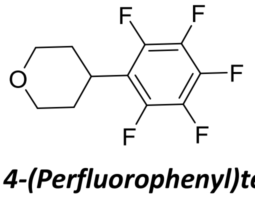

c3

4-(Perfluorophenyl)tetrahydro-2H-pyran, synthesized via GP1, colorless liquid, 18.8 mg (Yield = 67%). ^{1}H NMR (400 MHz, $CDCl_3$) δ 4.08 (dd, *J* = 11.7, 4.5 Hz, 2H), 3.50 (td, *J* = 11.9, 1.9 Hz, 2H), 3.24 (tt, *J* = 12.5, 3.8 Hz, 1H), 2.26 – 2.10 (m, 2H), 1.63 (dd, *J* = 12.9, 3.7 Hz, 2H). ^{19}F NMR (376 MHz, $CDCl_3$) δ -142.9 (m), -157.28 (t, *J* = 20.9 Hz), -162.37 (td, *J* = 21.5, 7.3 Hz). ^{13}C NMR (101 MHz, $CDCl_3$) δ 146.7(m), 144.1(m), 139.0(m), 138.6(m), 136.5(m), 117.9(m), 68.4, 32.6, 30.8(m). GC-MS: 252. HRMS (APPI/LTQ-Orbitrap) m/z: $[M + H_{-1}]^+$ Calcd for $C_{11}H_8F_5O^+$ 251.0490; Found 251.0496.

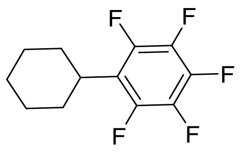

c4

1-Cyclohexyl-2,3,4,5,6-pentafluorobenzene,[7] synthesized via GP1, colorless liquid, 19.3mg (Yield = 77%). ^{1}H NMR (400 MHz, $CDCl_3$) δ 2.98 (m, 1H), 1.91 – 1.68 (m, 7H), 1.45 – 1.21 (m,

3H). ^{19}F NMR (376 MHz, $CDCl_3$) δ -143.0 (m), -158.7 (t, *J* = 20.6 Hz), -163.1 (td, *J* = 22.3, 7.4 Hz, 2F). ^{13}C NMR (101 MHz, $CDCl_3$) δ 146.5(m), 144.0(m), 140.6(m), 138.9(m), 138.1(m), 136.4(m), 119.9(m), 35.4, 31.1, 31.1, 31.1, 26.9, 25.8. GC-MS: 250.

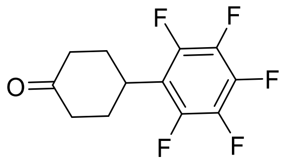

c5

4-(Perfluorophenyl)cyclohexan-1-one, synthesized via GP1, white solid, m.p. 115-117 °C , 19.4 mg (Yield = 73 %). ^{1}H NMR (400 MHz, $CDCl_3$) δ 3.48 (tt, *J* = 12.4, 3.6 Hz, 1H), 2.59 – 2.42 (m, 4H), 2.36 – 2.20 (m, 2H), 2.18 – 2.06 (m, 2H). ^{19}F NMR (376 MHz, $CDCl_3$) δ -142.6 (m), -156.6 (t, *J* = 21.2 Hz), -161.8 (m, 2F). ^{13}C NMR (101 MHz, $CDCl_3$) δ 209.4, 146.4(m), 144.0(m), 141.2(m), 139.1(m), 138.7(m), 136.6(m), 117.3(m), 41.3, 33.6, 30.6(m). GC-MS: 264. HRMS (APPI/LTQ-Orbitrap) m/z: $[M + H]^+$ Calcd for $C_{12}H_{10}F_5O^+$ 265.0646; Found 265.0651.

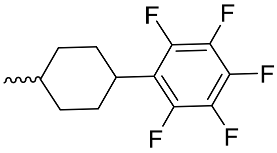

c6

1,2,3,4,5-Pentafluoro-6-(4-methylcyclohexyl)benzene, synthesized via GP1, colorless liquid, 20.1 mg (Yield = 76 %, dr = 1:2.6). ^{1}H NMR (400 MHz, $CDCl_3$) δ 2.94 (m, 1H), 2.11 – 1.95 (m, 1H), 1.92 – 1.56 (m, 6H), 1.56 – 1.39 (m, 1H), 1.14 – 0.90 (m, 4H). ^{19}F NMR (376 MHz, $CDCl_3$) δ -142.9 (major), -143.2 (minor), -158.6, -163.1. ^{13}C NMR (101 MHz, $CDCl_3$) δ (major &minor) 146.5(m), 144.0(m), 140.6(m), 138.9(m), 138.1(m), 136.4(m), 119.8(m), 35.4, 35.0, 32.1, 32.0, 30.9, 26.5, 25.0, 22.7, 17.3. GC-MS: 264. HRMS (nanochip-ESI/LTQ-Orbitrap) m/z: $[M + H_{-1}]^+$ Calcd for $C_{13}H_{12}F_5^+$ 263.0854; Found 263.0853.

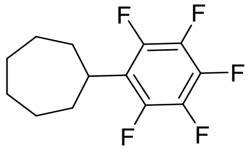

c7

(Perfluorophenyl)cycloheptane,[8] synthesized via GP1, colorless liquid, 14.6 mg (Yield = 55 %). ^{1}H NMR (400 MHz, $CDCl_3$) δ 3.11 (tt, *J* = 10.7, 3.3 Hz, 1H), 1.95 – 1.73 (m, 6H), 1.72 – 1.41 (m, 6H). ^{19}F NMR (376 MHz, $CDCl_3$) δ -143.0 (dd, *J* = 22.7, 7.9 Hz), -159.1 (t, *J* = 20.7 Hz), -162.9 – -163.4 (m). ^{13}C NMR (101 MHz, $CDCl_3$) δ 145.9(m), 143.5(m), 140.5(m), 138.9(m), 138.0(m), 136.4(m), 121.6(m), 36.9, 33.7, 27.9. GC-MS: 264.

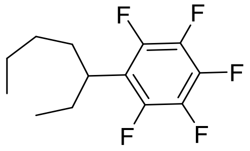

c8

(1,2,3,4,5-Pentafluoro-6-(heptan-3-yl)benzene, synthesized via GP1, colorless liquid, 20.5 mg (Yield = 77 %). ^{1}H NMR (400 MHz, $CDCl_3$) δ 2.97 (tt, *J* = 9.2, 6.4 Hz, 1H), 1.83 – 1.63 (m, 4H), 1.37 – 1.01 (m, 4H), 0.83 (m, 6H). ^{19}F NMR (376 MHz, $CDCl_3$) δ -142.4 (dd, *J* = 22.9, 8.0 Hz), -158.1 (td, *J* = 20.6, 4.0 Hz), -162.9 (m). ^{13}C NMR (101 MHz, $CDCl_3$) δ 146.8(m), 144.2(m), 140.6(m), 138.8(m), 138.1(m), 136.5(m), 118.2(m), 38.5, 33.4, 30.4, 27.1, 22.7, 14.1, 12.6. GC-

MS: 266. HRMS (EI/LTQ-Orbitrap) m/z: $[M]^+$ Calcd for $C_{13}H_{15}F_5^+$ 266.1088; Found 266.1087.

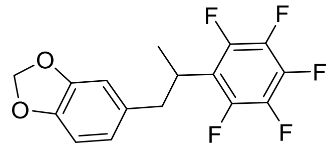

c9

5-(2-(perfluorophenyl)propyl)benzo[d][1,3]dioxole, synthesized via GP1, colorless liquid, 17.6 mg (Yield = 53 %).^{1}H NMR (400 MHz, $CDCl_3$) δ 6.66 (d, *J* = 7.8 Hz, 1H), 6.60 (d, *J* = 1.8 Hz, 1H), 6.53 (dd, *J* = 7.9, 1.7 Hz, 1H), 5.91 (s, 2H), 3.45 (h, *J* = 7.4 Hz, 1H), 2.90 (d, *J* = 8.1 Hz, 2H), 1.37 (d, *J* = 7.1 Hz, 3H). ^{19}F NMR (376 MHz, $CDCl_3$) δ -142.8 (m), -157.6 (t, *J* = 21.1 Hz), -162.7 (td, *J* = 22.3, 7.7 Hz). ^{13}C NMR (101 MHz, $CDCl_3$) δ 147.8, 146.5(m), 146.2, 144.0(m), 140.8(m), 138.8(m), 138.3(m), 136.3(m), 133.4, 121.7, 118.8(m), 109.0, 108.3, 101.0, 41.2(m), 32.8, 19.1(m). GC-MS: 330. HRMS (APPI/LTQ-Orbitrap) m/z: $[M]^+$ Calcd for $C_{16}H_{11}F_5O_2^+$ 330.0674; Found 330.0678.

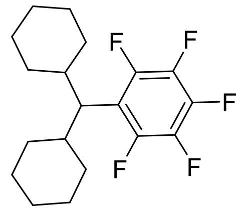

c10

((Perfluorophenyl)methylene)dicyclohexane, synthesized via GP1, colorless liquid, 22.1 mg (Yield = 64 %).^{1}H NMR (400 MHz, $CDCl_3$) δ 2.80 (t, *J* = 7.6 Hz, 1H), 1.99 (qd, *J* = 7.8, 4.1 Hz, 2H), 1.87 – 1.49 (m, 10H), 1.36 – 0.97 (m, 6H), 0.94 – 0.65 (m, 4H). ^{19}F NMR (376 MHz, $CDCl_3$) δ -135.6 (dd, *J* = 22.9, 8.0 Hz), -139.3 (dd, *J* = 24.0, 8.0 Hz), -157.7 (t, *J* = 20.6 Hz), -162.9 (td, *J* = 21.7, 8.0 Hz), -163.2 (td, *J* = 22.9, 8.0 Hz). ^{13}C NMR (101 MHz, $CDCl_3$) δ 146.9(m), 144.4(m), 140.5(m), 138.0(m), 136.3(m), 115.5(m), 48.7, 37.2, 32.4, 30.5, 26.6, 14.3. GC-MS: 346. HRMS (EI/LTQ-Orbitrap) m/z: $[M]^+$ Calcd for $C_{19}H_{23}F_5^+$ 346.1714; Found 346.1717.

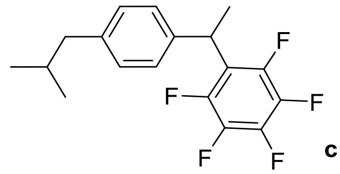

c11

1,2,3,4,5-pentafluoro-6-(1-(4-isobutylphenyl)ethyl)benzene, synthesized via GP1, colorless liquid, 28.9 mg (Yield = 88 %). ^{1}H NMR (400 MHz, $CDCl_3$) δ 7.19 (d, *J* = 8.2 Hz, 2H), 7.08 (d, *J* = 8.2 Hz, 2H), 4.55 (q, *J* = 7.3 Hz, 1H), 2.44 (d, *J* = 7.2 Hz, 2H), 1.83 (m, 1H), 1.74 (dt, *J* = 7.5, 1.2 Hz, 3H), 0.89 (d, *J* = 6.6 Hz, 6H). ^{19}F NMR (376 MHz, $CDCl_3$) δ -142.5 (m), -157.7 (t, *J* = 21.2 Hz), -162.5 (td, *J* = 22.3, 8.0 Hz). ^{13}C NMR (101 MHz, $CDCl_3$) δ 146.4(m), 144.0(m), 141.1(m), 140.5, 139.5, 139.0(m), 138.5(m), 136.5(m), 129.4, 126.9, 119.7(m), 45.1, 34.3, 30.3, 22.5, 18.5(m). GC-MS: 328. HRMS (APPI/LTQ-Orbitrap) m/z: $[M]^+$ Calcd for $C_{18}H_{17}F_5^+$ 328.1245; Found 328.1253.

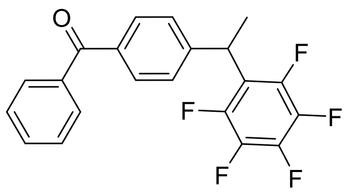

c12

(4-(1-(perfluorophenyl)ethyl)phenyl)(phenyl)methanone, synthesized via GP1, colorless liquid, 27.1mg (Yield = 72 %). ^{1}H NMR (400 MHz, $CDCl_3$) δ 7.83 – 7.74 (m, 3H), 7.68 – 7.55 (m, 2H), 7.54 – 7.46 (m, 3H), 7.42 (t, *J* = 7.7 Hz, 1H), 4.66 (q, *J* = 7.4 Hz, 1H), 1.79 (dt, *J* = 7.4, 1.2 Hz, 3H). ^{19}F NMR (376 MHz, $CDCl_3$) δ -142.3 (m), -156.6 (t, *J* = 20.9 Hz), -161.9 (td, *J* = 21.9, 7.9 Hz). ^{13}C NMR (101 MHz, $CDCl_3$) δ 196.6, 146.3(m), 143.9(m), 142.5, 141.3(m), 139.0(m), 138.1, 137.6, 136.6(m), 132.7, 131.3, 130.2, 128.9, 128.6, 128.4, 118.9(m), 34.4, 18.4. GC-MS: 376. HRMS (APPI/LTQ-Orbitrap) m/z: $[M + H]^+$ Calcd for $C_{21}H_{14}F_5O^+$ 377.0959; Found 377.0972.

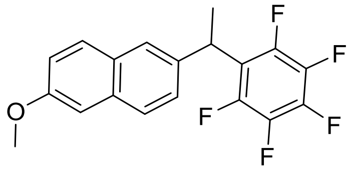

c13

2-methoxy-6-(1-(perfluorophenyl)ethyl)naphthalene, synthesized via GP1, white solid, m.p. 69-71 °C , 29.5 mg (Yield = 84 %). ^{1}H NMR (400 MHz, $CDCl_3$) δ 7.75 – 7.65 (m, 3H), 7.39 – 7.32 (m, 1H), 7.21 – 7.08 (m, 2H), 4.72 (q, *J* = 7.4 Hz, 1H), 3.92 (s, 3H), 1.85 (dt, *J* = 7.3, 1.1 Hz, 3H). ^{19}F NMR (376 MHz, $CDCl_3$) δ -142.5 (m), -157.4 (d, *J* = 21.0 Hz), -162.3 (m). ^{13}C NMR (101 MHz, $CDCl_3$) δ 157.9, 146.4(m), 143.9(m), 141.1(m), 139.0(m), 138.6(m), 137.3, 136.5(m), 133.6, 129.4, 129.0, 127.3, 126.3, 125.3, 119.6(m), 119.2, 105.7, 55.4, 34.6, 18.4. GC-MS: 352. HRMS (APPI/LTQ-Orbitrap) m/z: $[M]^+$ Calcd for $C_{19}H_{13}F_5O^+$ 352.0881; Found 352.0893.

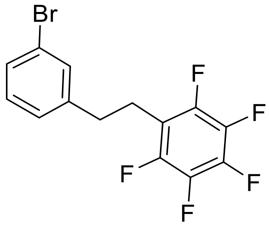

d1

1-(3-bromophenethyl)-2,3,4,5,6-pentafluorobenzene, synthesized via GP1, white solid, 24.5 mg (Yield = 70 %). ^{1}H NMR (400 MHz, $CDCl_3$) δ 7.40 – 7.31 (m, 2H), 7.16 (t, *J* = 7.8 Hz, 1H), 7.09 (dt, *J* = 7.6, 1.5 Hz, 1H), 2.98 (m, 2H), 2.84 (m, 2H). ^{19}F NMR (376 MHz, $CDCl_3$) δ -144.2 (m), -157.1 (t, *J* = 21.2 Hz), -162.5 (m). ^{13}C NMR (101 MHz, $CDCl_3$) δ 146.3(m), 144.0(m), 142.4, 141.2(m), 138.7(m), 136.3(m), 131.6, 130.3, 129.9, 127.1, 122.7, 114.1(m), 35.1, 24.3. HRMS (EI/LTQ-Orbitrap) m/z: $[M]^+$ Calcd for $C_{14}H_8BrF_5^+$ 349.9724; Found 349.9729.

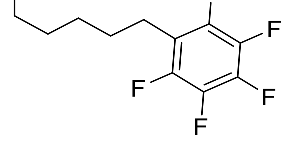

d2

1-(5-bromopentyl)-2,3,4,5,6-pentafluorobenzene, synthesized via GP1, colorless liquid, 24.7 mg (Yield = 78 %). ^{1}H NMR (400 MHz, $CDCl_3$) δ 3.40 (t, *J* = 6.7 Hz, 2H), 2.71 (tt, *J* = 7.6, 1.8 Hz, 2H), 1.89 (p, *J* = 6.9 Hz, 2H), 1.68 – 1.56 (m, 2H), 1.55 – 1.44 (m, 2H). ^{19}F NMR (376 MHz, $CDCl_3$)

δ -144.3 (m), -157.95 (t, *J* = 21.1 Hz), -162.91 (td, *J* = 22.2, 8.5 Hz). ^{13}C NMR (101 MHz, $CDCl_3$) δ 146.4(m), 144.0(m), 140.9(m), 138.8(m), 138.7(m), 136.3(m), 115.1(m), 33.5, 32.4, 28.5, 27.8, 22.3. GC-MS: 316.

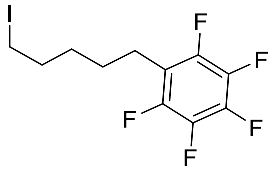

d3

1-(5-iodopentyl)-2,3,4,5,6-pentafluorobenzene, synthesized via GP1, colorless liquid, 16.2 mg (Yield = 45 %). ^{1}H NMR (400 MHz, $CDCl_3$) δ 3.21 (t, *J* = 7.0, 2H), 2.74 (m, 2H), 1.94 – 1.82 (m, 2H), 1.64 (p, *J* = 7.6 Hz, 2H), 1.48 (p, *J* = 7.7 Hz, 2H). ^{19}F NMR (376 MHz, $CDCl_3$) δ -144.4 (m), -157.9 (t, *J* = 20.8 Hz), -162.87 (td, *J* = 22.0, 8.5 Hz). ^{13}C NMR (101 MHz, $CDCl_3$) δ 146.4(m), 143.9(m), 140.9(m), 139.0(m), 138.4(m), 136.3(m), 115.2(m), 33.1, 30.1, 28.3, 22.2, 6.4. GC-MS: 364. HRMS (EI/LTQ-Orbitrap) m/z: $[M]^+$ Calcd for $C_{11}H_{10}F_5I^+$ 363.9742; Found 363.9748.

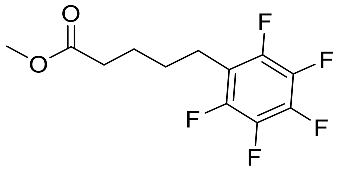

d4

methyl 5-(perfluorophenyl)pentanoate, synthesized via GP1, colorless liquid, 22.5 mg (Yield = 80 %). ^{1}H NMR (400 MHz, $CDCl_3$) δ 3.66 (s, 3H), 2.76 – 2.67 (m, 2H), 2.34 (t, *J* = 7.0 Hz, 2H), 1.64 (m, 4H). ^{19}F NMR (376 MHz, $CDCl_3$) δ -144.3 (dd, *J* = 22.1, 8.4 Hz), -157.9 (t, *J* = 20.6 Hz), -162.92 (td, *J* = 22.1, 8.5 Hz). ^{13}C NMR (101 MHz, $CDCl_3$) δ 173.8, 146.4(m), 144.0(m), 140.9(m), 138.8(m), 138.4(m), 136.3(m), 115.0(m), 51.7, 33.7, 28.8, 24.4, 22.1. HRMS (EI/LTQ-Orbitrap) m/z: $[M]^+$ Calcd for $C_{12}H_{11}F_5O_2^+$ 282.0674; Found 282.0679.

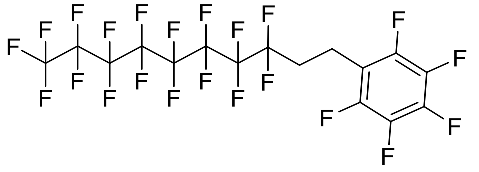

d5

1,2,3,4,5-pentafluoro-6-(3,3,4,4,5,5,6,6,7,7,8,8,9,9,10,10,10-heptadecafluorodecyl)benzene, synthesized via GP1, colorless liquid, 29.5 mg (Yield = 48 %). ^{1}H NMR (400 MHz, $CDCl_3$) δ 3.04 (m, 2H), 2.38 (m, 2H). ^{19}F NMR (376 MHz, $CDCl_3$) δ -80.8 (m), -115.3 (m), -121.5 – -122.1 (m), -122.7 (m), -123.5 (m), -126.1 (m), -143.9 (dt, *J* = 20.0, 9.6 Hz), -155.7 (t, *J* = 20.6 Hz), -161.8 (dt, *J* = 20.7, 10.7 Hz). ^{13}C NMR (101 MHz, $CDCl_3$) δ 29.3 (t, *J* = 22.3 Hz), 13.0. HRMS (EI/LTQ-Orbitrap) m/z: $[M]^+$ Calcd for $C_{16}H_4F_{22}^+$ 613.9956; Found 613.9965.

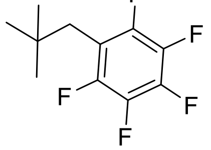

d6

1,2,3,4,5-pentafluoro-6-neopentylbenzene, synthesized via GP1, colorless liquid, 8.5 mg (Yield = 36 %). ^{1}H NMR (400 MHz, $CDCl_3$) δ 2.60 (s, 2H), 0.95 (t, *J* = 1.3 Hz, 9H). ^{19}F NMR (376 MHz,

$CDCl_3$) δ -140.1 (m), -157.5 (t, *J* = 21.1 Hz), -163.2(m). ^{13}C NMR (101 MHz, $CDCl_3$) δ 146.9(m), 144.3(m), 140.8(m), 138.8(m), 138.3(m), 136.3(m), 113.3(m), 35.9, 33.1, 29.3. GC-MS: 238. HRMS (EI/LTQ-Orbitrap) m/z: $[M]^+$ Calcd for $C_{11}H_{11}F_5^+$ 238.0775; Found 238.0770.

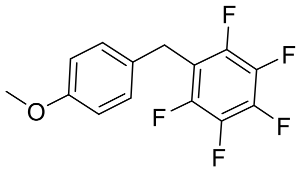

d7

1,2,3,4,5-pentafluoro-6-(4-methoxybenzyl)benzene, synthesized via GP1, colorless liquid, 19.5 mg (Yield = 68 %). ^1H NMR (400 MHz, $CDCl_3$) δ 7.21 – 7.13 (m, 2H), 6.87 – 6.79 (m, 2H), 3.96 (t, *J* = 2.1 Hz, 2H), 3.78 (s, 3H). ^{19}F NMR (376 MHz, $CDCl_3$) δ -143.6 (dd, *J* = 22.9, 8.0 Hz), -157.4 (t, *J* = 20.6 Hz), -162.4 (m). ^{13}C NMR (101 MHz, $CDCl_3$) δ 158.7, 146.2(m), 143.7(m), 141.2(m), 138.8(m), 136.4(m), 129.7, 129.6, 115.0(m), 114.3, 55.4, 27.5. GC-MS: 288. HRMS (APPI/LTQ-Orbitrap) m/z: $[M]^+$ Calcd for $C_{14}H_9F_5O^+$ 288.0568; Found 288.0578.

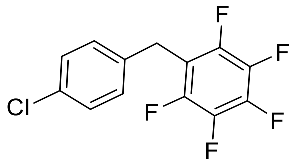

d8

1-(4-chlorobenzyl)-2,3,4,5,6-pentafluorobenzene, synthesized via GP1, colorless liquid, 21.3 mg (Yield = 73 %). ^1H NMR (400 MHz, $CDCl_3$) δ 7.29 – 7.21 (m, 2H), 7.15 (d, *J* = 8.2 Hz, 2H), 3.98 (s, 2H). ^{19}F NMR (376 MHz, $CDCl_3$) δ -143.24 – -143.38 (m), -156.47 (t, *J* = 21.1 Hz), -161.99 (td, *J* = 22.1, 8.5 Hz). ^{13}C NMR (101 MHz, $CDCl_3$) δ 146.3(m), 143.9(m), 141.5(m), 139.0(m), 136.5(m), 136.0, 133.1, 129.9, 129.1, 114.0(m), 27.7. GC-MS: 292. HRMS (APPI/LTQ-Orbitrap) m/z: $[M-H]^-$ Calcd for $C_{13}H_5F_5O^-$ 291.0005; Found 291.0002.

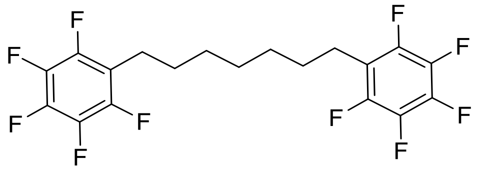

d9

1,7-bis(perfluorophenyl)heptane, synthesized via modified GP1 (with 200 mol% **b1**), colorless liquid, 27.2 mg (Yield = 63 %). ^1H NMR (400 MHz, $CDCl_3$) δ 2.68 (m, 4H), 1.63 – 1.50 (m, 4H), 1.41 – 1.27 (m, 6H). ^{19}F NMR (376 MHz, $CDCl_3$) δ -144.5 (m), -158.4 (m), -163.1 (m). ^{13}C NMR (101 MHz, $CDCl_3$) δ 146.3(m), 144.0(m), 140.8(m), 138.8(m), 138.3(m), 136.3(m), 115.6(m), 29.3, 29.0, 28.9, 22.4. GC-MS: 432. HRMS (EI/LTQ-Orbitrap) m/z: $[M]^+$ Calcd for $C_{19}H_{14}F_{10}^+$ 432.0930; Found 432.0929.

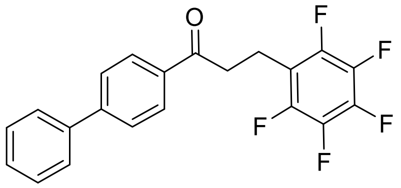

d10

1-([1,1'-biphenyl]-4-yl)-3-(perfluorophenyl)propan-1-one, synthesized via GP1, white solid, m.p. > 200 °C , 26.4mg (Yield = 70 %).^1H NMR (400 MHz, $CDCl_3$) δ 8.02 (d, *J* = 8.5 Hz, 2H), 7.69 (d, *J* = 8.4 Hz, 2H), 7.63 (d, *J* = 6.9 Hz, 2H), 7.48 (t, *J* = 7.3 Hz, 2H), 7.41 (t, *J* = 7.3 Hz, 1H), 3.33

(t, J = 7.4 Hz, 2H), 3.21 – 3.12 (m, 2H). ^{19}F NMR (376 MHz, $CDCl_3$) δ -143.4 (m), -157.3 (t, J = 20.7 Hz), -162.6 (td, J = 21.8, 8.1 Hz). ^{13}C NMR (101 MHz, $CDCl_3$) δ 197.4, 146.3, 139.9, 135.1, 129.1, 128.7, 128.5, 127.5, 127.4, 37.6, 17.3. GC-MS: 376. HRMS (ESI/QTOF) m/z: [M + H]$^+$ Calcd for $C_{21}H_{14}F_5O^+$ 377.0959; Found 377.0962.

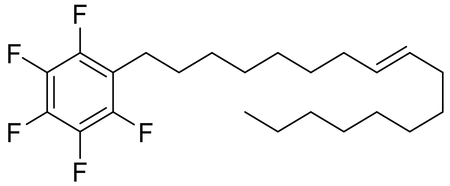

d11

(E)-1,2,3,4,5-pentafluoro-6-(heptadec-8-en-1-yl)benzene, synthesized via GP1, colorless liquid, 37.3 mg (Yield = 92 %). ^{1}H NMR (400 MHz, $CDCl_3$) δ 5.42 – 5.35 (m, 2H), 2.73 – 2.63 (m, 2H), 1.96 (q, J = 6.6 Hz, 4H), 1.56 (dd, J = 14.8, 7.3 Hz, 2H), 1.34 – 1.19 (m, 20H), 0.92 – 0.84 (m, 3H). ^{19}F NMR (376 MHz, $CDCl_3$) δ -144.5 (m), -158.5 (t, J = 20.8 Hz), -163.3 (m). ^{13}C NMR (101 MHz, $CDCl_3$) δ 146.4(m), 144.0(m), 140.8(m), 138.8(m), 136.3(m), 130.7, 130.3, 115.7(m), 32.8, 32.7, 32.1, 29.8, 29.7, 29.7, 29.5, 29.4, 29.3, 29.2, 29.2, 29.1, 22.8, 22.5, 14.2. GC-MS: 404.

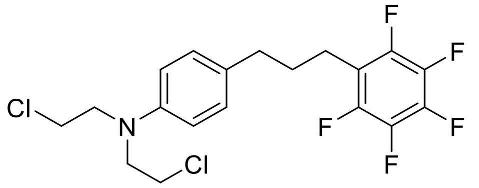

d12

N,N-bis(2-chloroethyl)-4-(3-(perfluorophenyl)propyl)aniline, synthesized via GP1, yellowish liquid, 27.2 mg (Yield = 64 %). ^{1}H NMR (400 MHz, $CDCl_3$) δ 7.12 – 7.04 (m, 2H), 6.68 – 6.58 (m, 2H), 3.71 (m,4H), 3.65 – 3.59 (m, 4H), 2.73 (t, J = 7.6 Hz, 2H), 2.59 (t, J = 7.8 Hz, 2H), 1.94 – 1.81 (m, 2H). ^{19}F NMR (376 MHz, $CDCl_3$) δ -144.2 (m), -158.1 (t, J = 20.8 Hz), -163.0 (m, 2F). ^{13}C NMR (101 MHz, $CDCl_3$) δ 146.4(m), 144.5, 144.0(m), 141.0(m), 138.8(m), 138.3(m), 136.3(m), 130.5, 129.6, 115.3(m), 112.3, 53.7, 40.6, 34.3, 31.0, 22.2.

HRMS (ESI/QTOF) m/z: [M + H]$^+$ Calcd for $C_{19}H_{19}Cl_2F_5N^+$ 426.0809; Found 426.0813.

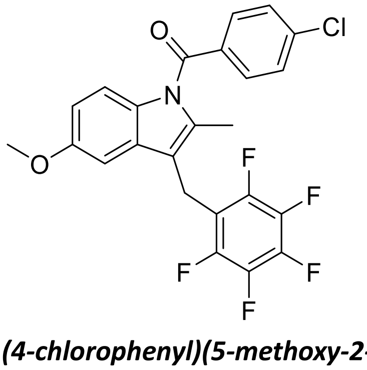

d13

(4-chlorophenyl)(5-methoxy-2-methyl-3-((perfluorophenyl)methyl)-1H-indol-1-yl)methanone, synthesized via GP1, white solid, m.p. 123-125 °C , 30.1 mg (Yield = 63 %). ^{1}H NMR (400 MHz, $CDCl_3$) δ 7.69 – 7.60 (m, 2H), 7.51 – 7.43 (m, 2H), 6.96 (d, J = 2.5 Hz, 1H), 6.84 (d, J = 9.0 Hz, 1H), 6.65 (dd, J = 9.0, 2.5 Hz, 1H), 4.05 (d, J = 1.7 Hz, 2H), 3.82 (s, 3H), 2.46 (s, 3H). ^{19}F NMR (376 MHz, $CDCl_3$) δ -142.1 (m), -156.7 (t, J = 20.9 Hz), -162.2 (m). ^{13}C NMR (101 MHz, $CDCl_3$) δ 168.5, 156.1, 146.6(m), 144.1(m), 141.4(m), 139.5, 139.0(m), 136.5(m), 135.7, 134.0, 131.3, 130.9, 130.1, 129.3, 115.1, 114.8, 113.3(m), 111.7, 100.9, 100.9, 100.8, 55.7, 17.6, 13.3. GC-MS: 479. HRMS (ESI/QTOF) m/z: [M + H]$^+$ Calcd for $C_{24}H_{16}ClF_5NO_2^+$ 480.0784;

Found 480.0794.

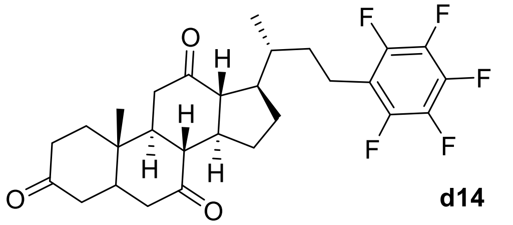

d14

Synthesized via GP1, white solid, m.p. >200 °C , 21.4 mg (Yield = 42 %). ^{1}H NMR (400 MHz, $CDCl_3$) δ 2.96 – 2.71 (m, 4H), 2.60 (m, 1H), 2.40 – 1.92 (m, 11H), 1.86 (m, 1H), 1.64 (m, 1H), 1.46 – 1.21 (m, 7H), 1.07 (s, 3H), 0.97 (d, *J* = 6.5 Hz, 3H). ^{19}F NMR (376 MHz, $CDCl_3$) δ -144.9 (dd, *J* = 22.5, 8.2 Hz), -158.4 (t, *J* = 20.9 Hz), -163.0 (td, *J* = 21.8, 8.2 Hz). ^{13}C NMR (101 MHz, $CDCl_3$) δ 211.9, 209.0, 208.7, 146.2(m), 143.8(m), 140.6(m), 138.7(m), 138.1(m), 136.2(m), 116.0(m), 115.8, 115.6, 56.9, 51.8, 49.0, 46.8, 45.6, 45.3, 45.0, 42.8, 38.6, 36.5, 36.1, 36.0, 35.3, 35.1, 27.7, 25.1, 21.9, 19.5, 18.7, 11.8. HRMS (ESI/QTOF) m/z: [M + Na]$^+$ Calcd for $C_{29}H_{33}F_5NaO_3^+$ 547.2242; Found 547.2259.

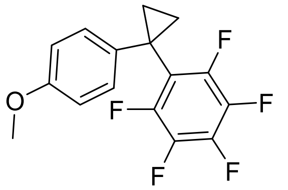

e3

1,2,3,4,5-pentafluoro-6-(1-(4-methoxyphenyl)cyclopropyl)benzene, synthesized via GP1, colorless liquid, 12.5 mg (Yield = 40 %). ^{1}H NMR (400 MHz, $CDCl_3$) δ 7.34 – 7.28 (m, 2H), 6.84 – 6.78 (m, 2H), 3.77 (s, 3H), 1.40 – 1.35 (m, 2H), 1.25 – 1.19 (m, 2H). ^{19}F NMR (376 MHz, $CDCl_3$) δ -140.7 (m), -156.9 (t, *J* = 20.8 Hz), -162.6 (m). ^{13}C NMR (101 MHz, $CDCl_3$) δ 158.7, 147.6(m), 145.1(m), 141.6(m), 139.1(m), 138.9(m), 136.4(m), 135.2, 129.4, 119.2(m), 114.1, 55.4, 19.1, 14.4(m). GC-MS: 314. HRMS (APPI/LTQ-Orbitrap) m/z: [M]$^+$ Calcd for $C_{16}H_{11}F_5O^+$ 314.0725; Found 314.0737.

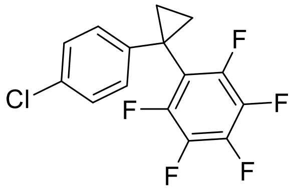

e4

1,2,3,4,5-pentafluoro-6-(1-(4-chlorophenyl)cyclopropyl)benzene, synthesized via GP1, colorless liquid, 19.1 mg (Yield = 60 %). ^{1}H NMR (400 MHz, $CDCl_3$) δ 7.25 (m, 4H), 1.41 (m, 2H), 1.32 – 1.22 (m, 2H). ^{19}F NMR (376 MHz, $CDCl_3$) δ -140.3 (dd, *J* = 22.0, 8.1 Hz), -155.9 (t, *J* = 21.0 Hz), -162.2 (td, *J* = 21.8, 8.0 Hz). ^{13}C NMR (101 MHz, $CDCl_3$) δ 147.7(m), 145.2(m), 141.9(m), 141.4, 139.0(m), 136.5(m), 132.9, 129.3, 128.9, 118.3(m), 19.2, 15.0(m). GC-MS: 318. HRMS (APPI/LTQ-Orbitrap) m/z: [M]$^+$ Calcd for $C_{15}H_8ClF_5^+$ 318.0229; Found 318.0240.

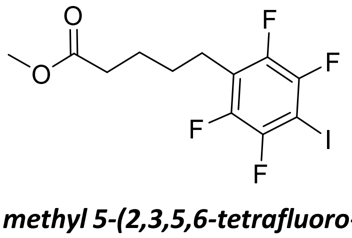

f1

methyl 5-(2,3,5,6-tetrafluoro-4-iodophenyl)pentanoate, synthesized via GP2, colorless liquid,

21.0 mg (Yield = 54 %). ^{1}H NMR (400 MHz, $CDCl_3$) δ 3.67 (s, 3H), 2.75 (t, *J* = 7.0Hz, 2H), 2.35 (t, *J* = 7.0 Hz, 2H), 1.67 (m, 4H). ^{19}F NMR (376 MHz, $CDCl_3$) δ -121.5 (m), -142.3 (m). ^{13}C NMR (101 MHz, $CDCl_3$) δ 173.8, 148.3(m), 145.7(m), 143.4(m), 121.3(m), 69.3(m), 51.7, 33.7, 28.5, 24.5, 23.0. GC-MS: 390. HRMS (EI/LTQ-Orbitrap) m/z: $[M]^+$ Calcd for $C_{12}H_{11}IF_4O_2^+$ 389.9734; Found 389.9724.

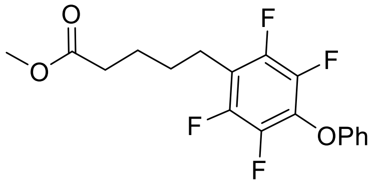

f2

methyl 5-(2,3,5,6-tetrafluoro-4-phenoxyphenyl)pentanoate, synthesized via GP2, colorless liquid, 24.1 mg (Yield = 68 %). ^{1}H NMR (400 MHz, $CDCl_3$) δ 7.37 – 7.29 (m, 2H), 7.10 (td, *J* = 7.4, 1.2 Hz, 1H), 6.97 (d, *J* = 8.2 Hz, 2H), 3.68 (s, 3H), 2.76 (t, *J* = 7.0 Hz, 2H), 2.37 (t, *J* = 6.9 Hz, 2H), 1.79 – 1.63 (m, 4H). ^{19}F NMR (376 MHz, $CDCl_3$) δ -144.68 – -144.81 (m), -155.13 – -155.30 (m). ^{13}C NMR (101 MHz, $CDCl_3$) δ 173.9, 157.4, 146.7(m), 144.3(m), 142.9(m), 140.4(m), 131.6(m), 129.9, 123.7, 116.3(m), 115.6, 51.7, 33.7, 28.8, 24.5, 22.4. GC-MS: 356. HRMS (APPI/LTQ-Orbitrap) m/z: $[M + H]^+$ Calcd for $C_{13}H_{12}F_7O_2^+$ 357.1108; Found 357.1105.

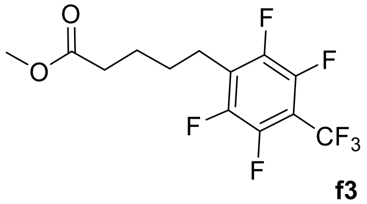

f3

methyl 5-(2,3,5,6-tetrafluoro-4-(trifluoromethyl)phenyl)pentanoate, synthesized via GP2, colorless liquid, 15.2 mg (Yield = 46 %).^{1}H NMR (400 MHz, $CDCl_3$) δ 3.67 (s, 3H), 2.80 (t, *J* = 6.9 Hz, 2H), 2.36 (t, *J* = 6.6 Hz, 2H), 1.68 (m, 4H). ^{19}F NMR (376 MHz, $CDCl_3$) δ -56.24 (t, *J* = 21.6 Hz), -141.46 (m), -142.37 (m). ^{13}C NMR (101 MHz, $CDCl_3$) δ 173.7, 146.4(m), 145.3(m), 144.1(m), 142.7(m), 125.2(m), 122.4, 119.7, 117.0, 107.7(m), 51.8, 33.6, 28.4, 24.5, 22.9. GC-MS: 332. HRMS (APPI/LTQ-Orbitrap) m/z: $[M + H]^+$ Calcd for $C_{13}H_{12}F_7O_2^+$ 333.0720; Found 333.0719.

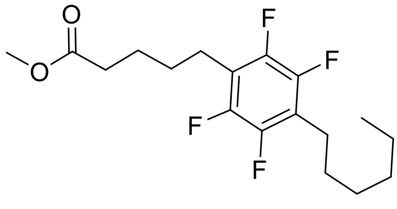

f4

methyl 5-(2,3,5,6-tetrafluoro-4-hexylphenyl)pentanoate, synthesized via GP2, colorless liquid, 17.0mg (Yield = 49 %). ^{1}H NMR (400 MHz, $CDCl_3$) δ 3.66 (s, 3H), 2.69 (m, 4H), 2.34 (t, *J* = 6.9 Hz, 2H), 1.74 – 1.51 (6H), 1.39 – 1.23 (6H), 0.92 – 0.84 (m, 3H). ^{19}F NMR (376 MHz, $CDCl_3$) δ -145.70 – -146.48 (m). ^{13}C NMR (151 MHz, $CDCl_3$) δ 174.0, 145.5(m), 143.9(m), 118.5(m), 117.3(m), 51.7, 33.8, 31.6, 29.4, 29.0, 28.8, 24.5, 22.8, 22.7, 22.4, 14.2. GC-MS: 348. HRMS (APPI/LTQ-Orbitrap) m/z: $[M + H]^+$ Calcd for $C_{13}H_{12}F_7O_2^+$ 349.1785; Found 349.1784.

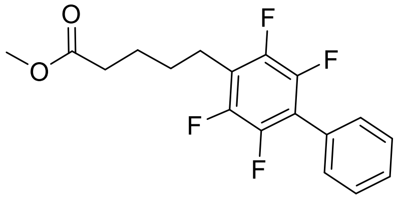

f5

methyl 5-(2,3,5,6-tetrafluoro-[1,1'-biphenyl]-4-yl)pentanoate, synthesized via GP2, white solid, m.p. 82-84 °C , 26.0 mg (Yield = 76 %). ^{1}H NMR (400 MHz, $CDCl_3$) δ 7.46 (m, 5H), 3.68 (s, 3H), 2.80 (t, *J* = 6.8 Hz, 2H), 2.38 (t, *J* = 6.9 Hz, 2H), 1.72 (m, 4H). ^{19}F NMR (376 MHz, $CDCl_3$) δ -144.78 – -145.73 (m). ^{13}C NMR (101 MHz, $CDCl_3$) δ 173.9, 146.6(m), 144.9(m), 144.0(m), 142.6(m), 130.3, 129.1, 128.7, 127.8, 119.3(m), 118.5(m), 51.7, 33.8, 28.8, 24.5, 22.6. GC-MS: 340. HRMS (APPI/LTQ-Orbitrap) m/z: $[M + H]^+$ Calcd for $C_{18}H_{17}F_4O_2^+$ 341.1159; Found 341.1161.

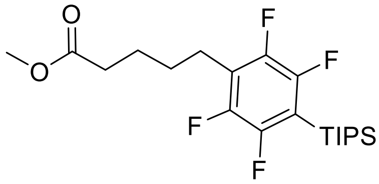

f6

methyl 5-(2,3,5,6-tetrafluoro-4-(triisopropylsilyl)phenyl)pentanoate, synthesized via GP2, colorless liquid, 23.2 mg (Yield = 55 %).^{1}H NMR (400 MHz, $CDCl_3$) δ 3.67 (s, 3H), 2.75 (t, *J* = 7.1 Hz, 2H), 2.36 (t, *J* = 6.9 Hz, 2H), 1.78 – 1.59 (m, 4H), 1.53 (m, 3H), 1.08 (d, *J* = 7.5 Hz, 18H). ^{19}F NMR (376 MHz, $CDCl_3$) δ -126.19 – -126.36 (m), -144.57 – -144.78 (m). ^{13}C NMR (101 MHz, $CDCl_3$) δ 173.9, 150.4(m), 148.0(m), 146.0(m), 143.6(m), 121.8(m), 110.4(m), 51.7, 33.8, 28.7, 24.7, 22.9, 18.7, 12.3. GC-MS: 420. HRMS (APPI/LTQ-Orbitrap) m/z: $[M + H]^+$ Calcd for $C_{21}H_{33}F_4O_2Si^+$ 421.2180; Found 421.2181.

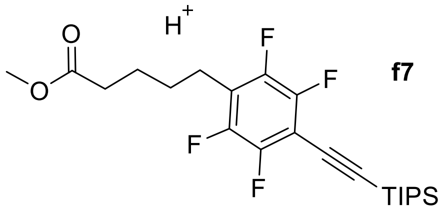

f7

methyl 5-(2,3,5,6-tetrafluoro-4-((triisopropylsilyl)ethynyl)phenyl)pentanoate, synthesized via GP2, colorless liquid, 23.9 mg (Yield = 54 %). ^{1}H NMR (400 MHz, $CDCl_3$) δ 3.66 (d, *J* = 1.2 Hz, 3H), 2.74 (t, *J* = 7.1 Hz, 2H), 2.34 (t, *J* = 6.8 Hz, 2H), 1.74 – 1.56 (m, 4H), 1.13 (m, 21H). ^{19}F NMR (376 MHz, $CDCl_3$) δ -137.44 – -137.67 (m), -145.06 – -145.21 (m). ^{13}C NMR (101 MHz, $CDCl_3$) δ 173.8, 148.5(m), 146.0(m), 143.5(m), 121.0(m), 105.3, 102.6(m), 90.9, 51.7, 33.7, 28.7, 24.5, 22.8, 22.7, 22.7, 18.6, 11.2. GC-MS: 444. HRMS (APPI/LTQ-Orbitrap) m/z: $[M + H]^+$ Calcd for $C_{13}H_{12}F_7O_2^+$ 445.2180; Found 445.2185.

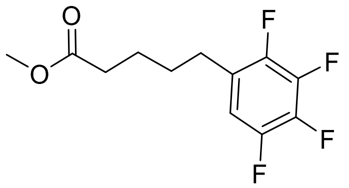

f8

methyl 5-(2,3,4,5-tetrafluorophenyl)pentanoate, synthesized via GP2, colorless liquid, 13.4

mg (Yield = 51 %). ^{1}H NMR (400 MHz, $CDCl_3$) δ 6.79 (m, 1H), 3.67 (s, 3H), 2.67 – 2.57 (m, 2H), 2.34 (t, *J* = 7.0 Hz, 2H), 1.73 – 1.55 (m, 4H). ^{19}F NMR (376 MHz, $CDCl_3$) δ -140.3 (dd, *J* = 21.1, 12.6 Hz), -144.2 (dd, *J* = 20.8, 12.4 Hz), -156.3 (t, *J* = 20.1 Hz), -159.3 (t, *J* = 20.2 Hz). ^{13}C NMR (101 MHz, $CDCl_3$) δ 173.9, 148.3(m), 147.1(m), 145.8(m), 144.7(m), 142.1(m), 140.2(m), 139.6(m), 137.5(m), 125.2(m), 111.3(m), 51.7, 33.8, 29.3, 28.3, 24.4. GC-MS: 264. HRMS (EI/LTQ-Orbitrap) m/z: $[M]^+$ Calcd for $C_{12}H_{12}F_4O_2^+$ 264.0768; Found 264.0770.

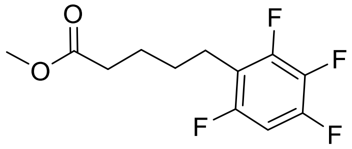

f9

methyl 5-(2,3,4,6-tetrafluorophenyl)pentanoate, synthesized via GP2, colorless liquid, 18.5mg (Yield = 70 %). ^{1}H NMR (400 MHz, $CDCl_3$) δ 6.79 – 6.67 (m, 1H), 3.66 (s, 3H), 2.66 (t, *J* = 7.1, 2H), 2.34 (t, *J* = 7.2 Hz, 2H), 1.73 – 1.54 (m, 4H). ^{19}F NMR (376 MHz, $CDCl_3$) δ -119.6 (d, *J* = 10.9 Hz), -136.0 (dd, *J* = 21.0, 4.8 Hz), -136.9 – -137.1 (m), -165.8 (td, *J* = 21.2, 11.4 Hz). ^{13}C NMR (101 MHz, $CDCl_3$) δ 173.9, 156.7(m), 154.3(m), 151.3(m), 151.2(m), 150.3(m), 150.2(m), 148.8(m), 147.8(m), 138.4(m), 136.0(m), 115.0(m), 100.6(m), 51.7, 33.8, 28.9, 24.5, 22.0.
GC-MS: 264. HRMS (EI/LTQ-Orbitrap) m/z: $[M]^+$ Calcd for $C_{12}H_{12}F_4O_2^+$ 264.0768; Found 264.0772.

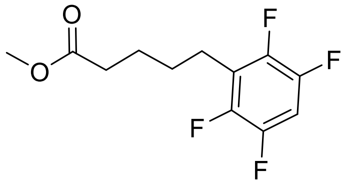

f10

methyl 5-(2,3,5,6-tetrafluorophenyl)pentanoate, synthesized via GP2, colorless liquid, 19.7 mg (Yield = 75 %). ^{1}H NMR (400 MHz, $CDCl_3$) δ 6.91 (m, 1H), 3.67 (s, 3H), 2.75 (t, *J* = 7.1, 2H), 2.35 (t, *J* = 7.1 Hz, 2H), 1.75 – 1.56 (m, 4H). ^{19}F NMR (376 MHz, $CDCl_3$) δ -140.0 (dd, *J* = 22.4, 12.9 Hz), -144.9 (dd, *J* = 22.1, 13.0 Hz). ^{13}C NMR (101 MHz, $CDCl_3$) δ 173.9, 147.0(m), 146.0(m), 144.7(m), 143.6(m), 121.1(m), 103.7(m), 51.7, 33.7, 28.7, 24.5, 22.7, 22.7, 22.7.
GC-MS: 264. HRMS (EI/LTQ-Orbitrap) m/z: $[M]^+$ Calcd for $C_{12}H_{12}F_4O_2^+$ 264.0768; Found 264.0771.

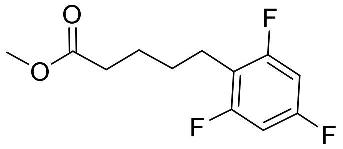

f11

methyl 5-(2,4,6-trifluorophenyl)pentanoate, synthesized via GP2, colorless liquid, 16.1 mg (Yield = 65 %). ^{1}H NMR (400 MHz, $CDCl_3$) δ 6.67 – 6.54 (m, 2H), 3.66 (s, 3H), 2.71 – 2.57 (m, 2H), 2.33 (t, *J* = 7.3 Hz, 2H), 1.74 – 1.51 (m, 4H). ^{19}F NMR (376 MHz, $CDCl_3$) δ -111.8 (m), -113.12 (d, *J* = 4.6 Hz). ^{13}C NMR (101 MHz, $CDCl_3$) δ 174.0, 162.3(m), 159.8(m), 113.7(m), 100.0(m), 51.7, 33.8, 29.0, 24.5, 21.7. GC-MS: 246. HRMS (EI/LTQ-Orbitrap) m/z: $[M]^+$ Calcd for $C_{12}H_{13}F_3O_2^+$ 246.0862; Found 246.0865.

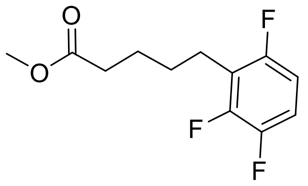

f12

methyl 5-(2,3,6-trifluorophenyl)pentanoate, synthesized via GP2, colorless liquid, 13.1 mg (Yield = 53 %). ^{1}H NMR (400 MHz, $CDCl_3$) δ 6.96 (m, 1H), 6.76 (m, 1H), 3.66 (s, 3H), 2.75 – 2.67 (m, 2H), 2.35 (t, *J* = 6.9 Hz, 2H), 1.65 (m, 4H). ^{19}F NMR (376 MHz, $CDCl_3$) δ -121.2 (dd, *J* = 15.5, 4.1 Hz), -139.1 (dd, *J* = 21.1, 3.9 H), -143.0 (dd, *J* = 21.0, 15.3 Hz). ^{13}C NMR (101 MHz, $CDCl_3$) δ 174.0, 158.0(m), 155.6(m), 150.4(m), 148.4(m), 148.0(m), 146.0(m), 119.7(m), 114.2(m), 110.4(m), 51.7, 33.8, 28.8, 24.6, 22.4. GC-MS: 246. HRMS (EI/LTQ-Orbitrap) m/z: $[M]^+$ Calcd for $C_{12}H_{13}F_3O_2^+$ 246.0862; Found 246.0867.

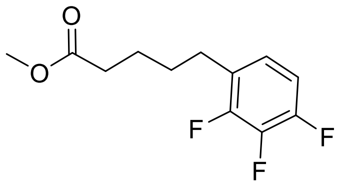

f13

methyl 5-(2,3,4-trifluorophenyl)pentanoate, synthesized via GP2, colorless liquid, 9.7 mg (Yield = 40 %). ^{1}H NMR (400 MHz, $CDCl_3$) δ 6.95 – 6.80 (m, 2H), 3.66 (s, 3H), 2.64 (t, *J* = 7.3 Hz, 2H), 2.34 (t, *J* = 7.1 Hz, 2H), 1.73 – 1.56 (m, 4H). ^{19}F NMR (376 MHz, $CDCl_3$) δ -138.1 (dd, *J* = 20.4, 6.1 Hz), -139.5 (dd, *J* = 20.3, 6.2 Hz), -161.2 (t, *J* = 20.2 Hz). ^{13}C NMR (101 MHz, $CDCl_3$) δ 174.0, 151.0(m), 148.5(m), 141.3(m), 138.8(m), 126.4(m), 123.7(m), 111.8(m), 51.7, 33.9, 29.5, 28.4, 24.5. GC-MS: 246. HRMS (EI/LTQ-Orbitrap) m/z: $[M]^+$ Calcd for $C_{12}H_{13}F_3O_2^+$ 246.0862; Found 246.0865.

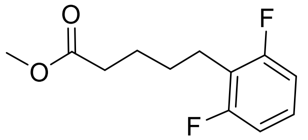

f14

methyl 5-(2,6-difluorophenyl)pentanoate, synthesized via GP2, colorless liquid, 14.7 mg (Yield = 64 %).^{1}H NMR (400 MHz, $CDCl_3$) δ 7.12 (m, 1H), 6.83 (m, 2H), 3.66 (s, 3H), 2.74 – 2.62 (m, 2H), 2.34 (t, *J* = 7.3 Hz, 2H), 1.74 – 1.53 (m, 4H). ^{19}F NMR (376 MHz, $CDCl_3$) δ -116.1 (s). ^{13}C NMR (101 MHz, $CDCl_3$) δ 174.1, 162.9(d, *J* = 9.1 Hz), 160.4(d, *J* = 9.1 Hz), 127.4(t, *J* = 10.3 Hz), 117.6(t, *J* = 20.4 Hz), 111.2(m), 51.6, 33.9, 29.0, 24.6, 22.0. GC-MS: 228. HRMS (EI/LTQ-Orbitrap) m/z: $[M]^+$ Calcd for $C_{12}H_{14}O_2F_2^+$ 228.0956; Found 228.0953.

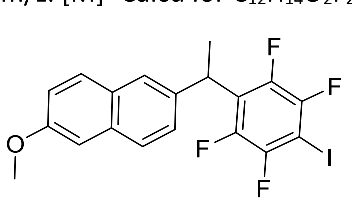

g1

2-methoxy-6-(1-(2,3,5,6-tetrafluoro-4-iodo-phenyl)ethyl)naphthalene, synthesized via GP2, white solid, m.p. 146-148 °C , 22.0 mg (Yield = 48 %). ^{1}H NMR (400 MHz, $CDCl_3$) δ 7.73 – 7.65 (m, 3H), 7.36 (dd, *J* = 8.5, 1.9 Hz, 1H), 7.15 (dd, *J* = 9.0, 2.5 Hz, 1H), 7.10 (d, *J* = 2.5 Hz, 1H), 4.75 (q, *J* = 7.3 Hz, 1H), 3.91 (s, 3H), 1.86 (dt, *J* = 7.4, 1.1 Hz, 3H). ^{19}F NMR (376 MHz, $CDCl_3$) δ -120.9 – -121.1 (m), -140.1 – -140.5 (m). ^{13}C NMR (101 MHz, $CDCl_3$) δ 157.8, 148.7(m), 146.2(m),

145.9(m), 143.4(m), 137.1, 133.6, 129.5, 129.0, 127.3, 126.4, 125.5(m), 125.4, 119.2, 105.7, 69.8(m), 55.5, 35.3, 18.2. GC-MS: 460. HRMS (APPI/LTQ-Orbitrap) m/z: $[M]^+$ Calcd for $C_{19}H_{13}F_4IO^+$ 459.9942; Found 459.9960.

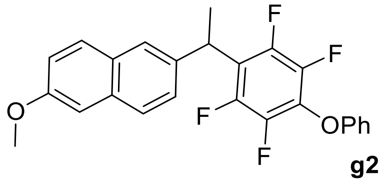

g2

2-methoxy-6-(1-(2,3,5,6-tetrafluoro-4-phenoxyphenyl)ethyl)naphthalene, synthesized via GP2, white solid, m.p. 120-121 °C ,36.6 mg (Yield = 86 %). ^{1}H NMR (400 MHz, $CDCl_3$) δ 7.81 – 7.67 (m, 3H), 7.41 (d, *J* = 8.5 Hz, 1H), 7.32 (t, *J* = 7.8 Hz, 2H), 7.20 – 7.02 (m, 3H), 6.97 (d, *J* = 8.1 Hz, 2H), 4.76 (q, *J* = 7.4 Hz, 1H), 3.92 (s, 3H), 1.89 (d, *J* = 7.4 Hz, 3H). ^{19}F NMR (376 MHz, $CDCl_3$) δ -142.78 – -142.93 (m), -154.55 – -154.70 (m). ^{13}C NMR (101 MHz, $CDCl_3$) δ 157.8, 157.4, 146.8(m), 144.2(m), 143.1(m), 140.6(m), 137.6, 133.6, 131.9(m), 129.9, 129.5, 129.0, 127.3, 126.5, 125.4, 123.7, 120.6(m), 119.2, 115.7, 105.7, 55.5, 34.8, 18.5. GC-MS: 426. HRMS (APPI/LTQ-Orbitrap) m/z: $[M]^+$ Calcd for $C_{25}H_{18}F_4O^+$ 426.1237; Found 426.1238.

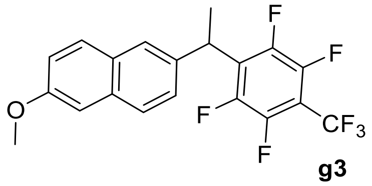

g3

2-methoxy-6-(1-(2,3,5,6-tetrafluoro-4-(trifluoromethyl)phenyl)ethyl)naphthalene, synthesized via GP2, white solid, m.p. 117-119 °C , 25.6 mg (Yield = 64 %). ^{1}H NMR (400 MHz, $CDCl_3$) δ 7.77 – 7.68 (m, 3H), 7.38 (d, *J* = 8.5 Hz, 1H), 7.25 – 7.11 (m, 2H), 4.81 (q, *J* = 7.4 Hz, 1H), 3.94 (s, 3H), 1.90 (d, *J* = 7.4 Hz, 3H). ^{19}F NMR (376 MHz, $CDCl_3$) δ -56.29 (t, *J* = 21.2 Hz), -140.55 (m), -140.67 – -140.99 (m). ^{13}C NMR (101 MHz, $CDCl_3$) δ 158.0, 146.6(m), 145.5(m), 144.1(m), 143.1(m), 136.3, 133.7, 129.5, 129.3(m), 127.5, 126.2, 125.5, 122.4, 119.7, 119.4, 108.1(m), 105.7, 55.5, 35.3, 18.1. GC-MS: 402. HRMS (APPI/LTQ-Orbitrap) m/z: $[M]^+$ Calcd for $C_{20}H_{13}F_7O^+$ 402.0849; Found 402.0854.

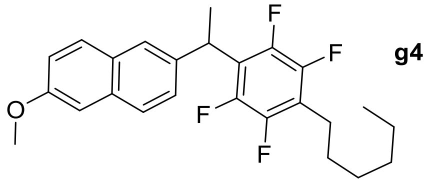

g4

2-methoxy-6-(1-(2,3,5,6-tetrafluoro-4-hexylphenyl)ethyl)naphthalene, synthesized via GP2, white solid, m.p. 66-68 °C , 24.6 mg (Yield = 64 %). ^{1}H NMR (400 MHz, $CDCl_3$) δ 7.77 – 7.67 (m, 3H), 7.42 (d, *J* = 8.5 Hz, 1H), 7.21 – 7.10 (m, 2H), 4.75 (q, *J* = 7.4 Hz, 1H), 3.94 (s, 3H), 2.70 (t, *J* = 7.7 Hz, 2H), 1.88 (d, *J* = 7.3 Hz, 3H), 1.60 (p, *J* = 7.3 Hz, 2H), 1.43 – 1.26 (m, 6H), 0.95 – 0.87 (m, 3H). ^{19}F NMR (376 MHz, $CDCl_3$) δ -144.31 (dd, *J* = 21.9, 12.3 Hz), -145.59 (dd, *J* = 21.9, 12.3 Hz). ^{13}C NMR (151 MHz, $CDCl_3$) δ 157.7, 145.9(m), 145.5(m), 144.3(m), 143.9(m), 138.0, 133.5, 129.4, 129.0, 127.1, 126.6, 125.3, 121.7(m), 119.1, 118.9(m), 105.7, 55.4, 34.8, 31.6, 29.4, 29.1, 22.8, 22.7, 18.5, 14.2. GC-MS: 418. HRMS (APPI/LTQ-Orbitrap) m/z: $[M]^+$ Calcd for $C_{25}H_{18}F_4O^+$ 418.1914; Found 418.1909.

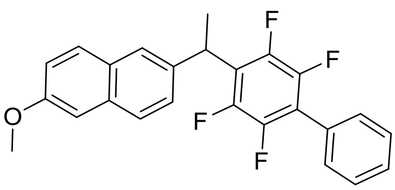

g5

2-methoxy-6-(1-(2,3,5,6-tetrafluoro-[1,1'-biphenyl]-4-yl)ethyl)naphthalene, synthesized via GP2, white solid, m.p. 175-176 °C , 31.8 mg (Yield = 78 %). ^{1}H NMR (400 MHz, $CDCl_3$) δ 7.79 – 7.67 (m, 3H), 7.45 (m, 6H), 7.22 – 7.09 (m, 2H), 4.80 (q, *J* = 7.4 Hz, 1H), 3.92 (s, 3H), 1.91 (d, *J* = 7.4 Hz, 3H). ^{19}F NMR (376 MHz, $CDCl_3$) δ -143.27 (dd, *J* = 22.2, 12.1 Hz), -144.55 (dd, *J* = 22.3, 12.0 Hz). ^{13}C NMR (101 MHz, $CDCl_3$) δ 157.8, 146.5(m), 145.2(m), 144.0(m), 142.8(m), 137.7, 133.6, 130.3, 129.5, 129.1, 129.0, 128.7, 127.7, 126.5, 125.4, 123.6(m), 119.2, 118.8(m), 105.7, 55.5, 35.1, 18.5. GC-MS: 410. HRMS (APPI/LTQ-Orbitrap) m/z: $[M]^+$ Calcd for $C_{25}H_{18}F_4O^+$ 410.1288; Found 410.1292.

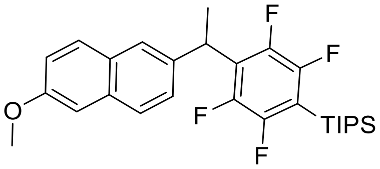

g6

triisopropyl(2,3,5,6-tetrafluoro-4-(1-(6-methoxynaphthalen-2-yl)ethyl)phenyl)silane, synthesized via GP2, white solid, m.p. 106-108 °C , 32.4 mg (Yield = 66 %). ^{1}H NMR (400 MHz, $CDCl_3$) δ 7.79 – 7.67 (m, 3H), 7.45 (d, *J* = 8.5 Hz, 1H), 7.19 – 7.09 (m, 2H), 4.77 (q, *J* = 7.4 Hz, 1H), 3.91 (s, 3H), 1.89 (d, *J* = 7.3 Hz, 3H), 1.63 – 1.47 (m, 3H), 1.09 (d, *J* = 7.4 Hz, 18H). ^{19}F NMR (376 MHz, $CDCl_3$) δ -125.57 – -125.76 (m), -142.58 – -142.76 (m). ^{13}C NMR (101 MHz, $CDCl_3$) δ 157.8, 150.7(m), 148.3(m), 145.9(m), 143.5(m), 137.8, 133.6, 129.5, 129.0, 127.2, 126.7(m), 125.9, 125.6, 119.1, 110.9(m), 105.7, 55.4, 35.3, 18.7, 18.5, 12.3. GC-MS: 490. HRMS (APPI/LTQ-Orbitrap) m/z: $[M]^+$ Calcd for $C_{28}H_{34}F_4OSi^+$ 490.2310; Found 490.2315.

g7

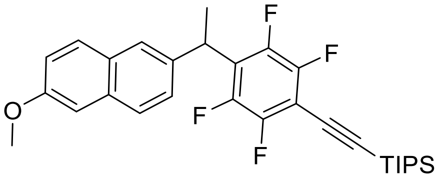

triisopropyl((2,3,5,6-tetrafluoro-4-(1-(6-methoxynaphthalen-2-yl)ethyl)phenyl)ethynyl)silane, synthesized via GP2, colorless liquid, 36.8 mg (Yield = 72 %). ^{1}H NMR (400 MHz, $CDCl_3$) δ 7.76 – 7.64 (m, 3H), 7.35 (d, *J* = 8.5 Hz, 1H), 7.21 – 7.08 (m, 2H), 4.75 (q, *J* = 7.4 Hz, 1H), 3.91 (s, 3H), 1.85 (d, *J* = 7.3 Hz, 3H), 1.14 (m, 21H). ^{19}F NMR (376 MHz, $CDCl_3$) δ -136.93 – -137.12 (m), -143.27 (dd, *J* = 21.9, 11.8 Hz). ^{13}C NMR (101 MHz, $CDCl_3$) δ 157.8, 148.7(m), 146.2(m), 143.6(m), 137.3, 133.6, 129.5, 129.0, 127.2, 126.4, 125.3, 119.2, 105.7, 105.6, 102.9(m), 90.9, 55.4, 35.0, 18.6, 18.3, 11.2. GC-MS: 514. HRMS (APPI/LTQ-Orbitrap) m/z: $[M]^+$ Calcd for $C_{25}H_{18}F_4O^+$ 514.2310; Found 514.2304.

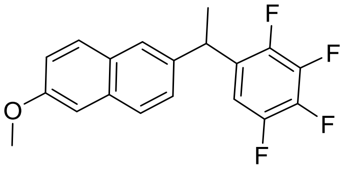

g8

2-methoxy-6-(1-(2,3,4,5-tetrafluorophenyl)ethyl)naphthalene, synthesized via GP2, oil, 22.1 mg (Yield = 66 %). ^{1}H NMR (400 MHz, $CDCl_3$) δ 7.70 (m, 2H), 7.63 – 7.60 (m, 1H), 7.27 – 7.23 (m, 1H), 7.16 (dd, *J* = 8.9, 2.6 Hz, 1H), 7.12 (d, *J* = 2.6 Hz, 1H), 6.85 – 6.69 (m, 1H), 4.59 (q, *J* = 7.2 Hz, 1H), 3.92 (s, 3H), 1.69 (d, *J* = 7.2 Hz, 3H). ^{19}F NMR (376 MHz, $CDCl_3$) δ -139.6 (dd, *J* = 21.2, 12.3 Hz), -143.7 (ddd, *J* = 20.2, 12.2, 2.5 Hz), -156.0 (t, *J* = 20.0 Hz), -158.9 – -159.1 (m). ^{13}C NMR (101 MHz, $CDCl_3$) δ 157.9, 148.3(m), 146.7(m), 145.9(m), 144.1(m), 142.1(m), 140.2(m), 139.6(m), 138.3, 137.7(m), 133.6, 130.2(m), 129.4, 129.0, 127.4, 126.7, 125.5, 119.3, 109.8(m), 105.8, 55.5, 37.3, 20.6. GC-MS: 334. HRMS (APPI/LTQ-Orbitrap) m/z: $[M + H_{-1}]^+$ Calcd for $C_{19}H_{13}F_4O^+$ 333.0897; Found 333.0904.

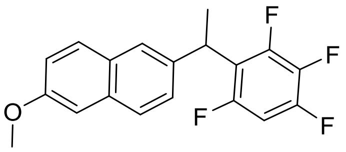

g9

2-methoxy-6-(1-(2,3,4,6-tetrafluorophenyl)ethyl)naphthalene, synthesized via GP2, white solid, m.p. 62-64 °C , 27.8 mg (Yield = 83 %). ^{1}H NMR (400 MHz, $CDCl_3$) δ 7.74 – 7.64 (m, 3H), 7.40 – 7.33 (m, 1H), 7.15 (dd, *J* = 8.9, 2.6 Hz, 1H), 7.11 (d, *J* = 2.5 Hz, 1H), 6.74 (m, 1H), 4.69 (q, *J* = 7.4 Hz, 1H), 3.92 (s, 3H), 1.84 (d, *J* = 7.3, 3H). ^{19}F NMR (376 MHz, $CDCl_3$) δ -116.9 (d, *J* = 10.8 Hz), -134.8 (dd, *J* = 20.9, 5.3 Hz), -135.4 (dd, *J* = 21.3, 5.0 Hz), -165.0 (td, *J* = 21.0, 10.7 Hz). ^{13}C NMR (101 MHz, $CDCl_3$) δ 157.7, 156.6(m), 154.1(m), 151.4(m), 150.5(m), 148.9(m), 148.0(m), 138.7(m), 138.0, 136.2(m), 133.5, 129.4, 129.0, 127.1, 126.5, 125.2, 119.5(m), 119.1, 105.7, 101.1(m), 55.4, 34.4, 18.5. GC-MS: 334. HRMS (APPI/LTQ-Orbitrap) m/z: $[M + H_{-1}]^+$ Calcd for $C_{19}H_{13}F_4O^+$ 333.0897; Found 333.0906.

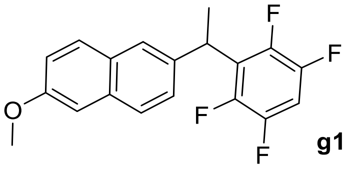

g10

2-methoxy-6-(1-(2,3,5,6-tetrafluorophenyl)ethyl)naphthalene, synthesized via GP2, white solid, m.p. 93-95°C , 28.0 mg (Yield = 84 %). ^{1}H NMR (400 MHz, $CDCl_3$) δ 7.75 – 7.65 (m, 3H), 7.39 (dd, *J* = 8.5, 1.9 Hz, 1H), 7.16 (ddd, *J* = 8.9, 2.6, 0.9 Hz, 1H), 7.11 (d, *J* = 2.5 Hz, 1H), 6.93 (tt, *J* = 9.7, 7.3 Hz, 1H), 4.77 (q, *J* = 7.4 Hz, 1H), 3.91 (s, 3H), 1.87 (d, *J* = 7.4, 3H). ^{19}F NMR (376 MHz, $CDCl_3$) δ -139.4 (dd, *J* = 21.8, 12.6 Hz), -143.0 (dd, *J* = 21.8, 12.7 Hz). ^{13}C NMR (101 MHz, $CDCl_3$) δ 157.8, 147.4(m), 146.1(m), 145.0(m), 143.7(m), 137.5, 133.5, 129.5, 129.0, 127.2, 126.5, 125.4(m), 125.3, 119.1, 105.7, 104.0(m), 55.4, 35.0, 18.3. GC-MS: 334. HRMS (APPI/LTQ-Orbitrap) m/z: $[M + H_{-1}]^+$ Calcd for $C_{19}H_{13}F_4O^+$ 333.0897; Found 333.0908

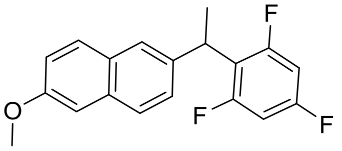

g11

2-methoxy-6-(1-(2,4,6-trifluorophenyl)ethyl)naphthalene, synthesized via GP2, white solid, m.p. 70-72°C , 22.5 mg (Yield = 71 %). ^{1}H NMR (400 MHz, $CDCl_3$) δ 7.64 – 7.53 (m, 3H), 7.27 (dd, *J* = 8.5, 1.9 Hz, 1H), 7.09 – 6.98 (m, 2H), 6.53 (t, *J* = 8.5 Hz, 2H), 4.58 (q, *J* = 7.3 Hz, 1H), 3.81 (s, 3H), 1.72 (d, *J* = 7.4 Hz, 3H). ^{19}F NMR (376 MHz, $CDCl_3$) δ -110.2 (d, *J* = 5.7 Hz), -111.2 (t, *J* = 6.0 Hz). ^{13}C NMR (101 MHz, $CDCl_3$) δ 162.5(m), 160.1(m), 157.6, 138.8, 133.3, 129.4,

129.0, 126.9, 126.7, 125.1, 118.9, 118.2(m), 105.7, 100.6(m), 55.4, 33.9, 18.7. GC-MS: 316. HRMS (APPI/LTQ-Orbitrap) m/z: [M]+ Calcd for $C_{19}H_{15}F_3O^+$ 316.1070; Found 316.1078.

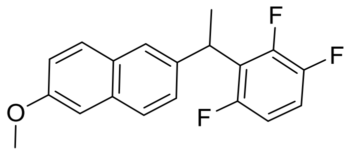

g12

2-methoxy-6-(1-(2,3,6-trifluorophenyl)ethyl)naphthalene, synthesized via GP2, white solid, m.p. 83-84 °C , 20.1 mg (Yield = 64 %). ^{1}H NMR (400 MHz, $CDCl_3$) δ 7.74 – 7.63 (m, 3H), 7.38 (dd, *J* = 8.5, 1.9 Hz, 1H), 7.17 – 7.07 (m, 2H), 6.98 (qd, *J* = 9.2, 4.9 Hz, 1H), 6.78 (m, 1H), 4.73 (q, *J* = 7.3 Hz, 1H), 3.90 (s, 3H), 1.84 (d, *J* = 7.3, 3H). ^{19}F NMR (376 MHz, $CDCl_3$) δ -118.5 (dd, *J* = 14.9, 3.3 Hz), -137.0 (d, *J* = 21.7 Hz), -142.3 (dd, *J* = 20.6, 14.9 Hz). ^{13}C NMR (101 MHz, $CDCl_3$) δ 157.7, 155.4(m), 150.5(m), 148.7(m), 146.3(m), 138.3, 133.4, 129.4, 129.0, 127.0, 126.7, 125.2, 124.0(m), 119.0, 114.7(m), 111.2(m), 105.7, 55.4, 34.7, 18.5. GC-MS: 316. HRMS (APPI/LTQ-Orbitrap) m/z: [M]+ Calcd for $C_{19}H_{15}F_3O^+$ 316.1070; Found 316.1076.

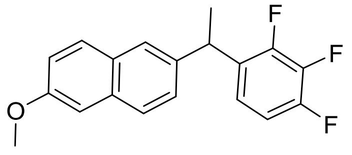

g13

2-methoxy-6-(1-(2,3,6-trifluorophenyl)ethyl)naphthalene, synthesized via GP2, oil, 18.2 mg (Yield = 58 %). ^{1}H NMR (400 MHz, $CDCl_3$) δ 7.68 (dd, *J* = 11.1, 8.7 Hz, 2H), 7.63 – 7.60 (m, 1H), 7.30 – 7.25 (m, 1H), 7.15 (dd, *J* = 8.8, 2.5 Hz, 1H), 7.11 (d, *J* = 2.6 Hz, 1H), 6.94 – 6.83 (m, 2H), 4.57 (q, *J* = 7.2 Hz, 1H), 3.91 (s, 3H), 1.70 (d, *J* = 7.2 Hz, 3H). ^{19}F NMR (376 MHz, $CDCl_3$) δ -137.5 – -137.9 (m), -138.8 (dd, *J* = 20.6, 6.8 Hz), -160.8 (d, *J* = 20.6 Hz). ^{13}C NMR (101 MHz, $CDCl_3$) δ 157.7, 150.8(m), 148.3(m), 141.3(m), 139.2, 138.8(m), 133.5, 131.2(m), 129.4, 129.0, 127.2, 126.9, 125.4, 122.0(m), 119.1, 111.9(m), 105.8, 55.5, 37.4, 20.8. GC-MS: 316. HRMS (APPI/LTQ-Orbitrap) m/z: [M]+ Calcd for $C_{19}H_{15}F_3O^+$ 316.1070; Found 316.1072.

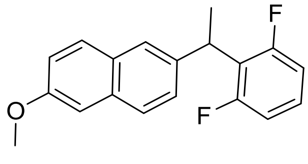

g14

2-methoxy-6-(1-(2,6-difluorophenyl)ethyl)naphthalene, synthesized via GP2, white solid, m.p. 93-95 °C , 18.7 mg (Yield = 63 %). ^{1}H NMR (400 MHz, $CDCl_3$) δ 7.71 (m, 2H), 7.66 (d, *J* = 8.5 Hz, 1H), 7.44 – 7.37 (m, 1H), 7.18 – 7.08 (m, 3H), 6.85 (t, *J* = 8.3 Hz, 2H), 4.74 (q, *J* = 7.4 Hz, 1H), 3.90 (s, 3H), 1.84 (d, *J* = 7.4 Hz, 3H). ^{19}F NMR (376 MHz, $CDCl_3$) δ -113.2 (s). ^{13}C NMR (101 MHz, $CDCl_3$) δ 162.7(m), 160.2(m), 157.5, 139.1, 133.3, 129.4, 129.0, 127.9(m), 126.9, 126.8, 125.1, 122.0(m), 118.8, 111.7(m), 105.7, 55.4, 34.1, 18.7. GC-MS: 298. HRMS (APPI/LTQ-Orbitrap) m/z: [M + H]+ Calcd for $C_{19}H_{17}F_2O^+$ 299.1242; Found 299.1243.

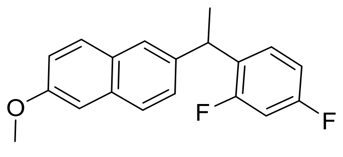

g15

2-methoxy-6-(1-(2,4-difluorophenyl)ethyl)naphthalene, synthesized via GP2, liquid, 9.0 mg (Yield = 30 %). ^{1}H NMR (400 MHz, $CDCl_3$) δ 7.67 (m, 2H), 7.63 – 7.60 (m, 1H), 7.28 (dd, *J* = 8.5, 1.9 Hz, 1H), 7.19 – 7.10 (m, 3H), 6.87 – 6.72 (m, 2H), 4.56 (q, *J* = 7.2 Hz, 1H), 3.91 (s, 3H), 1.69

(d, J = 7.2 Hz, 3H). ^{19}F NMR (376 MHz, $CDCl_3$) δ -113.6 (d, J = 6.9 Hz), -113.9 (d, J = 7.1 Hz). ^{13}C NMR (101 MHz, $CDCl_3$) δ 162.7(m), 161.8(m), 160.4(m), 159.4(m), 157.6, 140.0, 133.4, 129.5(m), 129.4, 129.1, 127.1, 127.1, 125.3, 119.0, 111.3(m), 105.8, 103.9(m), 55.5, 37.2, 20.9. GC-MS: 298. HRMS (APPI/LTQ-Orbitrap) m/z: $[M + H]^+$ Calcd for $C_{19}H_{17}F_2O^+$ 299.1242; Found 299.1246.

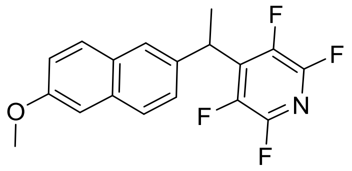

g18

2,3,5,6-tetrafluoro-4-(1-(6-methoxynaphthalen-2-yl)ethyl)pyridine, synthesized via GP2, white solid, m.p. 106-108 °C ,, 10.1 mg (Yield = 29 %). ^{1}H NMR (400 MHz, $CDCl_3$) δ 7.76 – 7.68 (m, 3H), 7.36 (dd, J = 8.4, 1.9 Hz, 1H), 7.16 (dd, J = 9.0, 2.5 Hz, 1H), 7.11 (d, J = 2.5 Hz, 1H), 4.79 (q, J = 7.3 Hz, 1H), 3.91 (s, 3H), 1.89 (d, J = 7.4, 3H). ^{19}F NMR (376 MHz, $CDCl_3$) δ -91.1 – -91.3 (m), -143.6 – -143.8 (m). ^{13}C NMR (101 MHz, $CDCl_3$) δ 158.1, 145.1(m), 142.6(m), 141.6(m), 139.2(m), 138.6(m), 135.7, 133.9, 129.5, 129.0, 127.6, 126.1, 125.7, 119.5, 105.7, 55.5, 35.9, 17.8. GC-MS: 335. HRMS (APPI/LTQ-Orbitrap) m/z: $[M + H]^+$ Calcd for $C_{18}H_{14}F_4NO^+$ 336.1006; Found 336.1008.

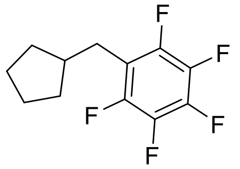

h1

1-(cyclopentylmethyl)-2,3,4,5,6-pentafluorobenzene, synthesized via GP1, colorless liquid, 15.0 mg (Yield = 60 %). ^{1}H NMR (400 MHz, $CDCl_3$) δ 2.69 (d, J = 7.5 Hz, 1H), 2.08 (h, J = 7.7 Hz, 1H), 1.77 – 1.62 (m, 4H), 1.61 – 1.48 (m, 2H), 1.21 (m, 2H). ^{19}F NMR (376 MHz, $CDCl_3$) δ -143.5 (m), -158.4 (t, J = 20.9 Hz), -163.2(m). ^{13}C NMR (101 MHz, $CDCl_3$) δ 146.5(m), 144.0(m), 140.7(m), 138.7(m), 138.2(m), 136.4(m), 115.3(m), 40.3, 32.3, 27.9, 24.9. GC-MS: 250. HRMS (EI/LTQ-Orbitrap) m/z: $[M]^+$ Calcd for $C_{12}H_{11}F_5^+$ 250.0775; Found 250.0778.

5. Mechanistic study

5.1. UV-Vis spectroscopy

All solutions and samples were prepared in the glovebox, including the stock solutions of (diglyme)Zn(C_6F_5)$_2$ (21.3 mg in 10.0 mL DCM, 4.0×10^{-3} M), (bpy)Cu(C_6F_5) (15.4 mg in 10.0 mL DCM, 4.0×10^{-3} M) and (bpy)Cu(OTf)$_2$ (14.4 mg Cu(OTf)$_2$, 6.3 mg bipyridine in 10.0 mL DCM, 4.0×10^{-3} M). The sample solutions were prepared by mixing a given volume of stock solutions, and then diluted to 2.0 mL with DCM. The sample solution was placed in a cuvette with screw cap for further measurements on the UV-Vis spectrometer.

The UV-Vis spectra of the mixtures of (bpy)Cu(C_6F_5) and (diglyme)Zn(C_6F_5)$_2$ were measured (Figure S4). (diglyme)Zn(C_6F_5)$_2$ had almost no absorption in the range 350 -600 nm, while (bpy)Cu(C_6F_5) had a strong absorption. With the increasing amount of (diglyme)Zn(C_6F_5)$_2$, the absorbance in this region decreased and converged when the ratio was above 1:1. This could indicate the conversion of (bpy)Cu(C_6F_5) to another species during the addition of (diglyme)Zn(C_6F_5)$_2$.

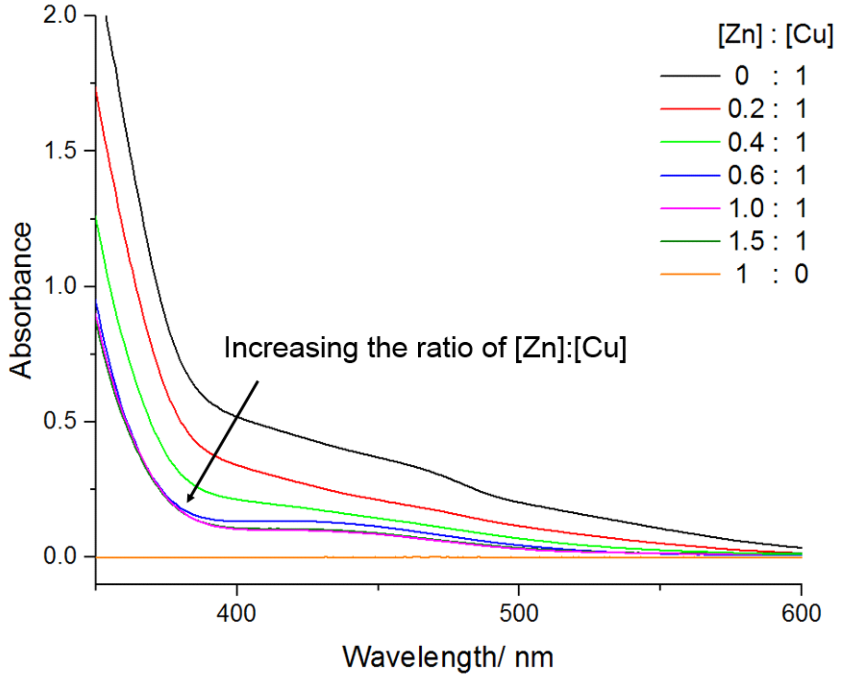

Figure S4 UV-Vis spectra of (diglyme)Zn(C_6F_5)$_2$ (**b1**), [(bpy)Cu(C_6F_5)], and their mixtures. c(**b1**) was 0.0008 M when alone. In all other samples, c[(bpy)Cu(C_6F_5)] was 0.0008 M and c(**b1**) was adjusted according to the given ratio.

5.2. Cyclic voltammograms of reagents

The CVs were recorded in DCM-CH_3CN (2:1, 4 mL) in a four-neck flask, with Bu_4NBF_4 (0.03 M) as electrolyte, glassy carbon disk as working electrode (diameter, 3 mm), Pt wire as counter electrode, SSCE as reference electrode. The device was assembled in the glovebox to guarantee an inert atmosphere. The scan rate for CV was 100 mV/s.

E(Fc^+/Fc) comparing to the reference electrode was 0.227 V (determined with 0.003 M Fc). All the potential values were converted using E(Fc^+/Fc) as reference.

5.3 Stern-Volmer quenching

All solutions and samples were prepared in a glovebox, including the stock solutions of 4CzIPN (3.2 mg in 10.0 mL DCM, 4.0×10^{-4} M), **a1** (37.8 mg in 10.0 mL DCM, 10^{-2} M), (diglyme)Zn(C_6F_5)$_2$ (21.3 mg in 10.0 mL DCM, 4.0×10^{-3} M), (bpy)Cu(C_6F_5) (15.4 mg in 10.0 mL DCM, 4.0×10^{-3} M). The sample solutions were prepared by mixing given volume of stock solutions, and then diluted to 2.0 mL with DCM. The sample solution was placed in a cuvette with screw cap for further measurements on a fluorescence spectrometer. Parameters: exciting wavelength (420 nm, slit 2.5 nm), emission measurement (430 nm-700 nm).

Neither **a1** nor (diglyme)Zn(C_6F_5)$_2$ could effectively quench the excited 4CzIPN (Figure S5). The addition of these reagents to the solution of 4CzIPN didn't affect the emission spectra of the photocatalyst under the same excitation conditions.

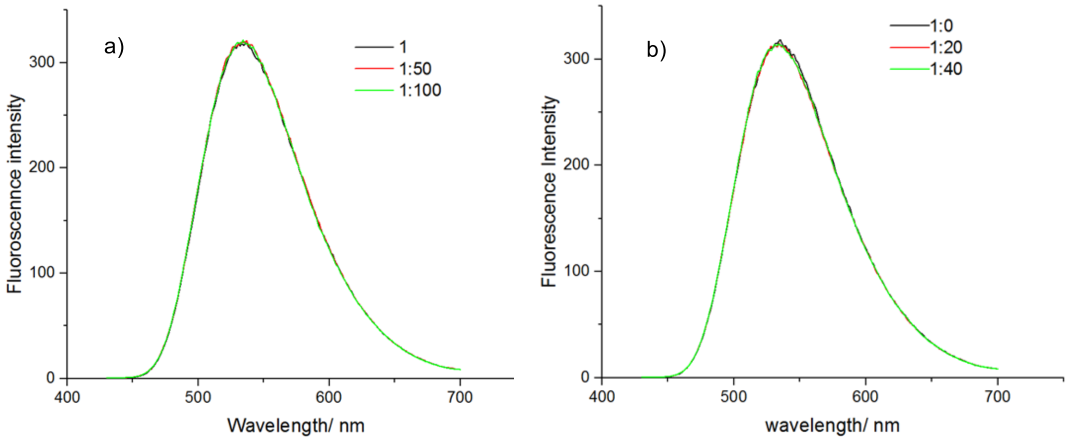

Figure S5 a) Emission spectra at different ratios of 4CzIPN to NHPI ester. b) Fluoroscence spectra at different ratios of 4CzIPN to (diglyme)Zn(C_6F_5)$_2$. c[4CzIPN]= 8 x 10^{-5} M.

When [(bpy)Cu(C_6F_5)] was added to the solution of 4CzIPN, the emission was weakened (Figure S6). The slope for Stern-Volmer quenching plots is 6226 M^{-1} for the copper complex.

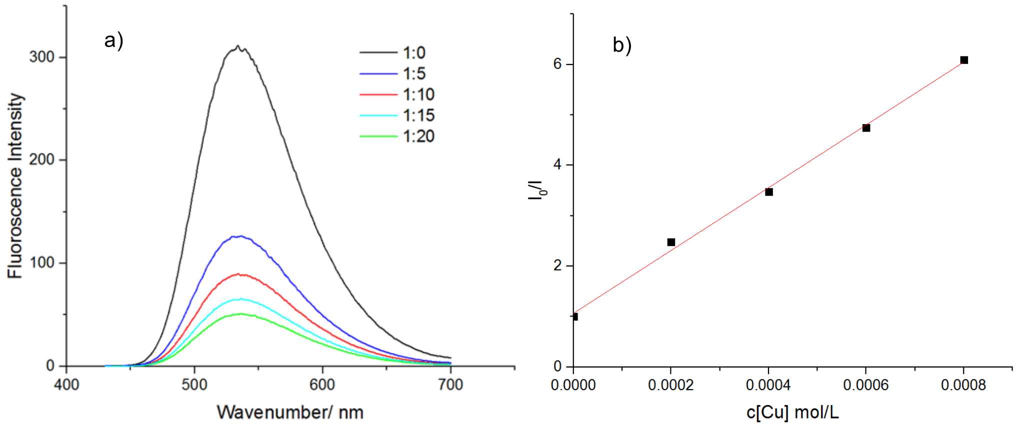

Figure S6. a) Emission spectra at different ratios of 4CzIPN to (bpy)Cu(C_6F_5). c[4CzIPN]= 8 x 10^{-5} M. b) Stern-Volmer quenching plot for (bpy)Cu(C_6F_5).

When (bpy)Cu(C_6F_5) and (diglyme)Zn(C_6F_5)$_2$ in equal amounts were added to the solution of 4CzIPN,

the emission was also weakened (Figure S7). The Stern-Volmer quenching plot deviated from linearity, but still indicated effective quenching.

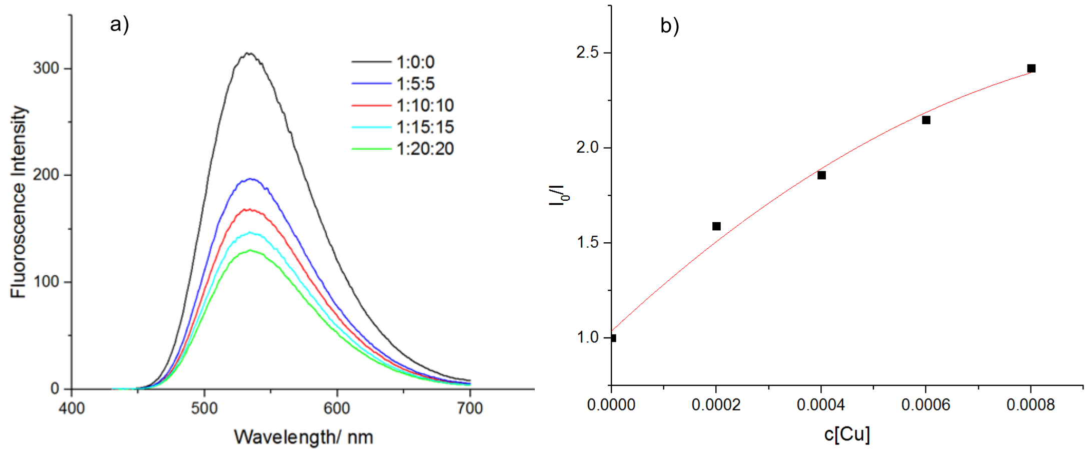

Figure S7. a) Emission spectra at different ratios of 4CzIPN to (bpy)Cu(C_6F_5) &(diglyme)Zn(C_6F_5)$_2$. c[4CzIPN]= 8 x 10^{-5} M. b) Stern-Volmer quenching plot in relative to the concentration of (bpy)Cu(C_6F_5).

6. NMR spectra of selected substrates and all products

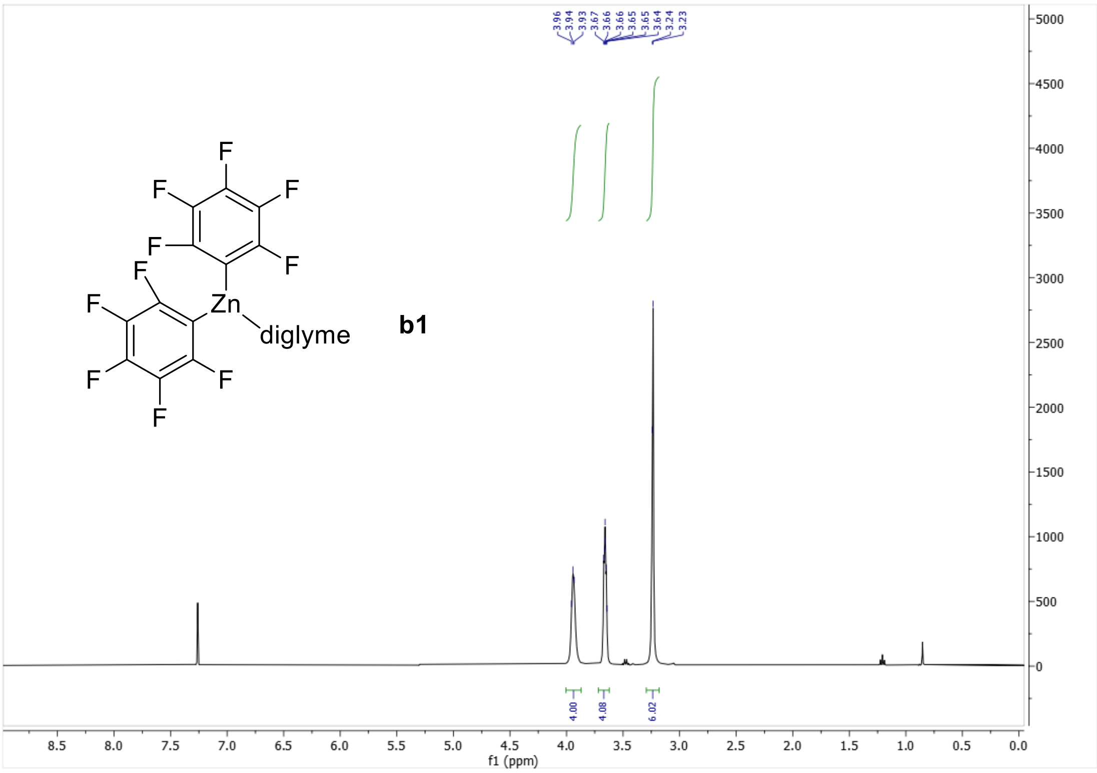

^{1}H-NMR spectrum of (diglyme)Zn(C_6F_5)$_2$ (**b1**) (400 MHz, $CDCl_3$)

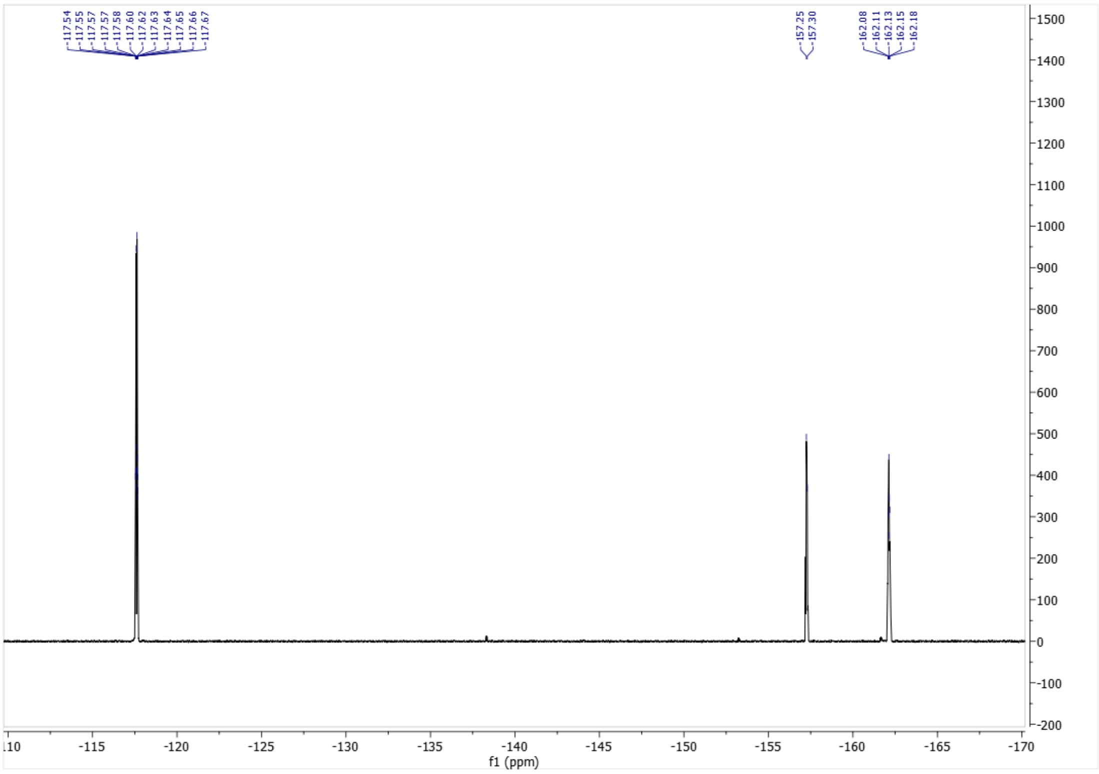

^{19}F-NMR spectrum of (diglyme)Zn(C_6F_5)$_2$ (**b1**) (400 MHz, $CDCl_3$)

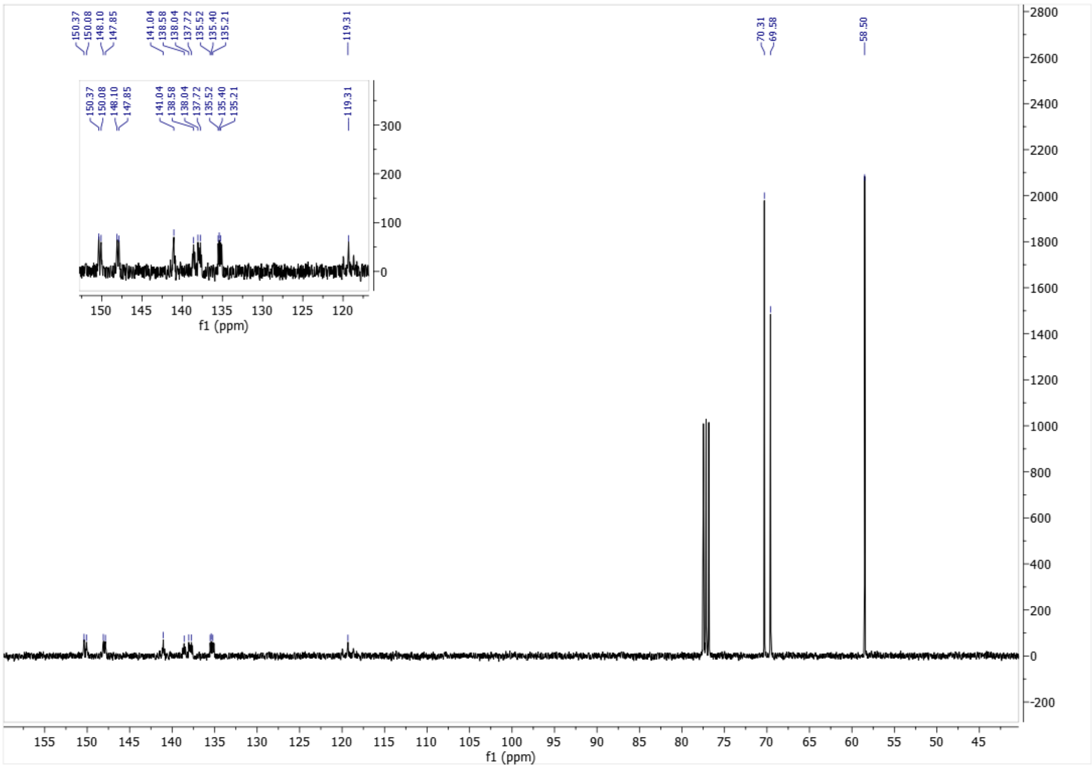

^{13}C-NMR spectrum of (diglyme)Zn(C_6F_5)$_2$ (**b1**) (400 MHz, $CDCl_3$)

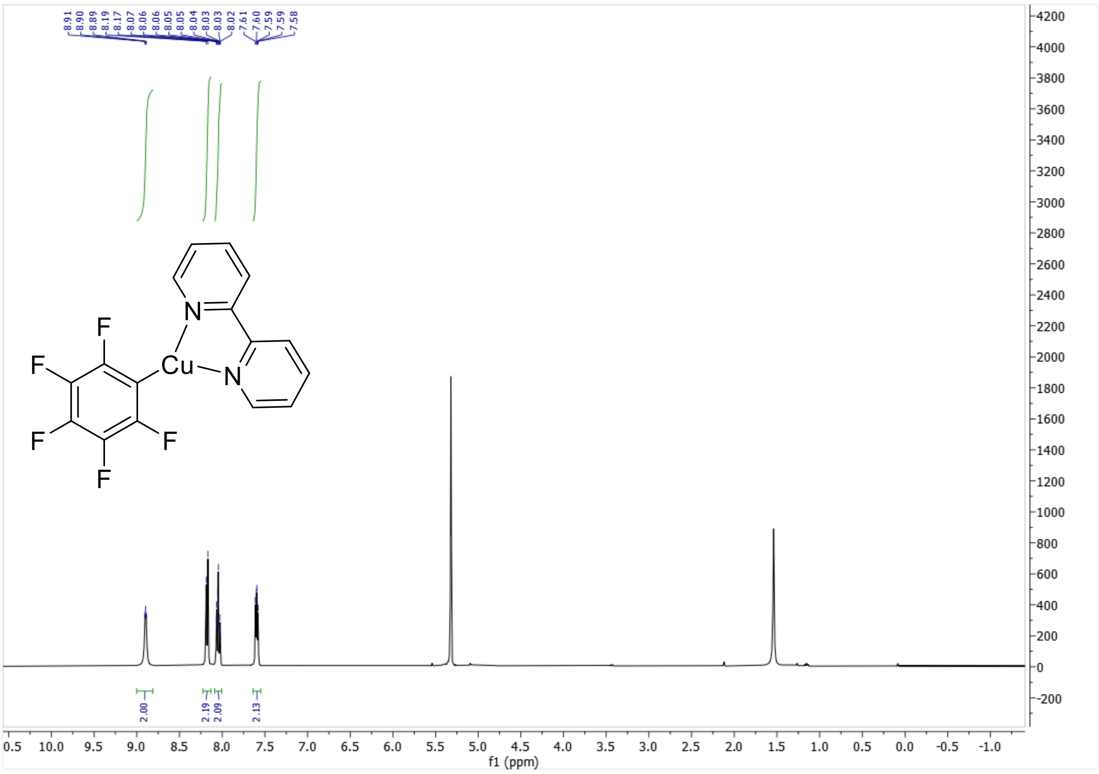

^{1}H-NMR spectrum of (bpy)CuC$_6$F$_5$ (400 MHz, CD$_2$Cl$_2$)

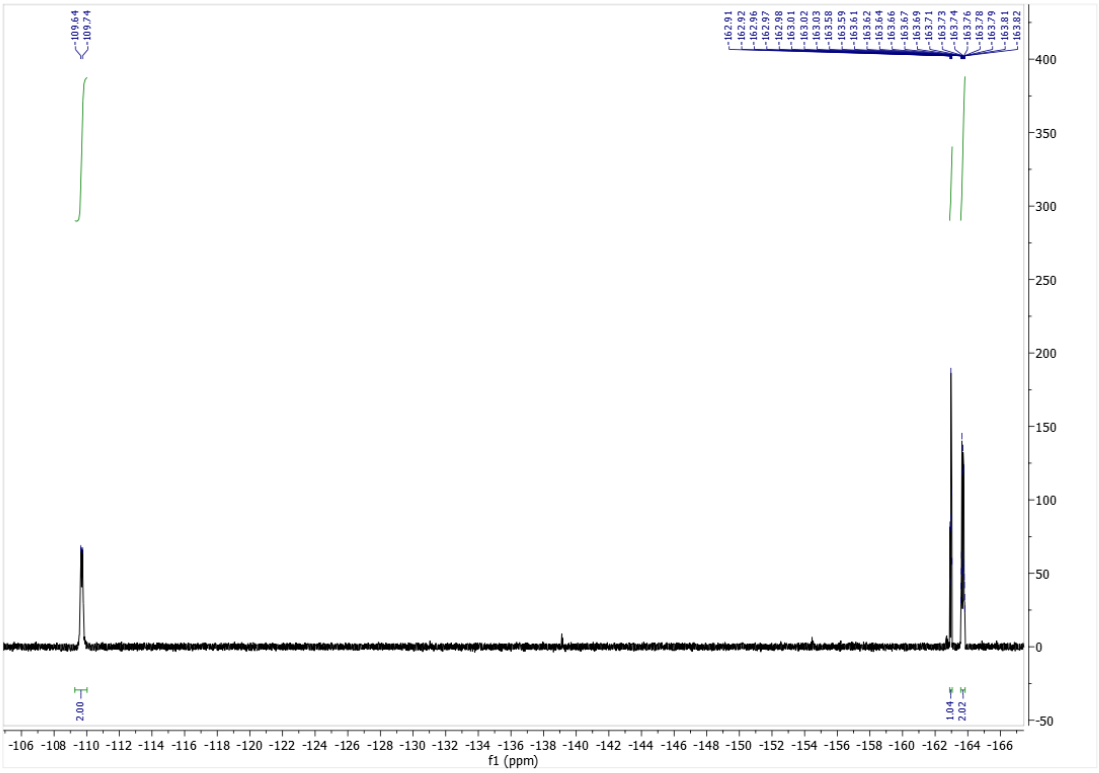

^{19}F-NMR spectrum of (bpy)CuC$_6$F$_5$ (400 MHz, CD$_2$Cl$_2$)

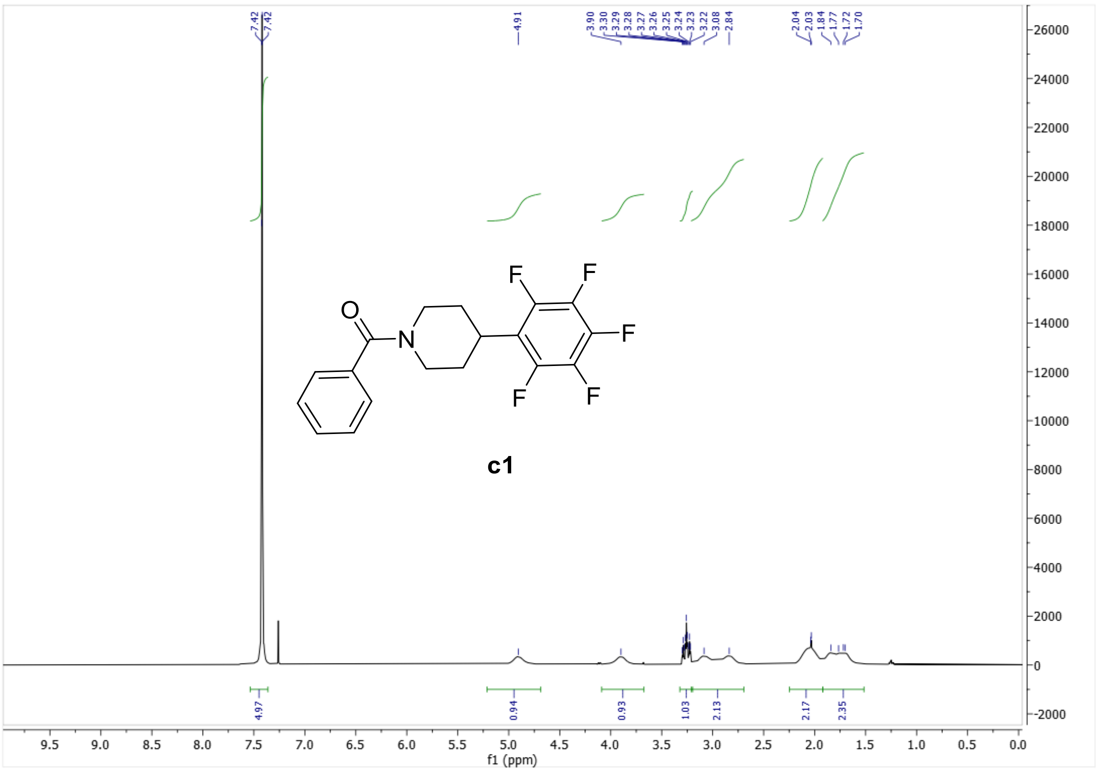

^{1}H-NMR spectrum of (**c1**) (400 MHz, $CDCl_3$)

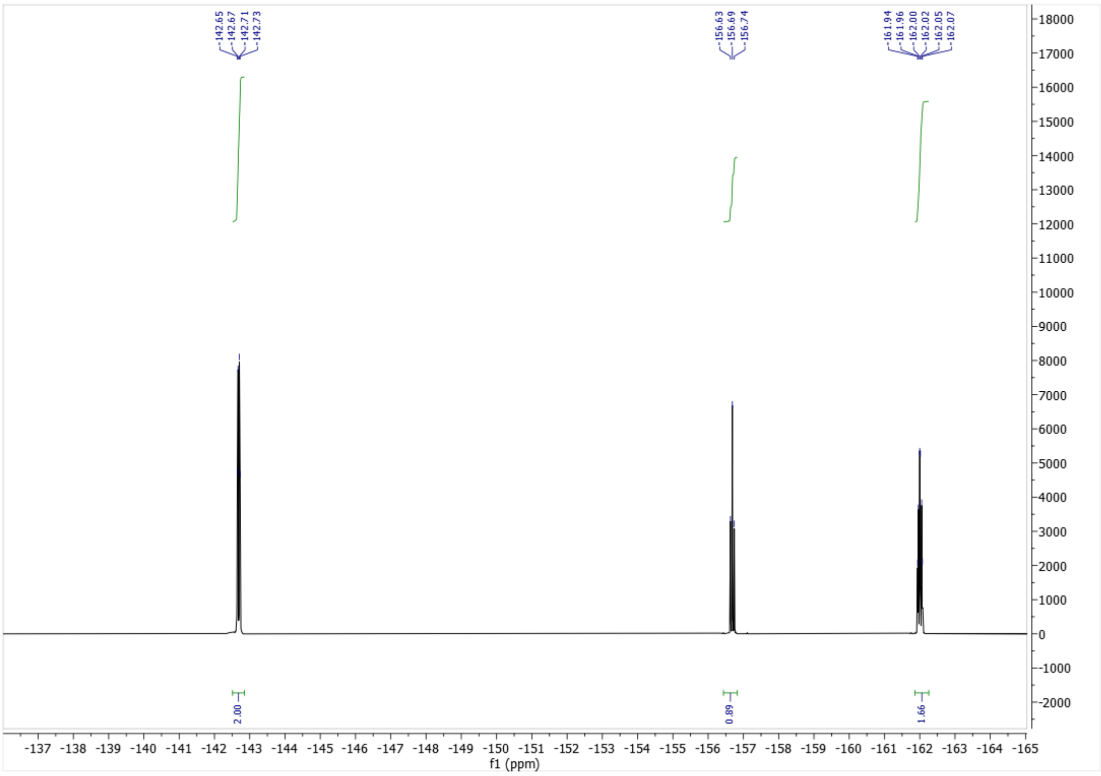

^{19}F-NMR spectrum of (**c1**) (400 MHz, $CDCl_3$)

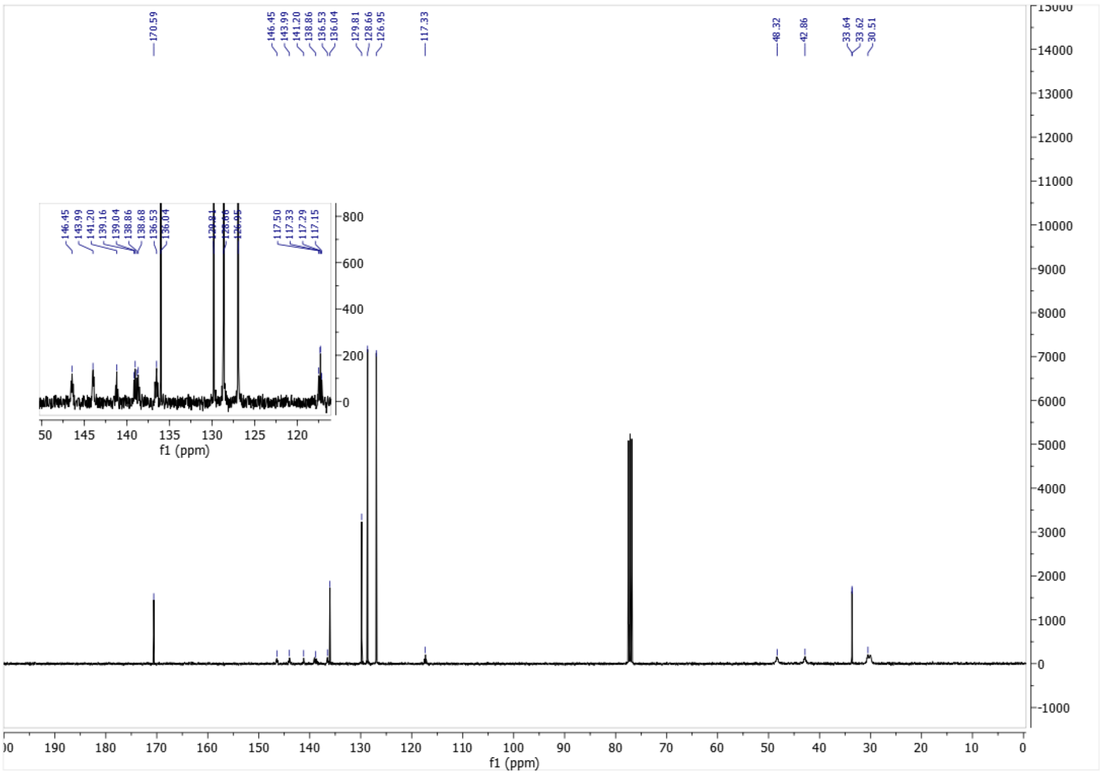

^{13}C-NMR spectrum of (**c1**) (400 MHz, $CDCl_3$)

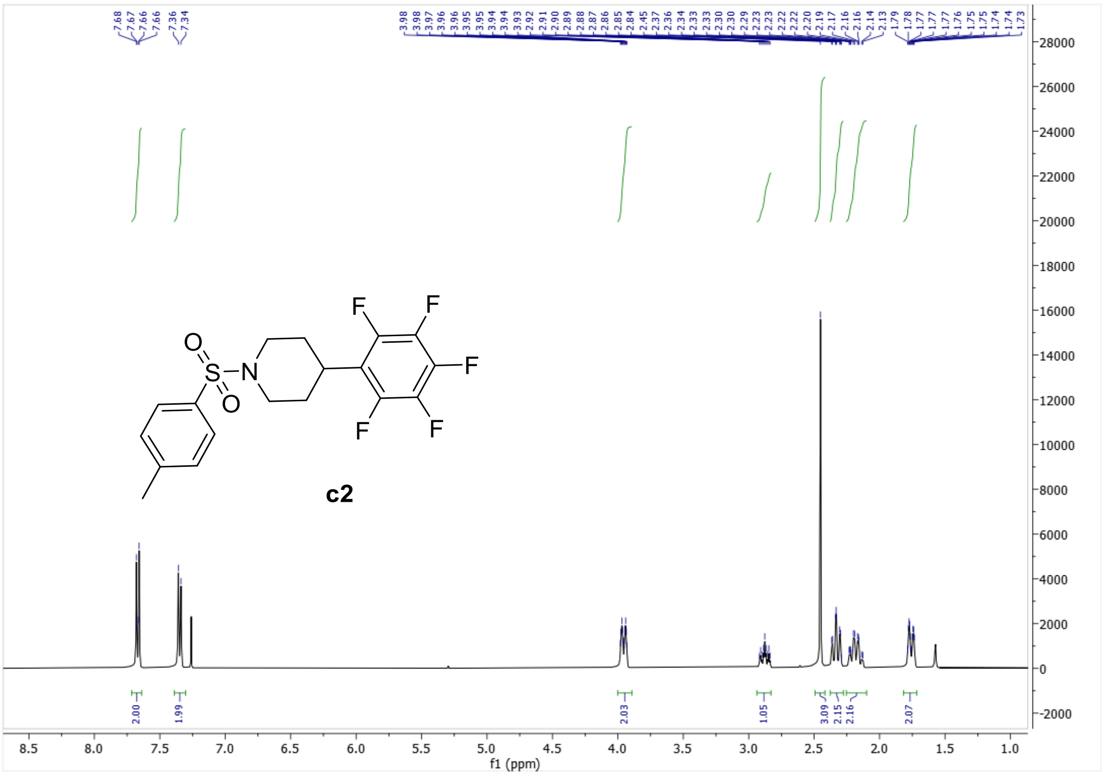

^{1}H-NMR spectrum of (**c2**) (400 MHz, $CDCl_3$)

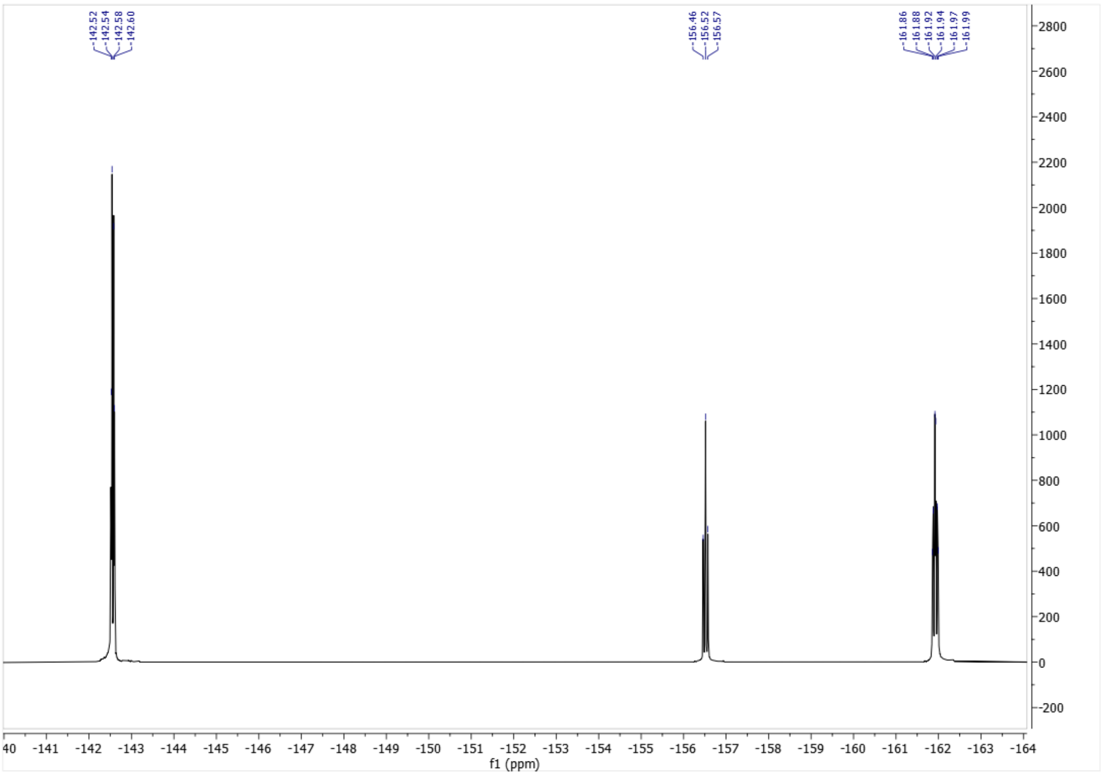

^{19}F-NMR spectrum of (**c2**) (400 MHz, $CDCl_3$)

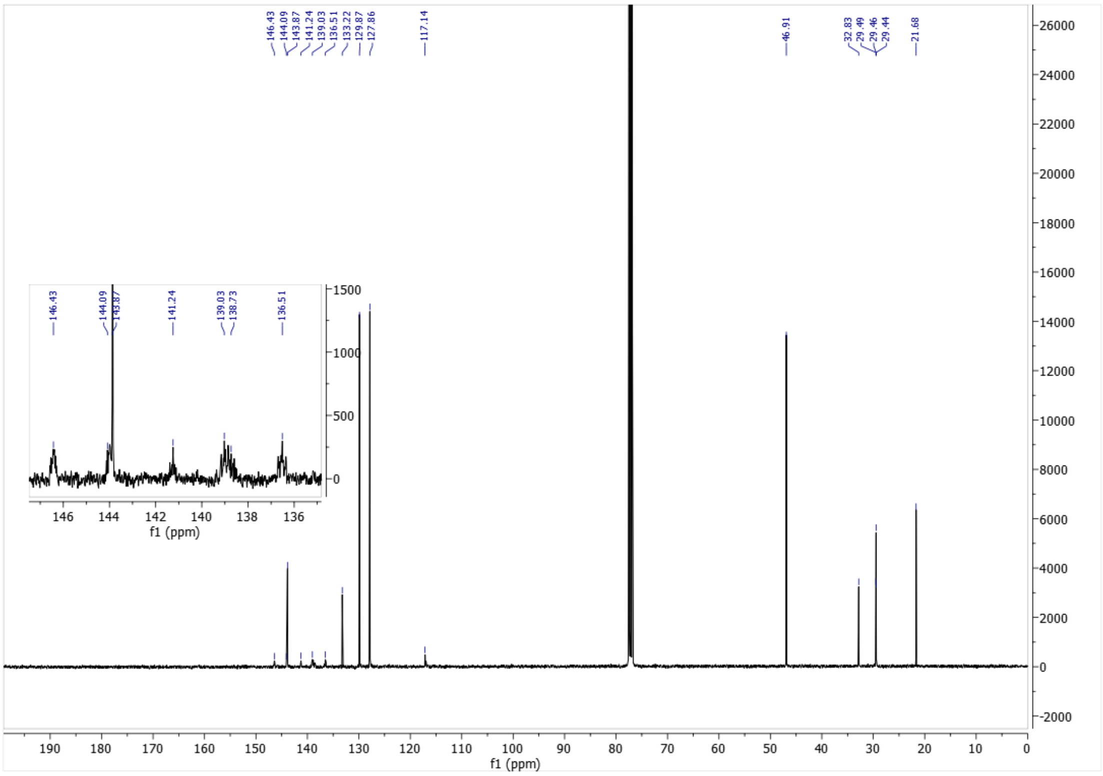

^{13}C-NMR spectrum of (**c2**) (400 MHz, $CDCl_3$)

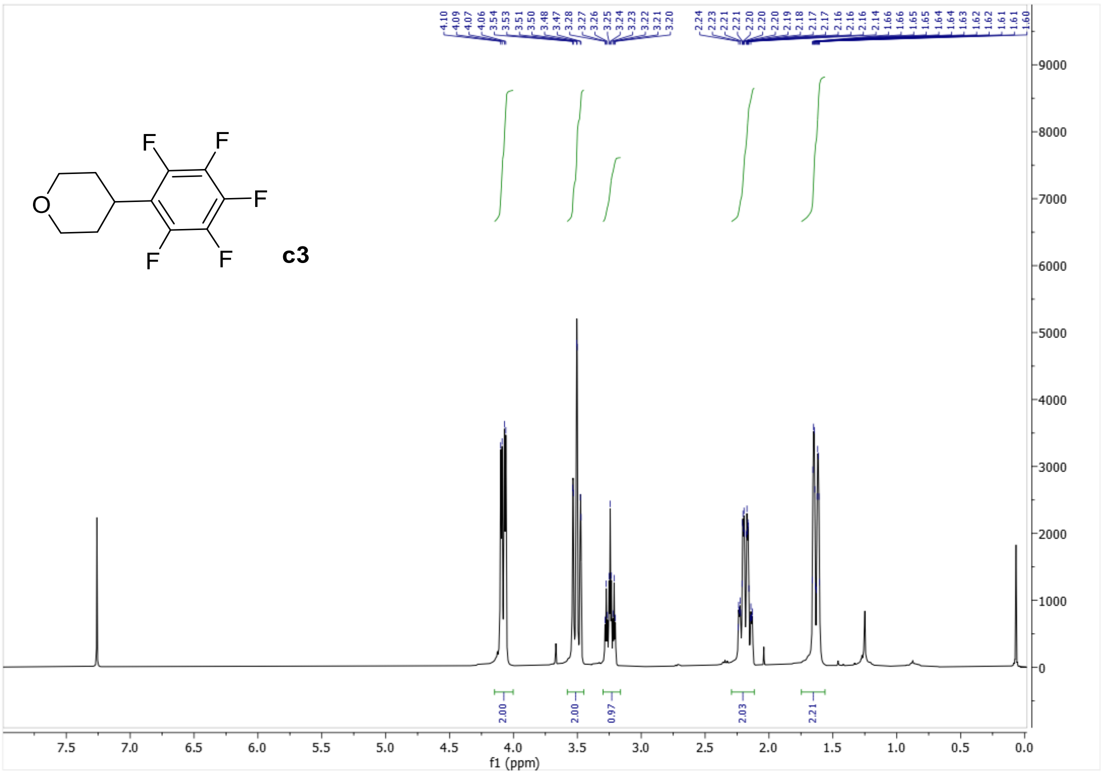

^{1}H-NMR spectrum of (**c3**) (400 MHz, $CDCl_3$)

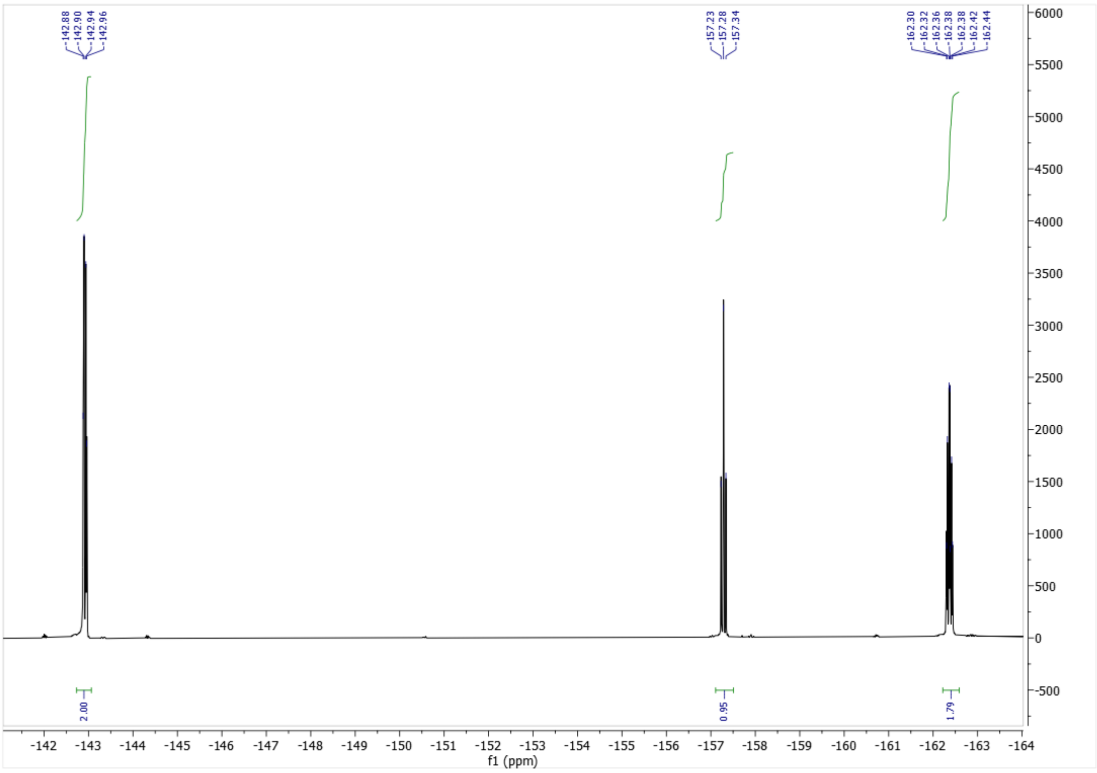

^{19}F-NMR spectrum of (**c3**) (400 MHz, $CDCl_3$)

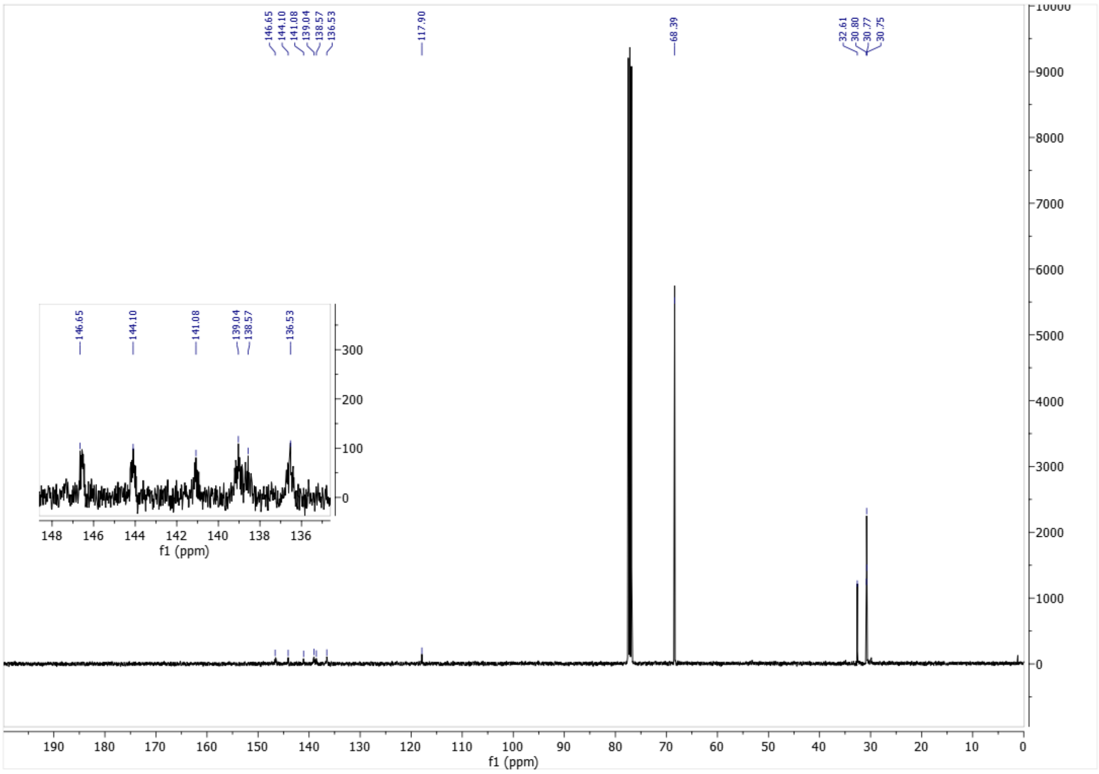

^{13}C-NMR spectrum of (**c3**) (400 MHz, $CDCl_3$)

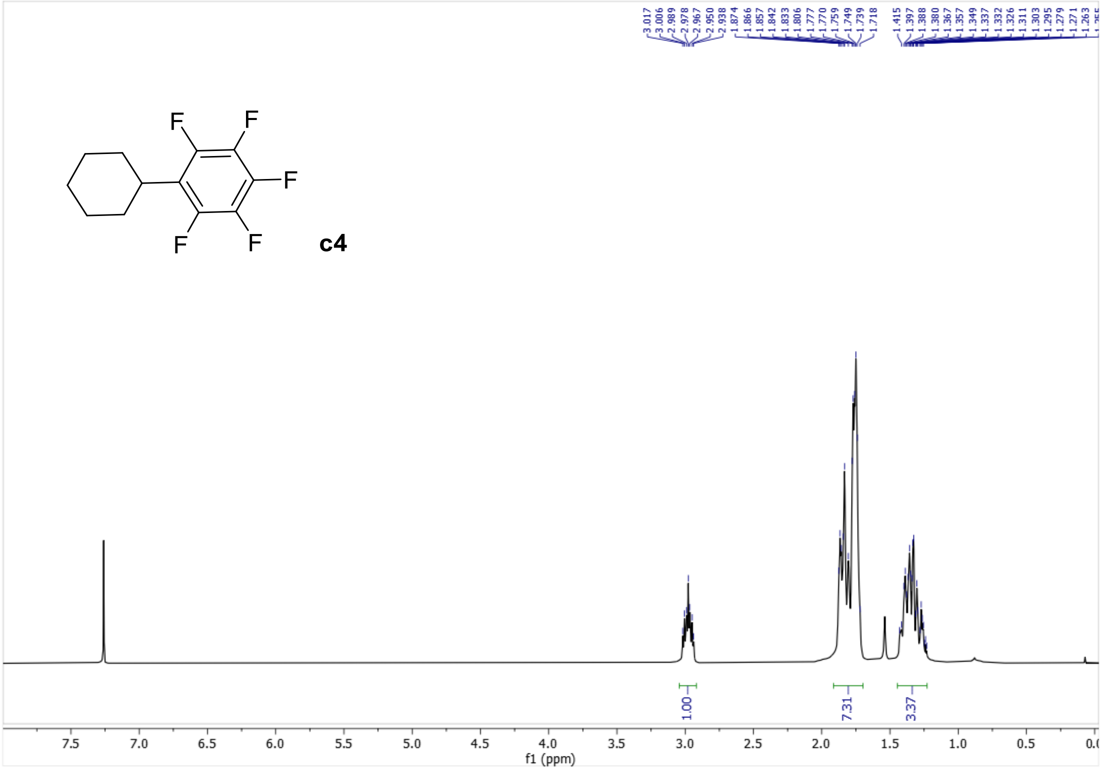

^{1}H-NMR spectrum of (**c4**) (400 MHz, $CDCl_3$)

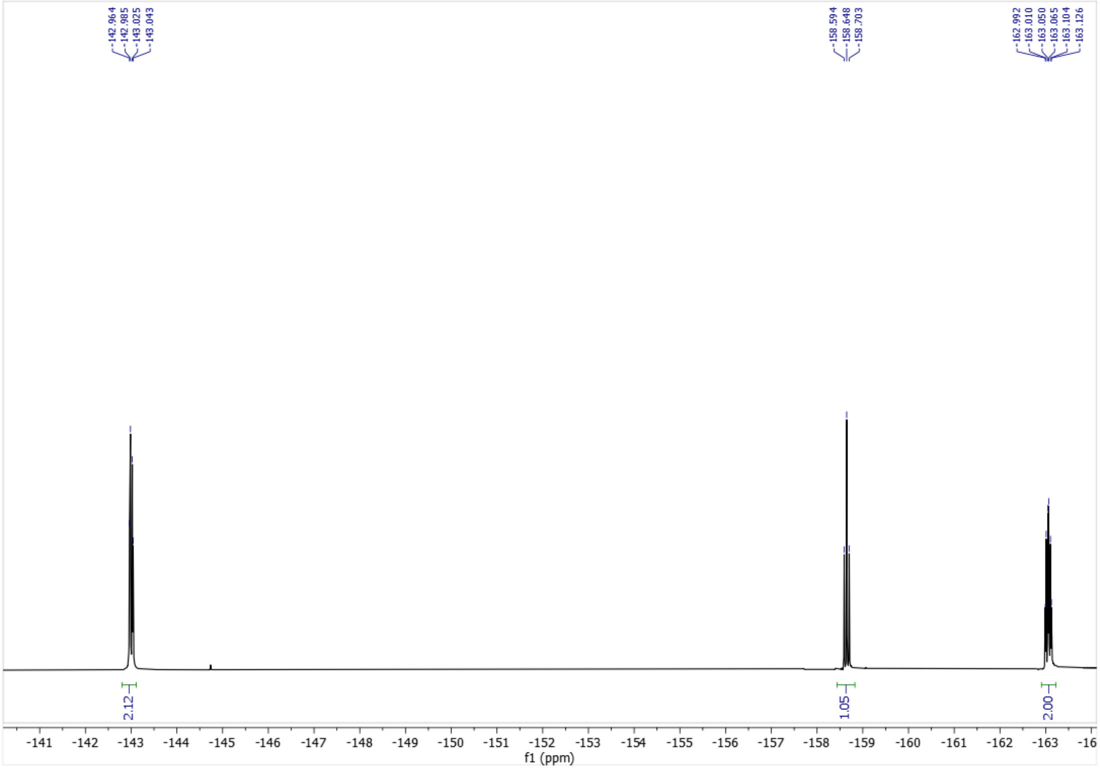

^{19}F-NMR spectrum of (**c4**) (400 MHz, $CDCl_3$)

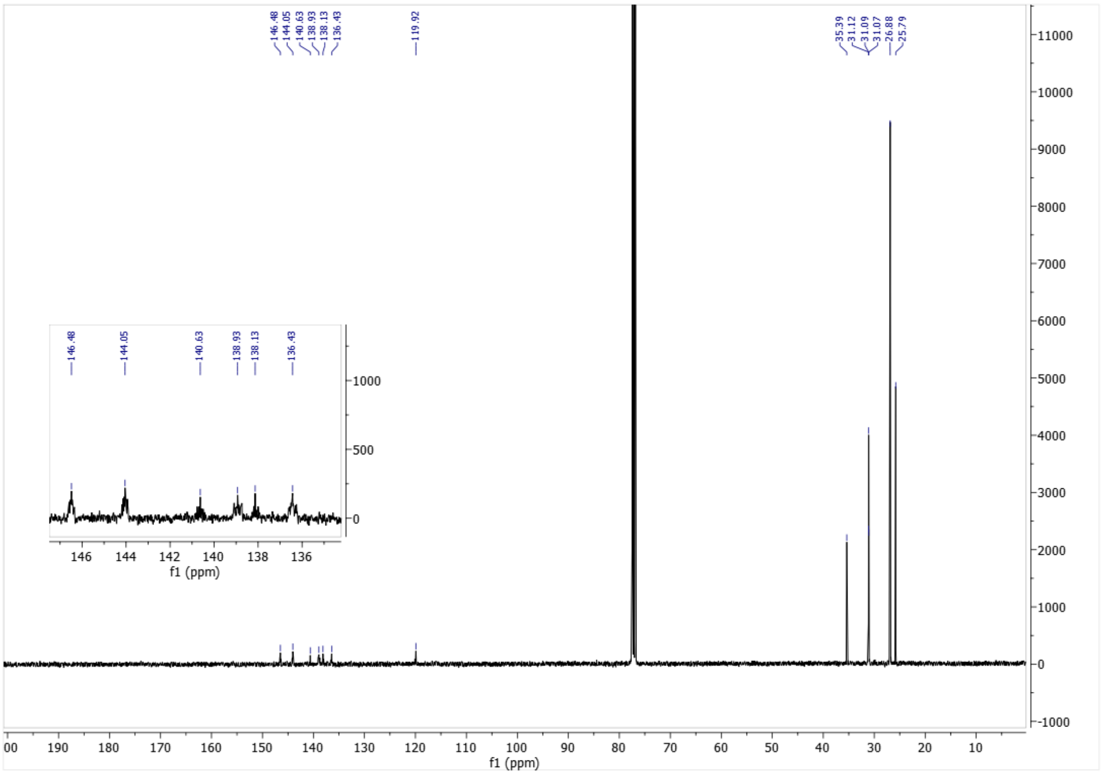

^{13}C-NMR spectrum of (**c4**) (400 MHz, $CDCl_3$)

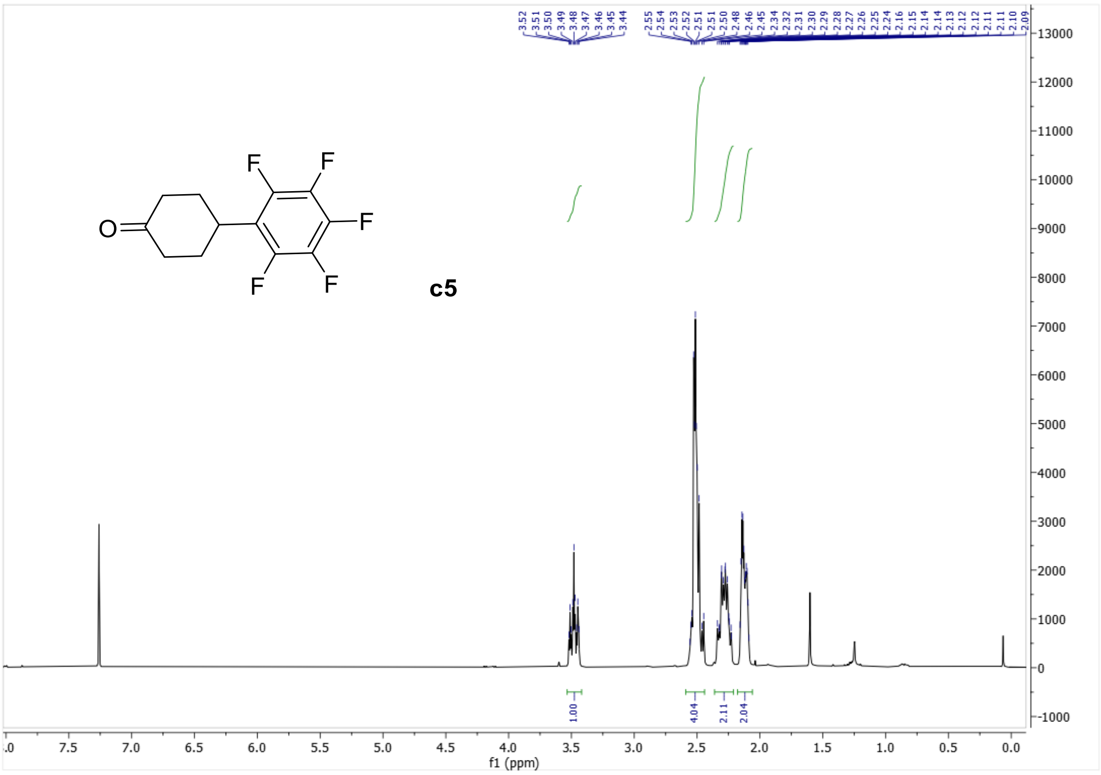

^{1}H-NMR spectrum of (**c5**) (400 MHz, $CDCl_3$)

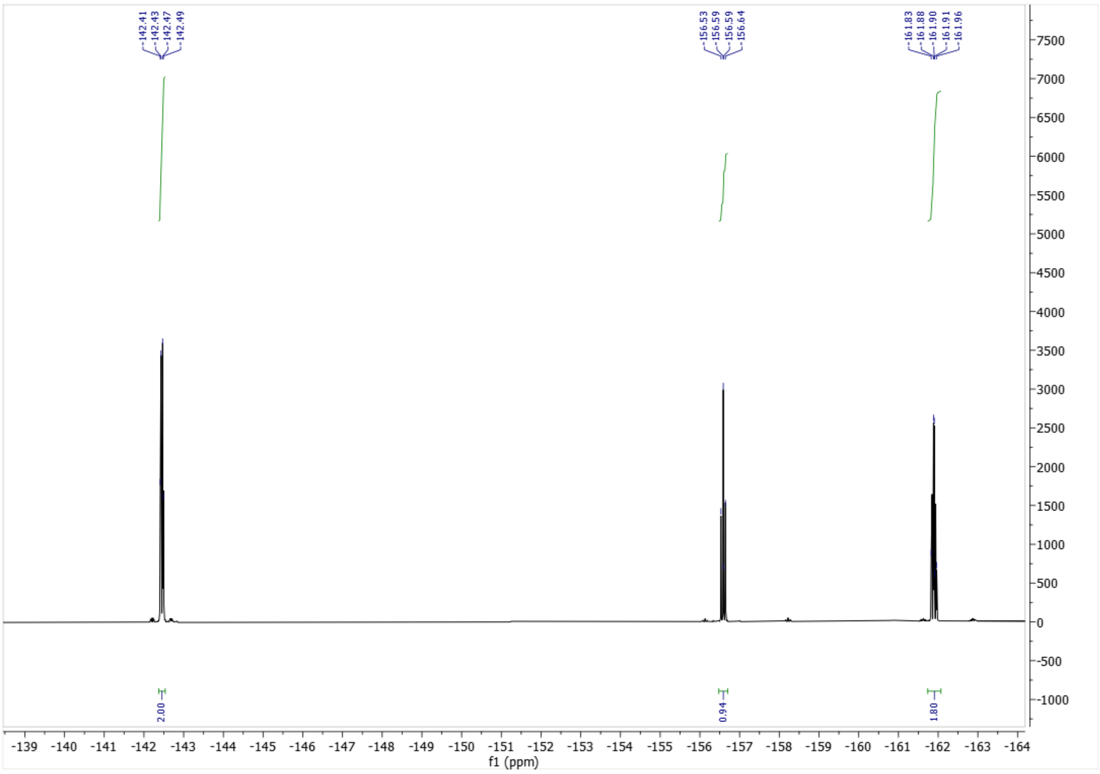

^{19}F-NMR spectrum of (**c5**) (400 MHz, $CDCl_3$)

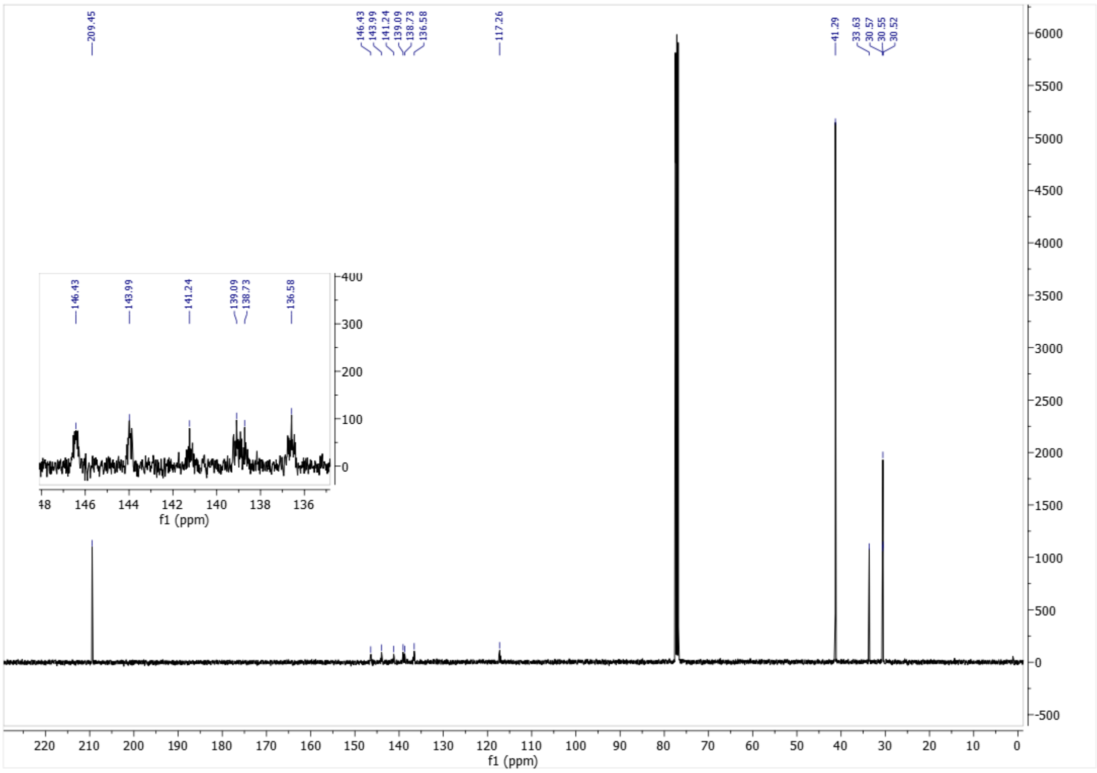

^{13}C-NMR spectrum of (**c5**) (400 MHz, $CDCl_3$)

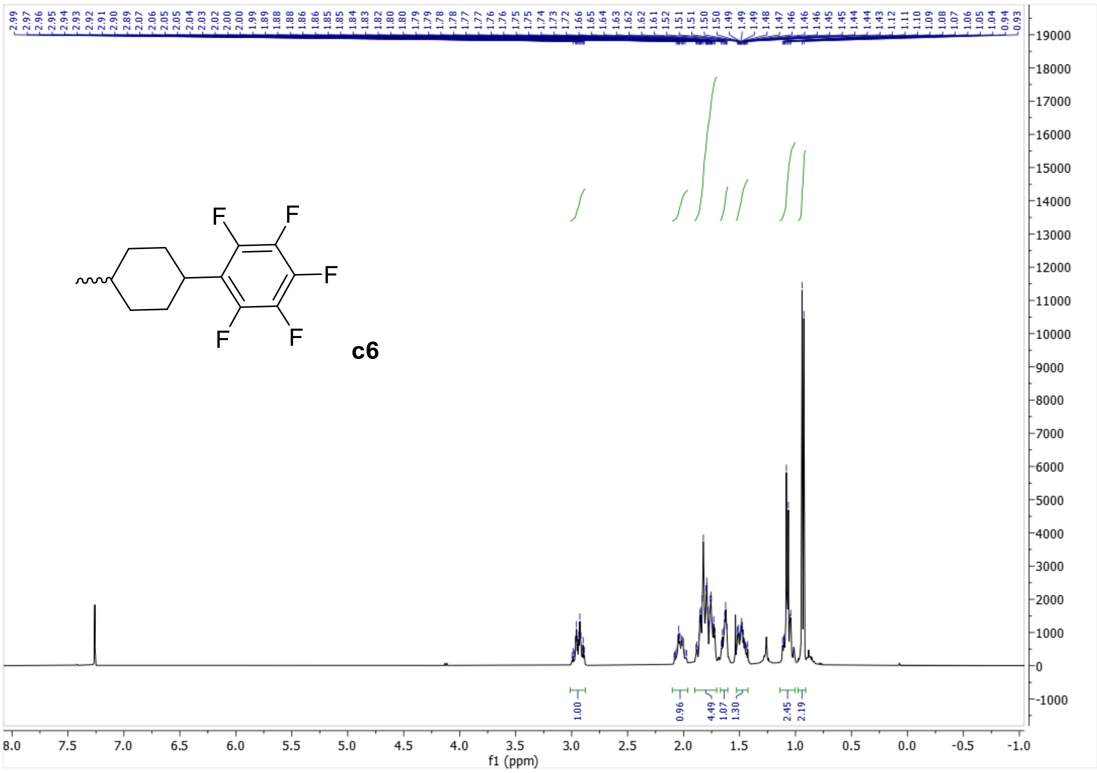

^{1}H-NMR spectrum of (**c6**) (400 MHz, $CDCl_3$)

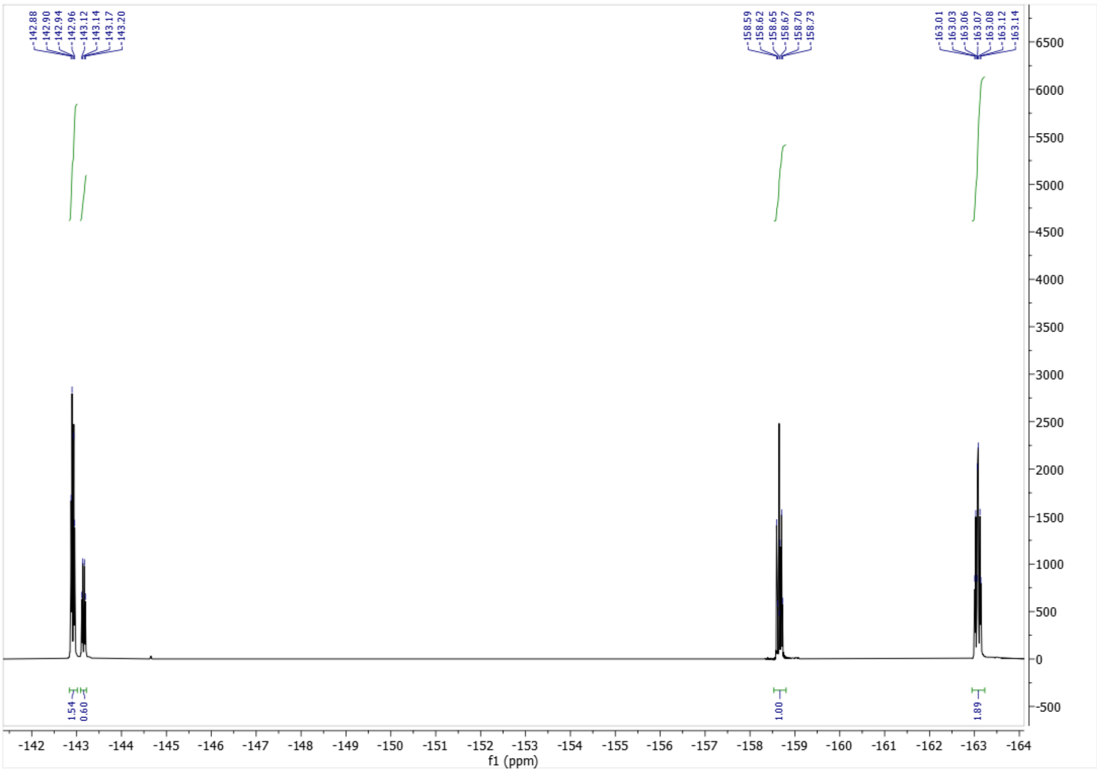

^{19}F-NMR spectrum of (**c6**) (400 MHz, $CDCl_3$)

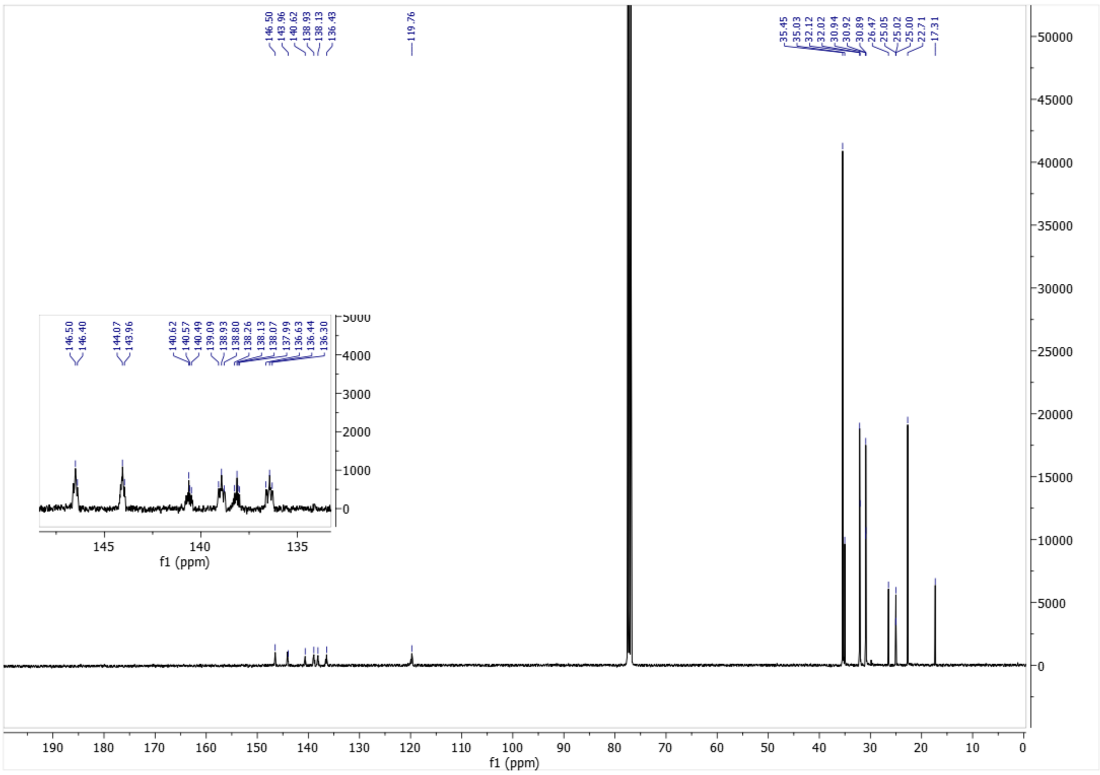

^{13}C-NMR spectrum of (**c6**) (400 MHz, $CDCl_3$)

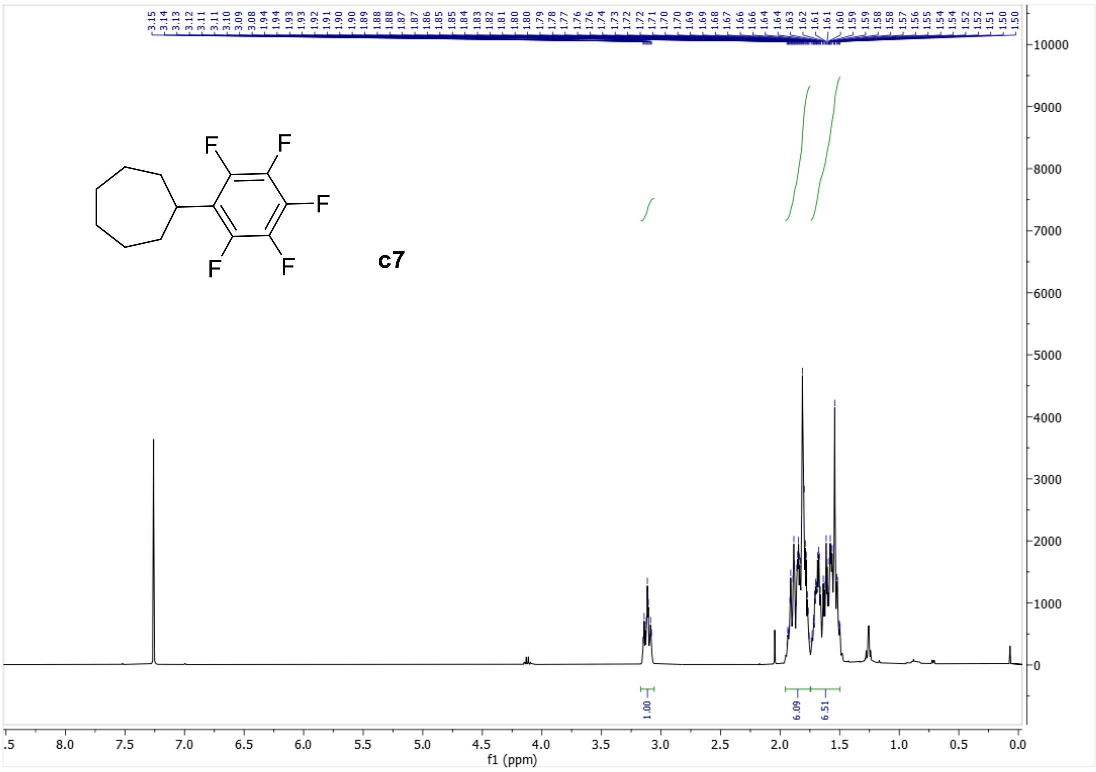

^{1}H-NMR spectrum of (**c7**) (400 MHz, $CDCl_3$)

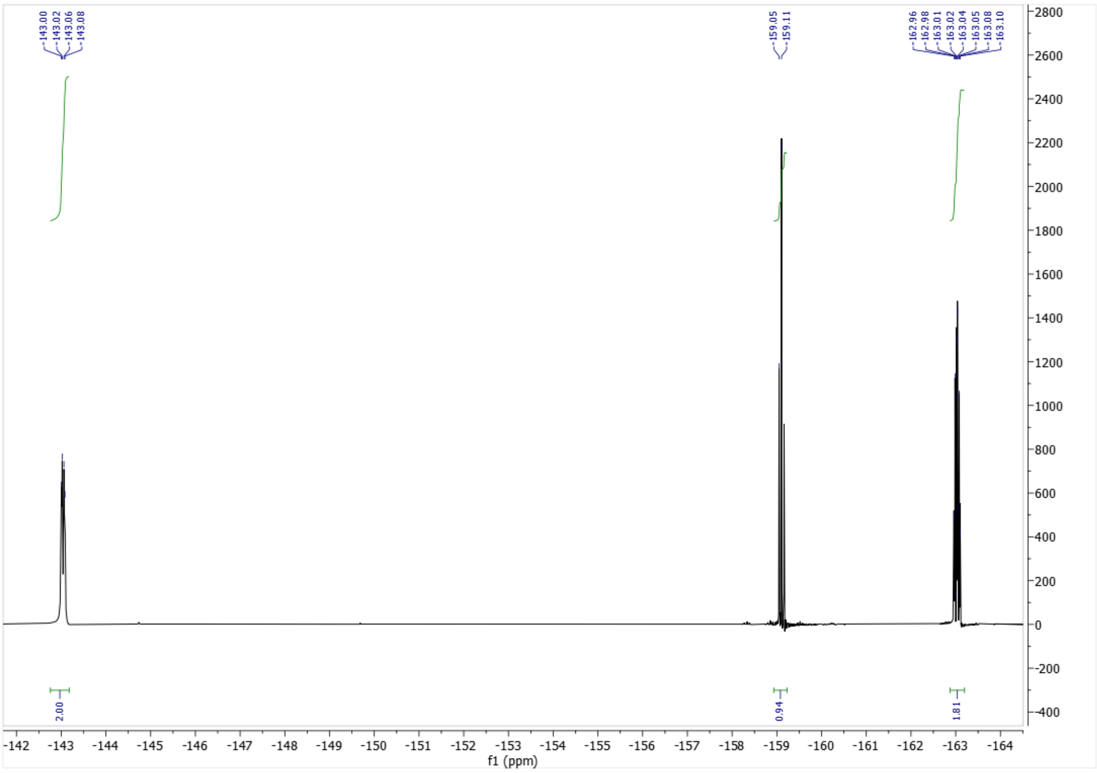

^{19}F-NMR spectrum of (**c7**) (400 MHz, $CDCl_3$)

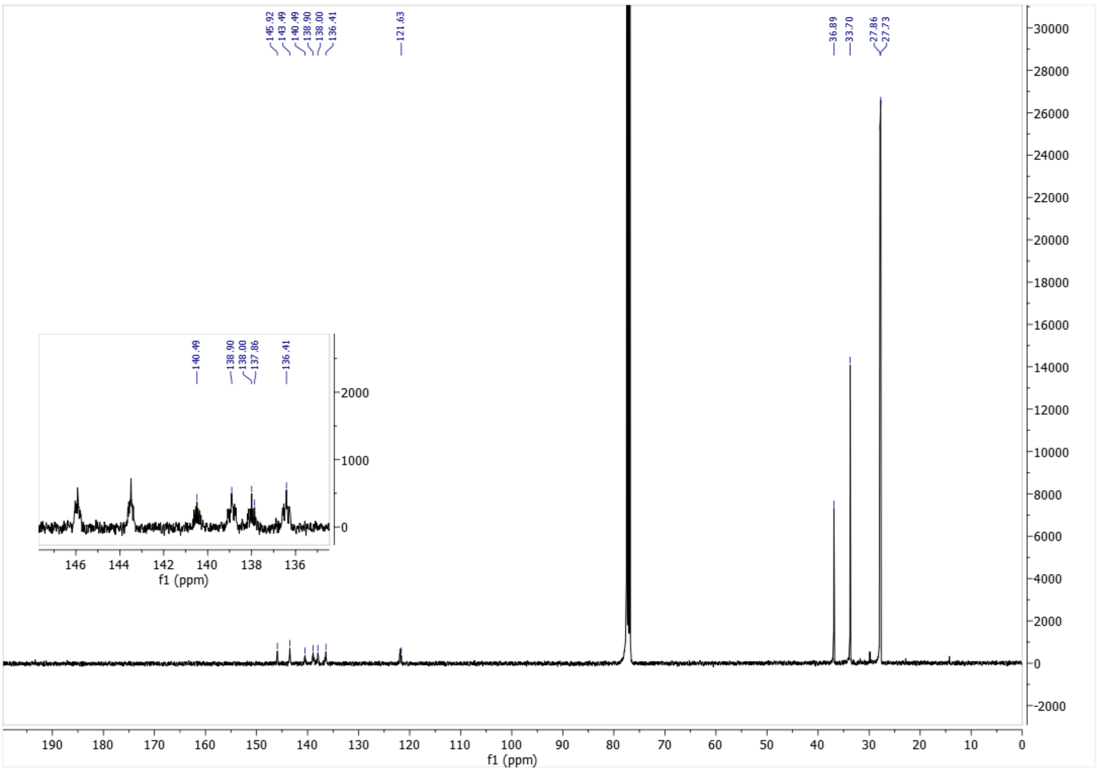

^{13}C-NMR spectrum of (**c7**) (400 MHz, $CDCl_3$)

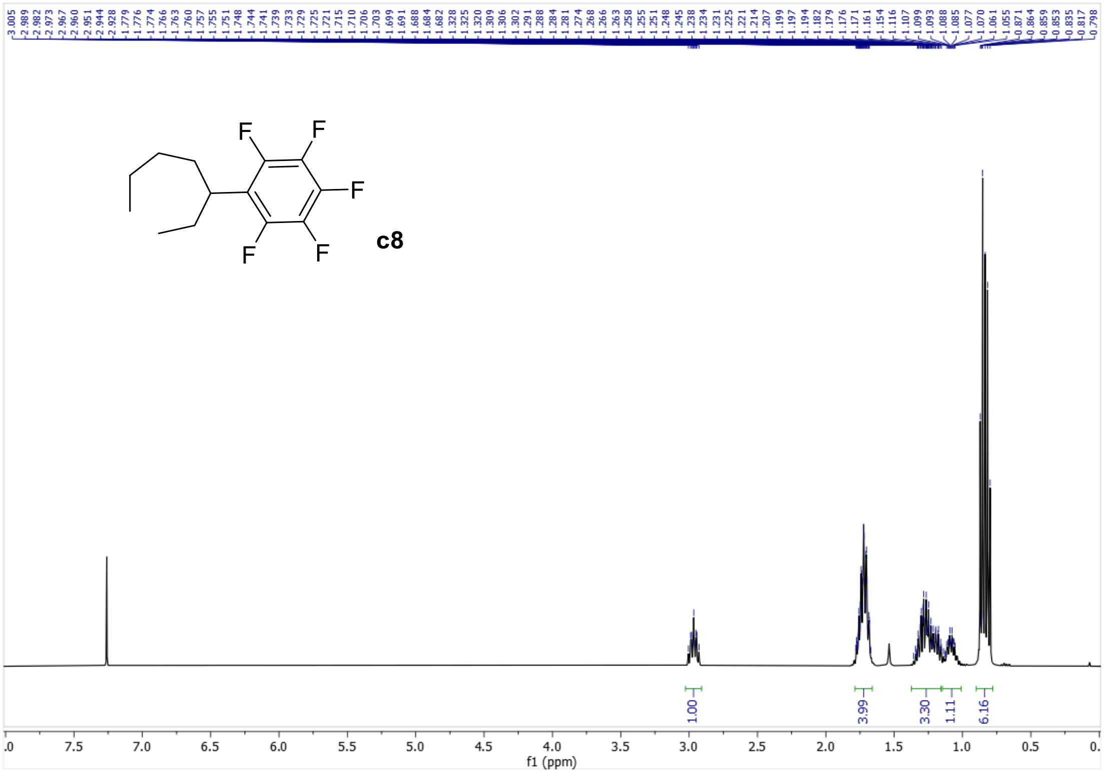

^{1}H-NMR spectrum of (**c8**) (400 MHz, $CDCl_3$)

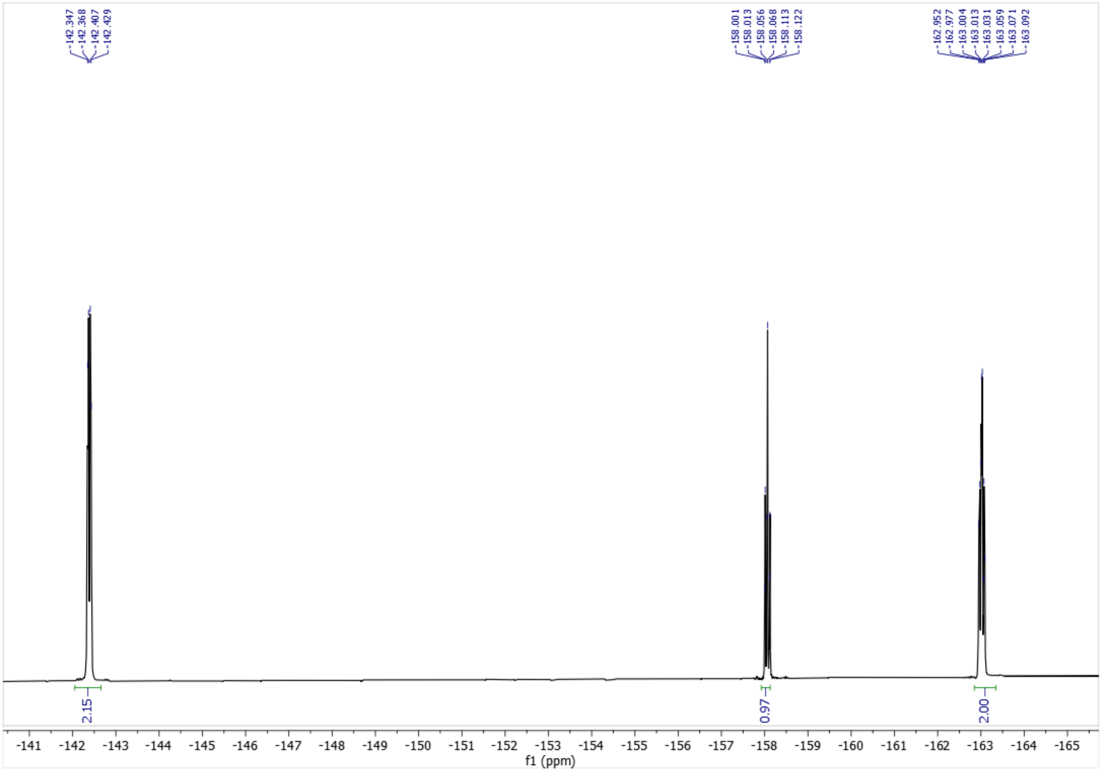

^{19}F-NMR spectrum of (**c8**) (400 MHz, $CDCl_3$)

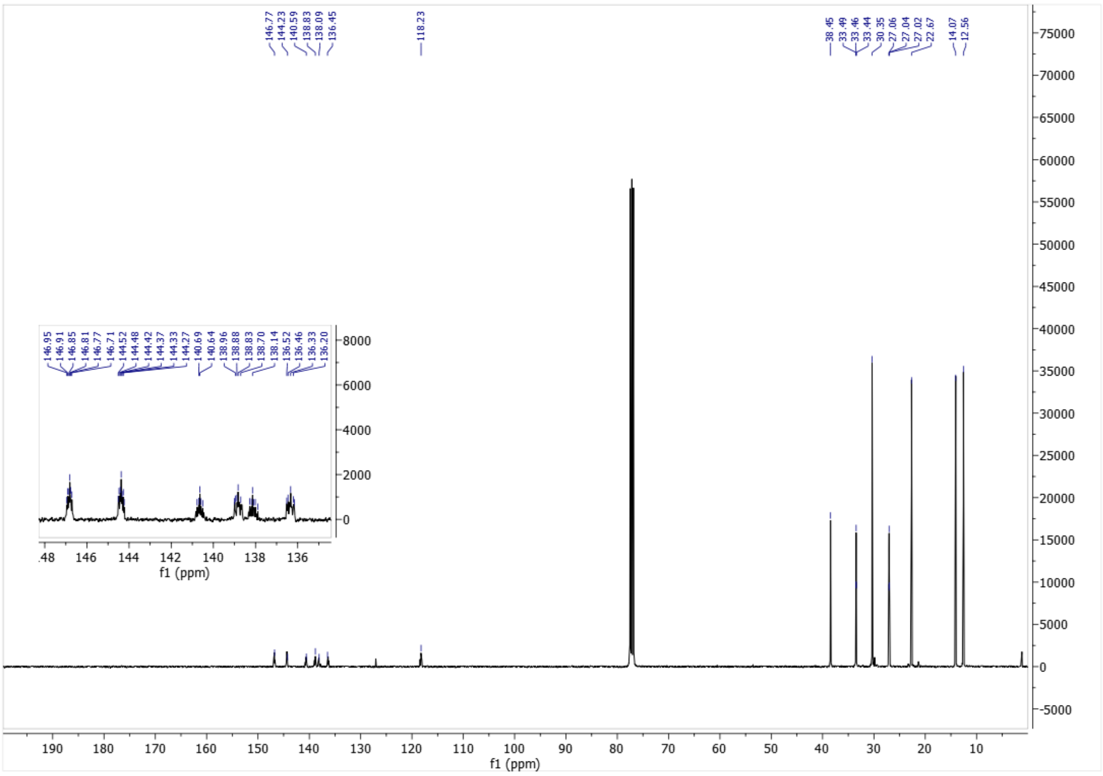

^{13}C-NMR spectrum of (**c8**) (400 MHz, $CDCl_3$)

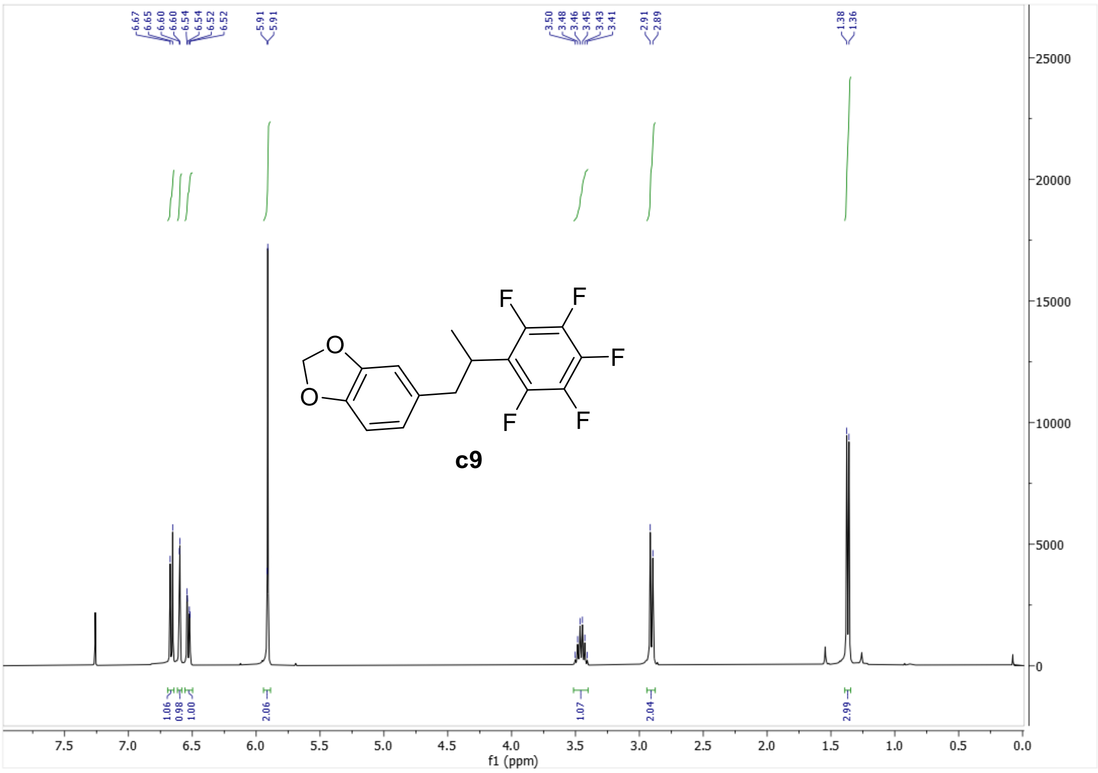

^{1}H-NMR spectrum of (**c9**) (400 MHz, $CDCl_3$)

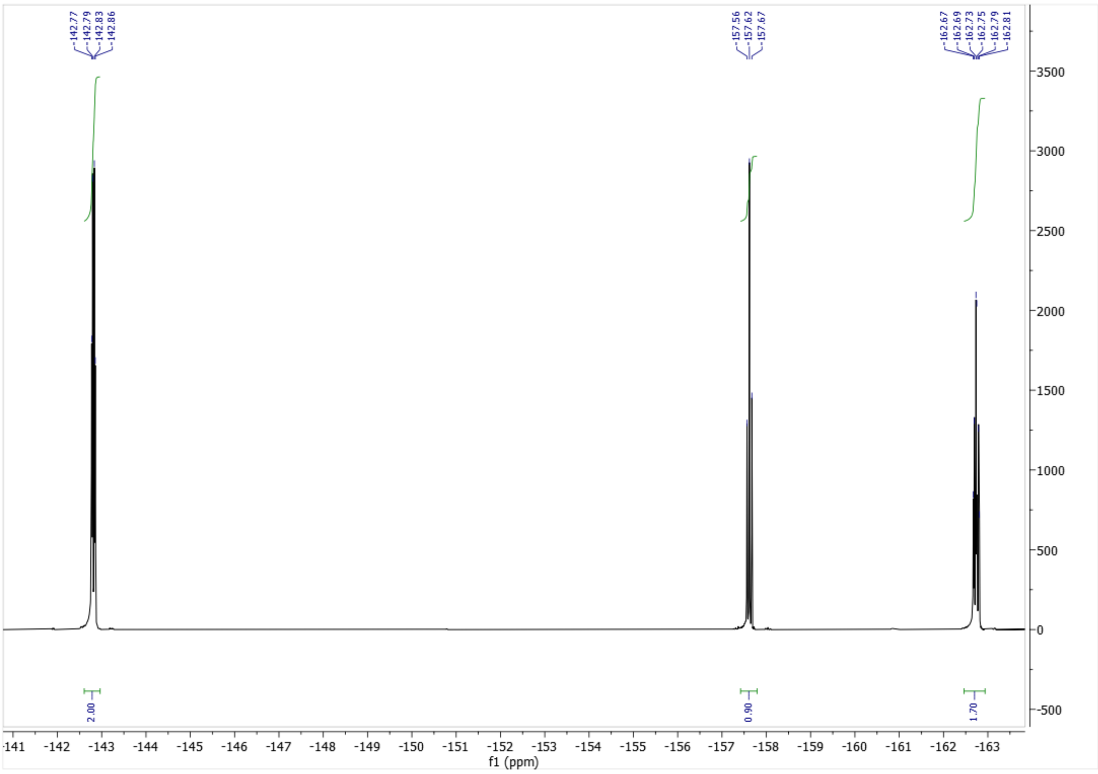

^{19}F-NMR spectrum of (**c9**) (400 MHz, $CDCl_3$)

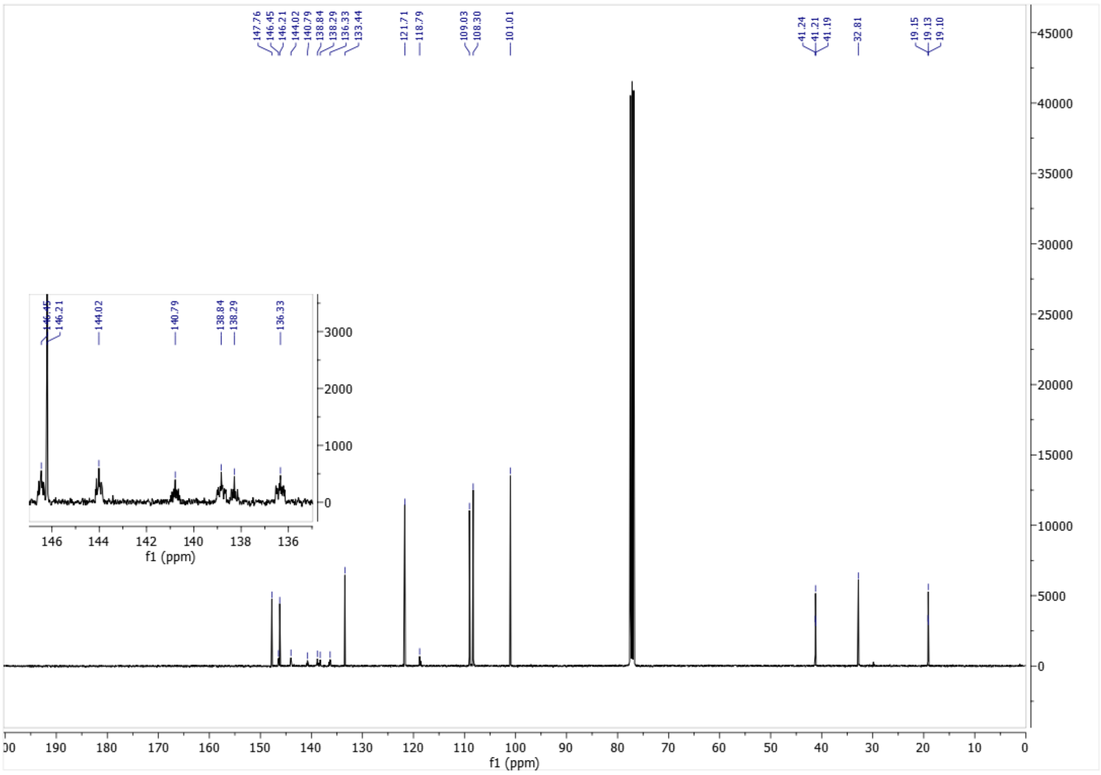

^{13}C-NMR spectrum of (**c9**) (400 MHz, $CDCl_3$)

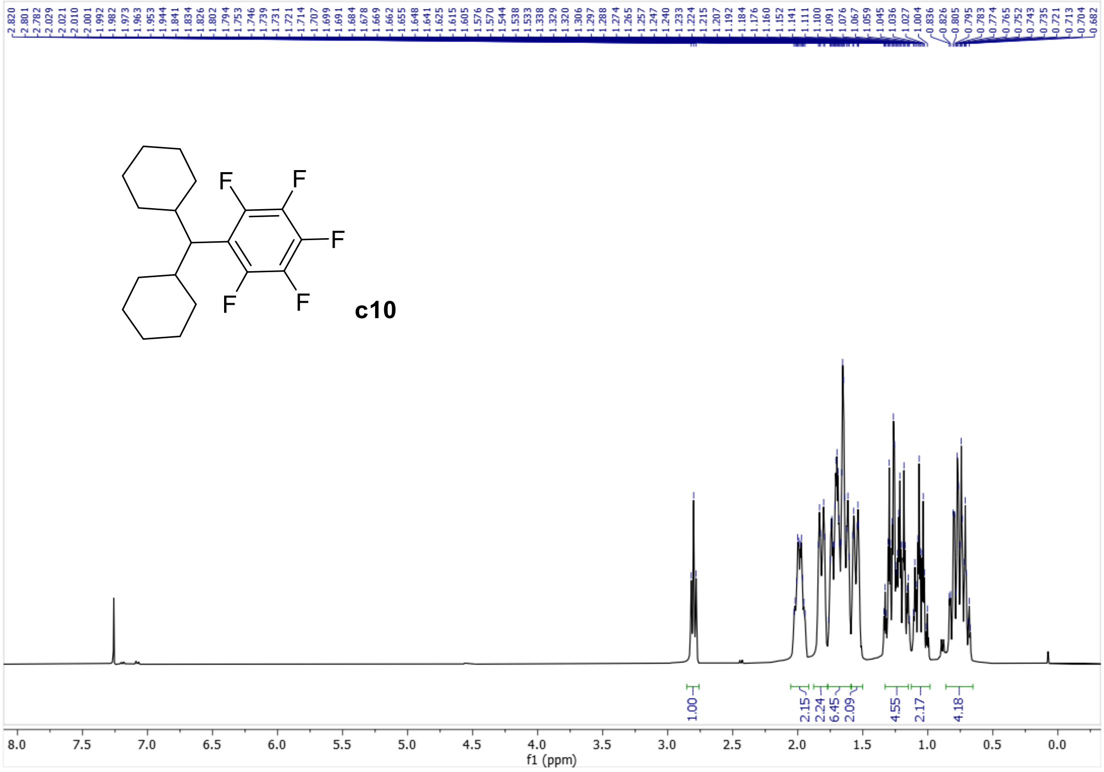

^{1}H-NMR spectrum of (**c10**) (400 MHz, $CDCl_3$)

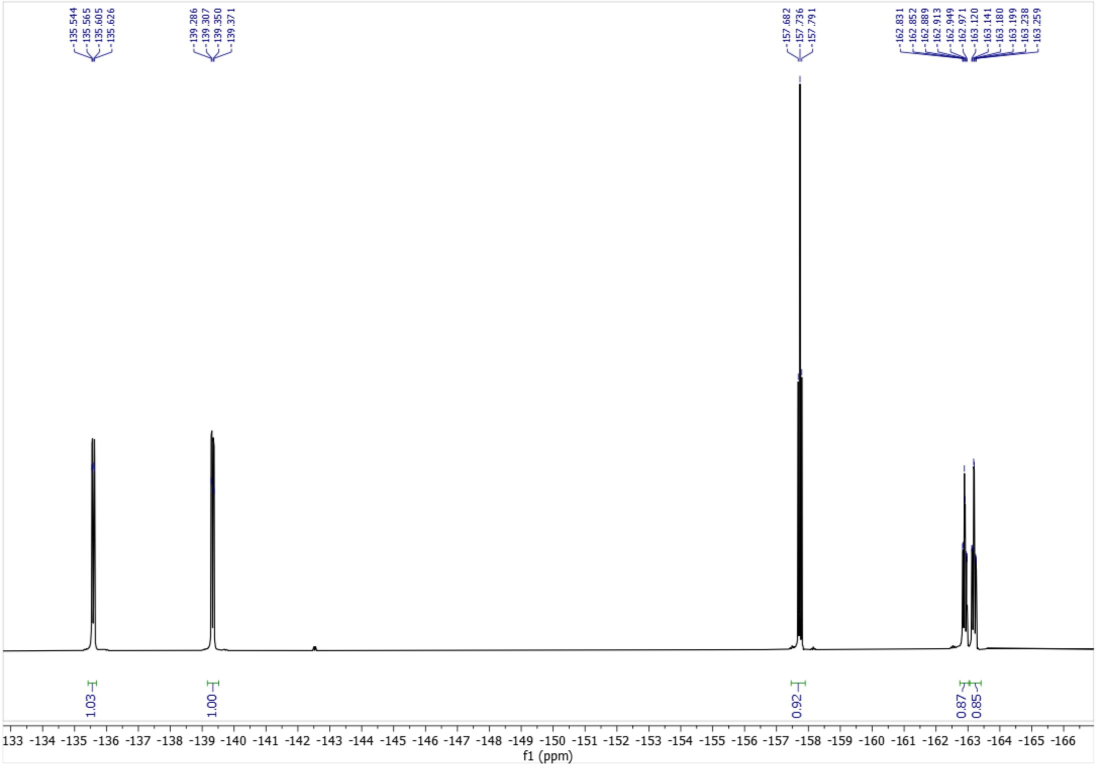

^{19}F-NMR spectrum of (**c10**) (400 MHz, $CDCl_3$)

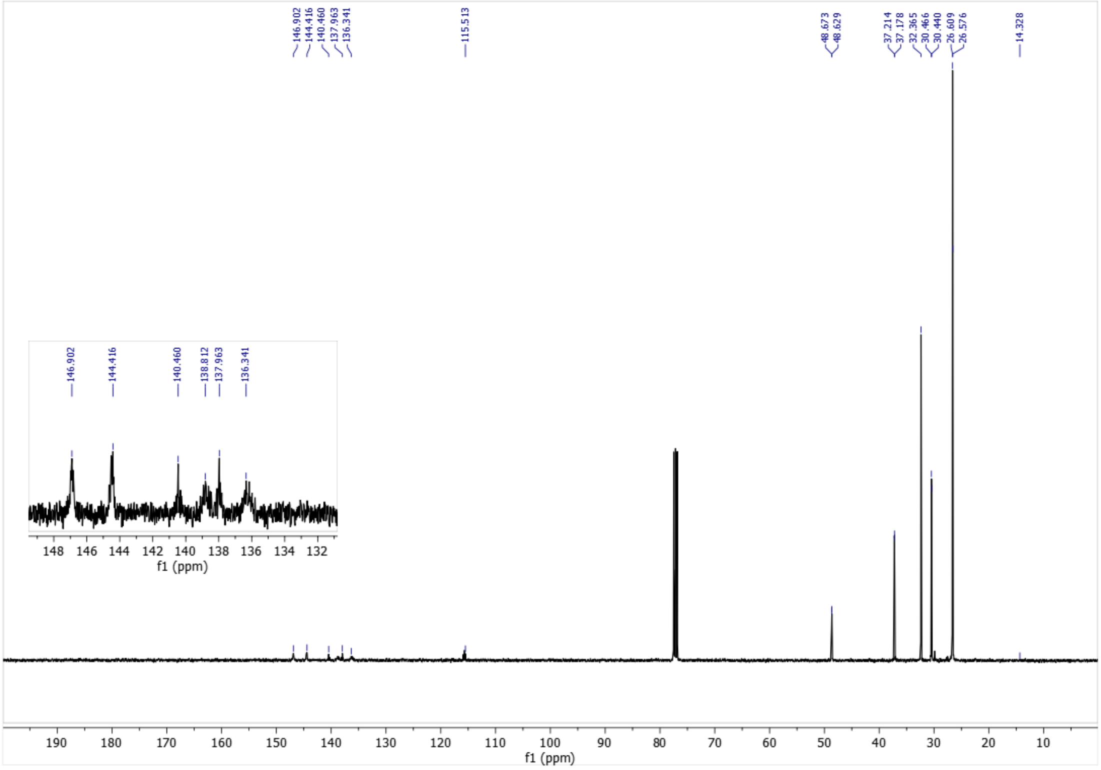

^{13}C-NMR spectrum of (**c10**) (400 MHz, $CDCl_3$)

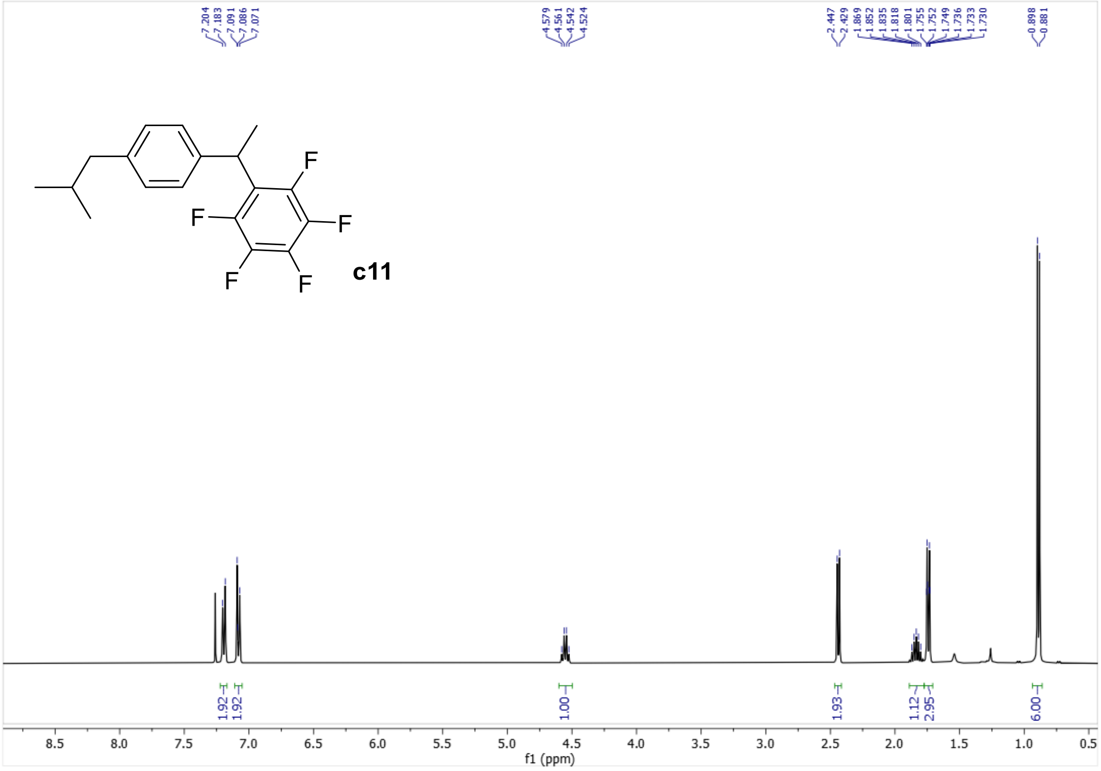

^{1}H-NMR spectrum of (**c11**) (400 MHz, $CDCl_3$)

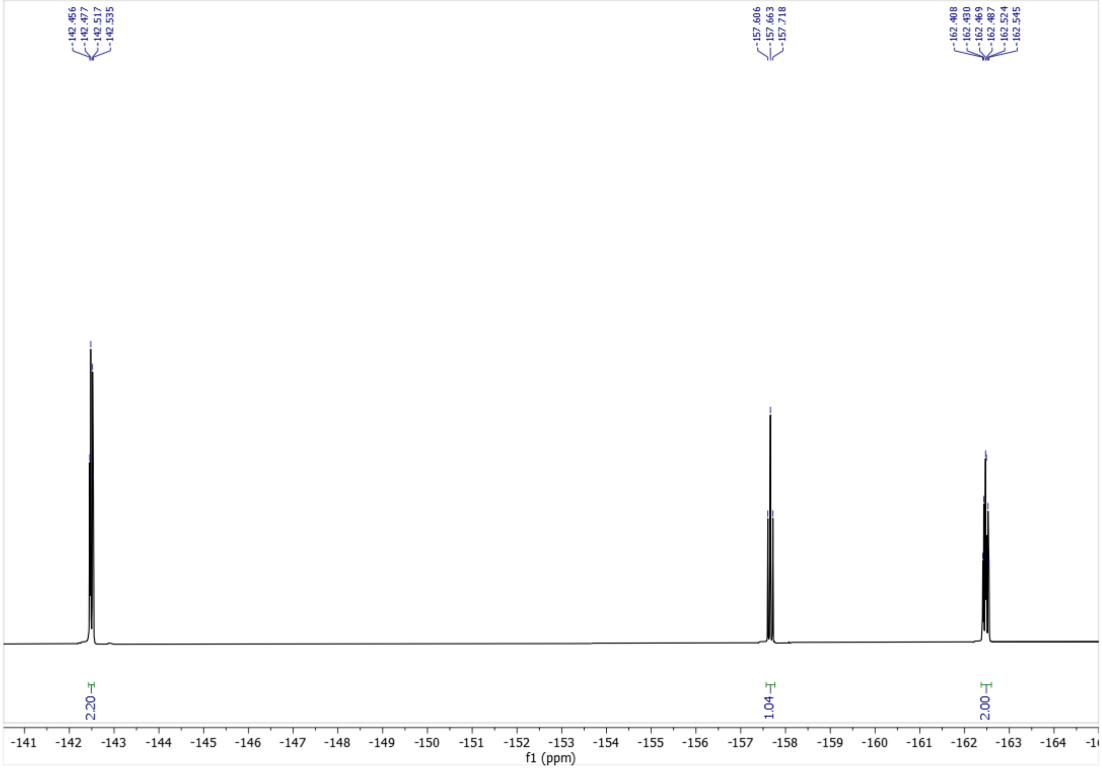

^{19}F-NMR spectrum of (**c11**) (400 MHz, $CDCl_3$)

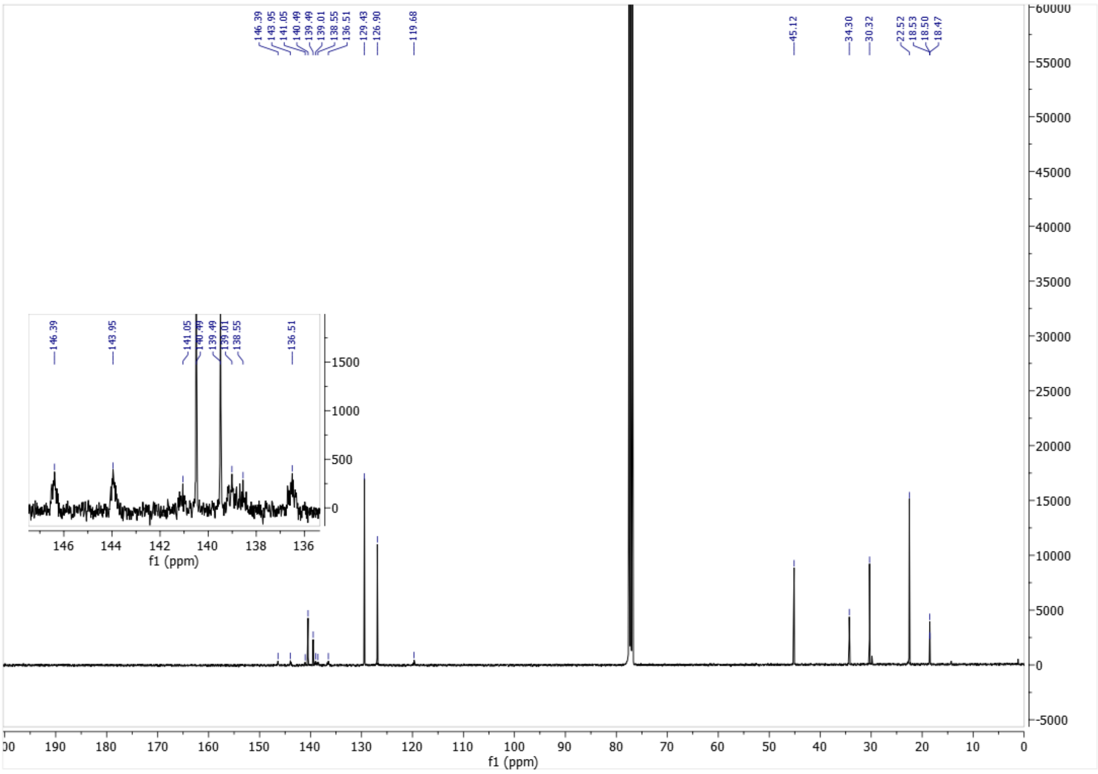

^{13}C-NMR spectrum of (**c11**) (400 MHz, $CDCl_3$)

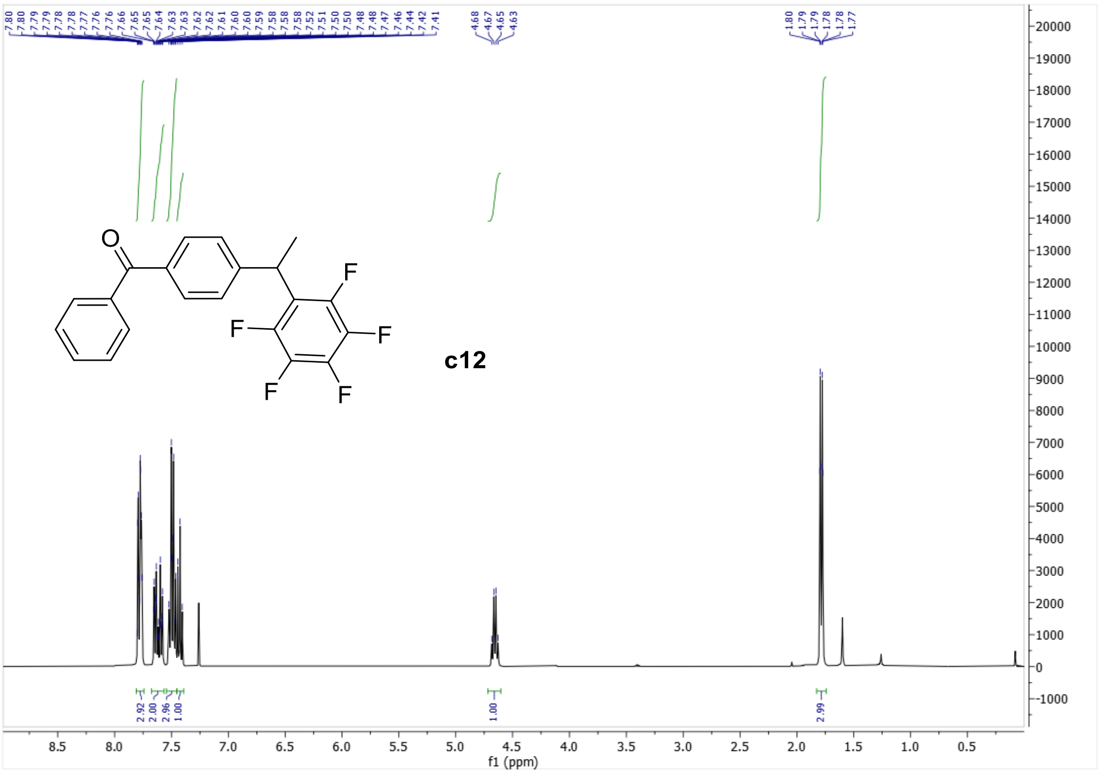

^{1}H-NMR spectrum of (**c12**) (400 MHz, $CDCl_3$)

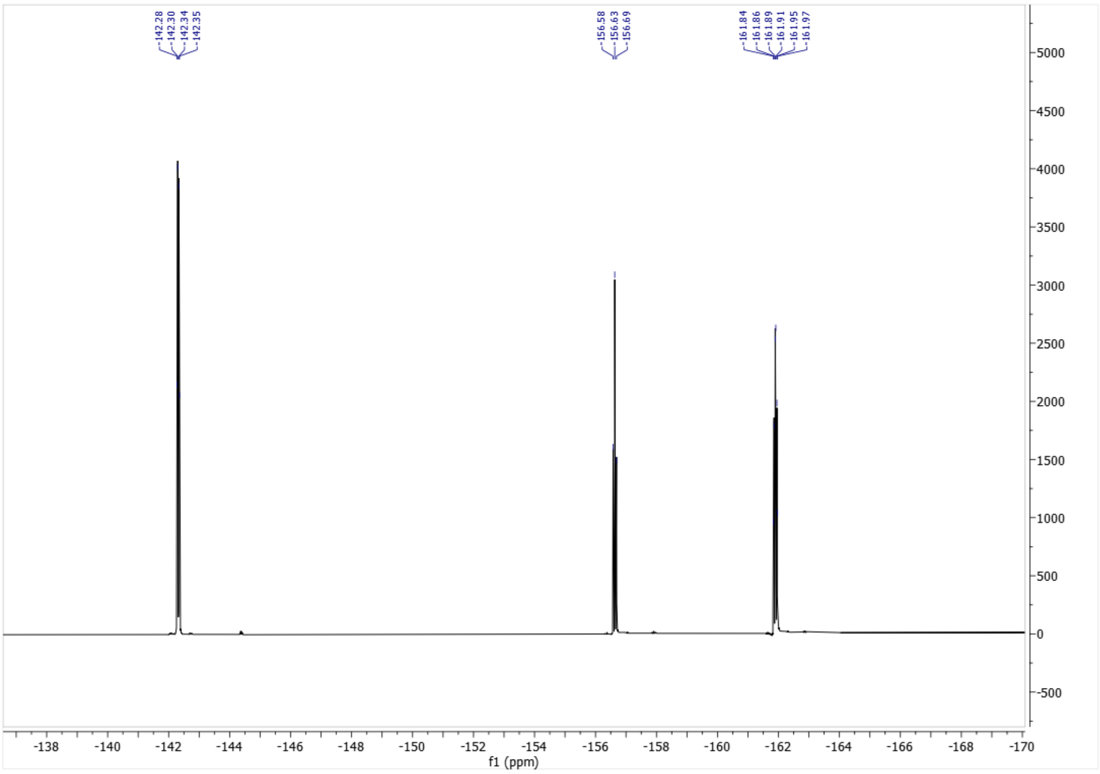

^{19}F-NMR spectrum of (**c12**) (400 MHz, $CDCl_3$)

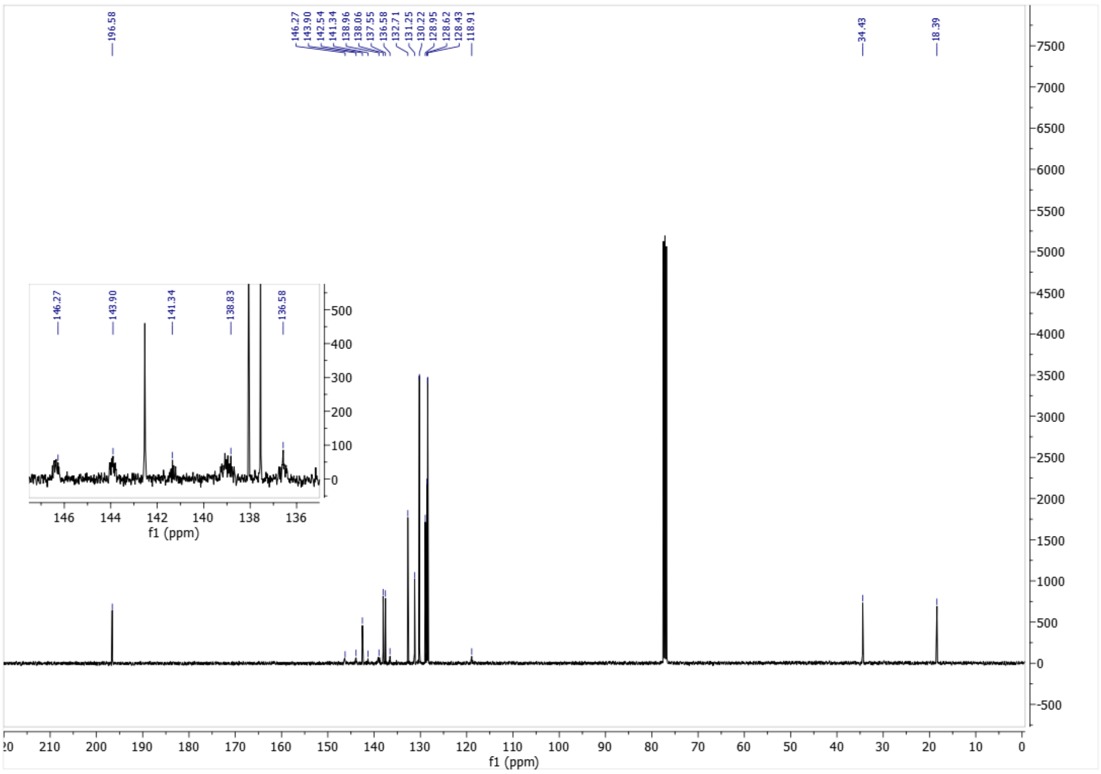

^{13}C-NMR spectrum of (**c12**) (400 MHz, $CDCl_3$)

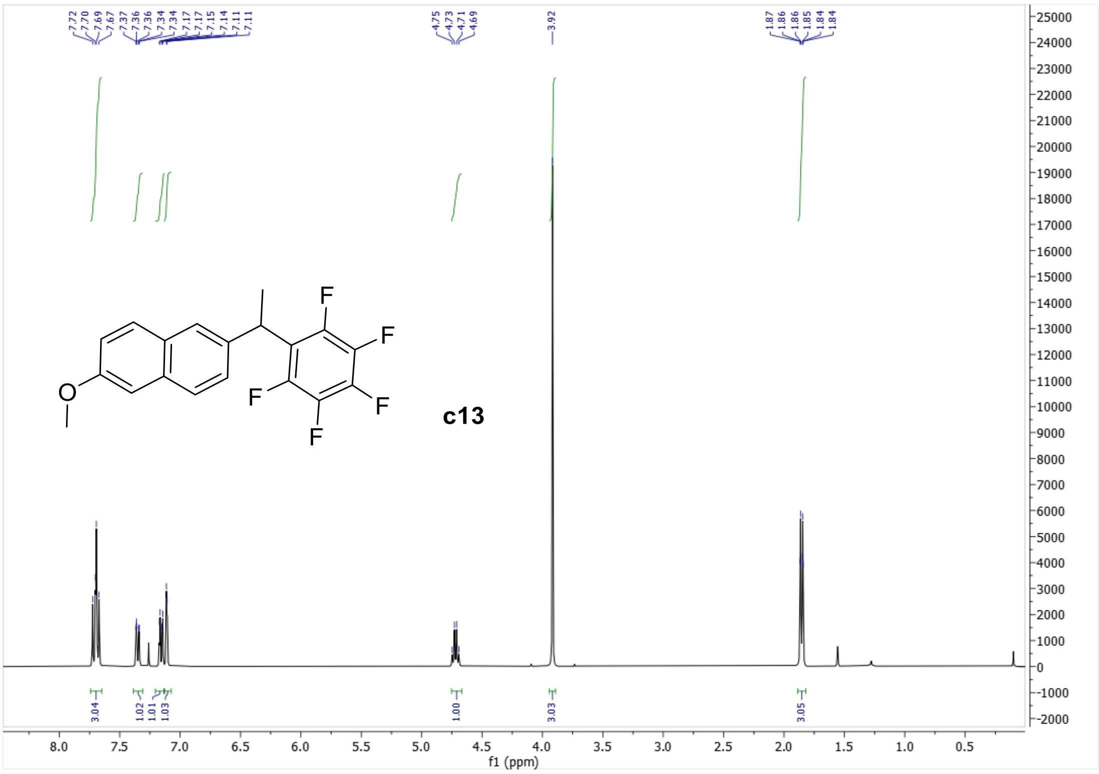

^{1}H-NMR spectrum of (**c13**) (400 MHz, $CDCl_3$)

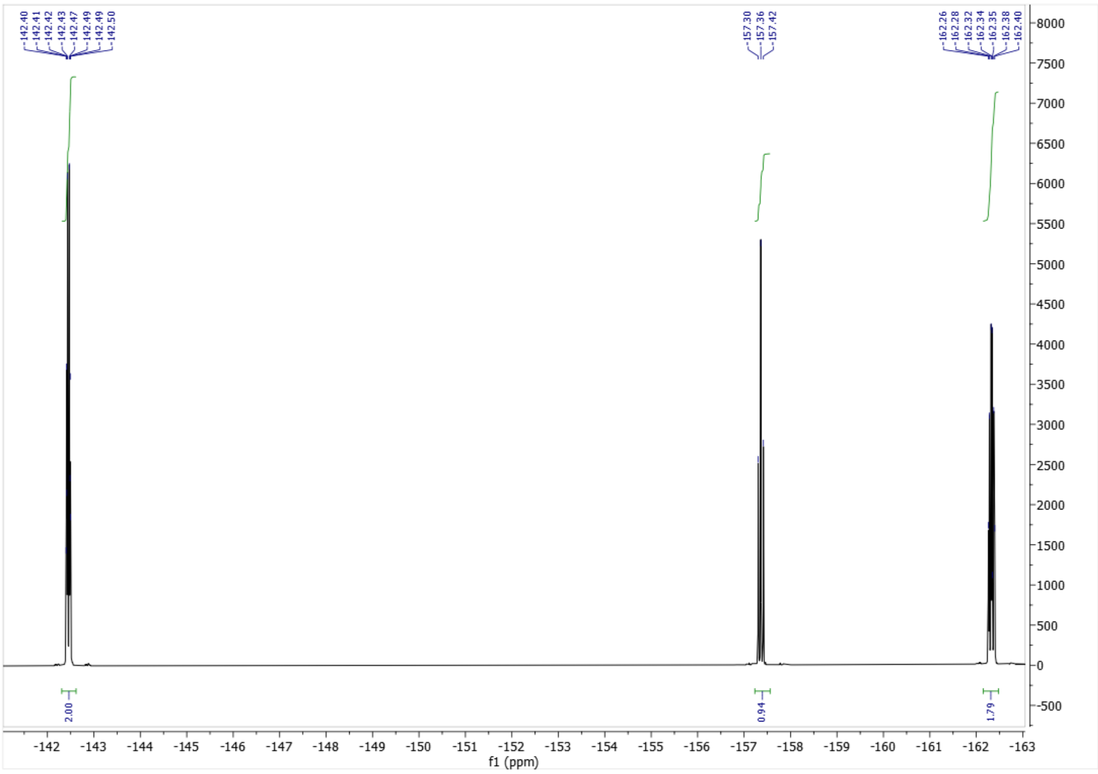

^{19}F-NMR spectrum of (**c13**) (400 MHz, $CDCl_3$)

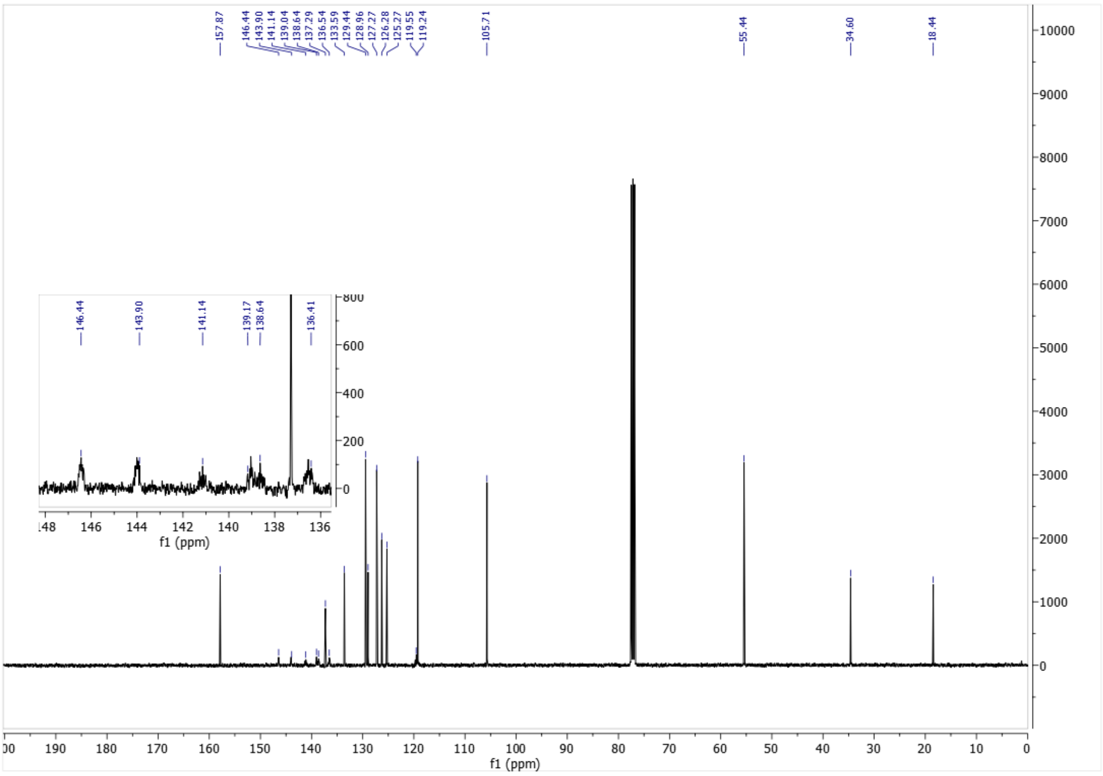

^{13}C-NMR spectrum of (**c13**) (400 MHz, $CDCl_3$)

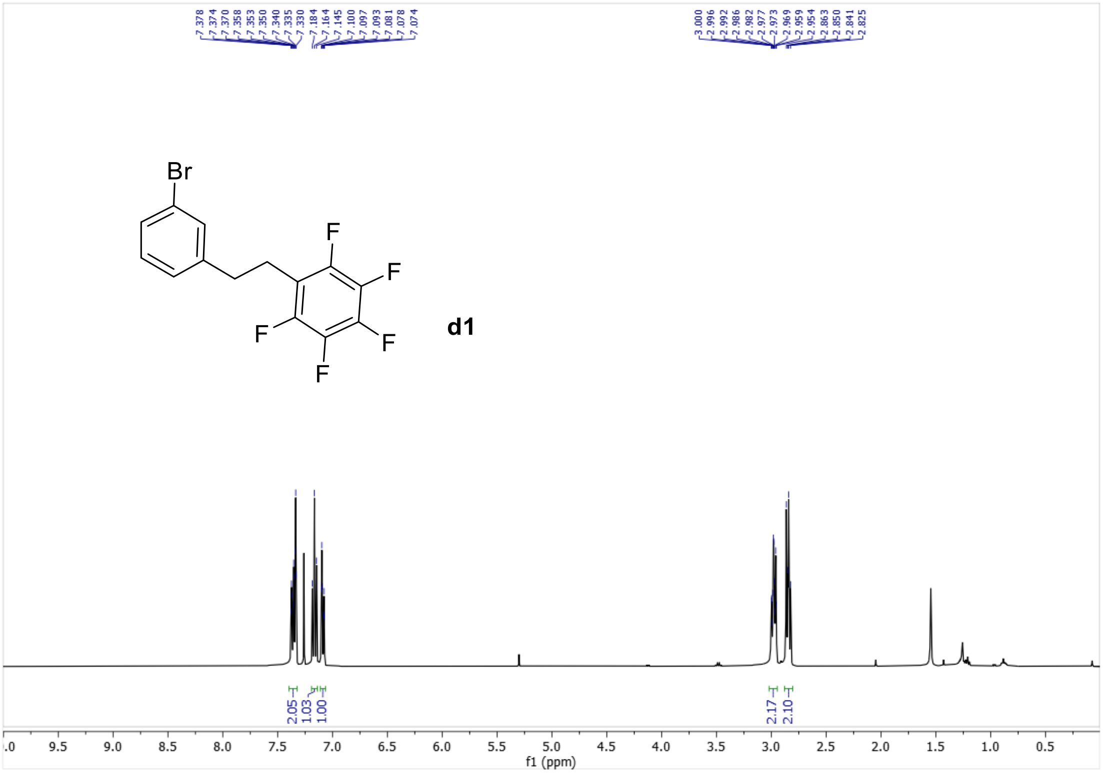

^{1}H-NMR spectrum of (**d1**) (400 MHz, $CDCl_3$)

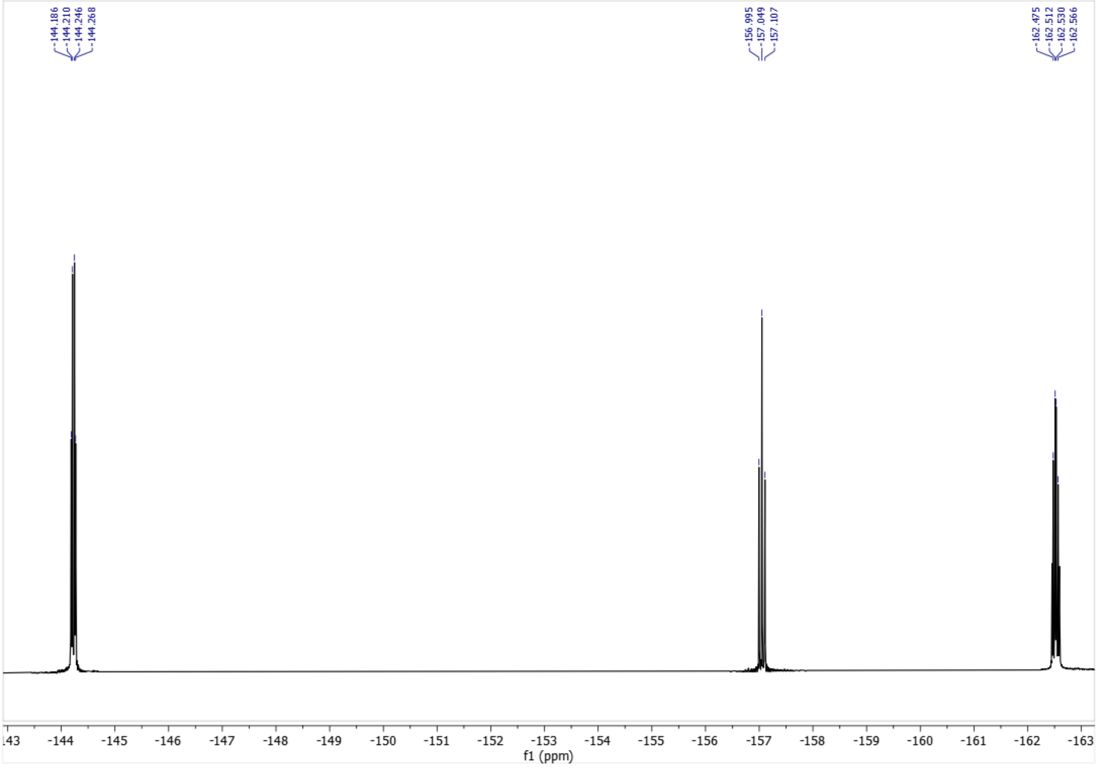

^{19}F-NMR spectrum of (**d1**) (400 MHz, $CDCl_3$)

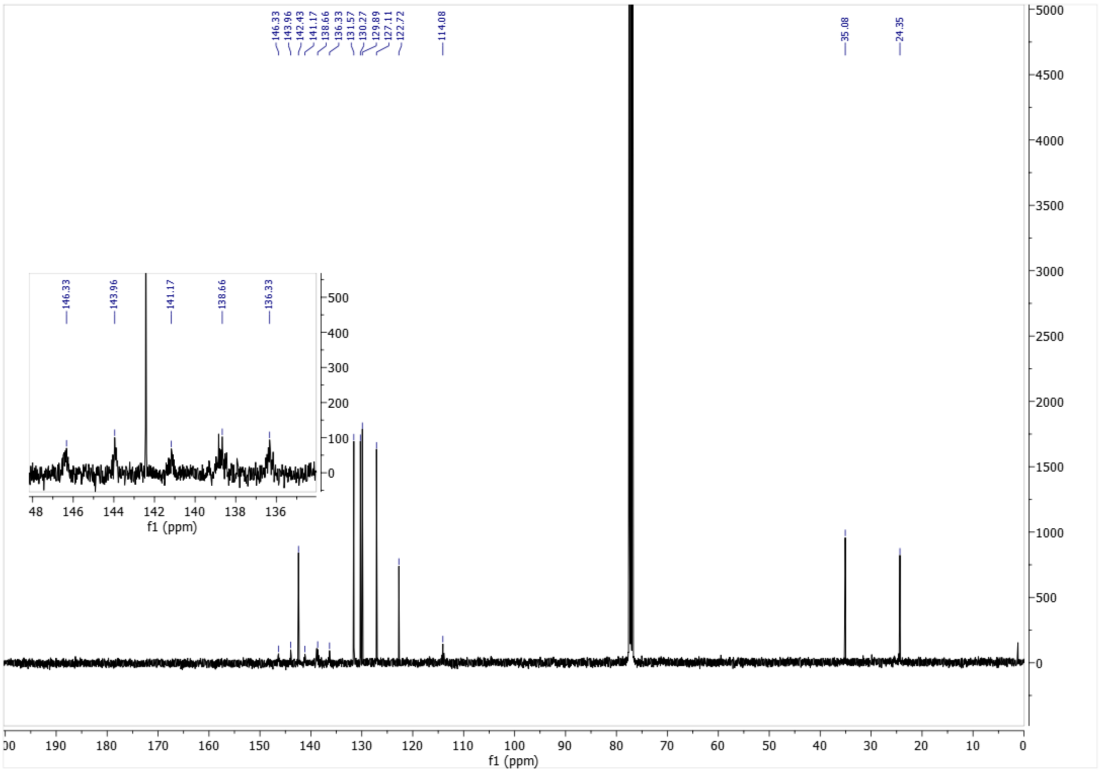

^{13}C-NMR spectrum of (**d1**) (400 MHz, $CDCl_3$)

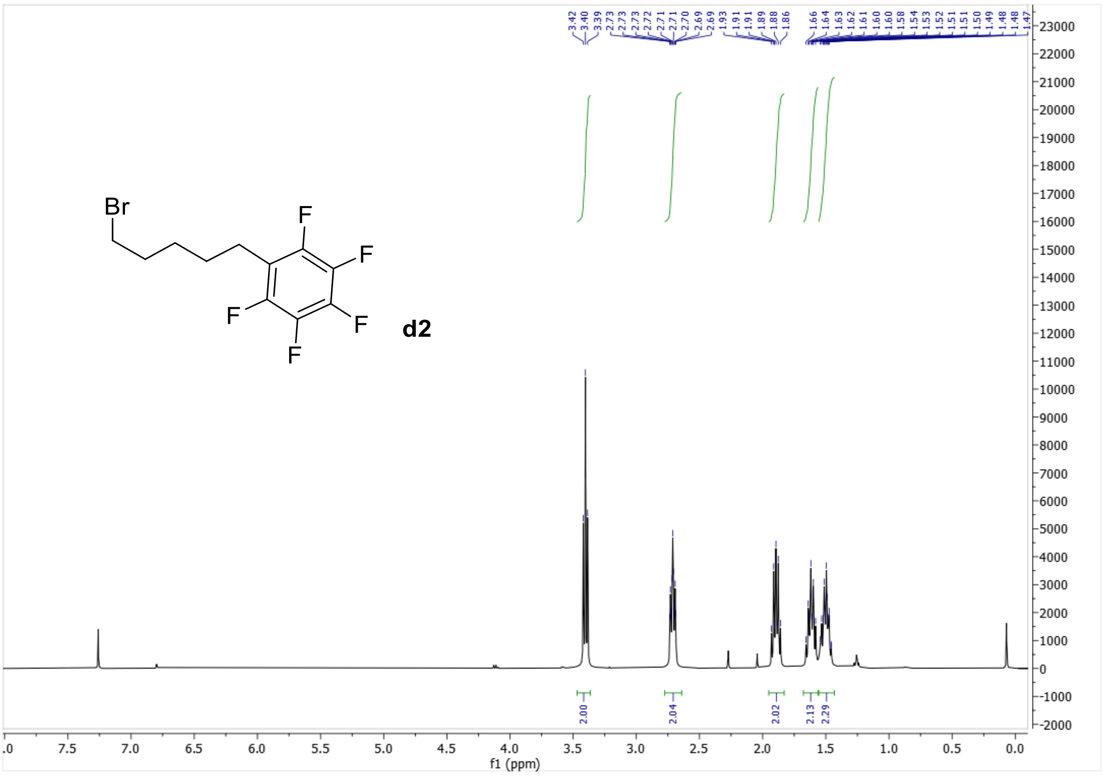

^{1}H-NMR spectrum of (**d2**) (400 MHz, $CDCl_3$)

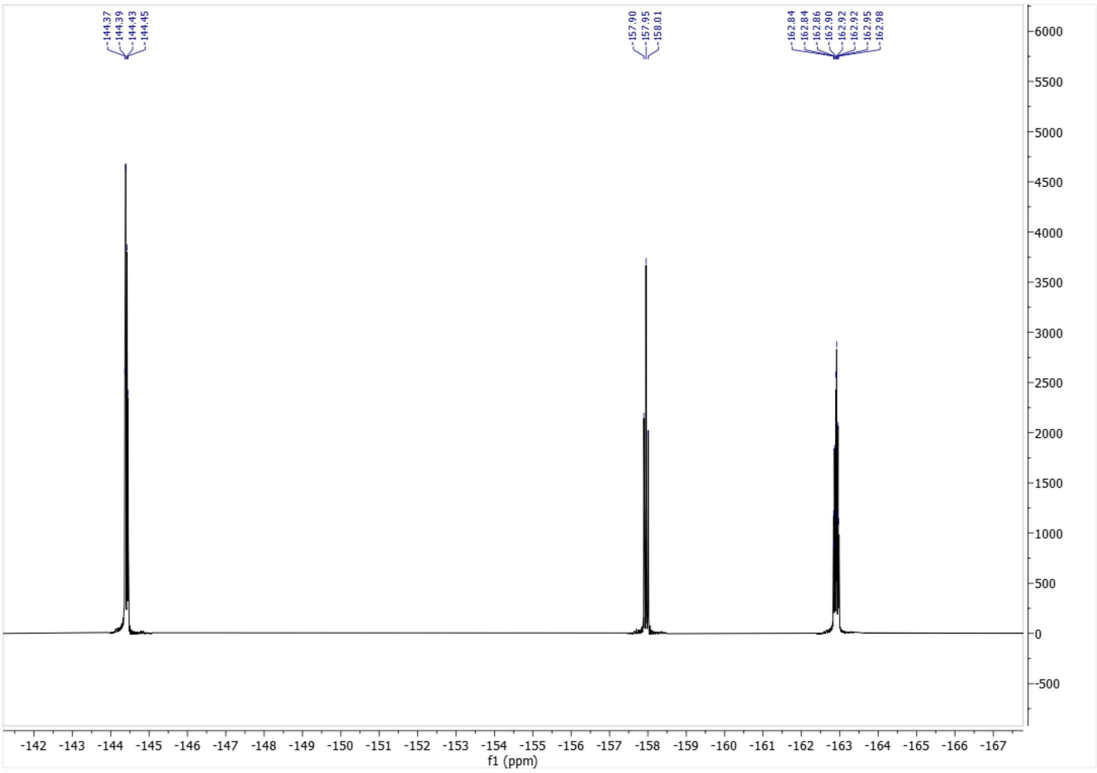

^{19}F-NMR spectrum of (**d2**) (400 MHz, $CDCl_3$)

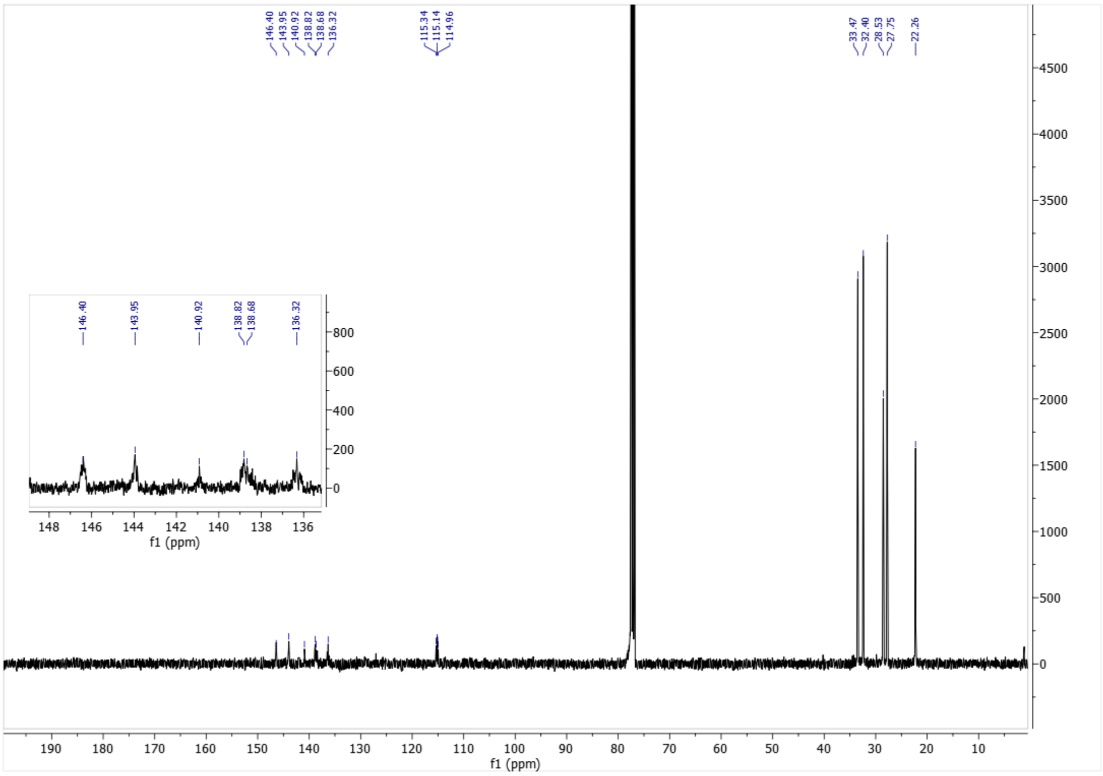

^{13}C-NMR spectrum of (**d2**) (400 MHz, $CDCl_3$)

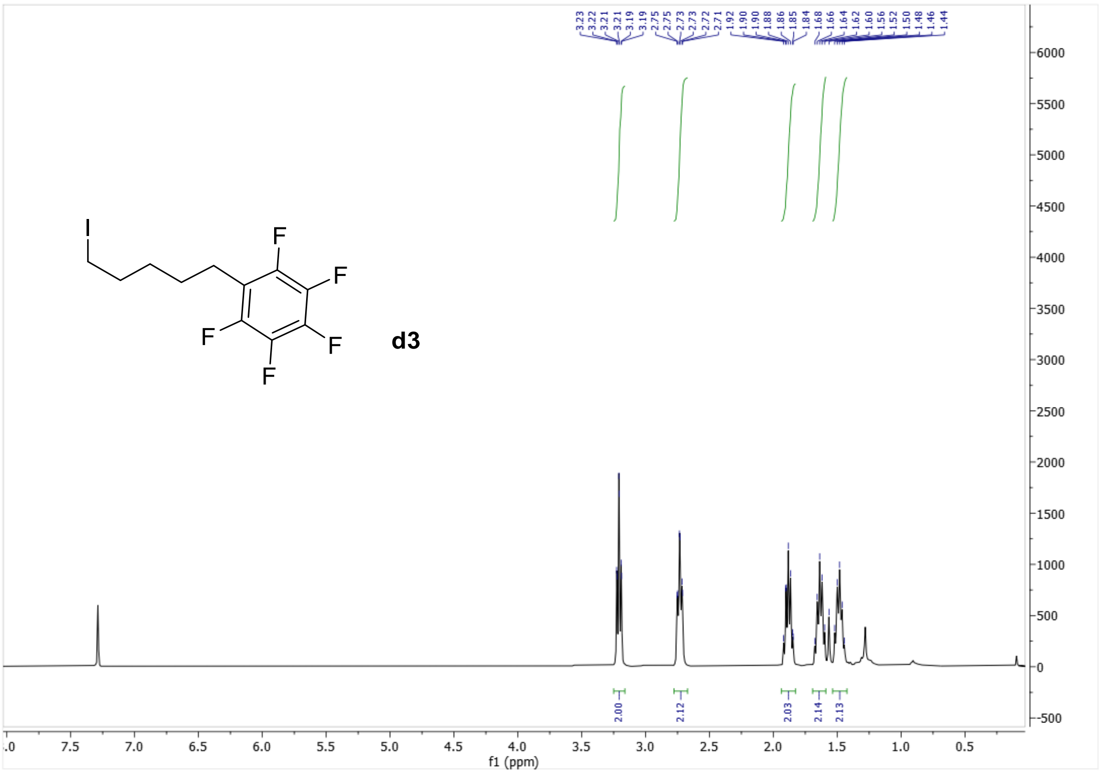

^{1}H-NMR spectrum of (**d3**) (400 MHz, $CDCl_3$)

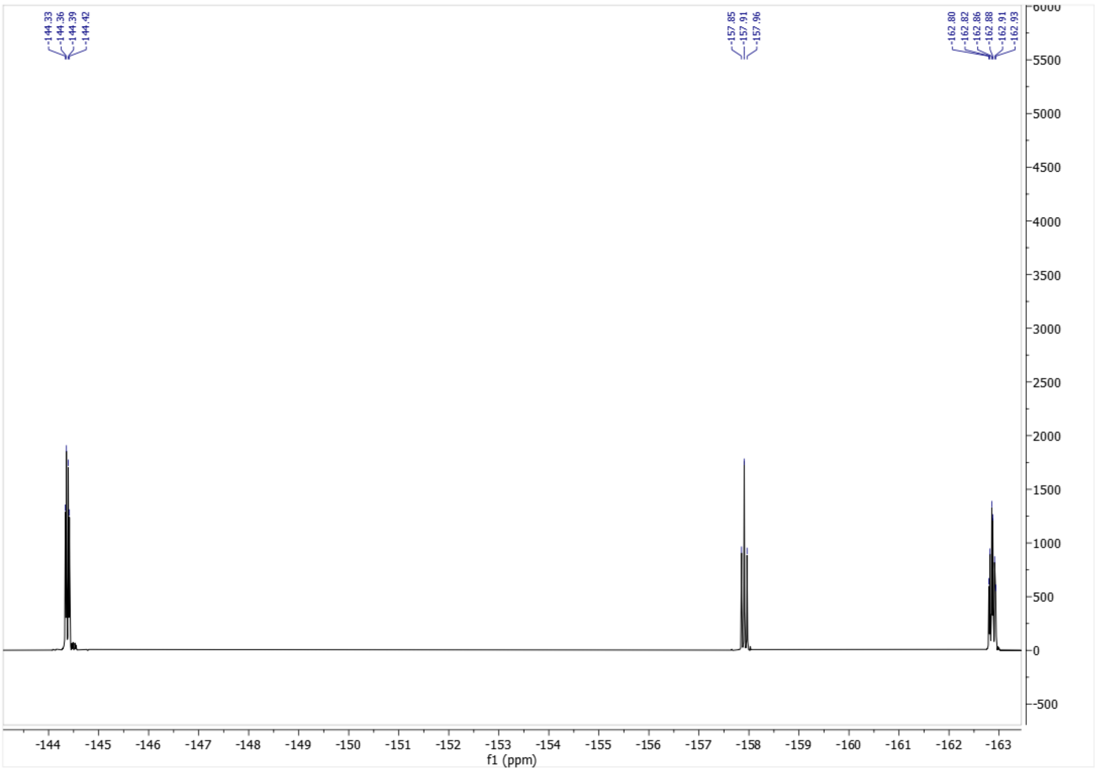

^{19}F-NMR spectrum of (**d3**) (400 MHz, $CDCl_3$)

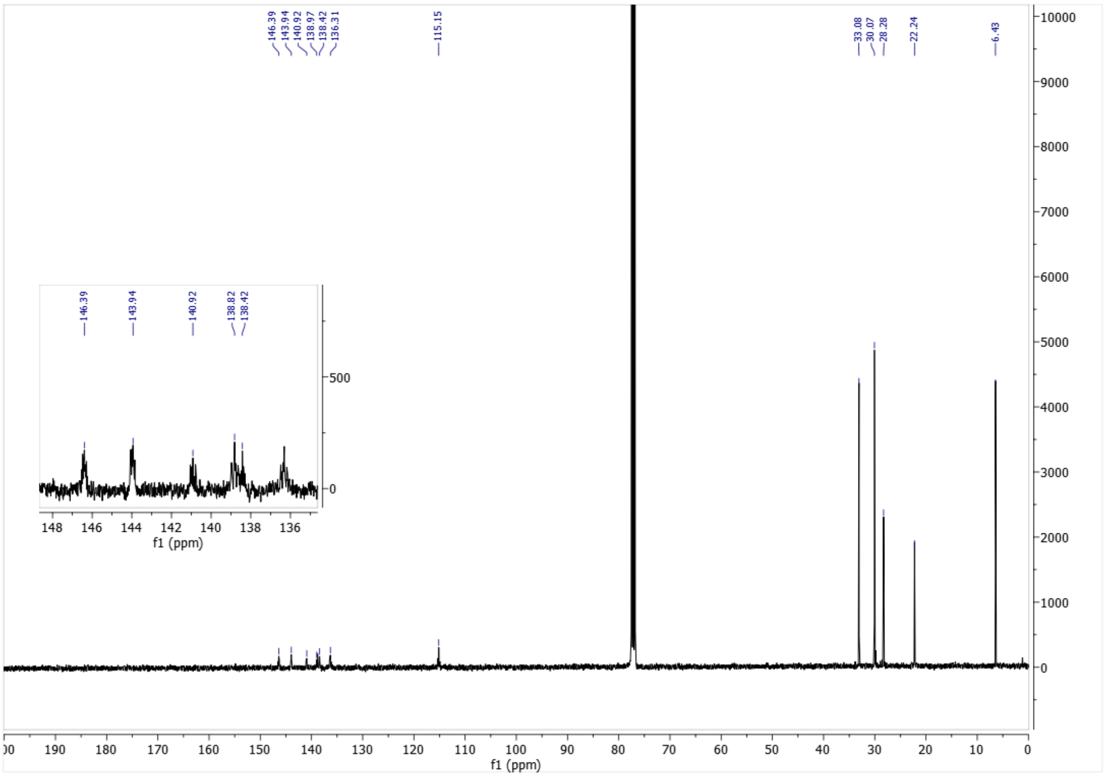

^{13}C-NMR spectrum of (**d3**) (400 MHz, $CDCl_3$)

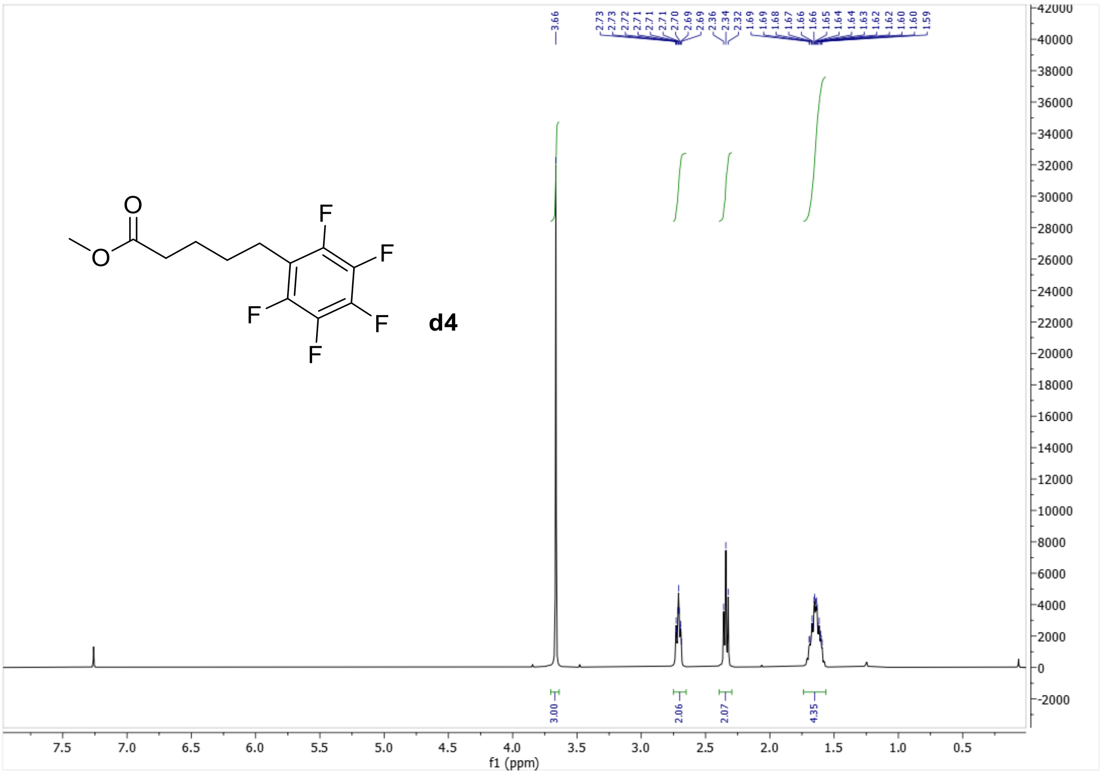

^{1}H-NMR spectrum of (**d4**) (400 MHz, $CDCl_3$)

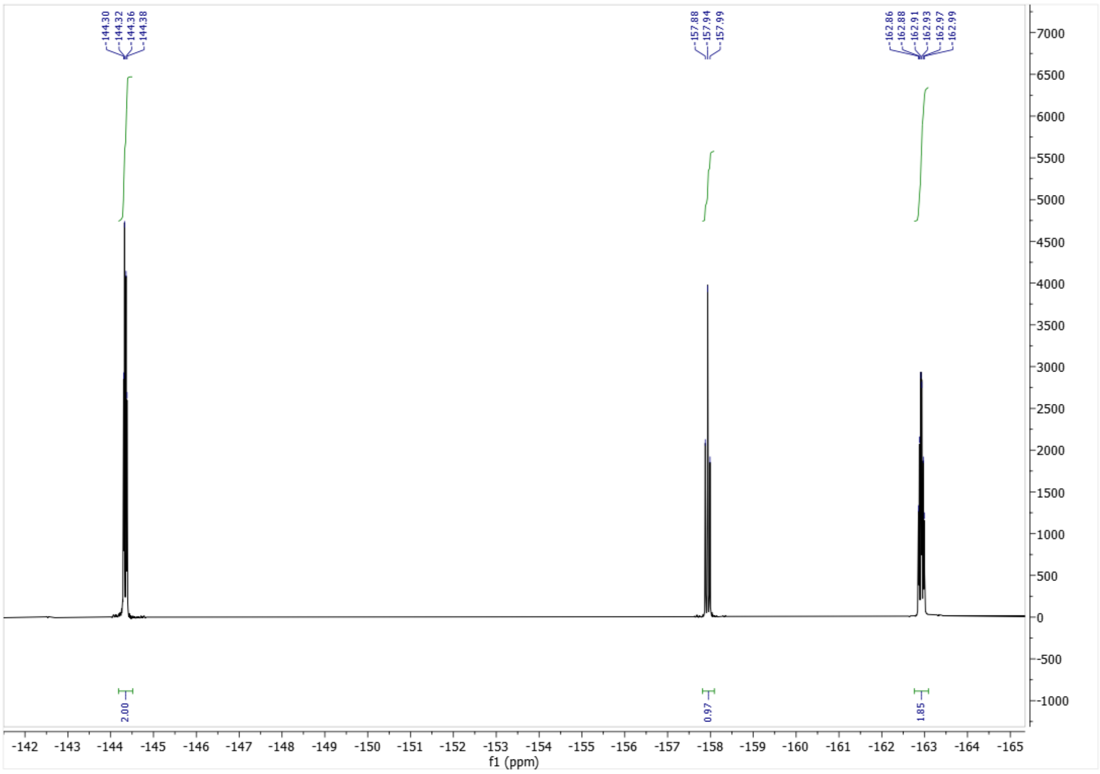

^{19}F-NMR spectrum of (**d4**) (400 MHz, $CDCl_3$)

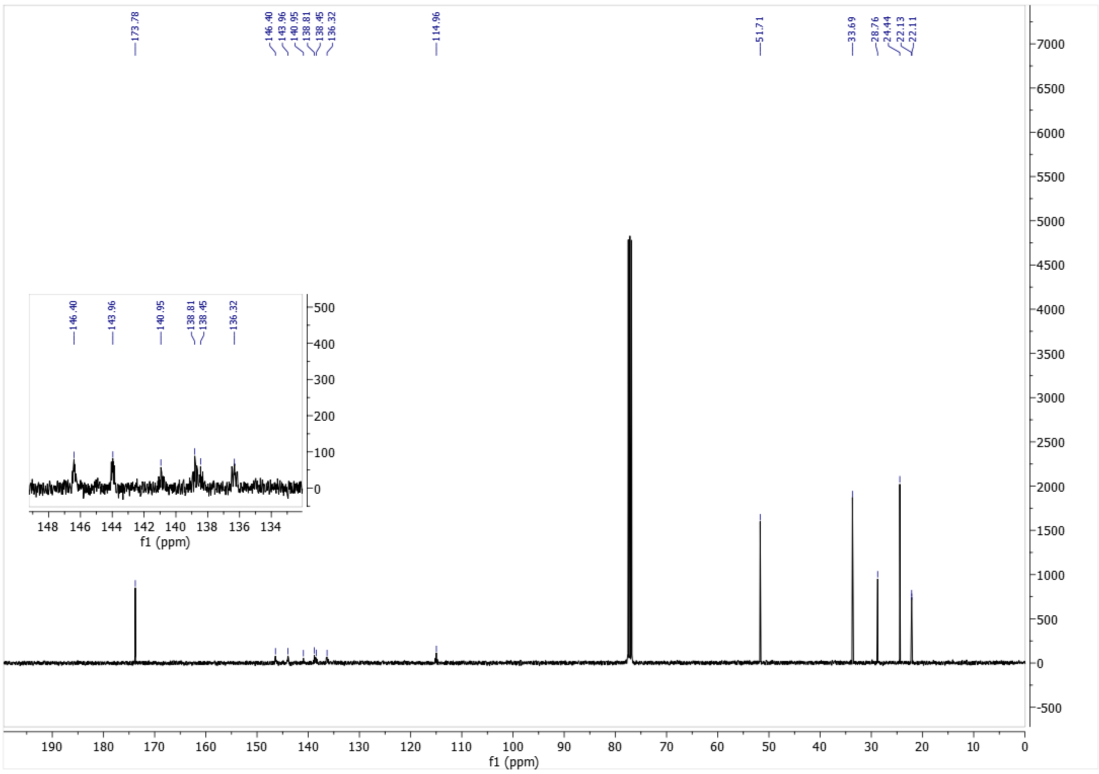

^{13}C-NMR spectrum of (**d4**) (400 MHz, $CDCl_3$)

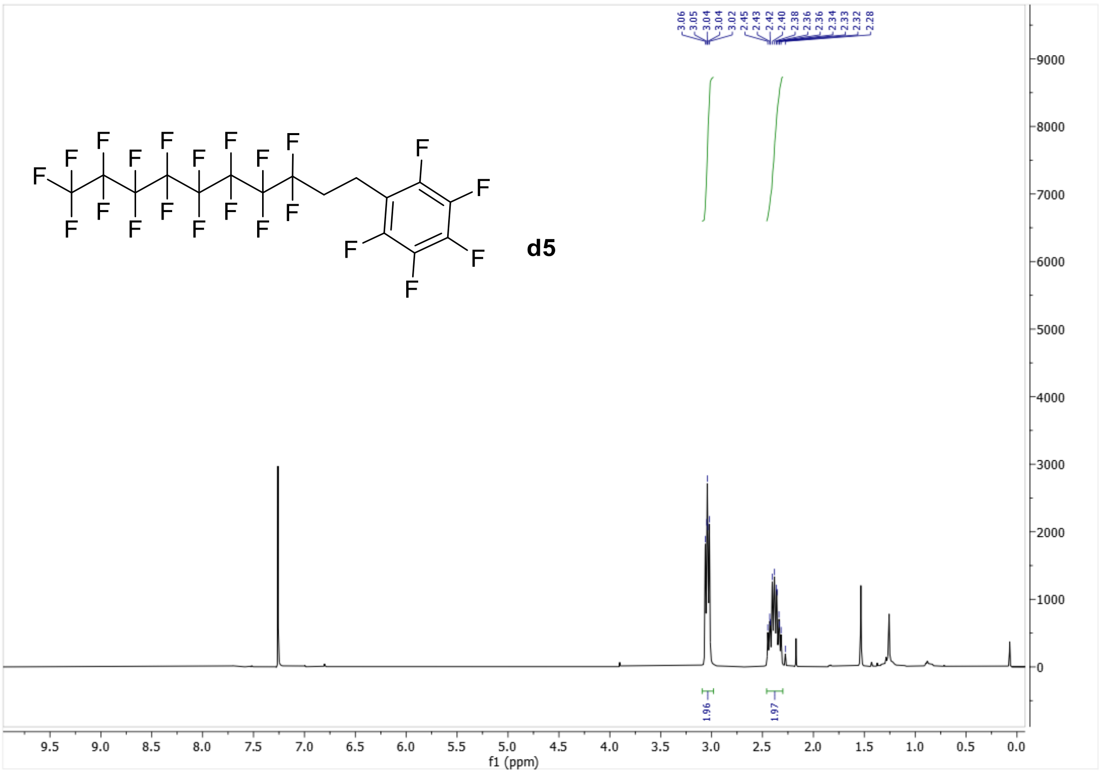

^{1}H-NMR spectrum of (**d5**) (400 MHz, $CDCl_3$)

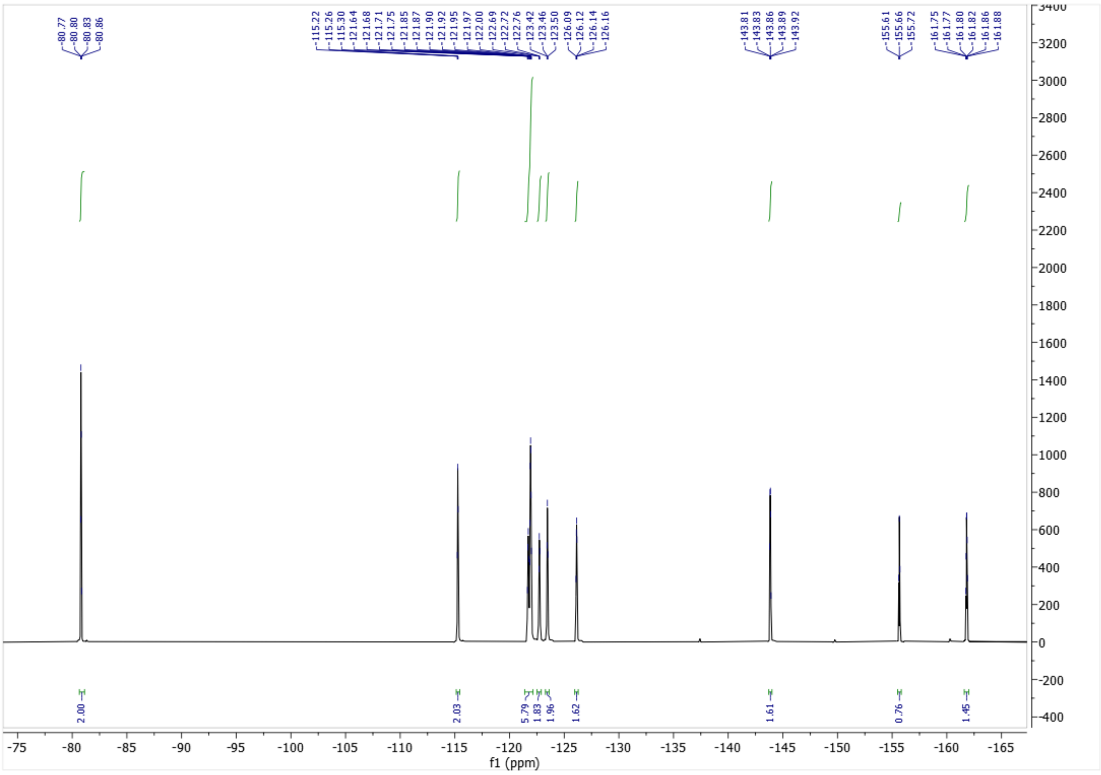

^{19}F-NMR spectrum of (**d5**) (400 MHz, $CDCl_3$)

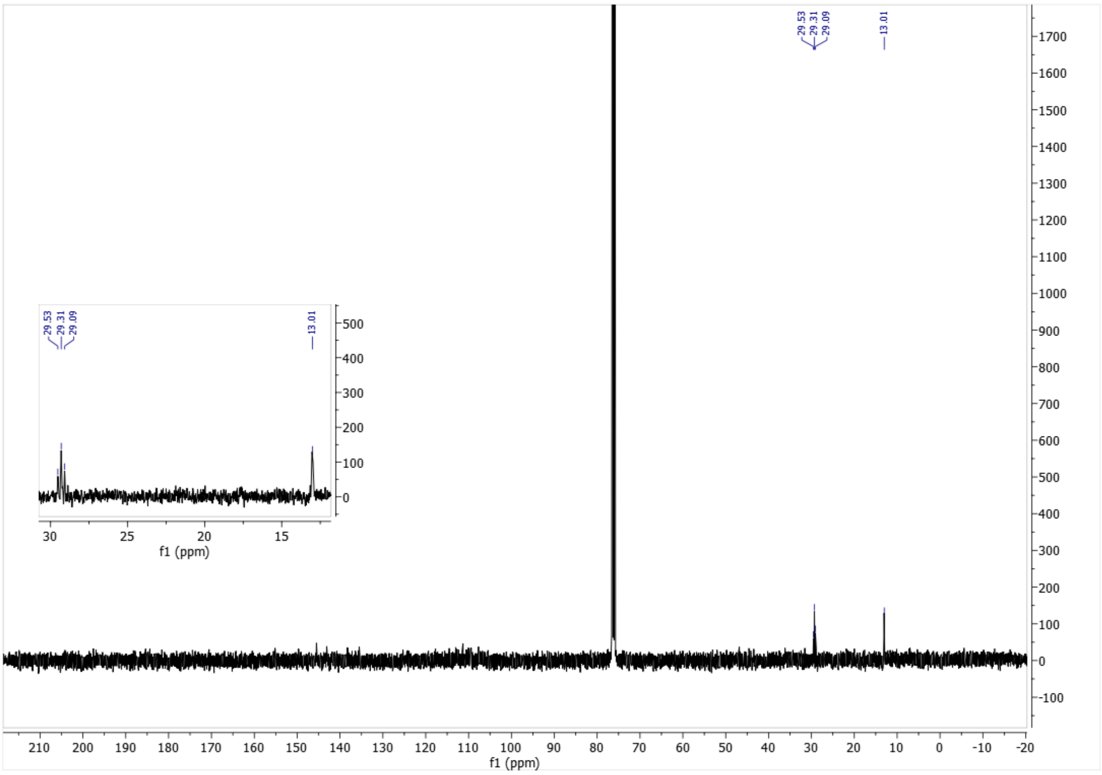

^{13}C-NMR spectrum of (**d5**) (400 MHz, $CDCl_3$)

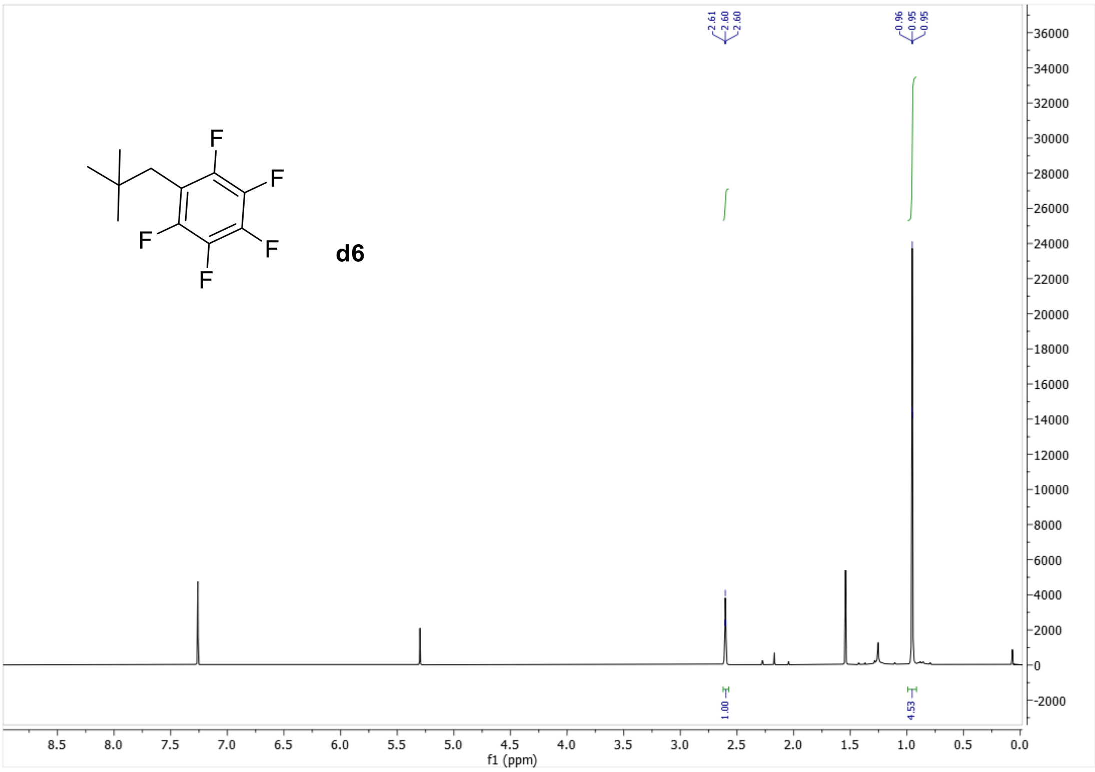

^{1}H-NMR spectrum of (**d6**) (400 MHz, $CDCl_3$)

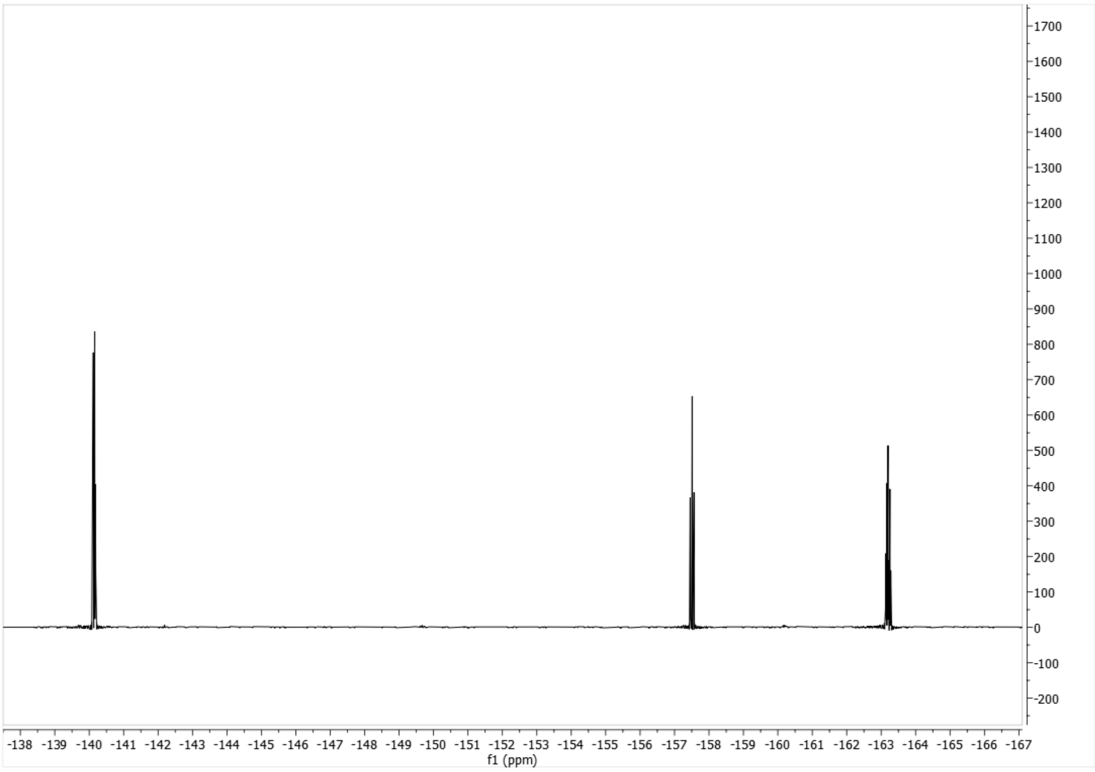

^{19}F-NMR spectrum of (**d6**) (400 MHz, $CDCl_3$)

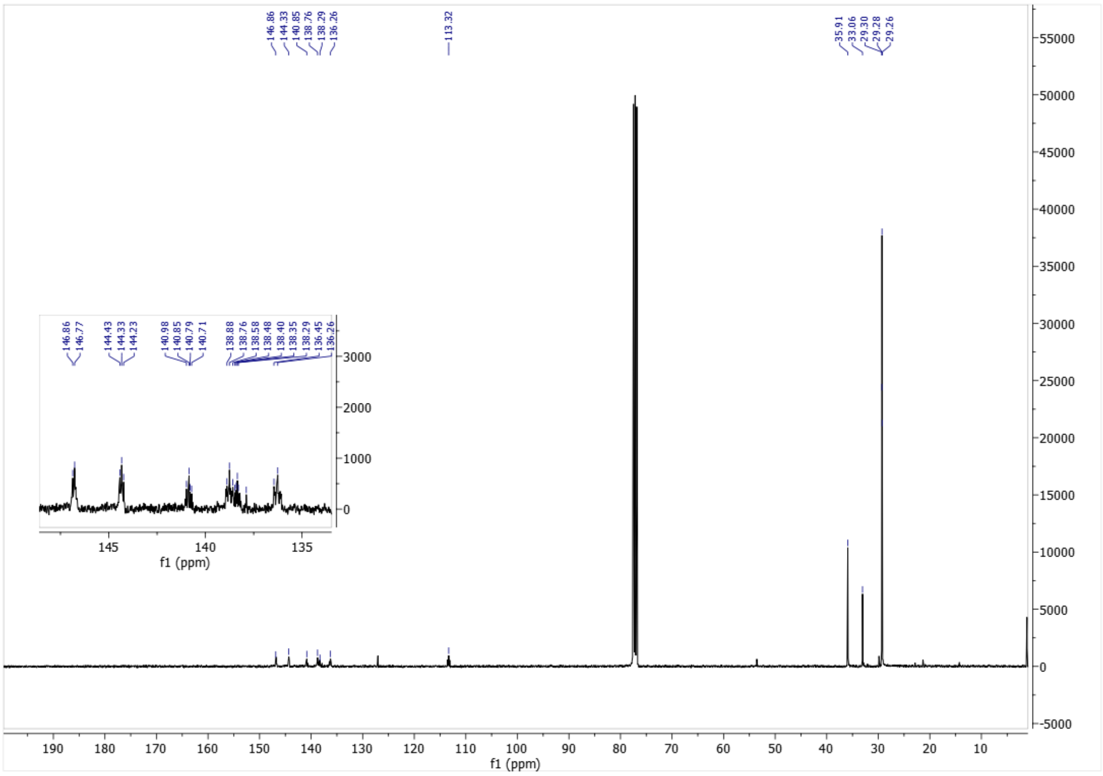

^{13}C-NMR spectrum of (**d6**) (400 MHz, $CDCl_3$)

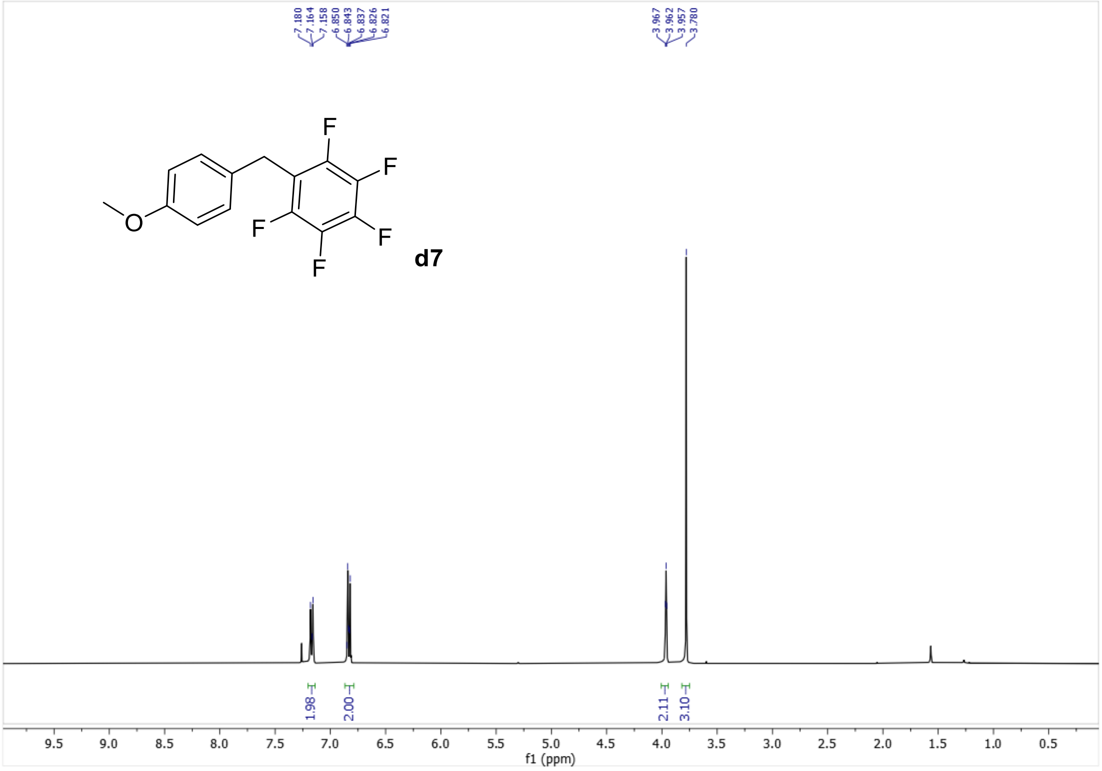

^{1}H-NMR spectrum of (**d7**) (400 MHz, $CDCl_3$)

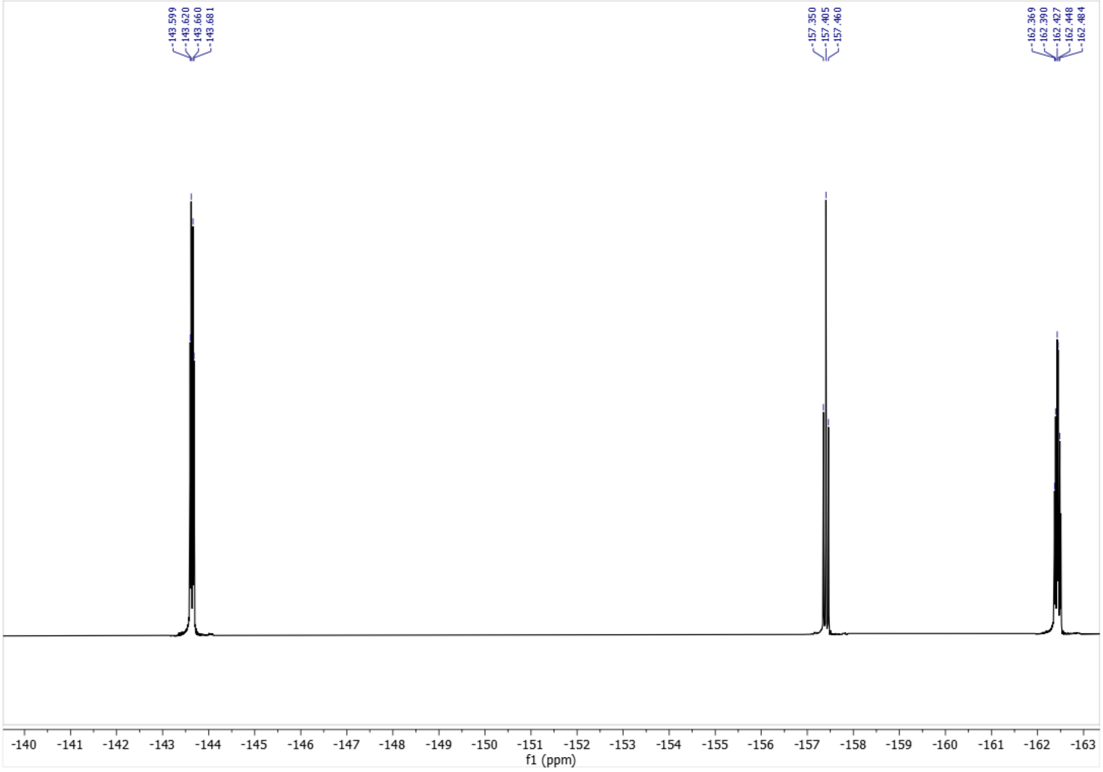

^{19}F-NMR spectrum of (**d7**) (400 MHz, $CDCl_3$)

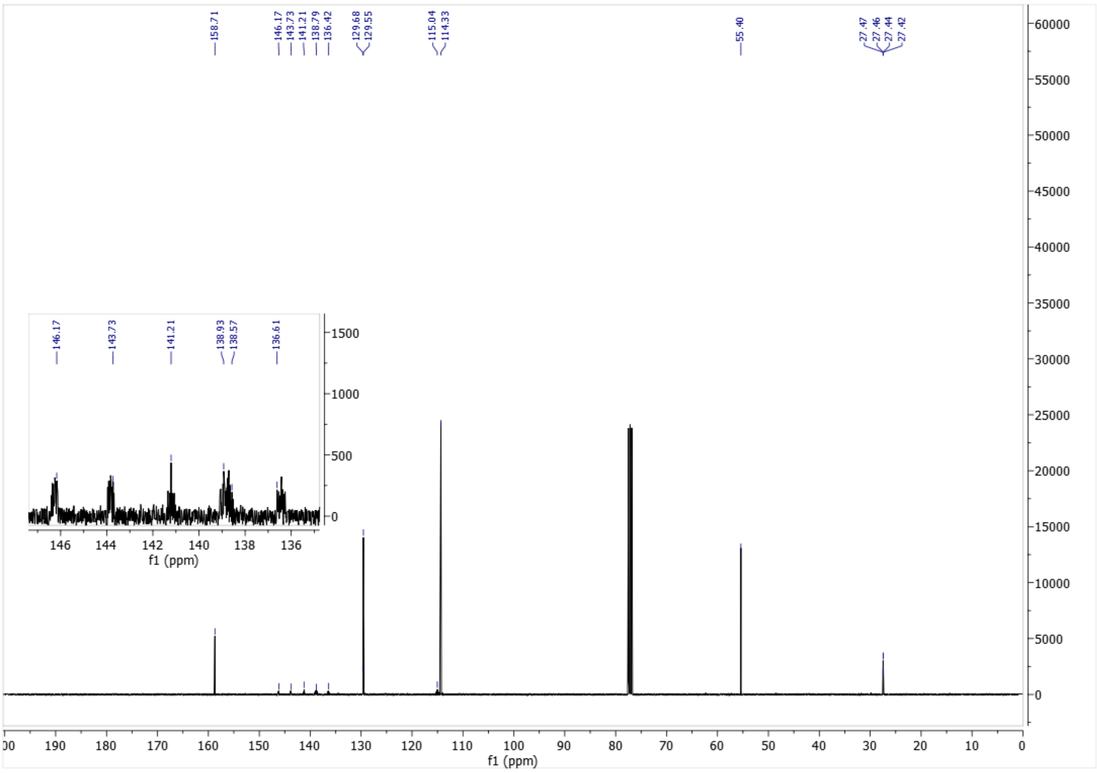

^{13}C-NMR spectrum of (**d7**) (400 MHz, $CDCl_3$)

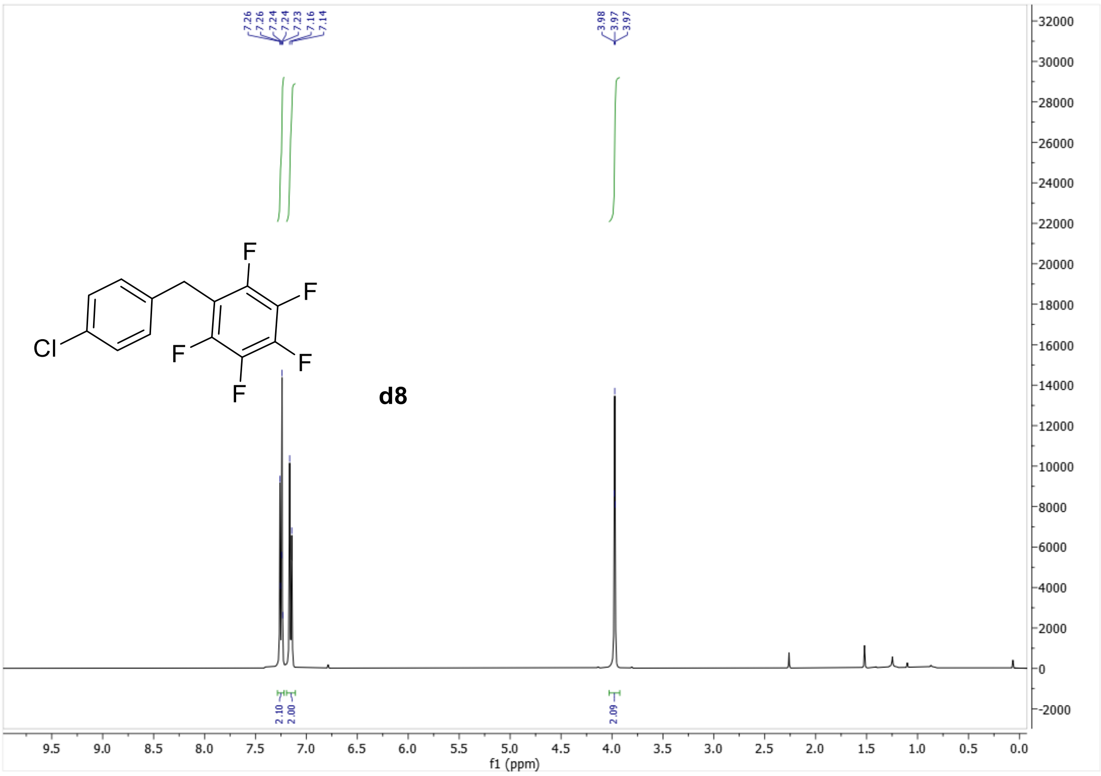

^{1}H-NMR spectrum of (**d8**) (400 MHz, $CDCl_3$)

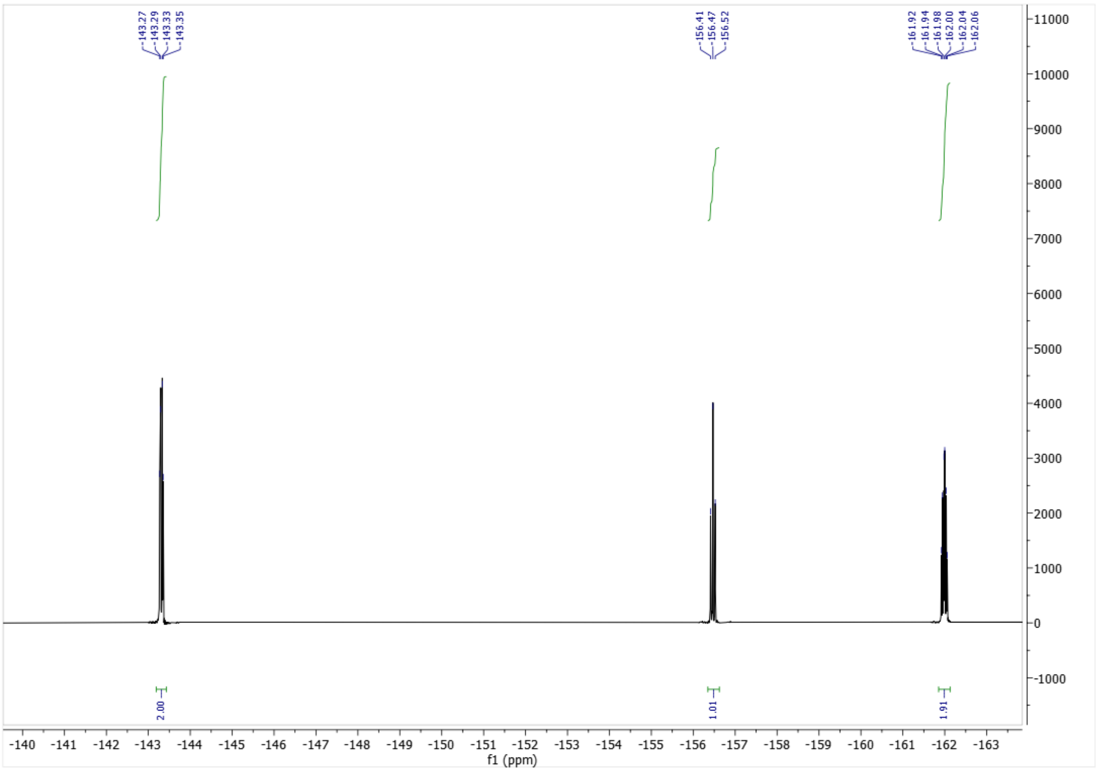

^{19}F-NMR spectrum of (**d8**) (400 MHz, $CDCl_3$)

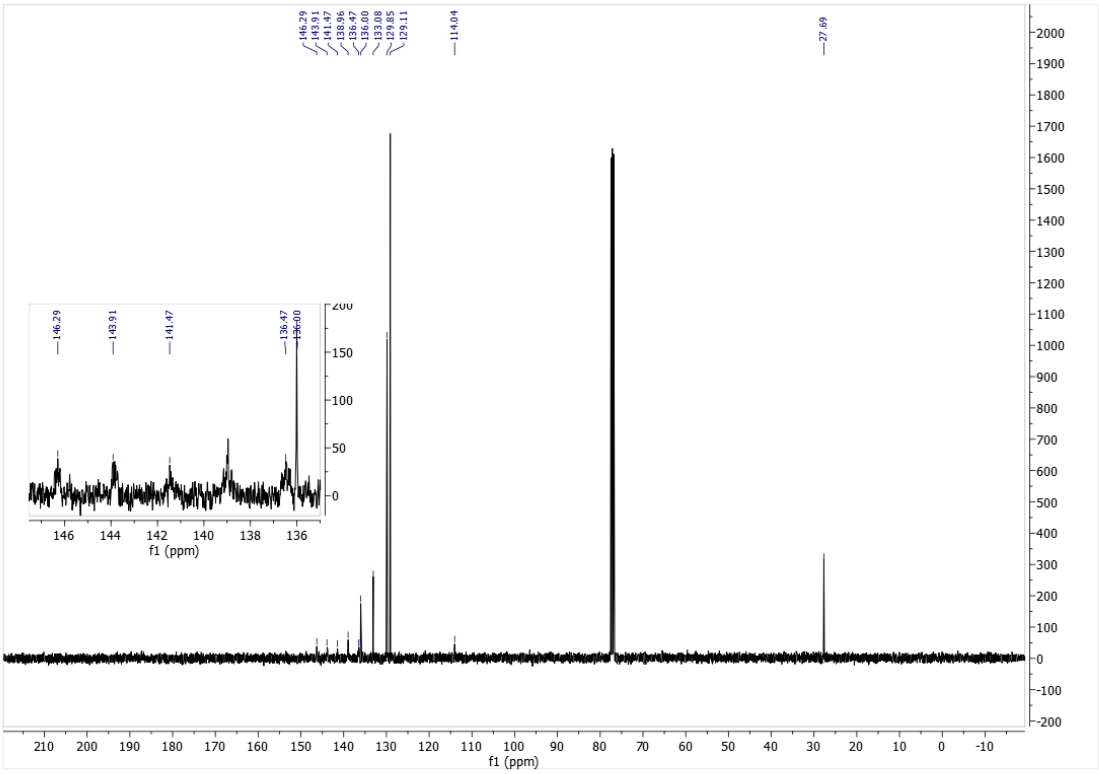

^{13}C-NMR spectrum of (**d8**) (400 MHz, $CDCl_3$)

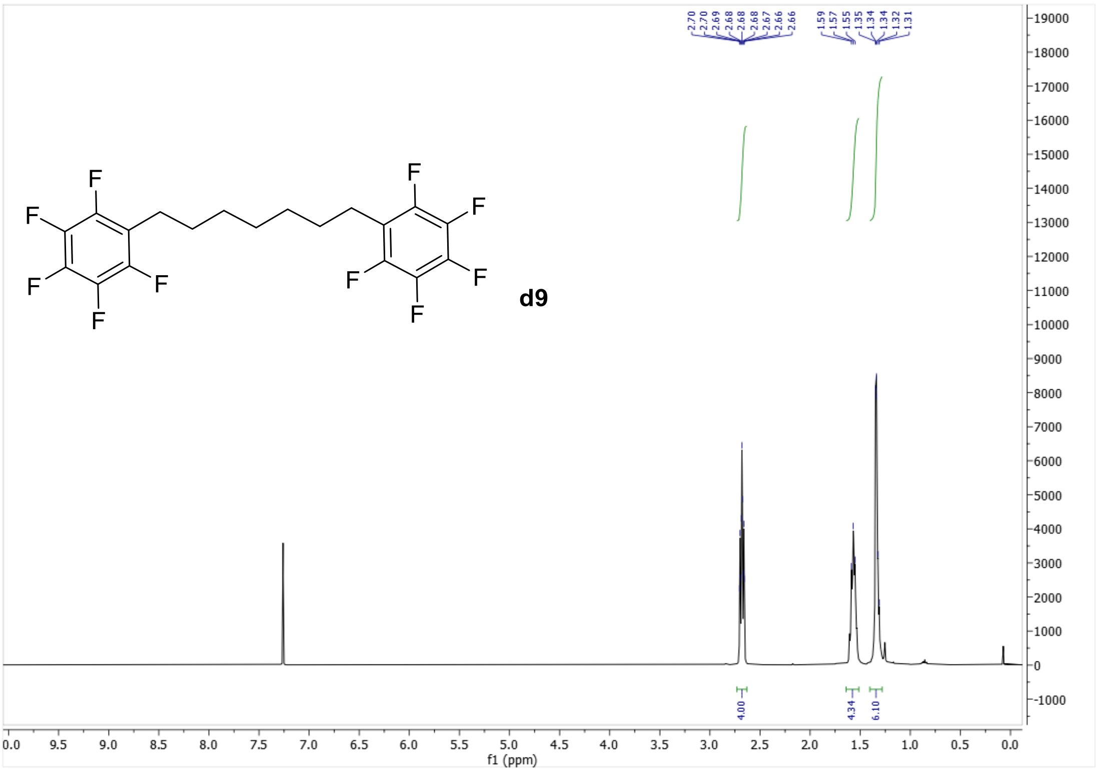

^{1}H-NMR spectrum of (**d9**) (400 MHz, $CDCl_3$)

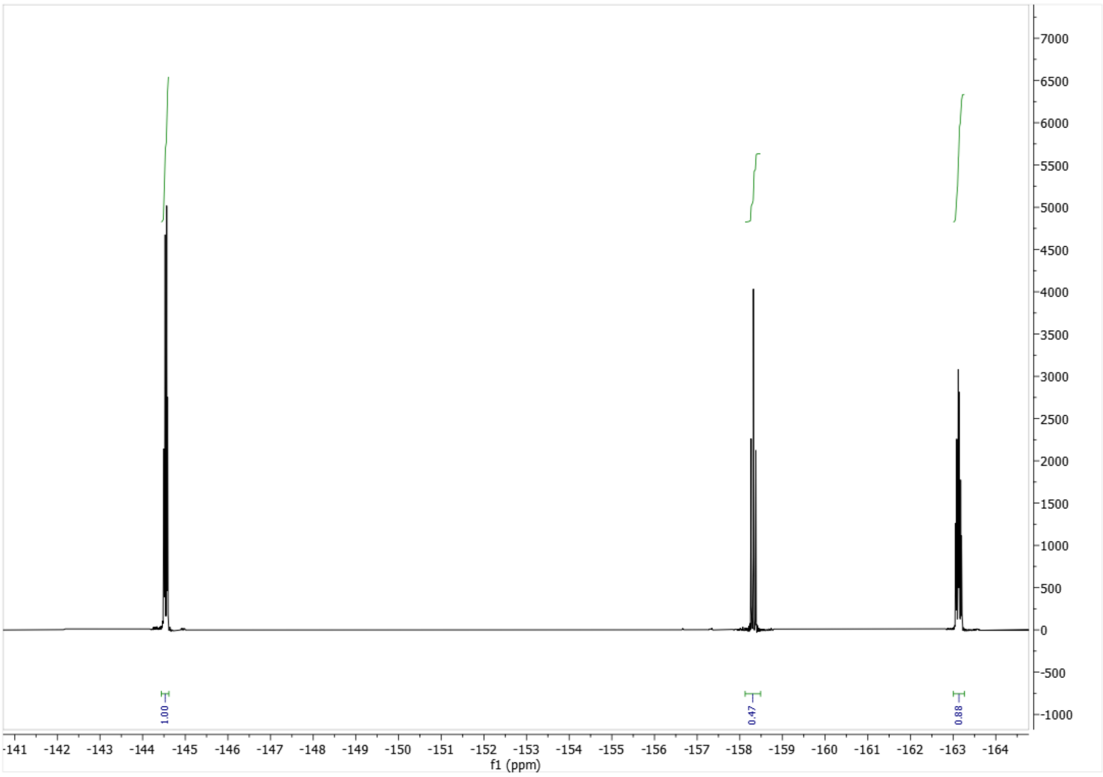

^{19}F-NMR spectrum of (**d9**) (400 MHz, $CDCl_3$)

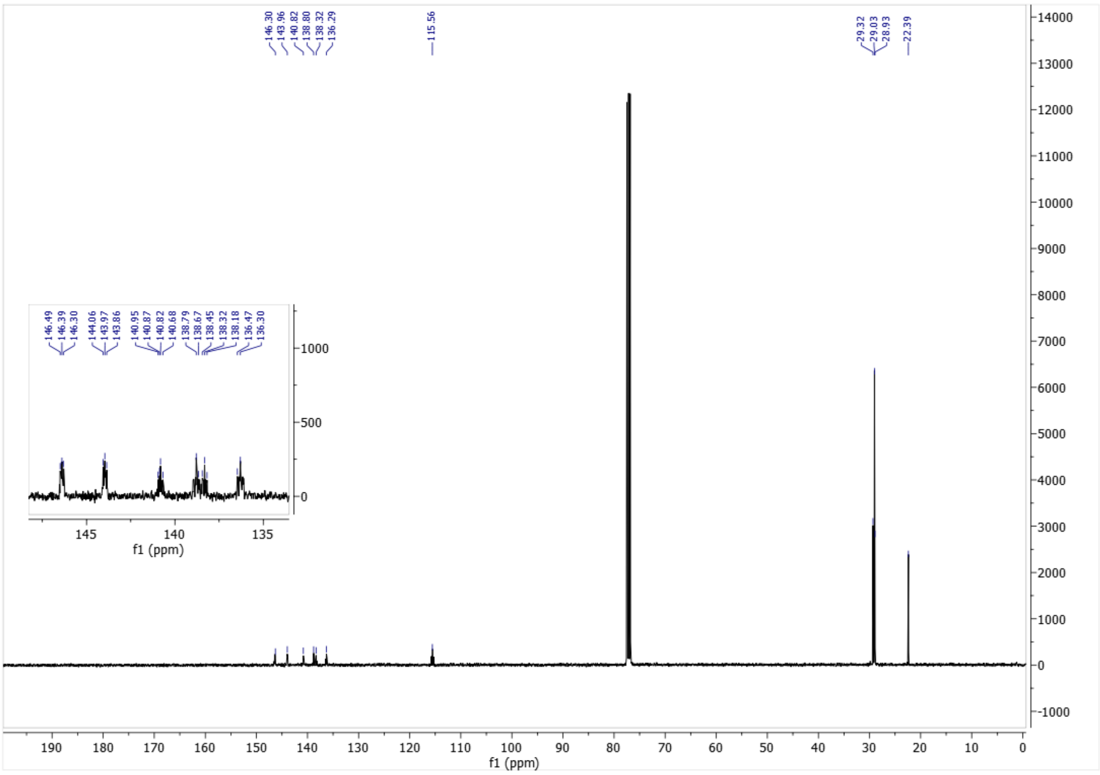

^{13}C-NMR spectrum of (**d9**) (400 MHz, $CDCl_3$)

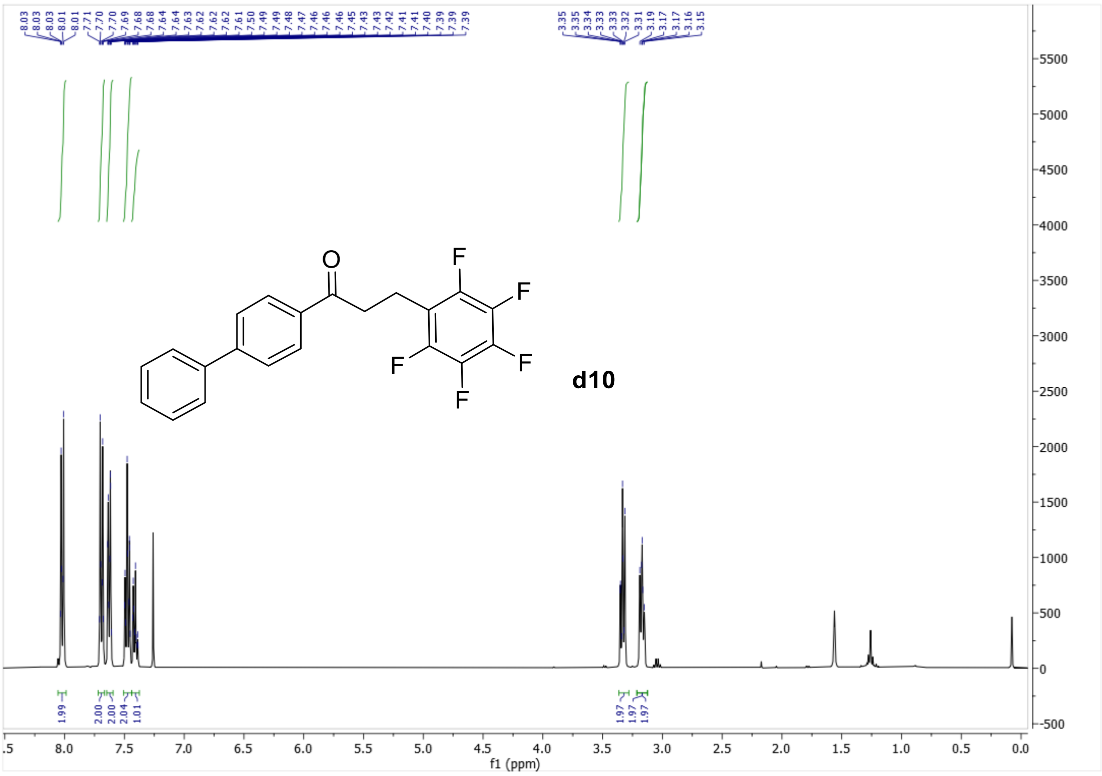

^{1}H-NMR spectrum of (**d10**) (400 MHz, $CDCl_3$)

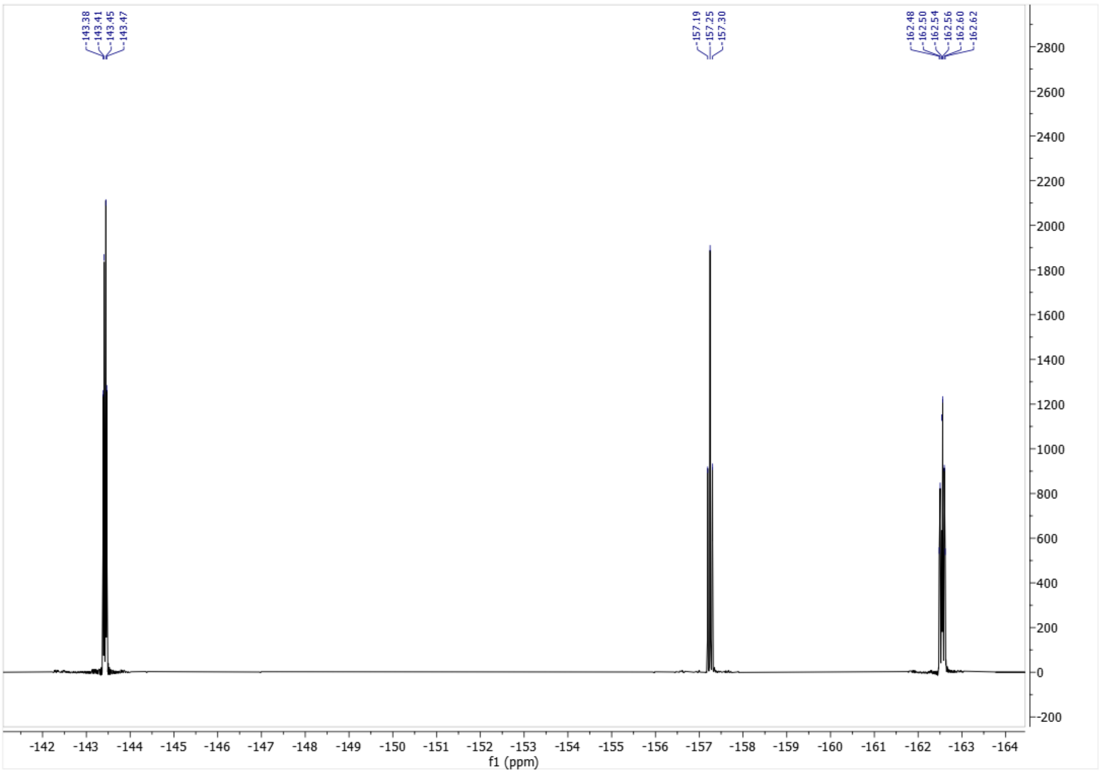

^{19}F-NMR spectrum of (**d10**) (400 MHz, $CDCl_3$)

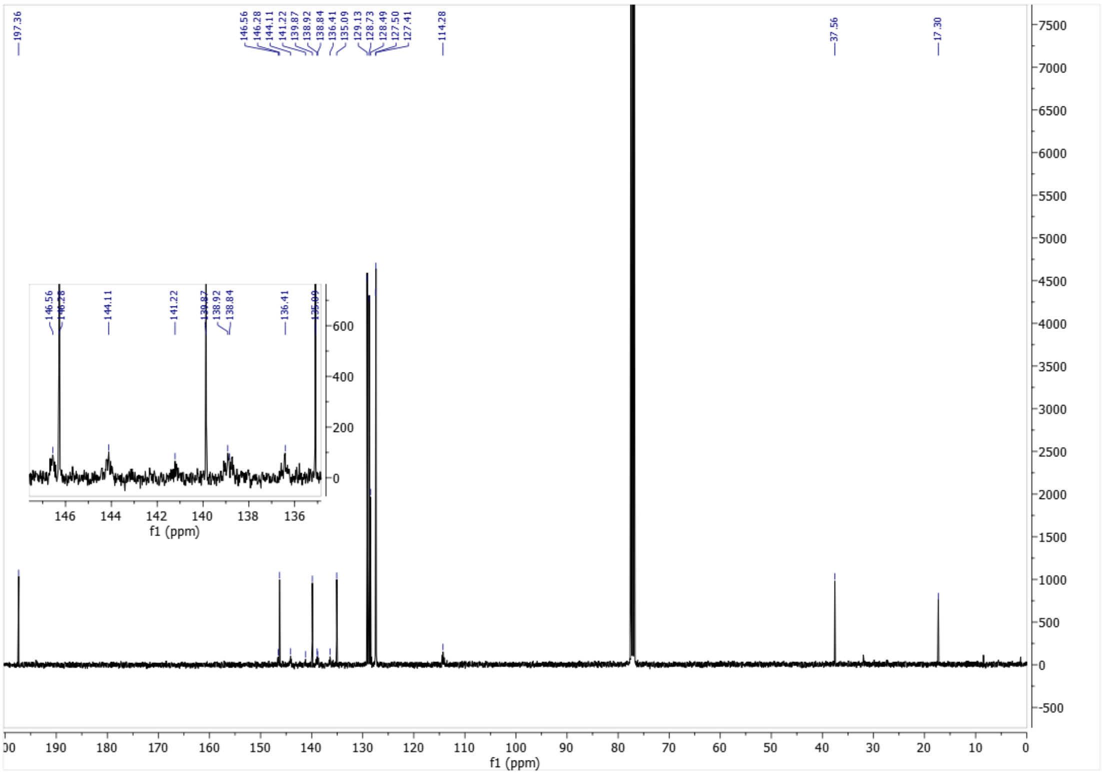

^{13}C-NMR spectrum of (**d10**) (400 MHz, $CDCl_3$)

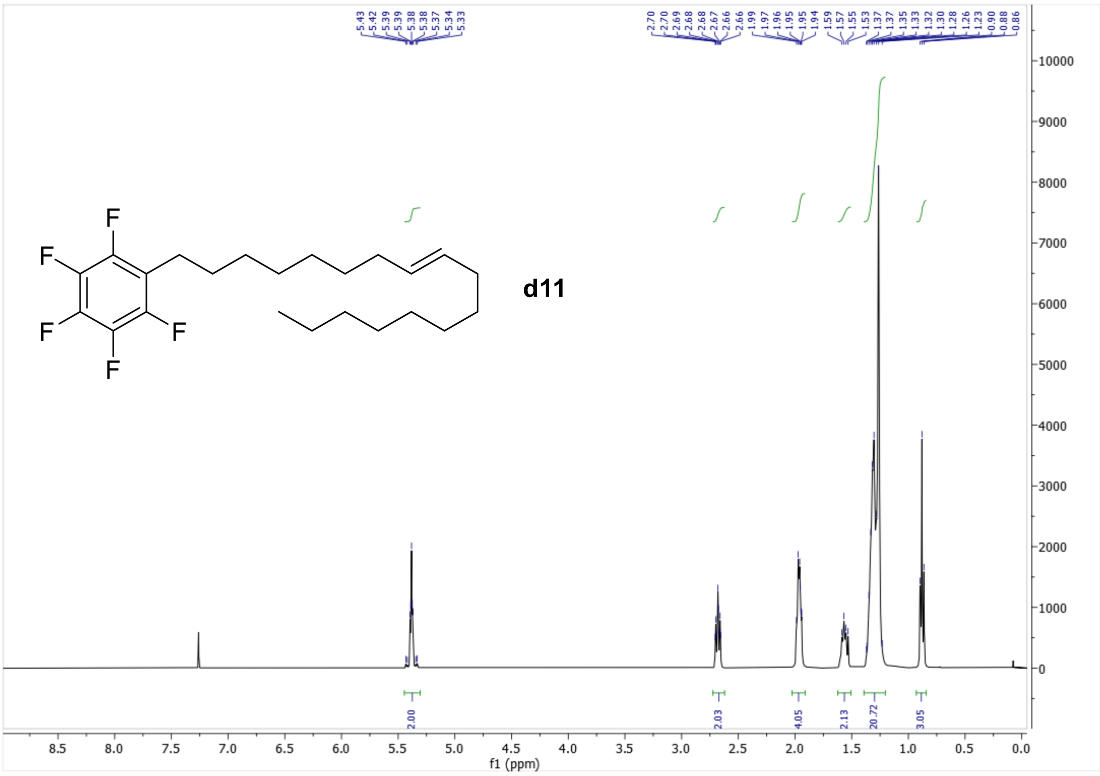

^{1}H-NMR spectrum of (**d11**) (400 MHz, $CDCl_3$)

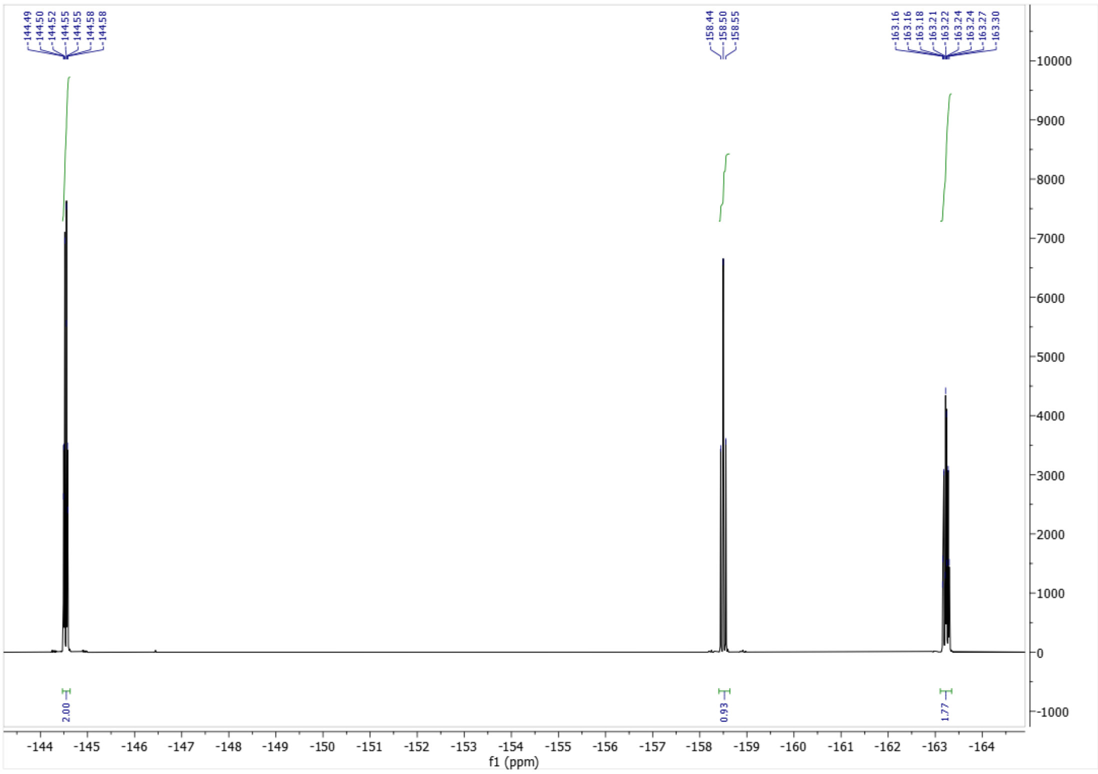

^{19}F-NMR spectrum of (**d11**) (400 MHz, $CDCl_3$)

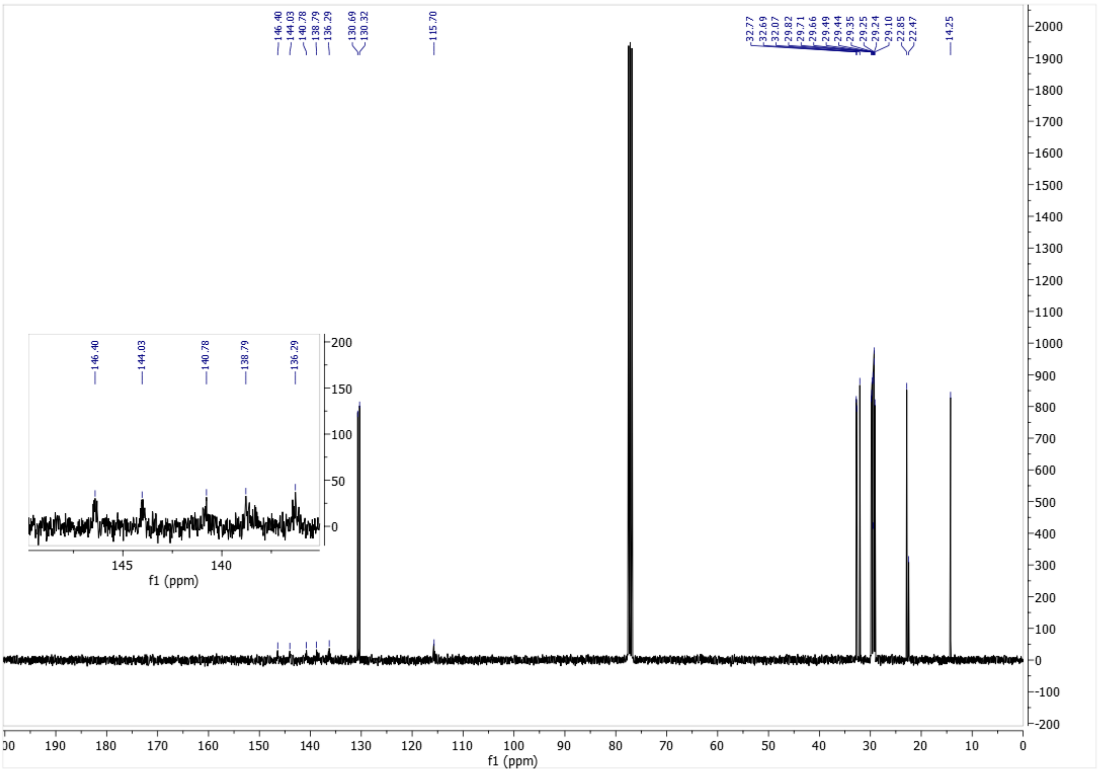

^{13}C-NMR spectrum of (**d11**) (400 MHz, $CDCl_3$)

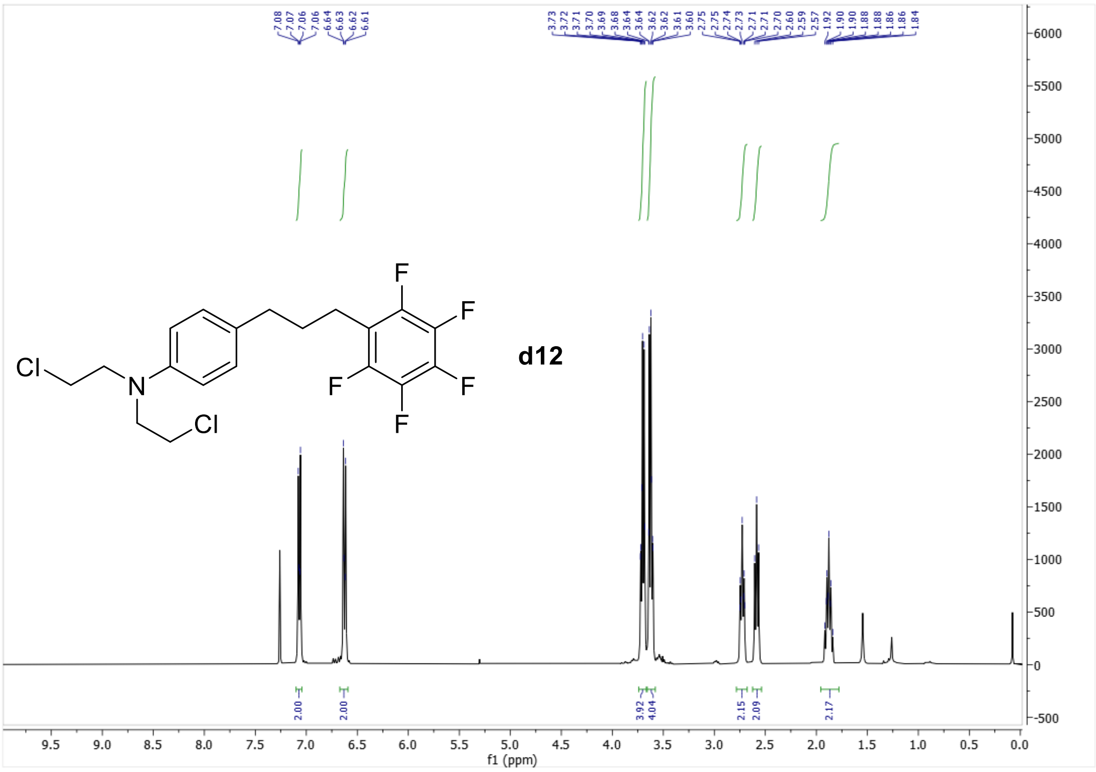

^{1}H-NMR spectrum of (**d12**) (400 MHz, $CDCl_3$)

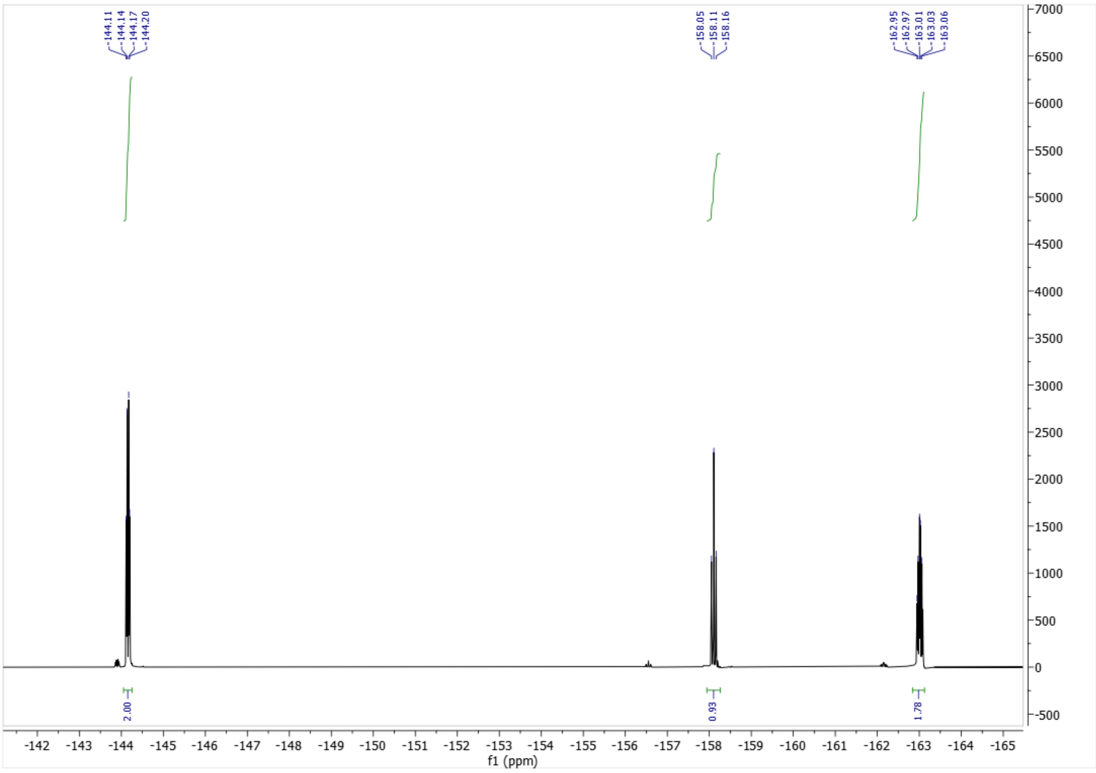

^{19}F-NMR spectrum of (**d12**) (400 MHz, $CDCl_3$)

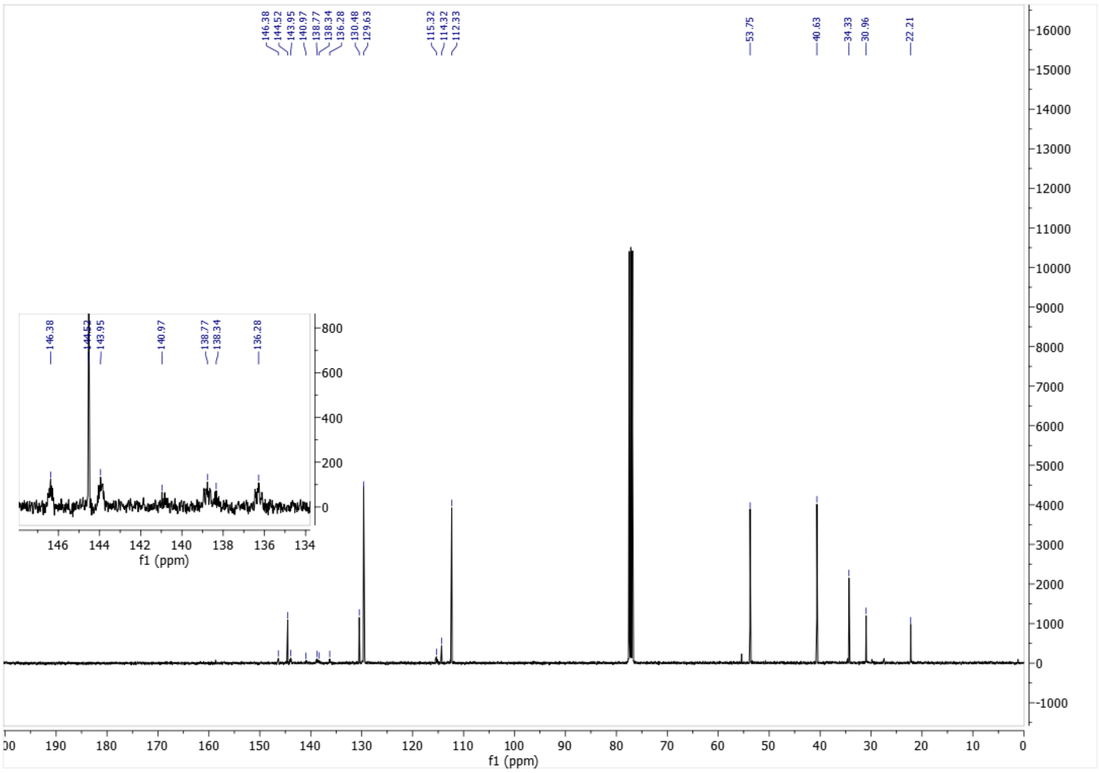

^{13}C-NMR spectrum of (**d12**) (400 MHz, $CDCl_3$)

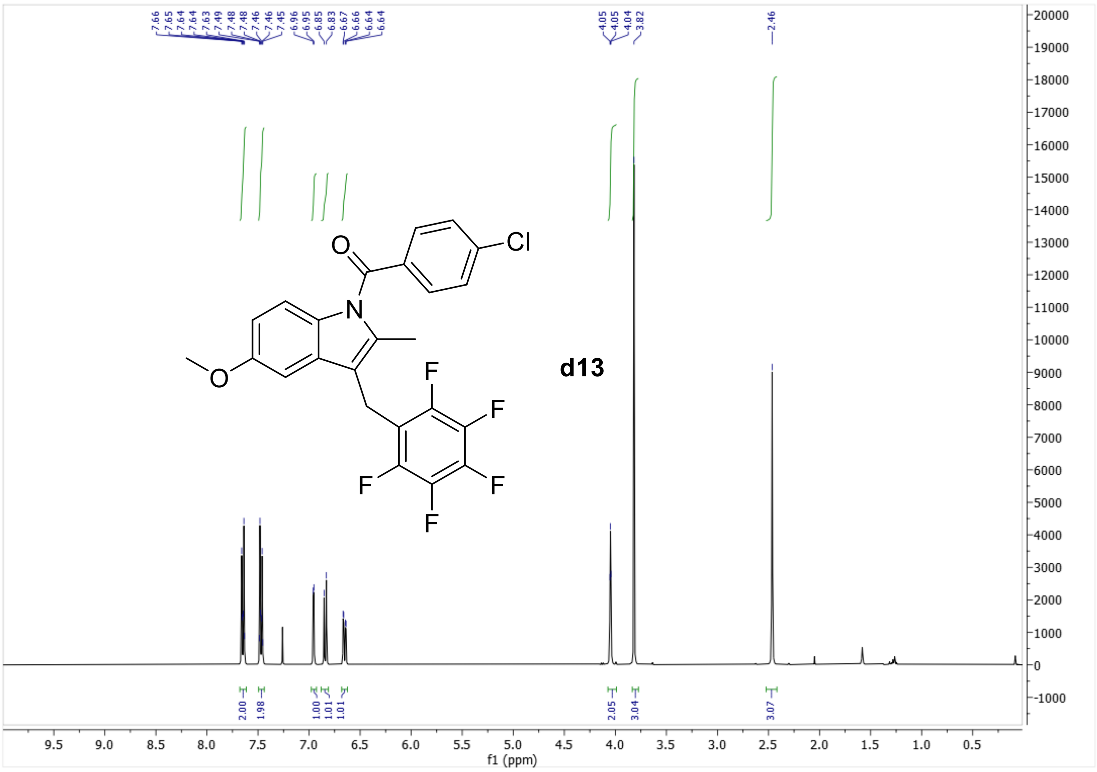

^{1}H-NMR spectrum of (**d13**) (400 MHz, $CDCl_3$)

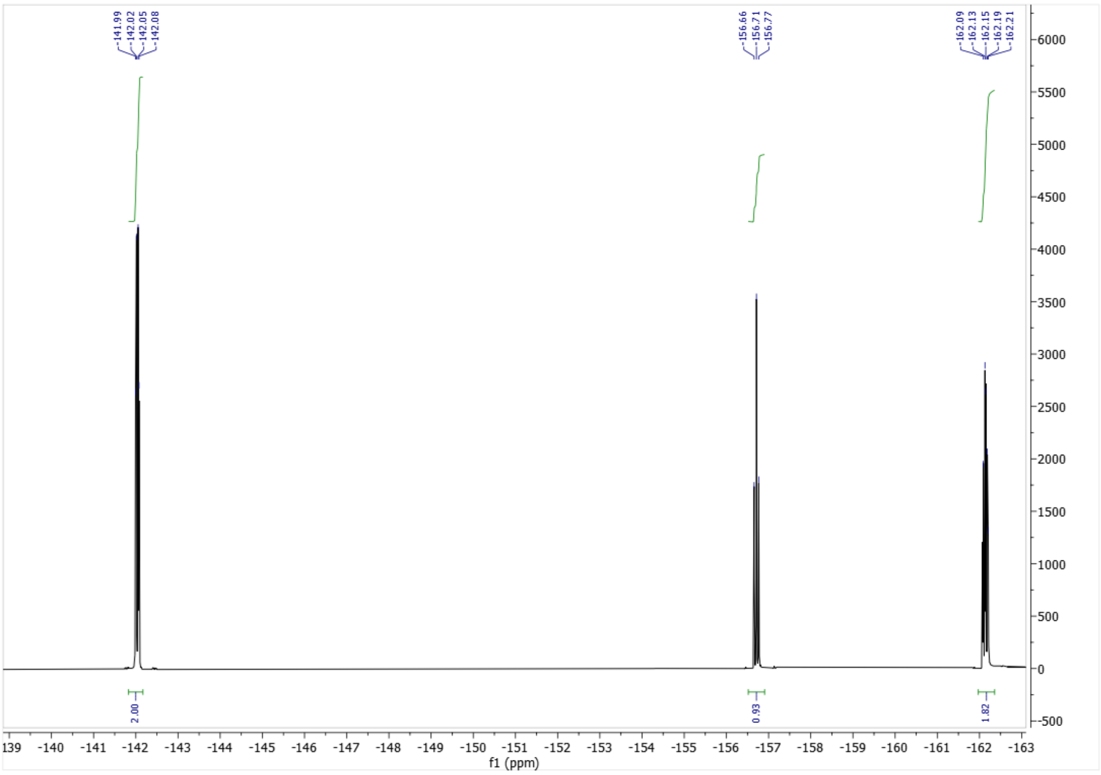

^{19}F-NMR spectrum of (**d13**) (400 MHz, $CDCl_3$)

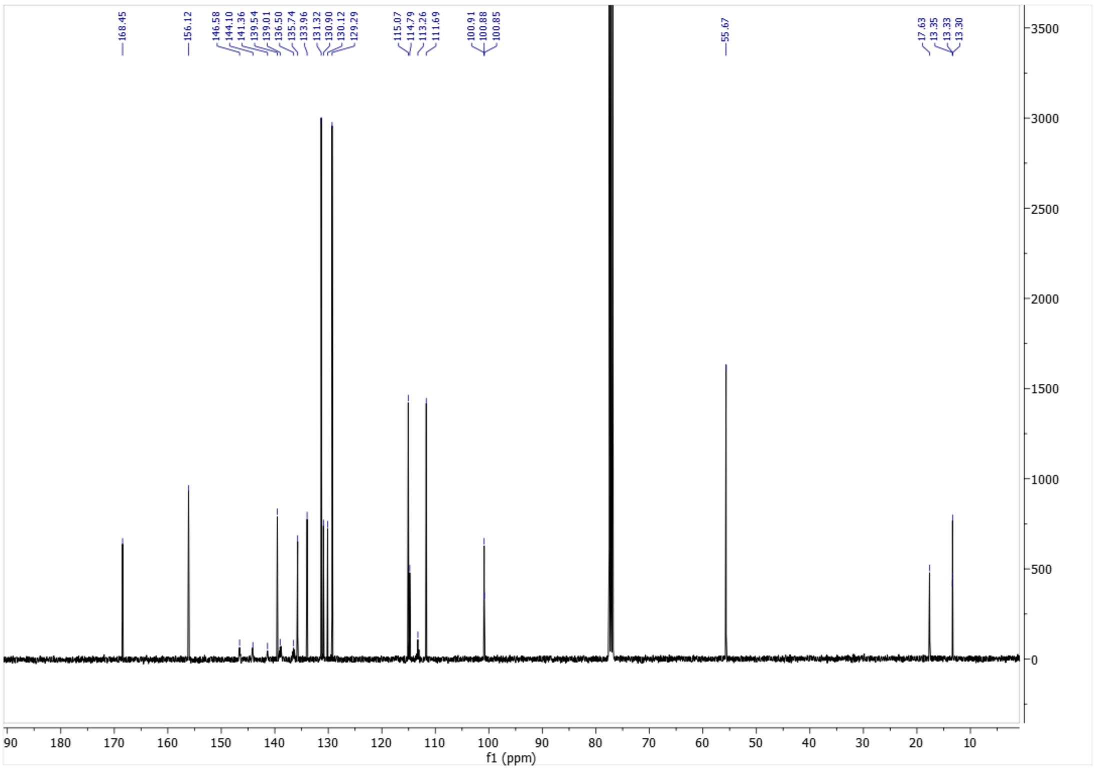

^{13}C-NMR spectrum of (**d13**) (400 MHz, $CDCl_3$)

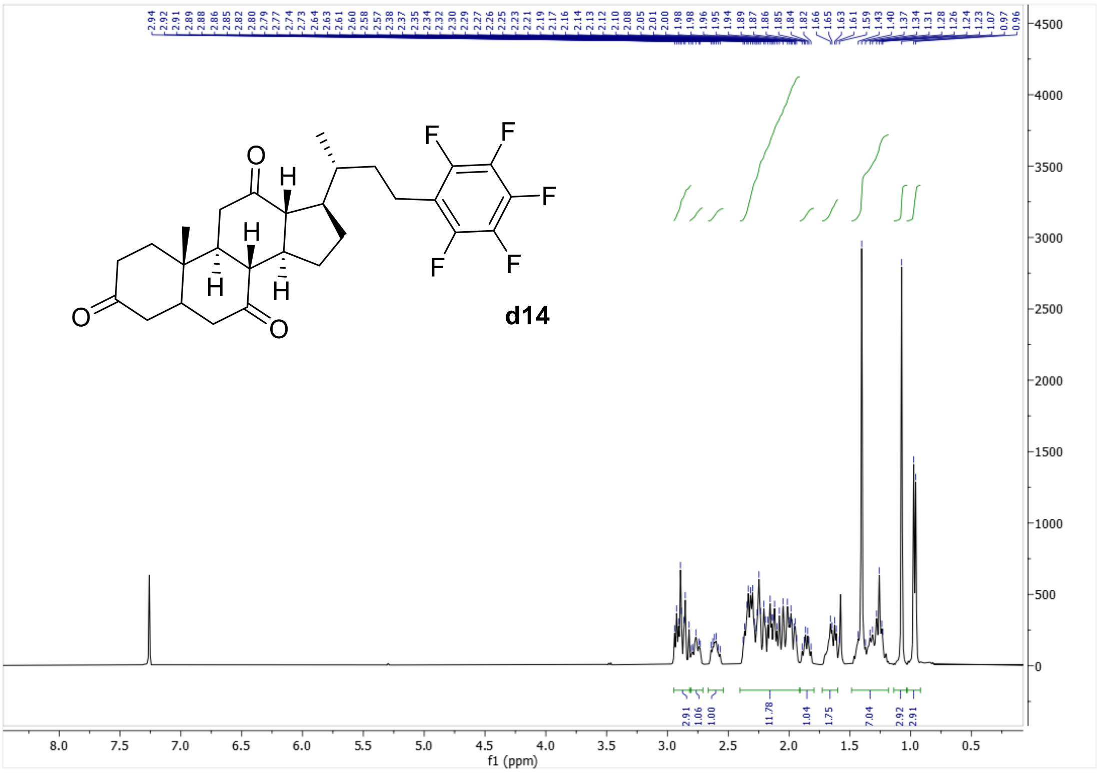

^{1}H-NMR spectrum of (**d14**) (400 MHz, $CDCl_3$)

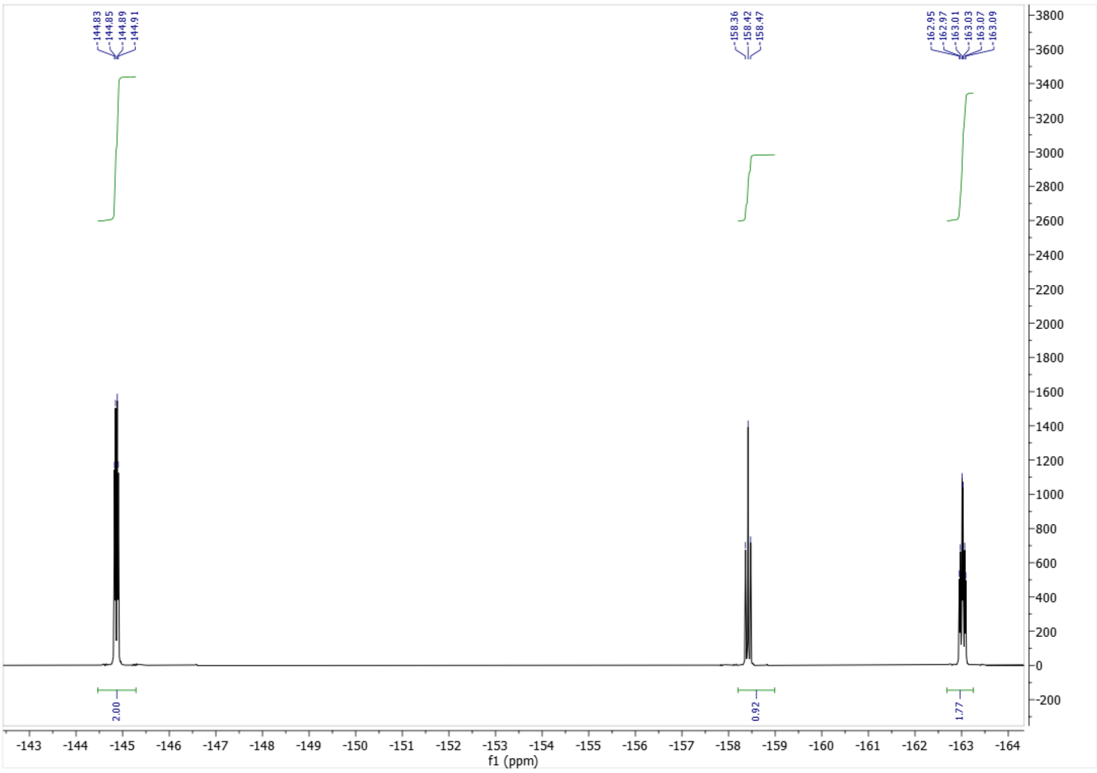

^{19}F-NMR spectrum of (**d14**) (400 MHz, $CDCl_3$)

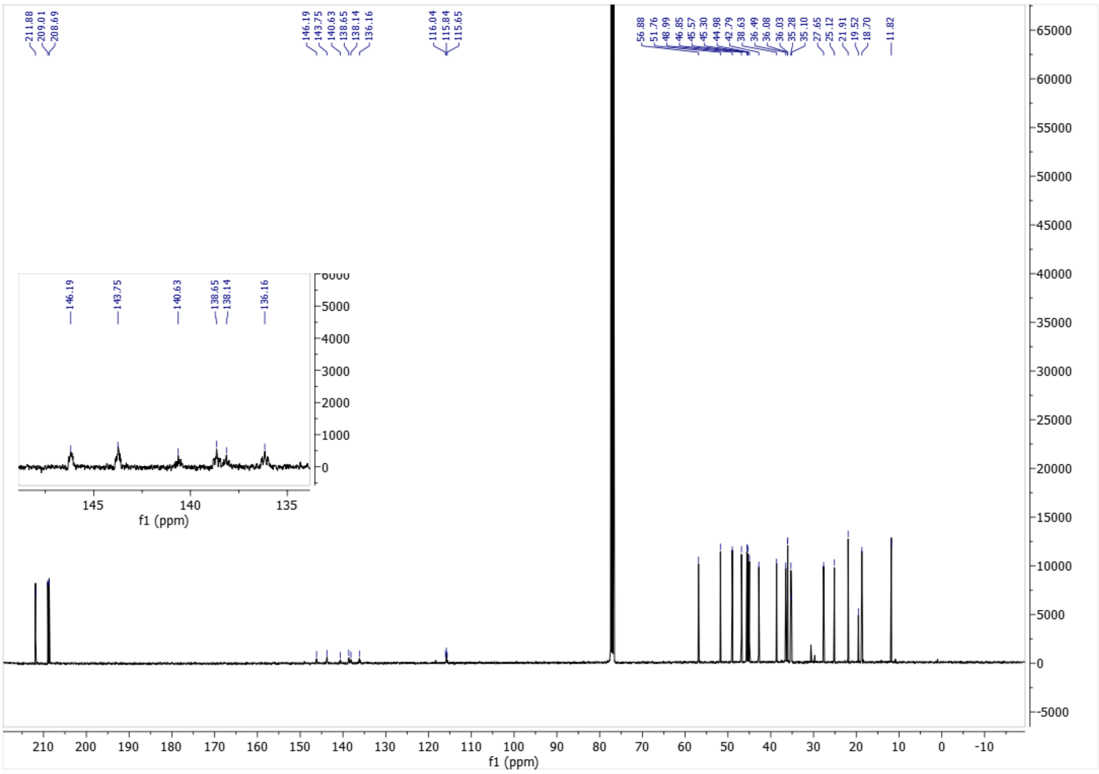

^{13}C-NMR spectrum of (**d14**) (400 MHz, $CDCl_3$)

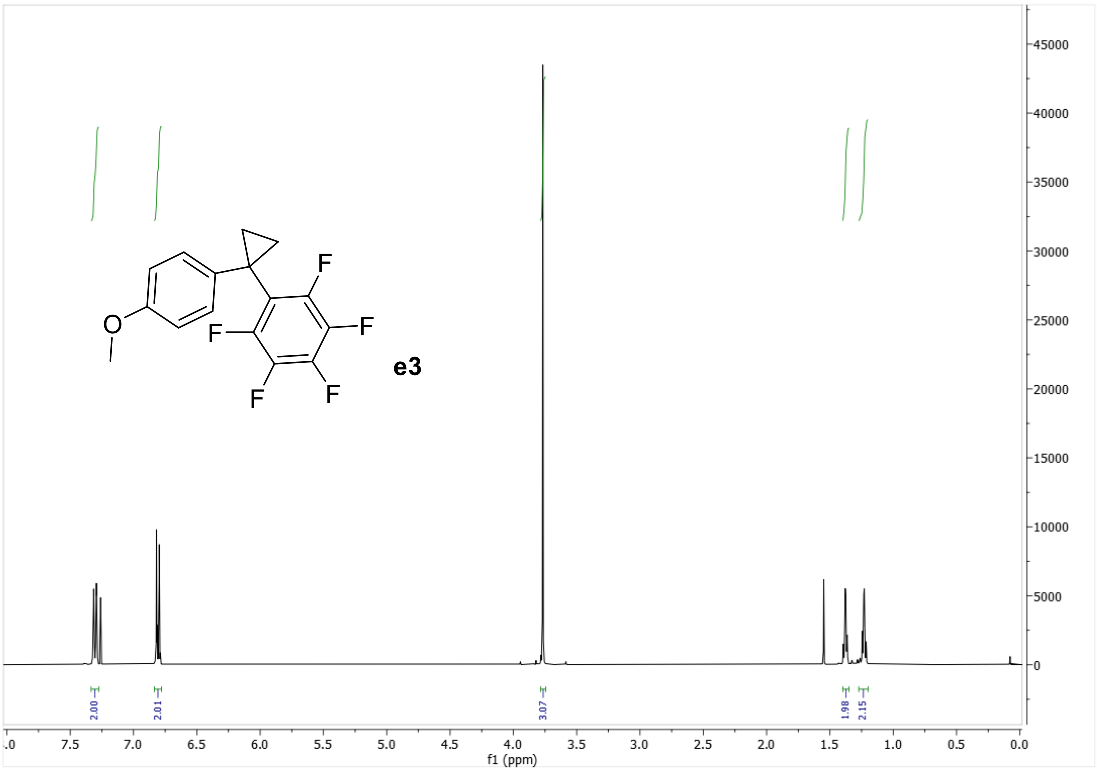

^{1}H-NMR spectrum of (**e3**) (400 MHz, $CDCl_3$)

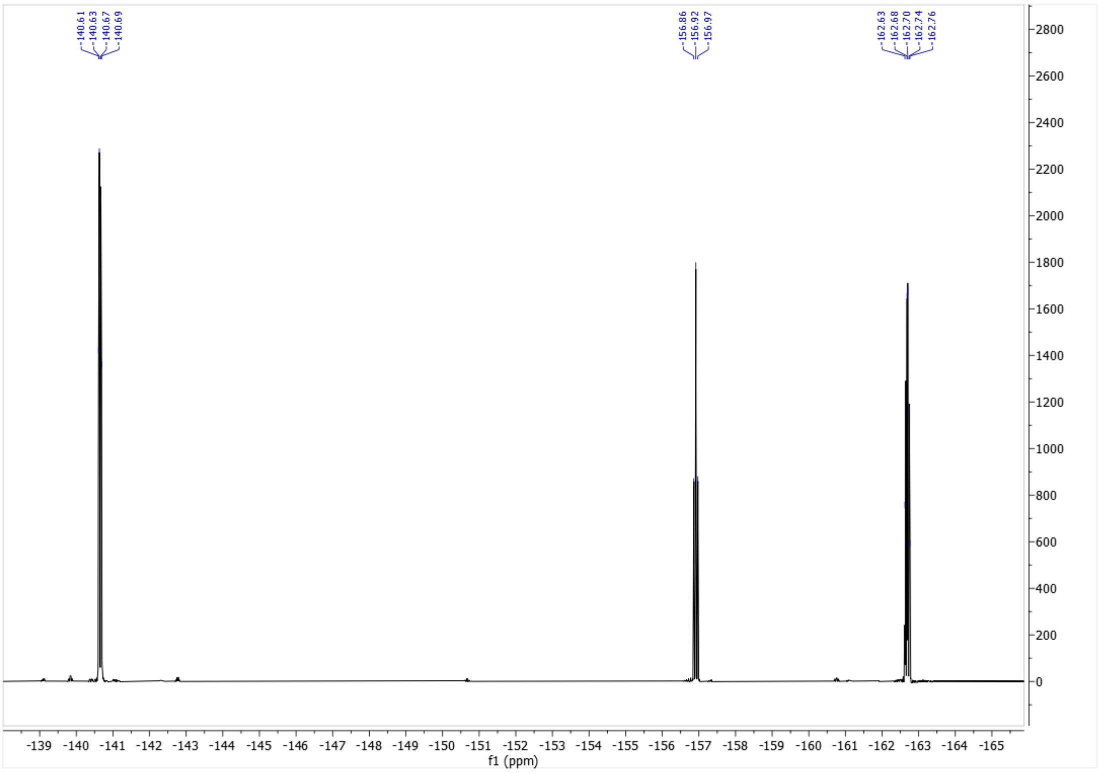

^{19}F-NMR spectrum of (**e3**) (400 MHz, $CDCl_3$)

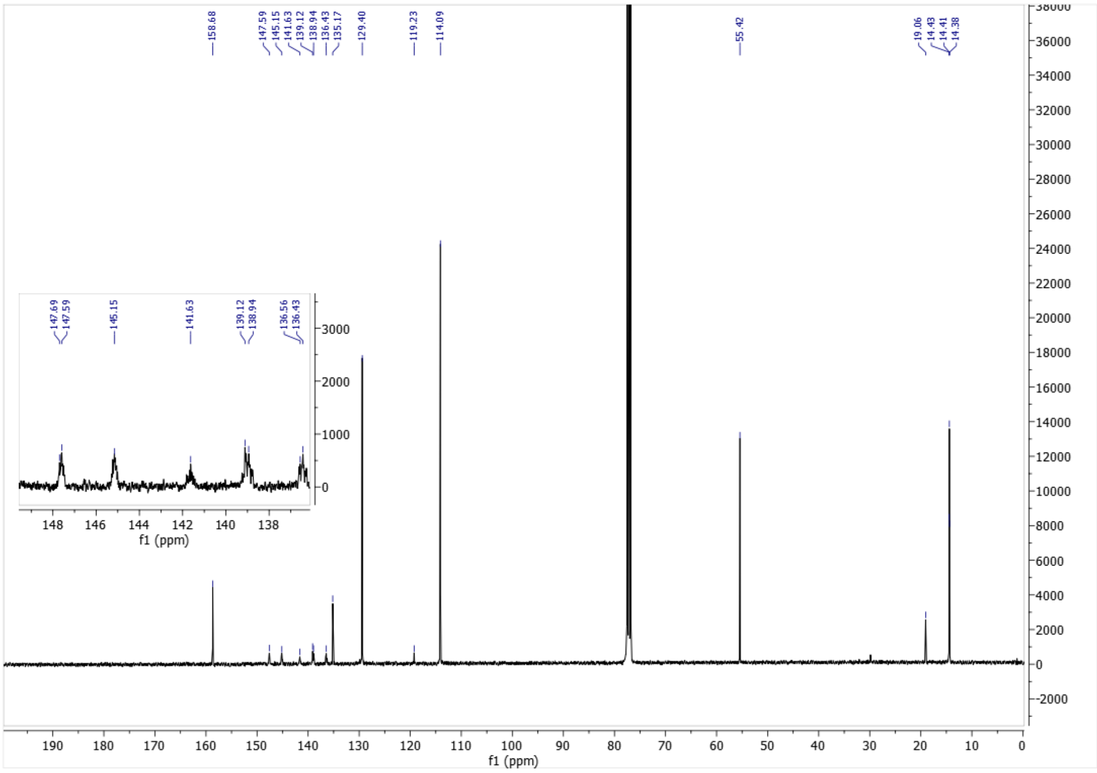

^{12}C-NMR spectrum of (**e3**) (400 MHz, $CDCl_3$)

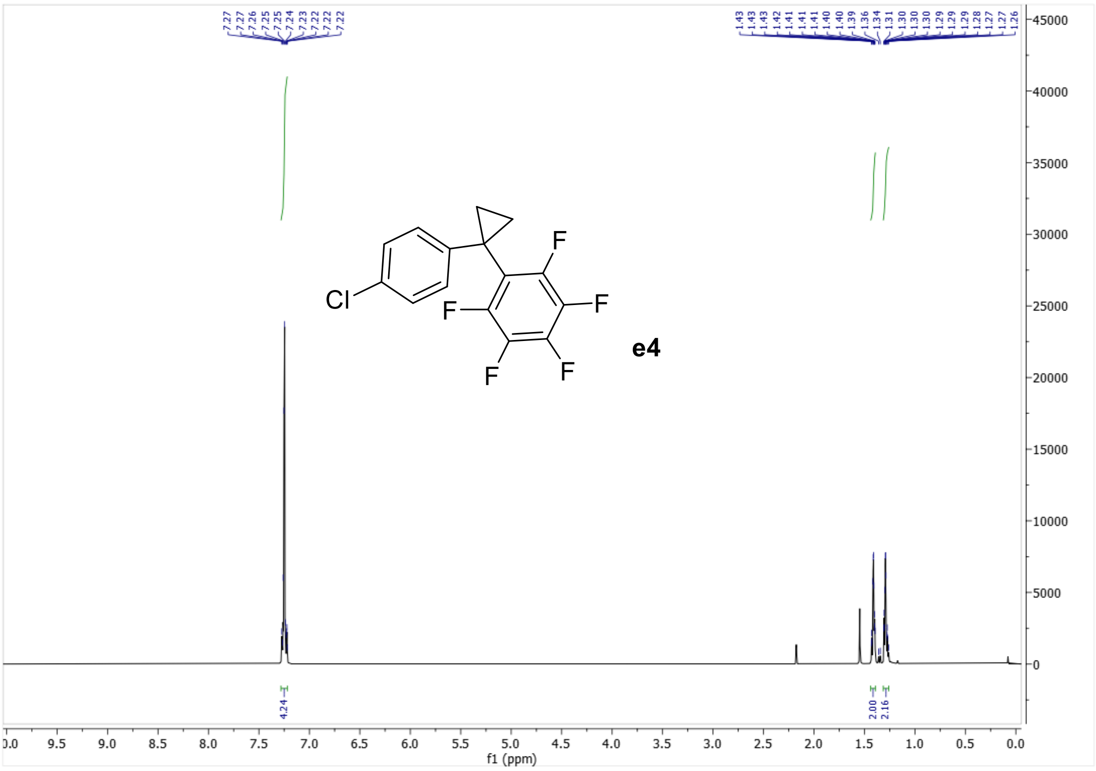

^{1}H-NMR spectrum of (**e4**) (400 MHz, $CDCl_3$)

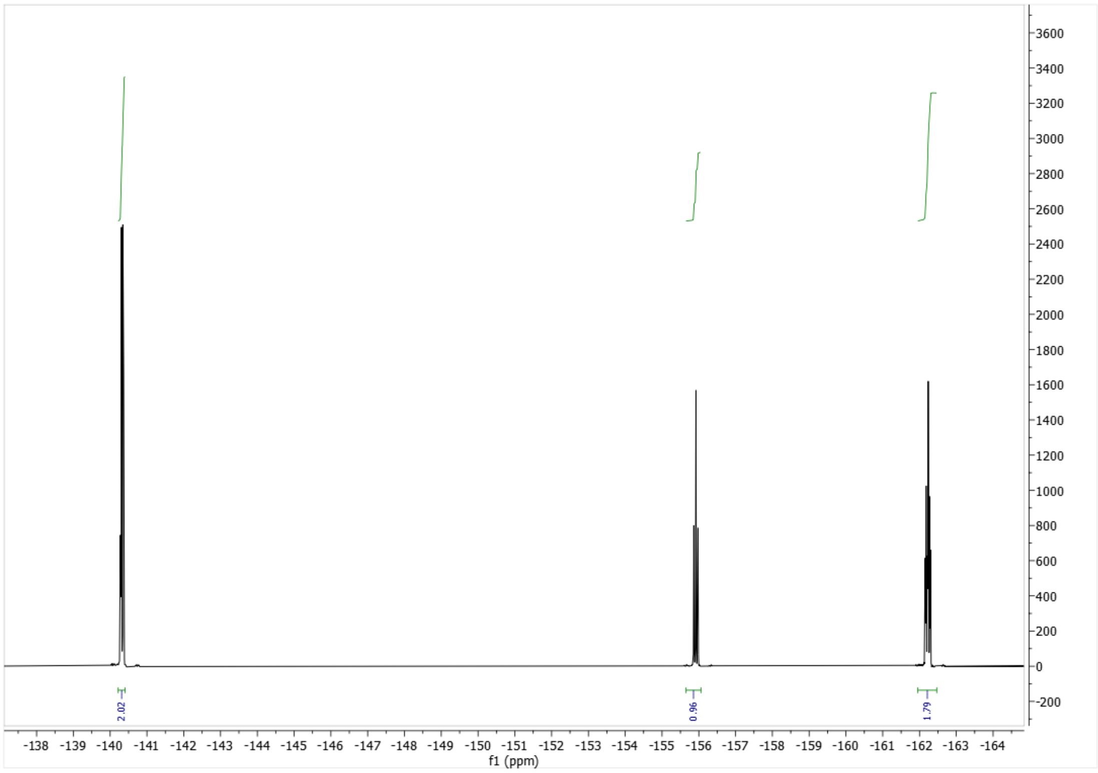

^{19}F-NMR spectrum of (**e4**) (400 MHz, $CDCl_3$)

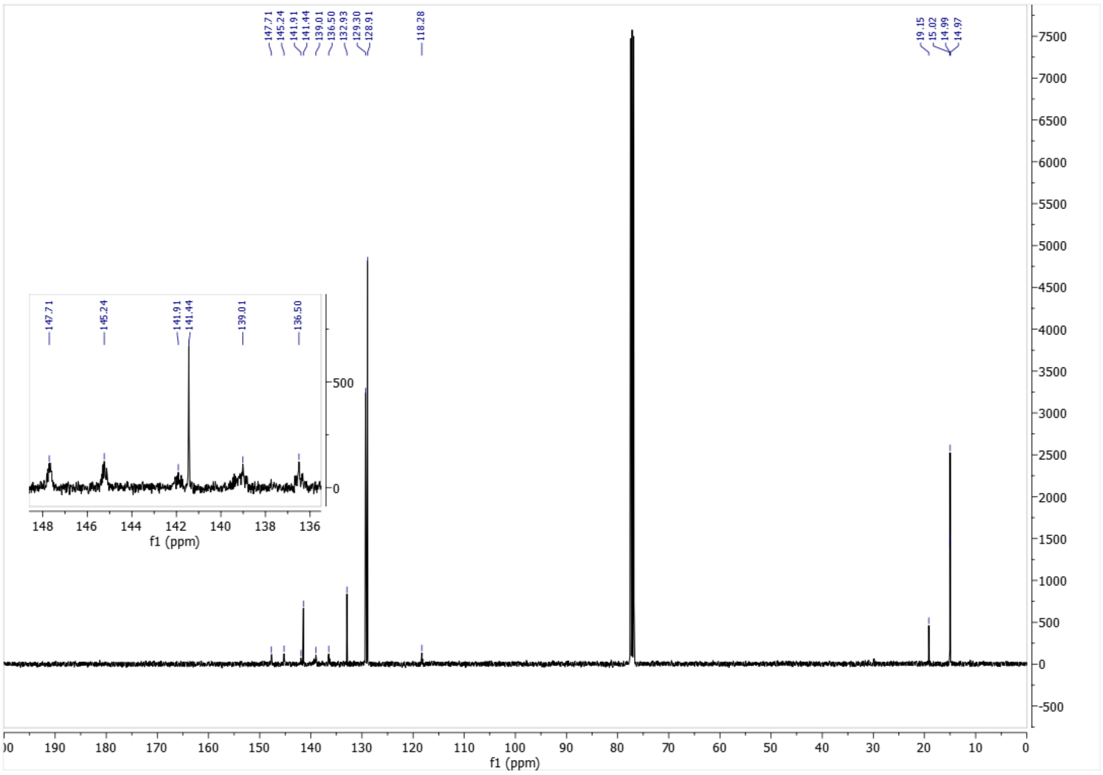

^{13}C-NMR spectrum of (**e4**) (400 MHz, $CDCl_3$)

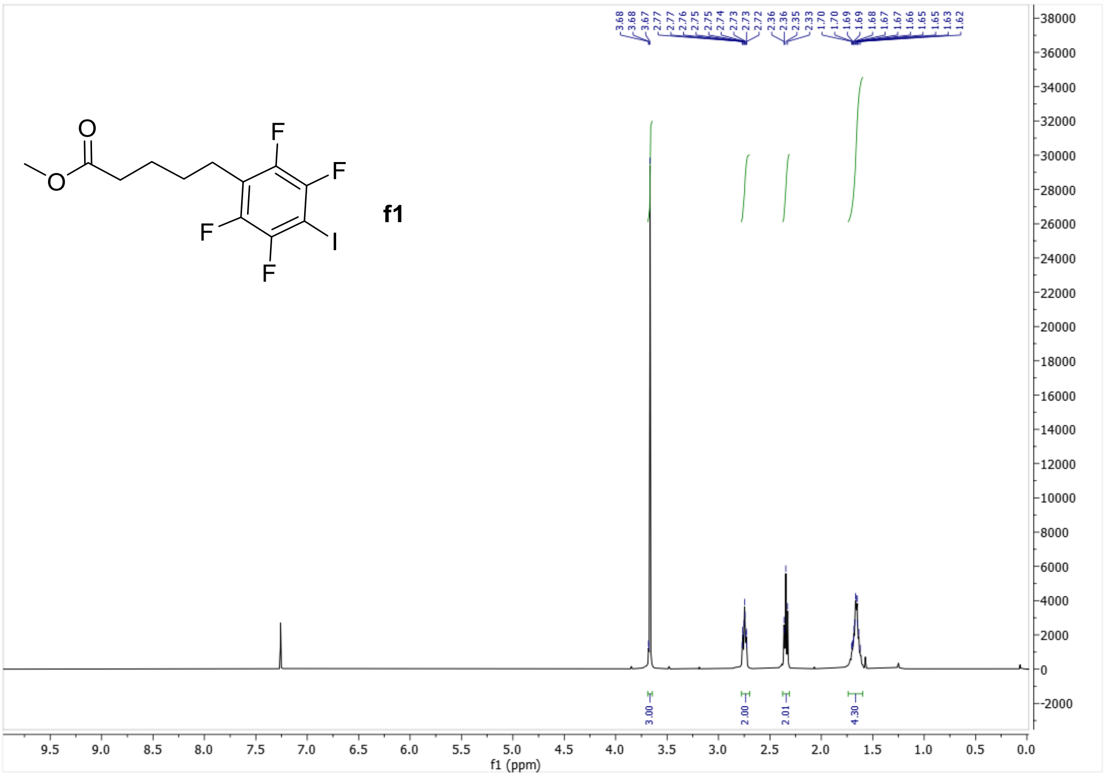

^{1}H-NMR spectrum of (**f1**) (400 MHz, $CDCl_3$)

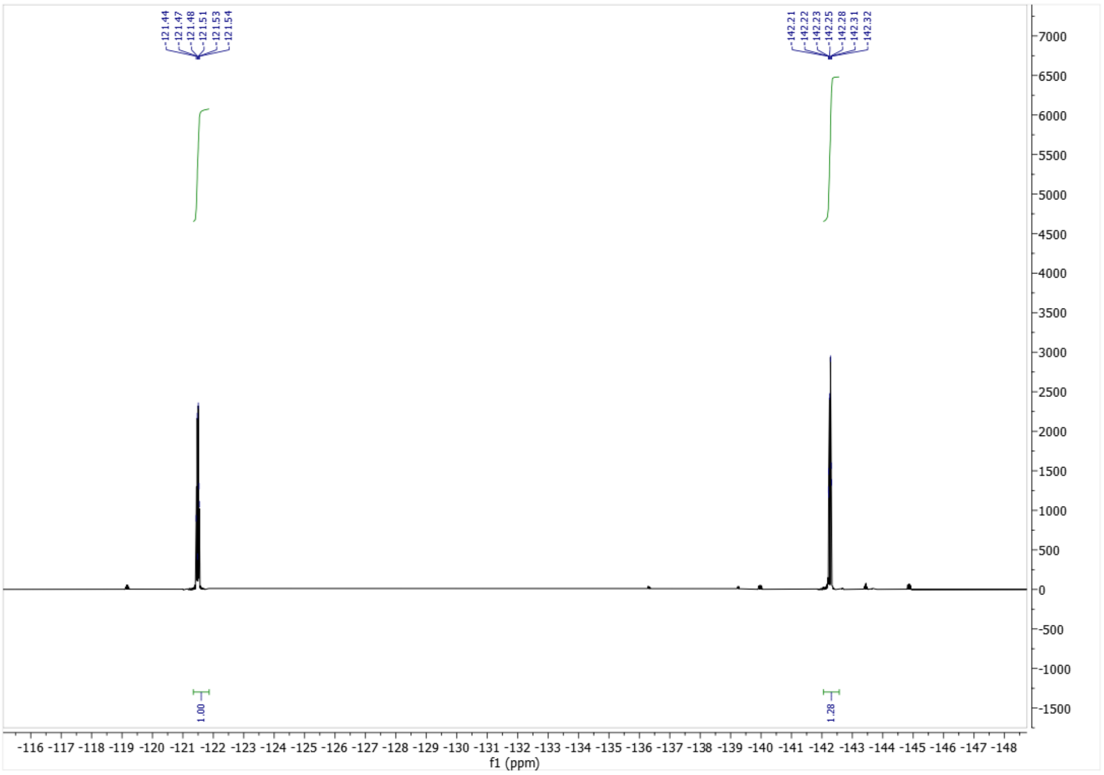

^{19}F-NMR spectrum of (**f1**) (400 MHz, $CDCl_3$)

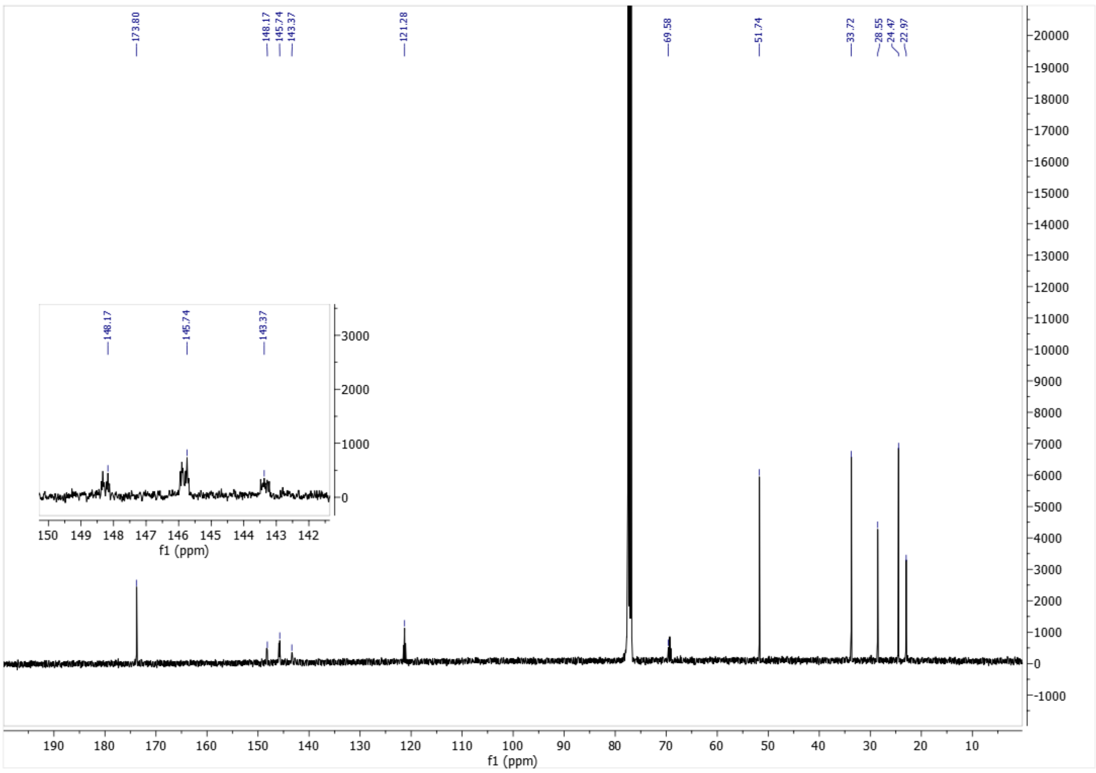

^{13}C-NMR spectrum of (**f1**) (400 MHz, $CDCl_3$)

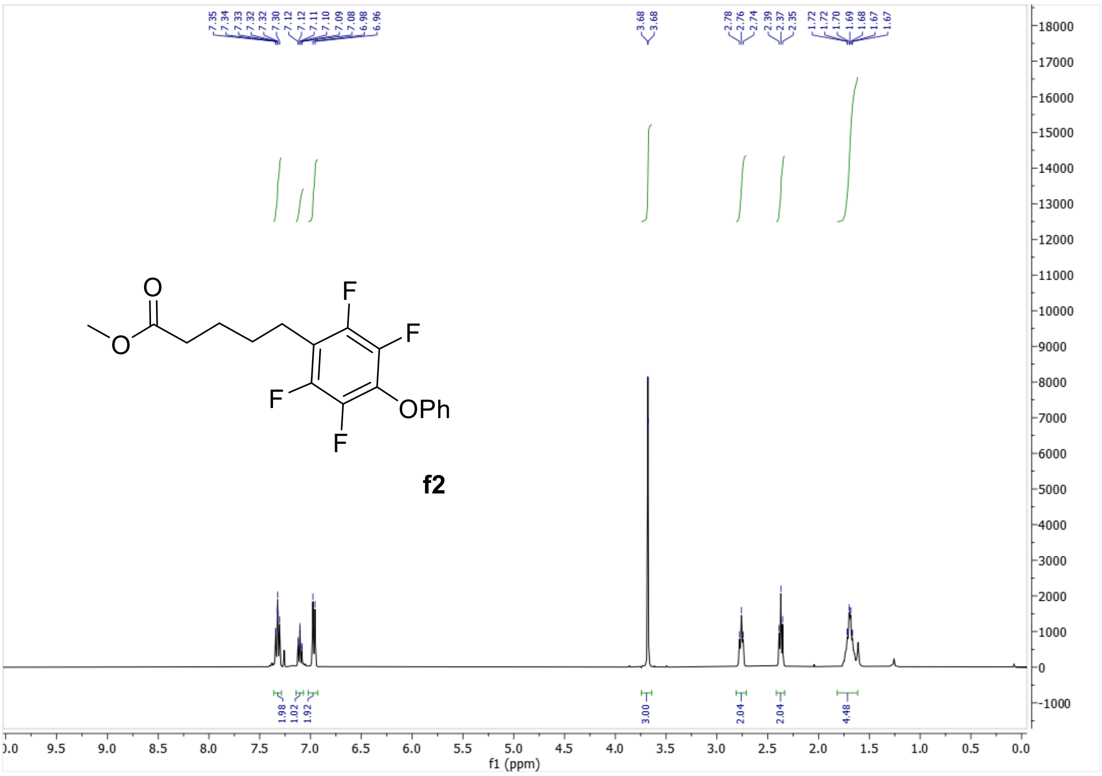

^{1}H-NMR spectrum of (**f2**) (400 MHz, $CDCl_3$)

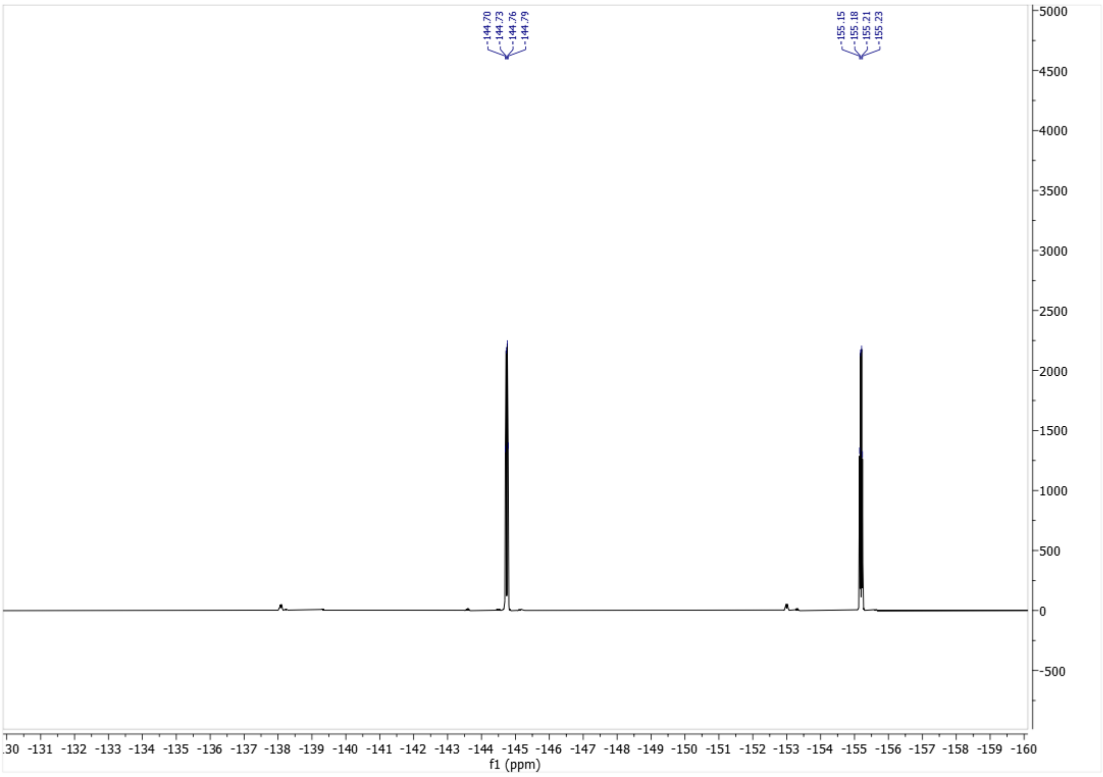

^{19}F-NMR spectrum of (**f2**) (400 MHz, $CDCl_3$)

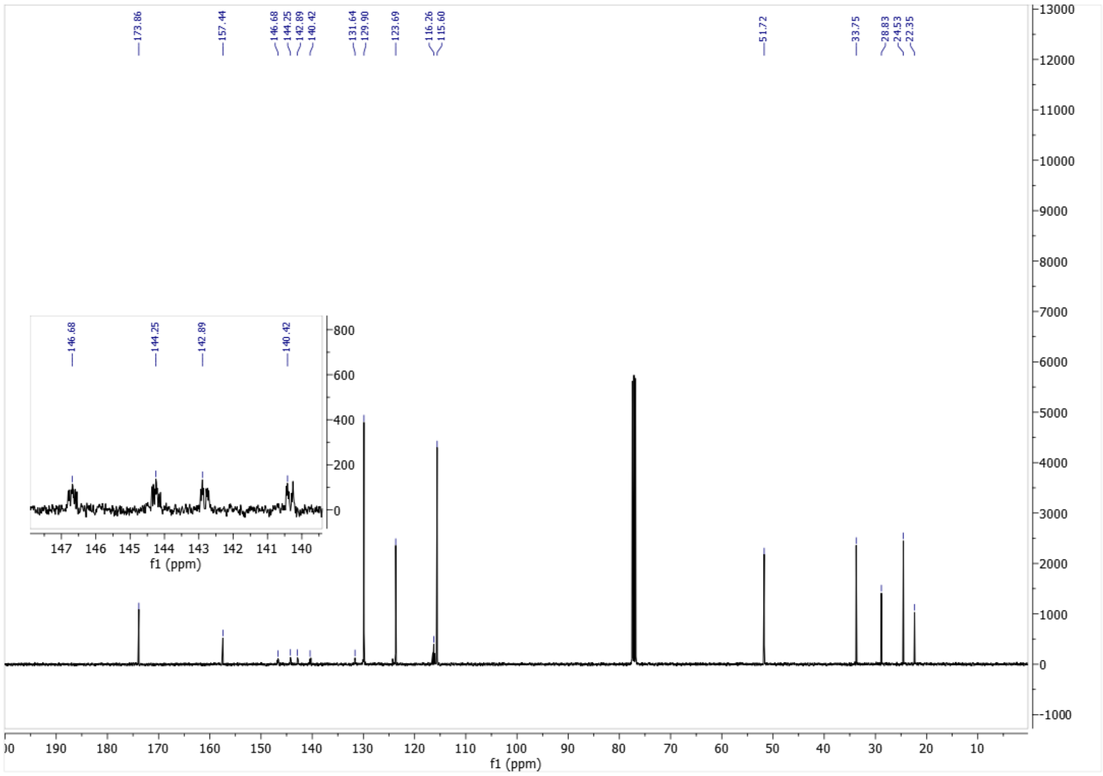

^{13}C-NMR spectrum of (**f2**) (400 MHz, $CDCl_3$)

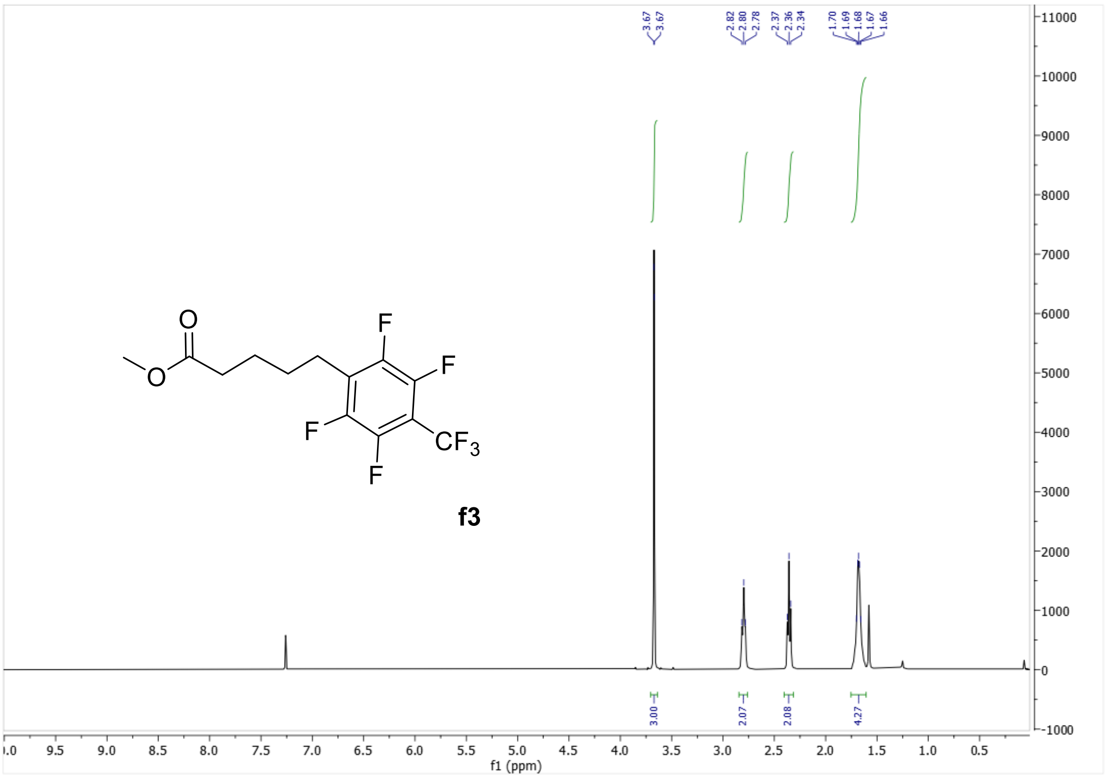

^{1}H-NMR spectrum of (**f3**) (400 MHz, $CDCl_3$)

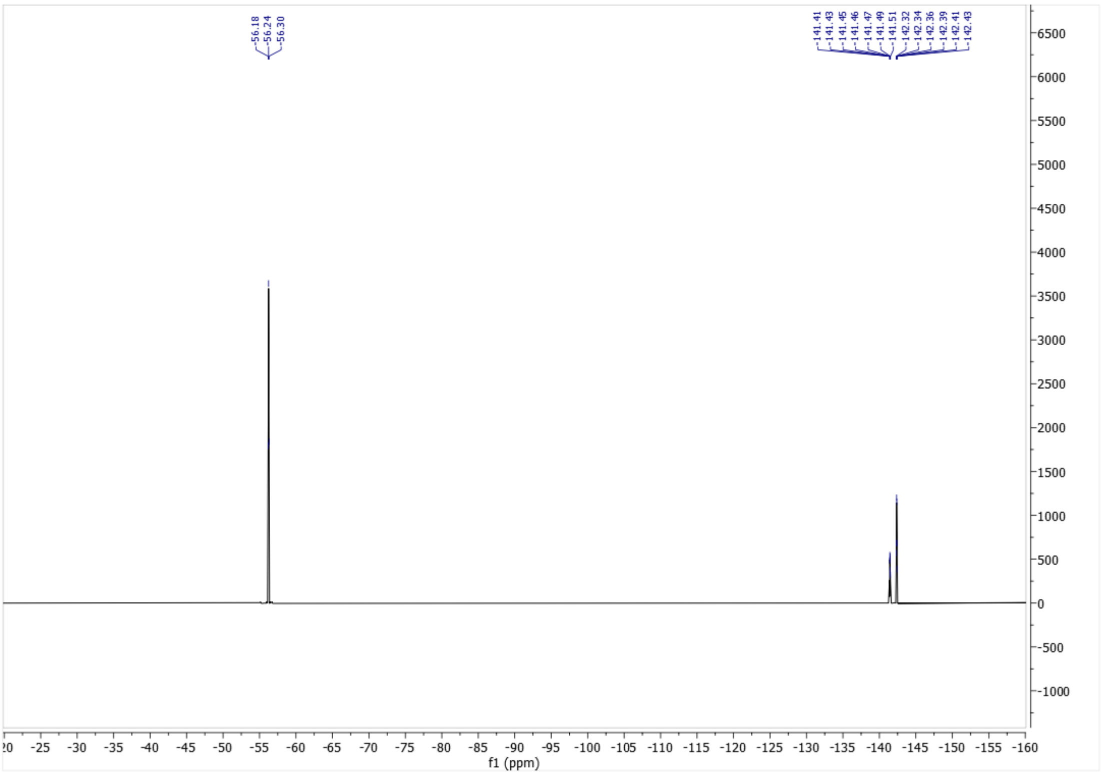

^{19}F-NMR spectrum of (**f3**) (400 MHz, $CDCl_3$)

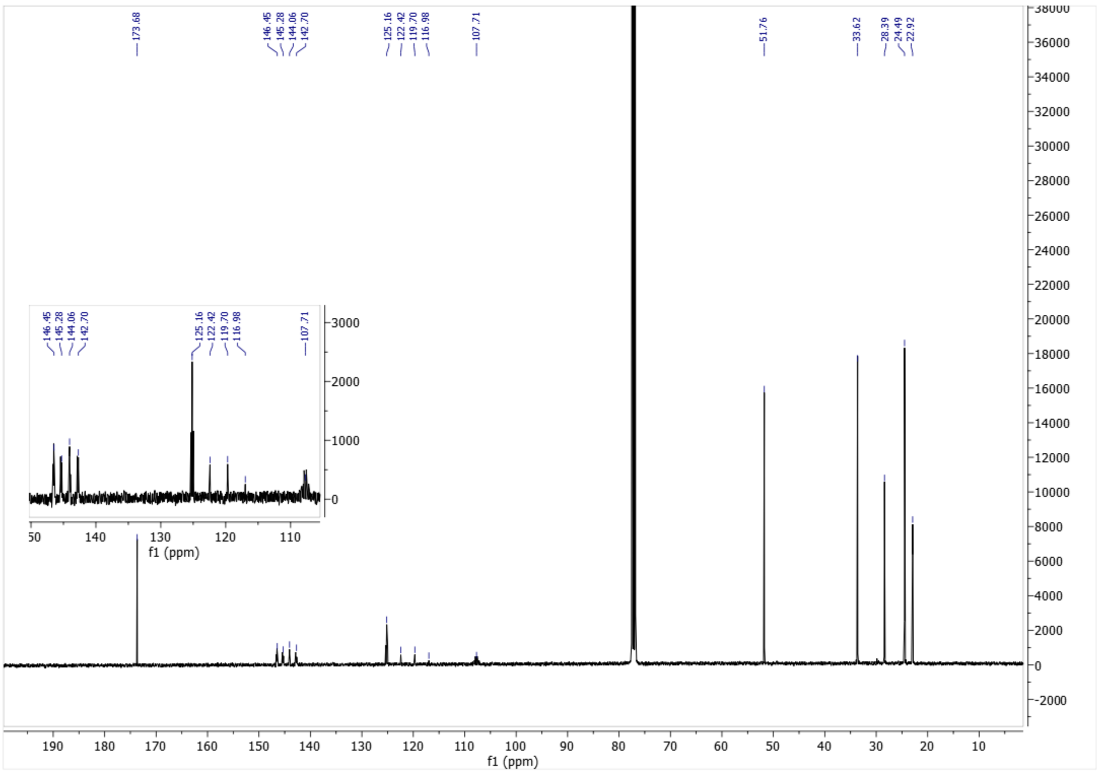

^{13}C-NMR spectrum of (**f3**) (400 MHz, $CDCl_3$)

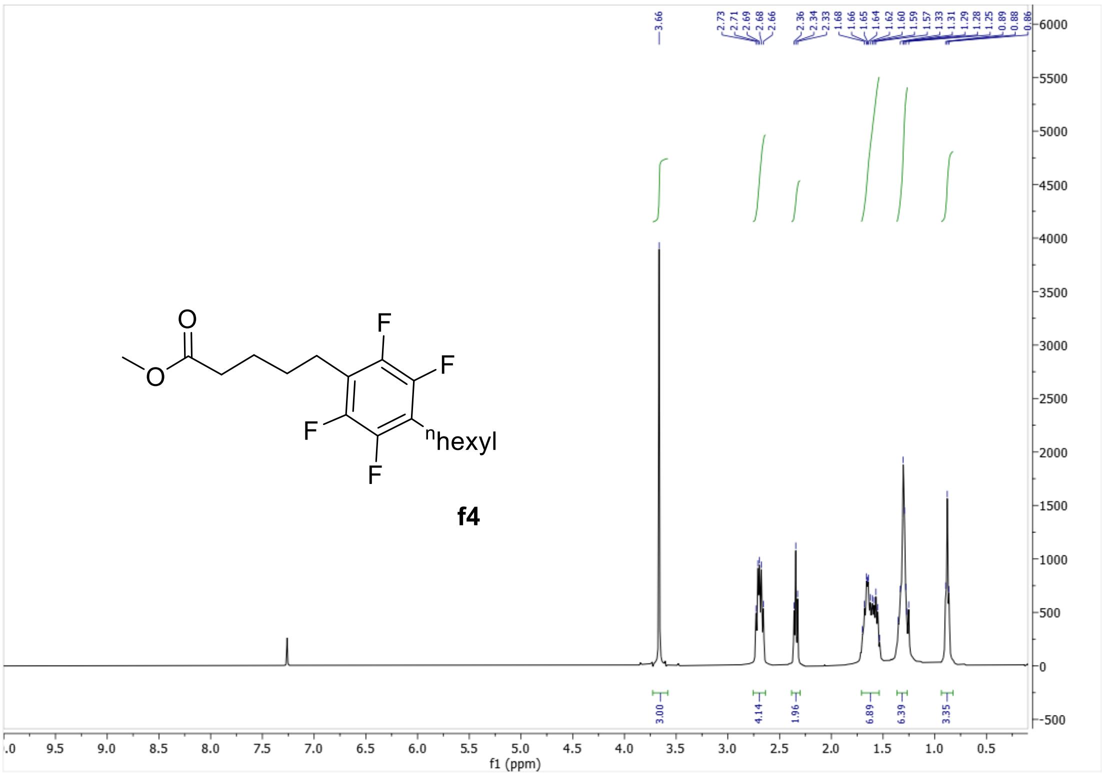

^{1}H-NMR spectrum of (**f4**) (400 MHz, $CDCl_3$)

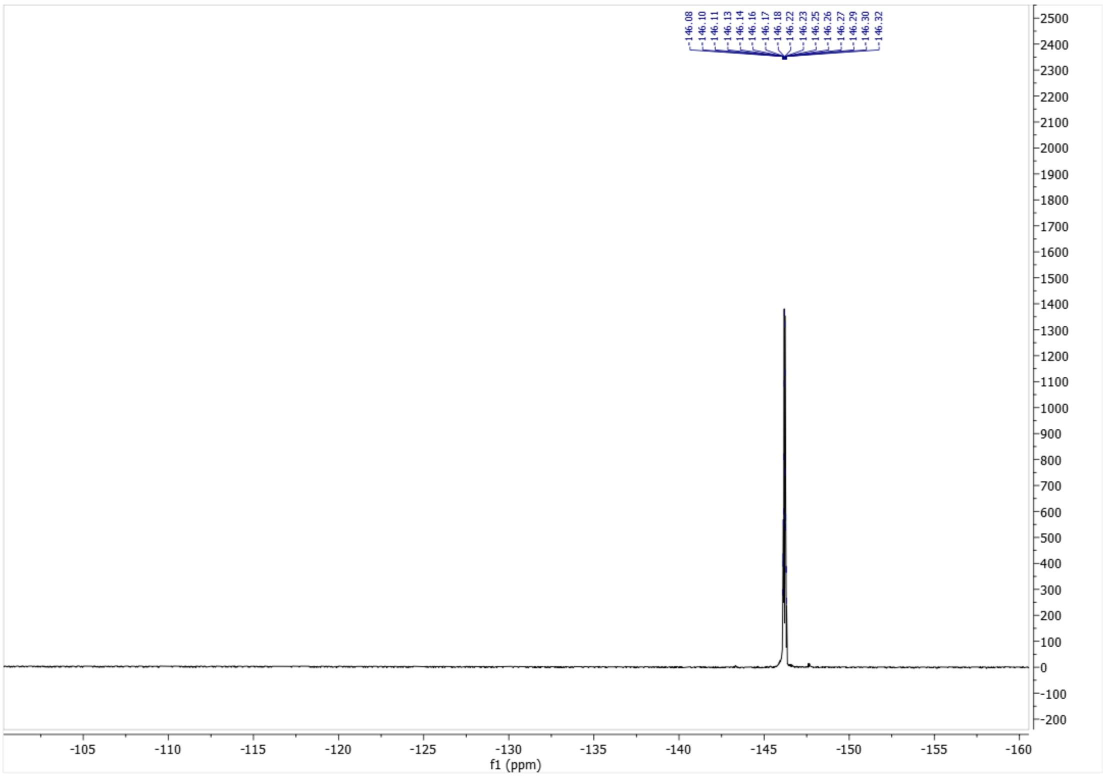

^{19}F-NMR spectrum of (**f4**) (400 MHz, $CDCl_3$)

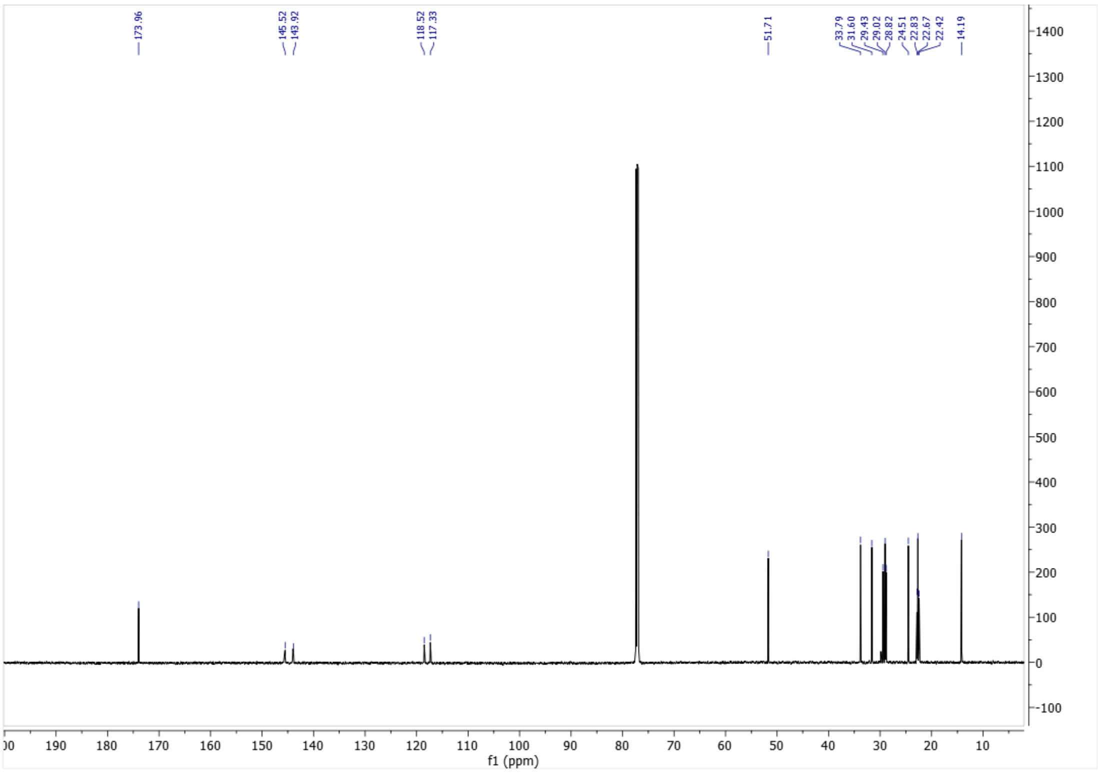

^{13}C-NMR spectrum of (**f4**) (400 MHz, $CDCl_3$)

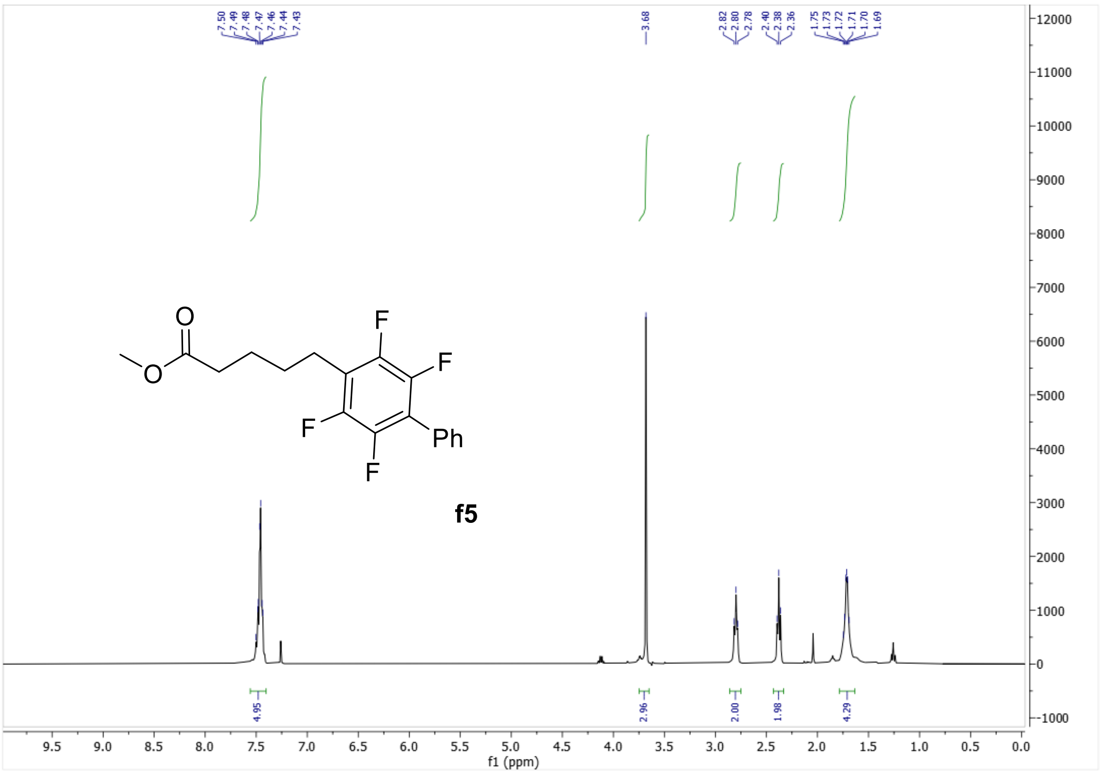

^{1}H-NMR spectrum of (**f5**) (400 MHz, $CDCl_3$)

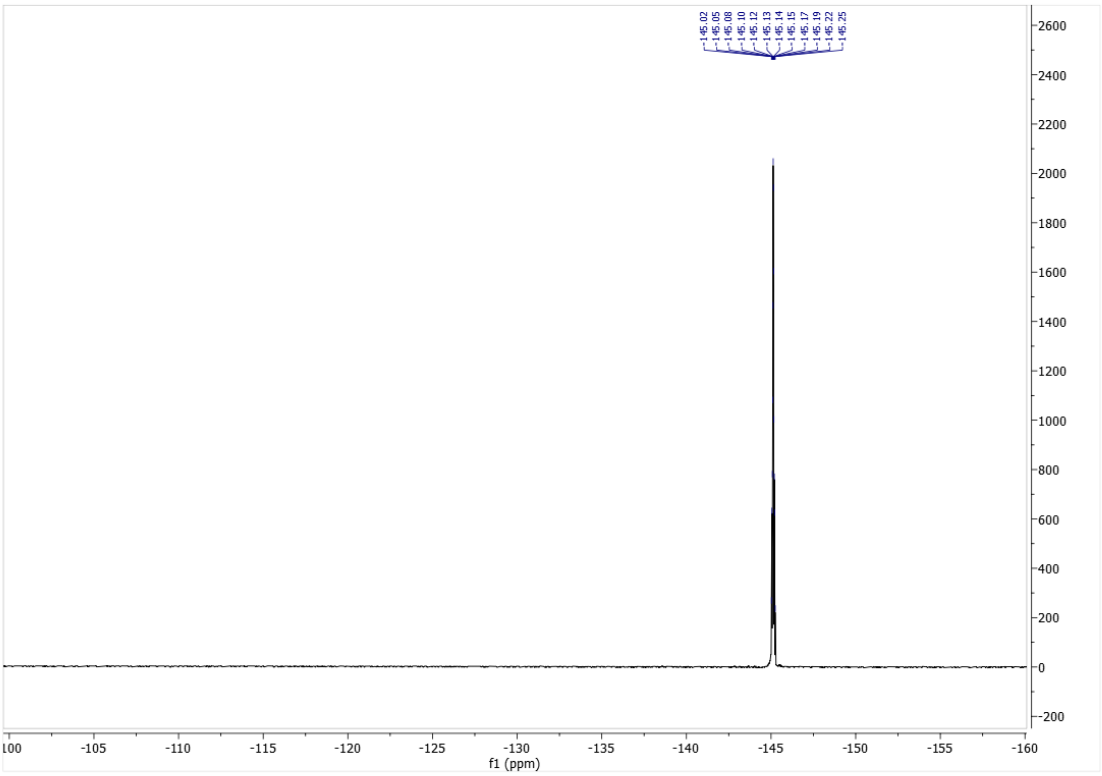

^{19}F-NMR spectrum of (**f5**) (400 MHz, $CDCl_3$)

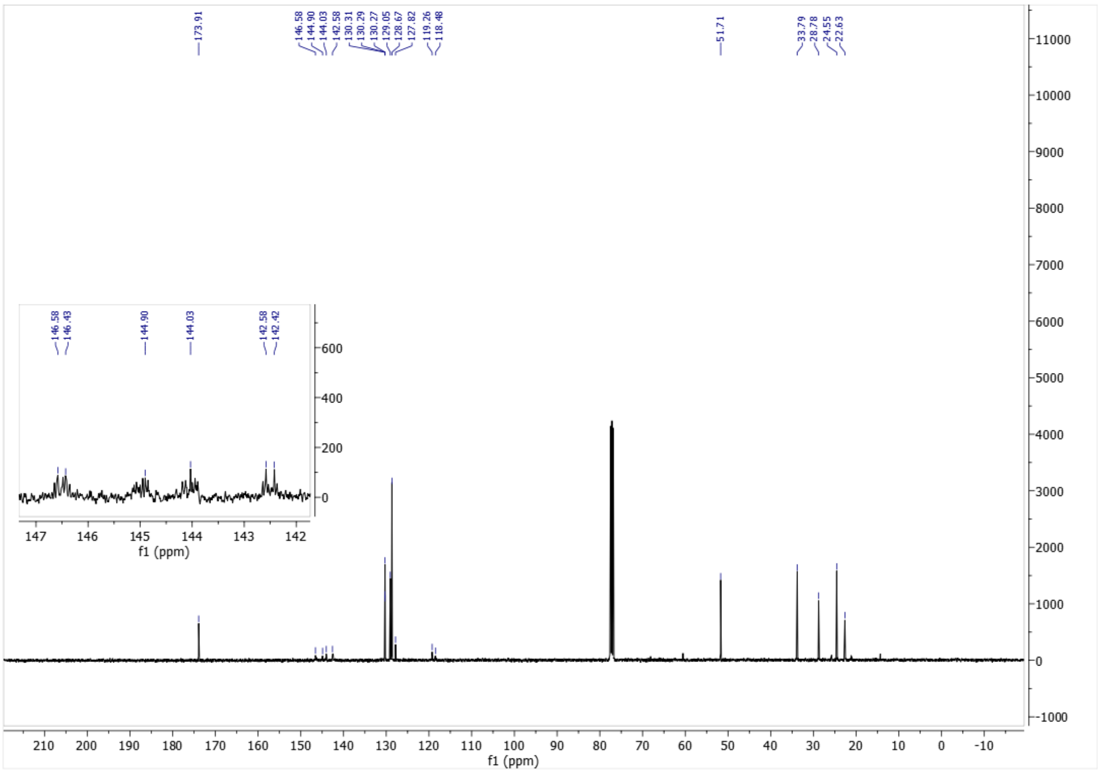

^{13}C-NMR spectrum of (**f5**) (400 MHz, $CDCl_3$)

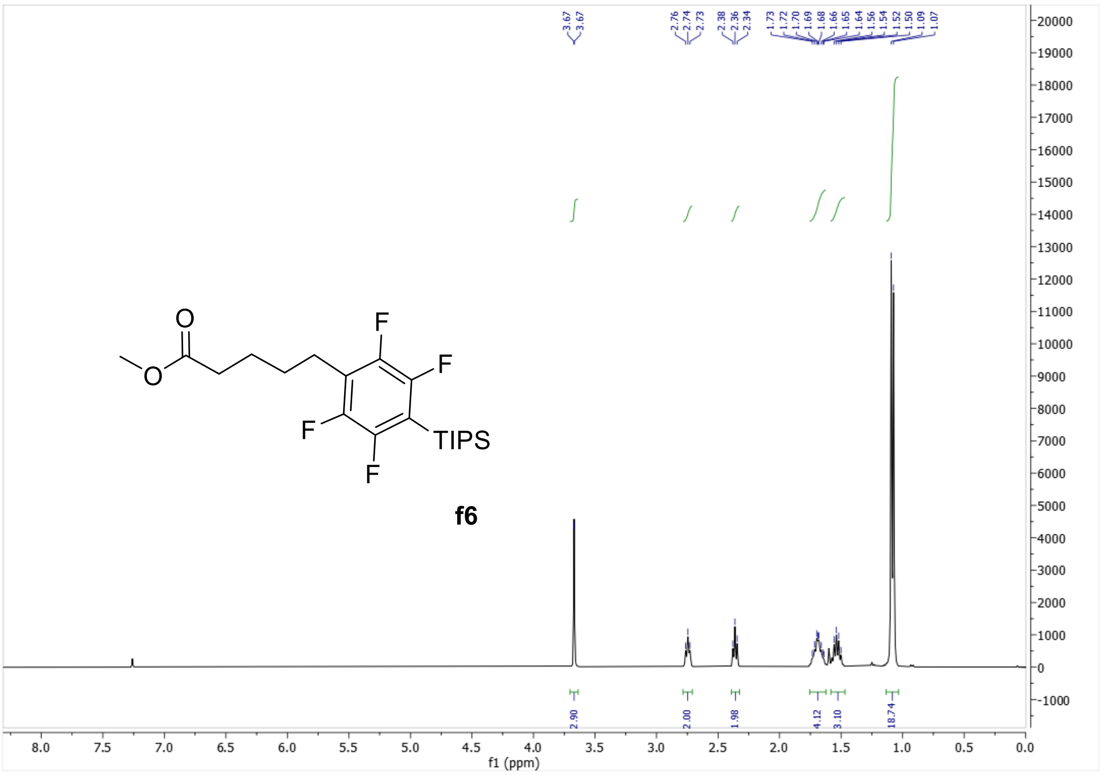

^{1}H-NMR spectrum of (**f6**) (400 MHz, $CDCl_3$)

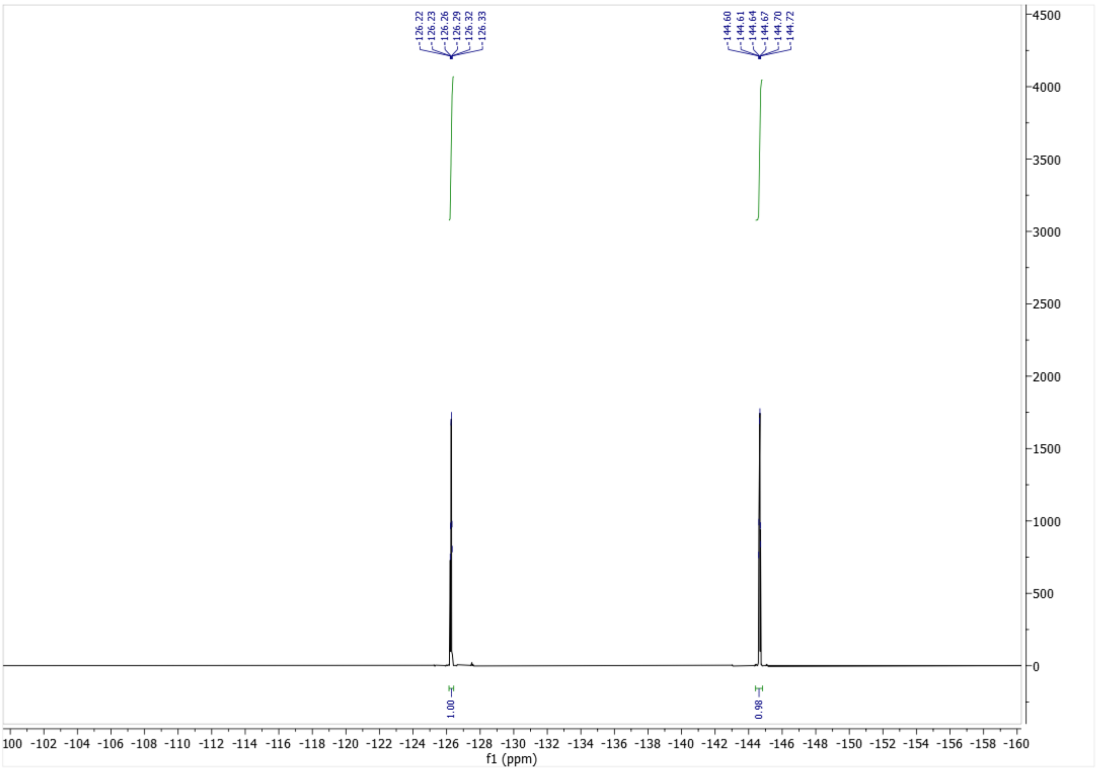

^{19}F-NMR spectrum of (**f6**) (400 MHz, $CDCl_3$)

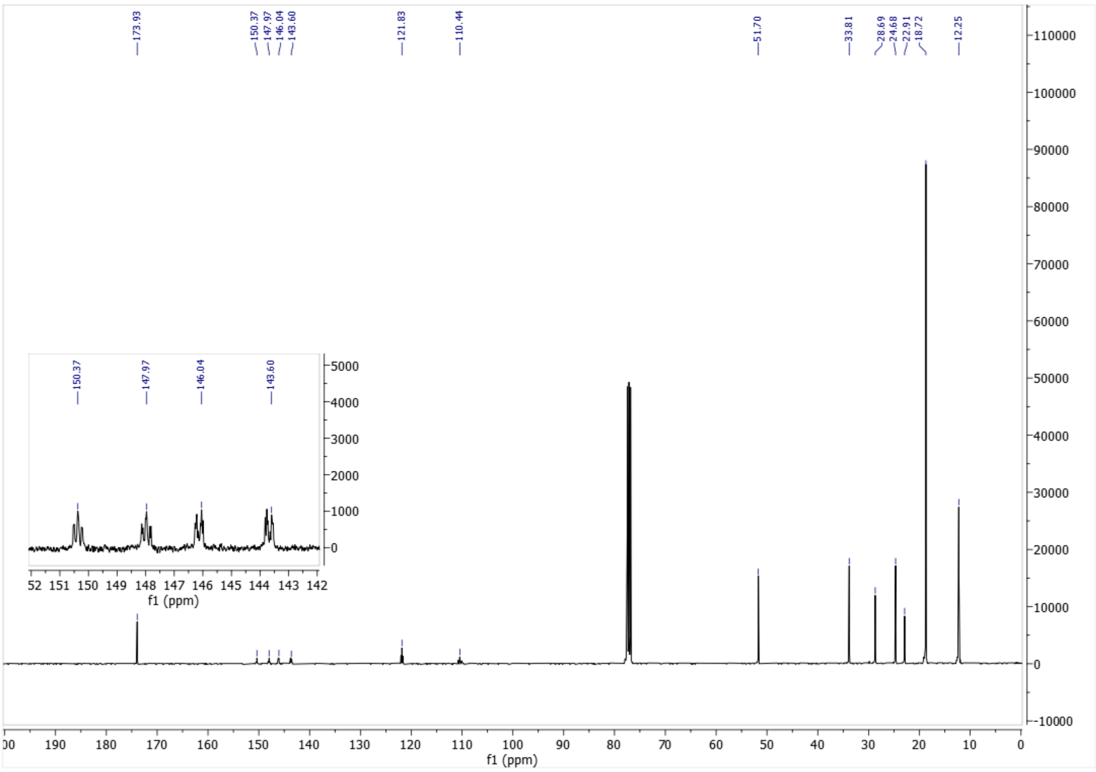

^{13}C-NMR spectrum of (**f6**) (400 MHz, $CDCl_3$)

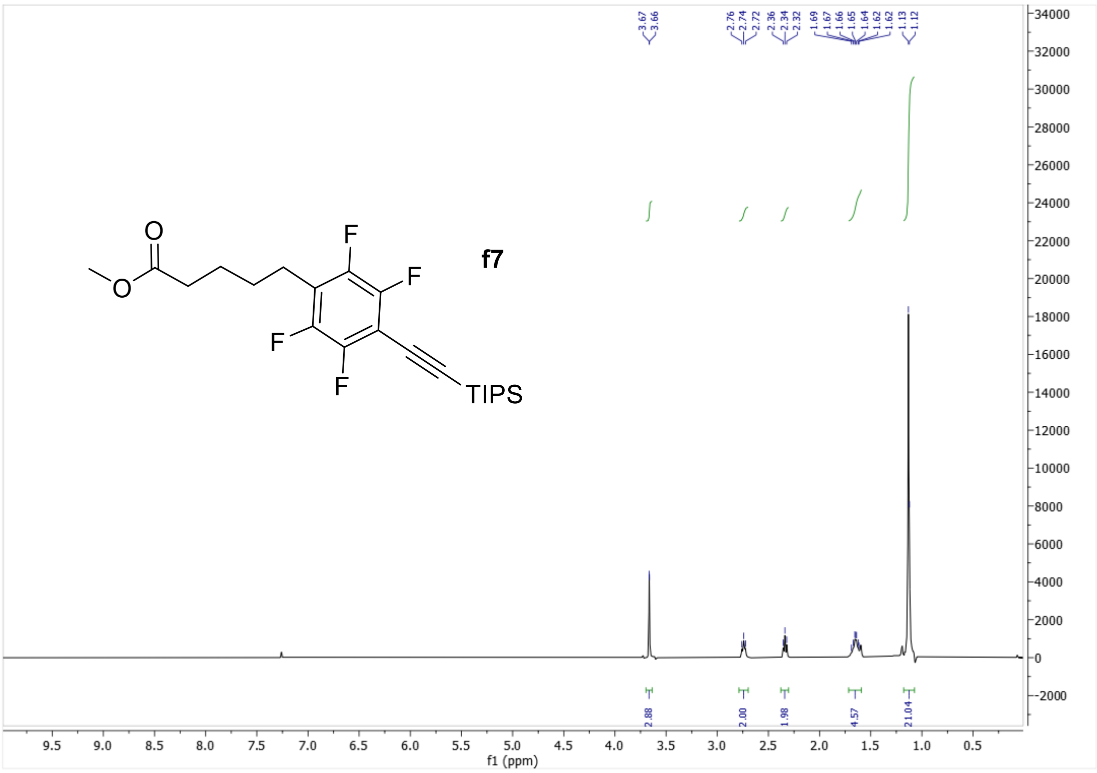

^{1}H-NMR spectrum of (**f7**) (400 MHz, $CDCl_3$)

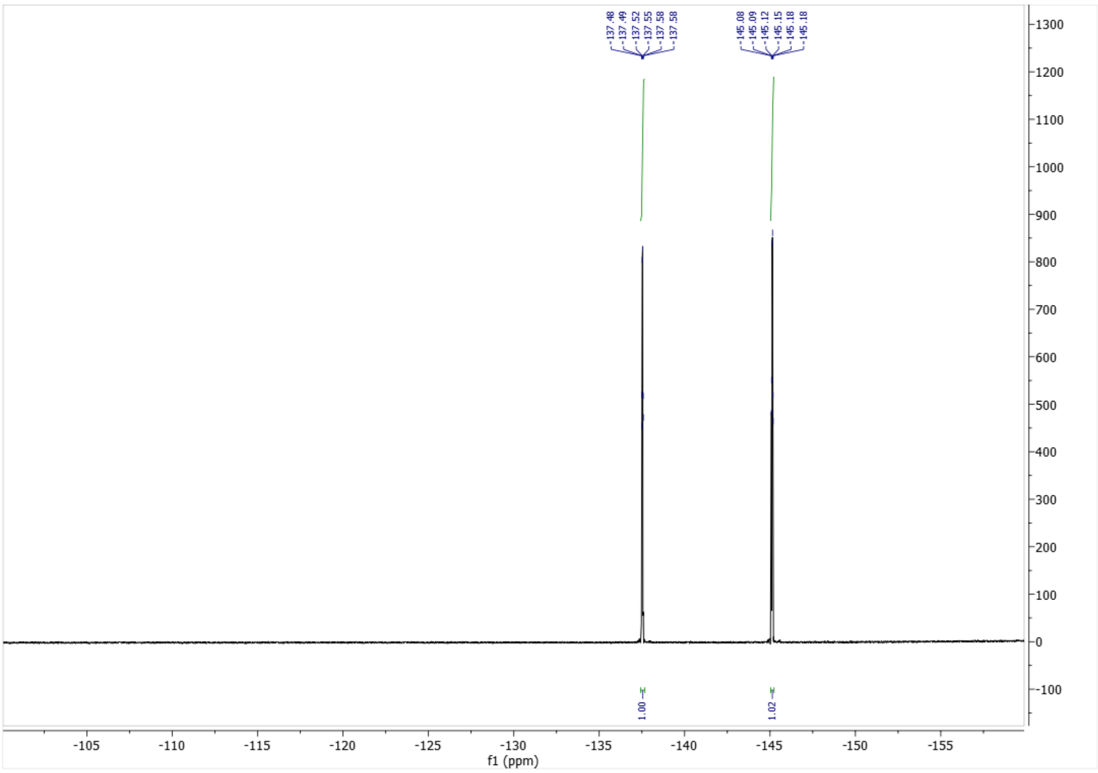

^{19}F-NMR spectrum of (**f7**) (400 MHz, $CDCl_3$)

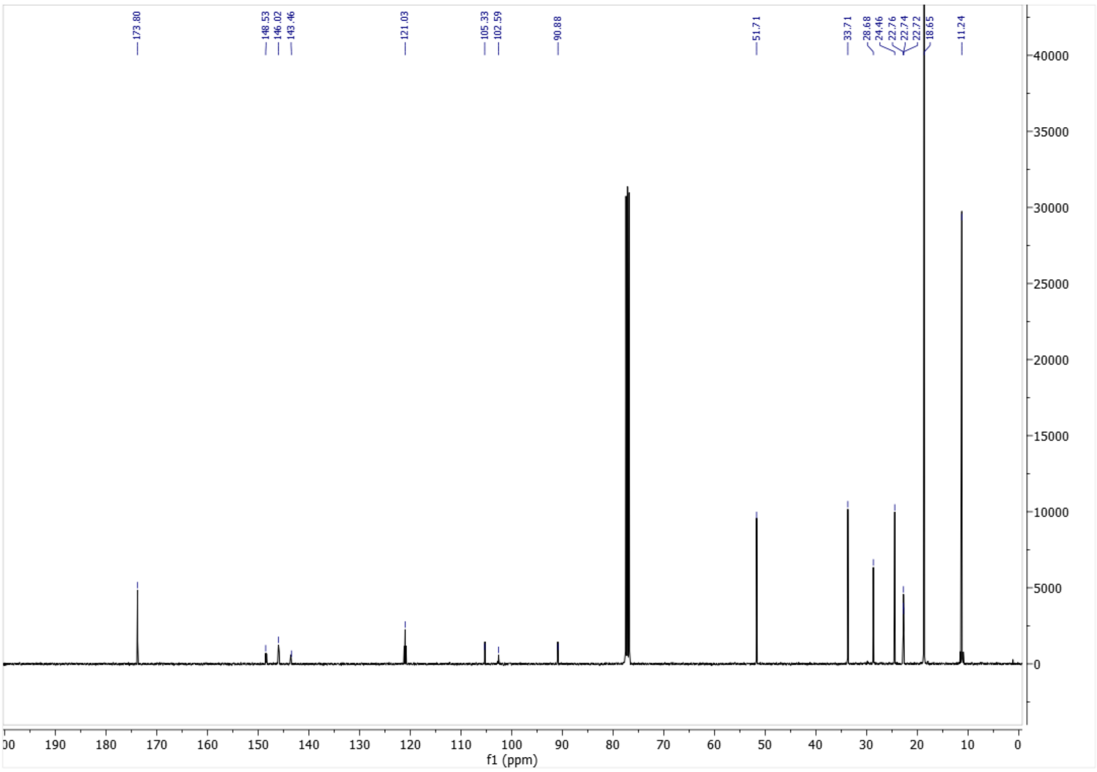

^{13}C-NMR spectrum of (**f7**) (400 MHz, $CDCl_3$)

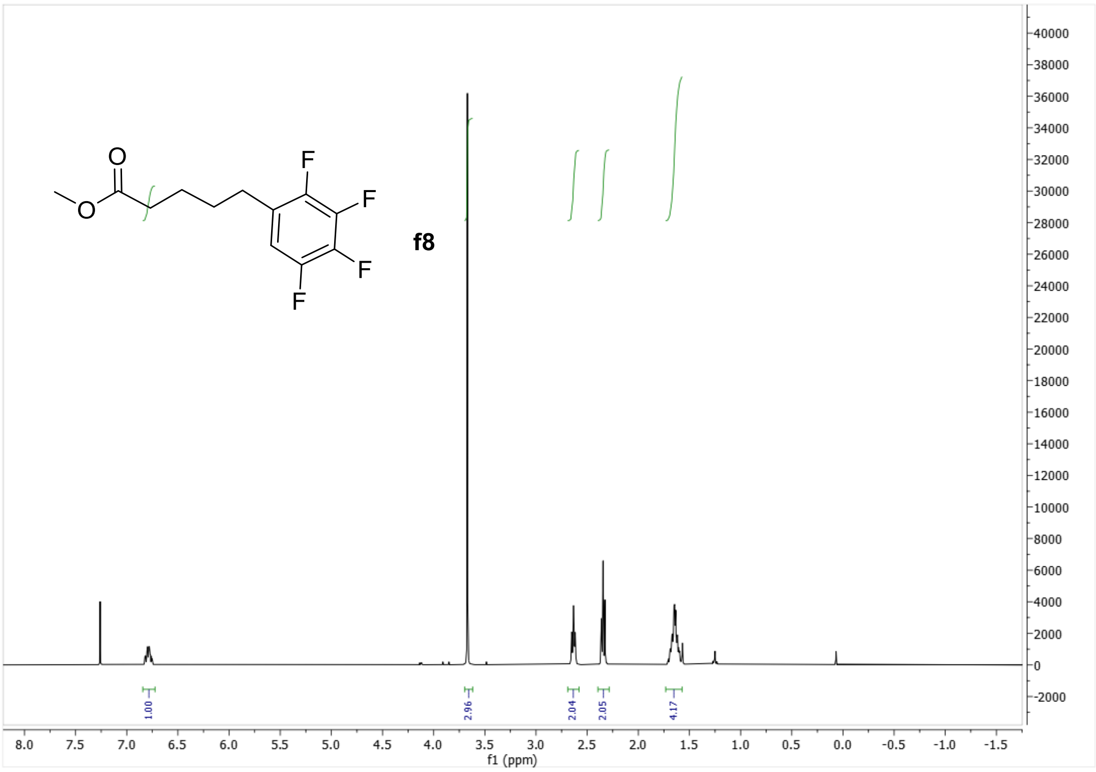

^{1}H-NMR spectrum of (**f8**) (400 MHz, $CDCl_3$)

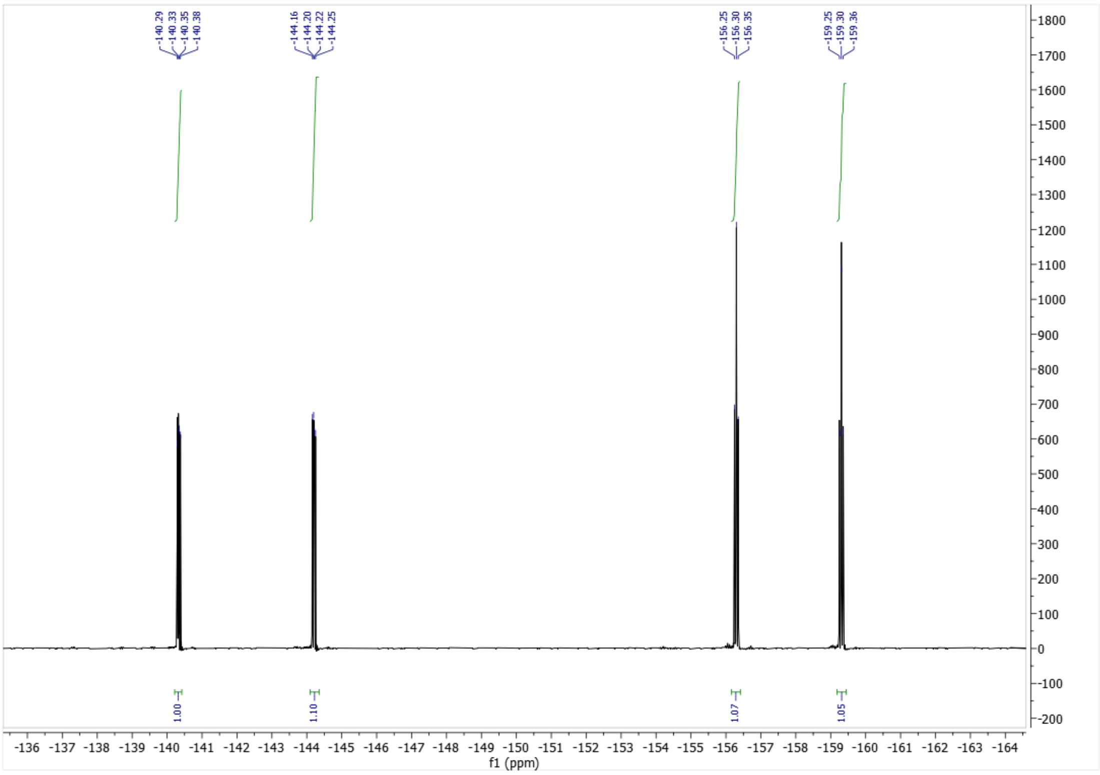

^{19}F-NMR spectrum of (**f8**) (400 MHz, $CDCl_3$)

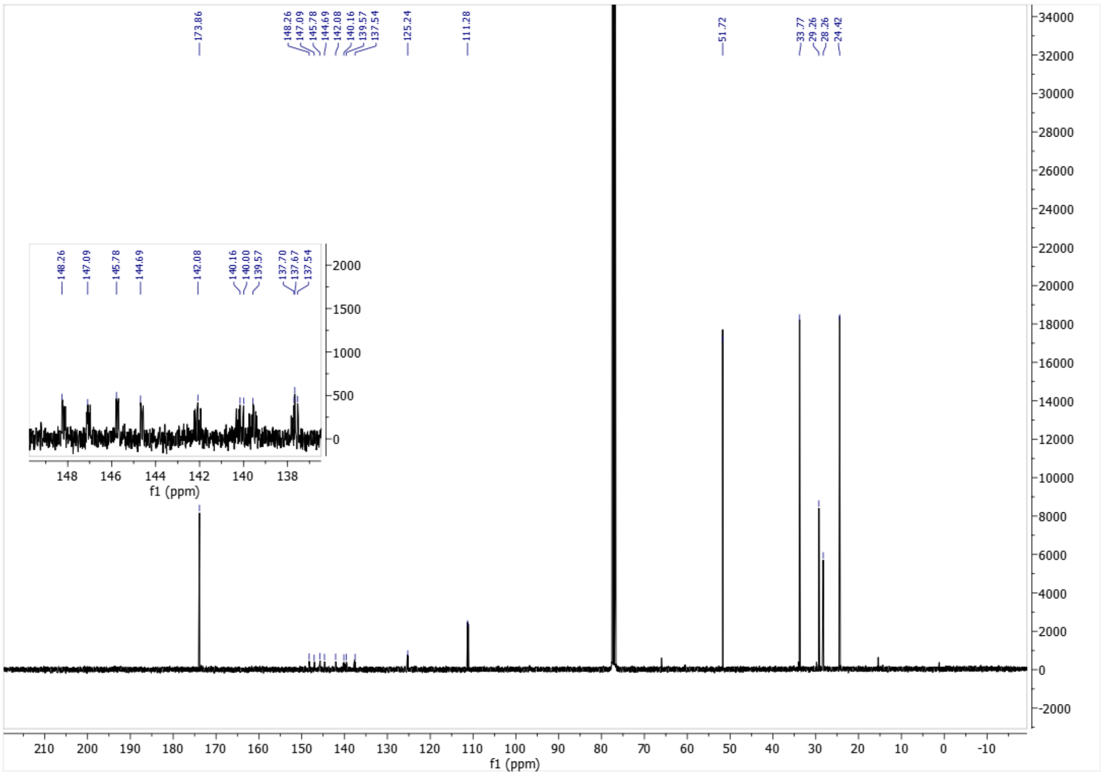

^{13}C-NMR spectrum of (**f8**) (400 MHz, $CDCl_3$)

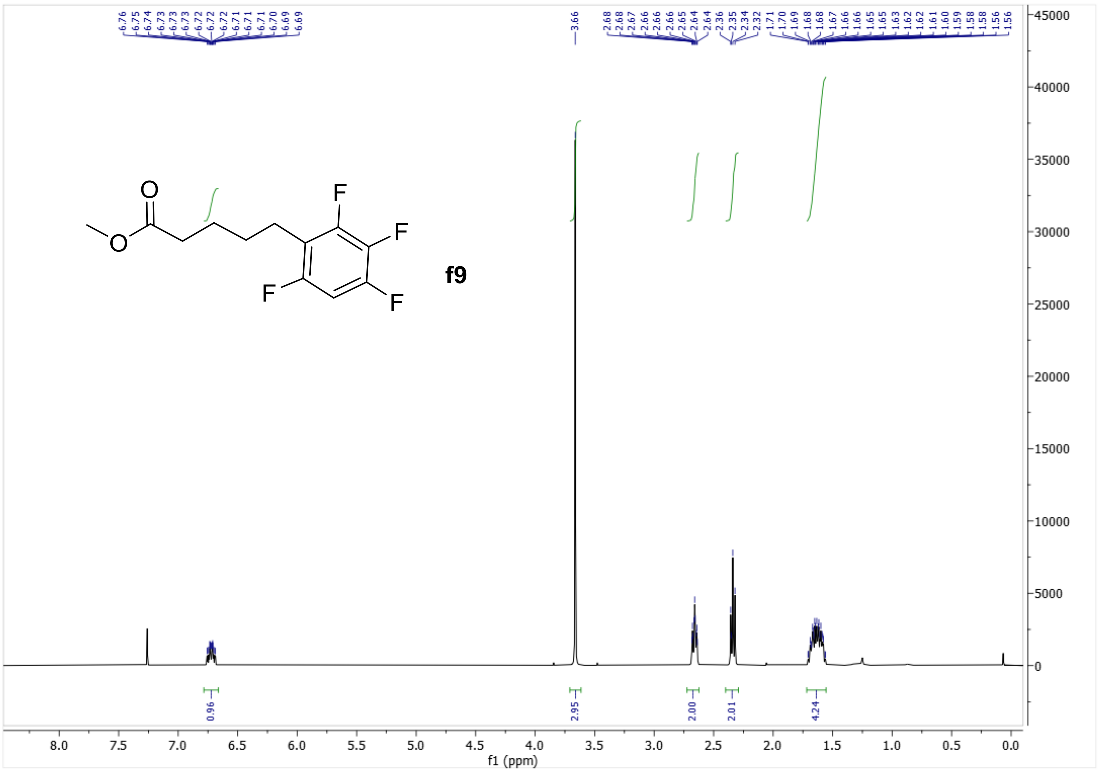

^{1}H-NMR spectrum of (**f9**) (400 MHz, $CDCl_3$)

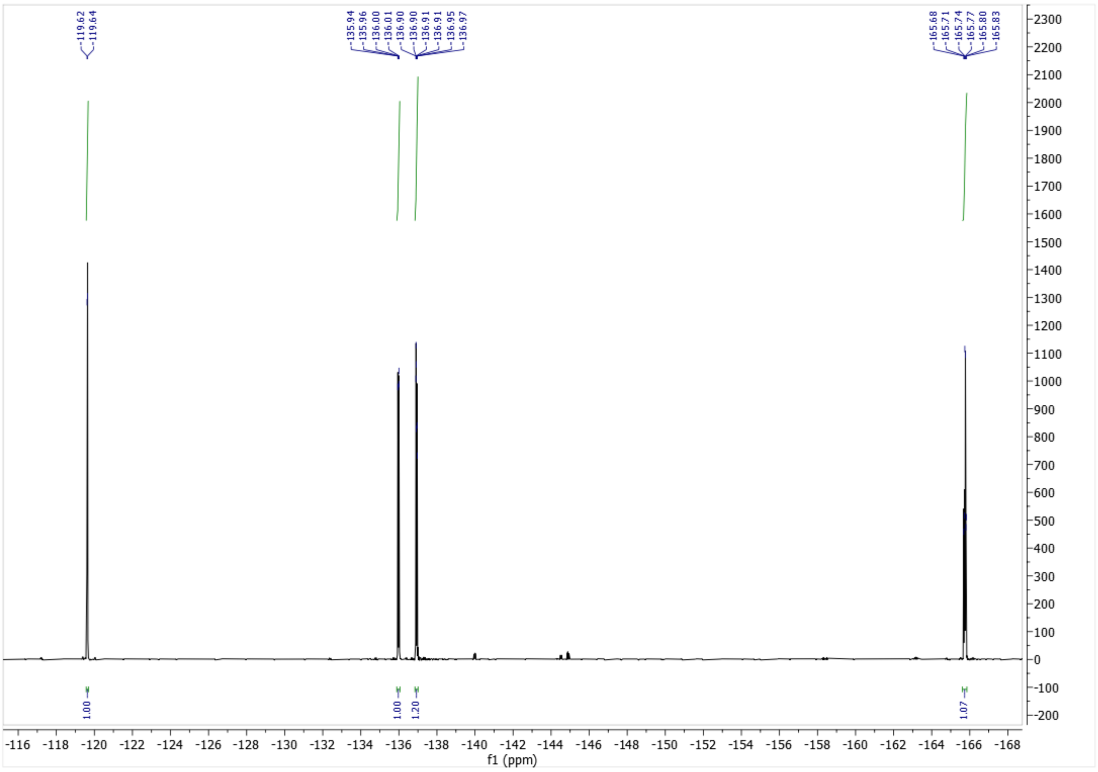

^{19}F-NMR spectrum of (**f9**) (400 MHz, $CDCl_3$)

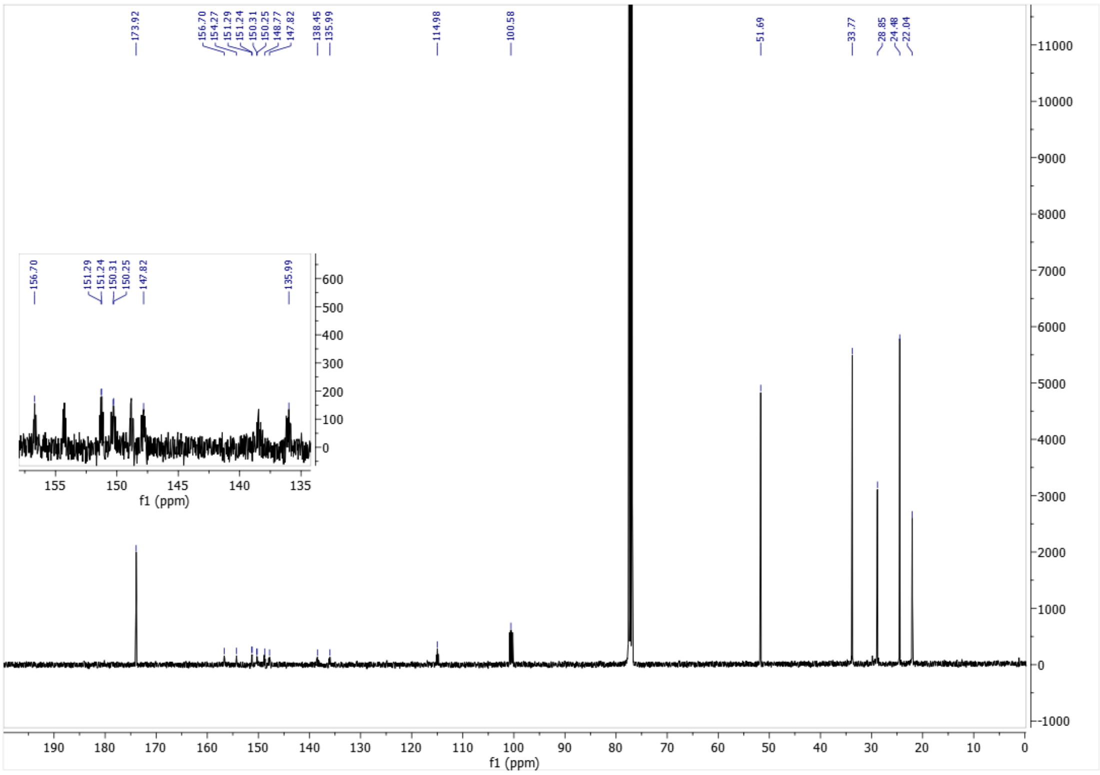

^{13}C-NMR spectrum of (**f9**) (400 MHz, $CDCl_3$)

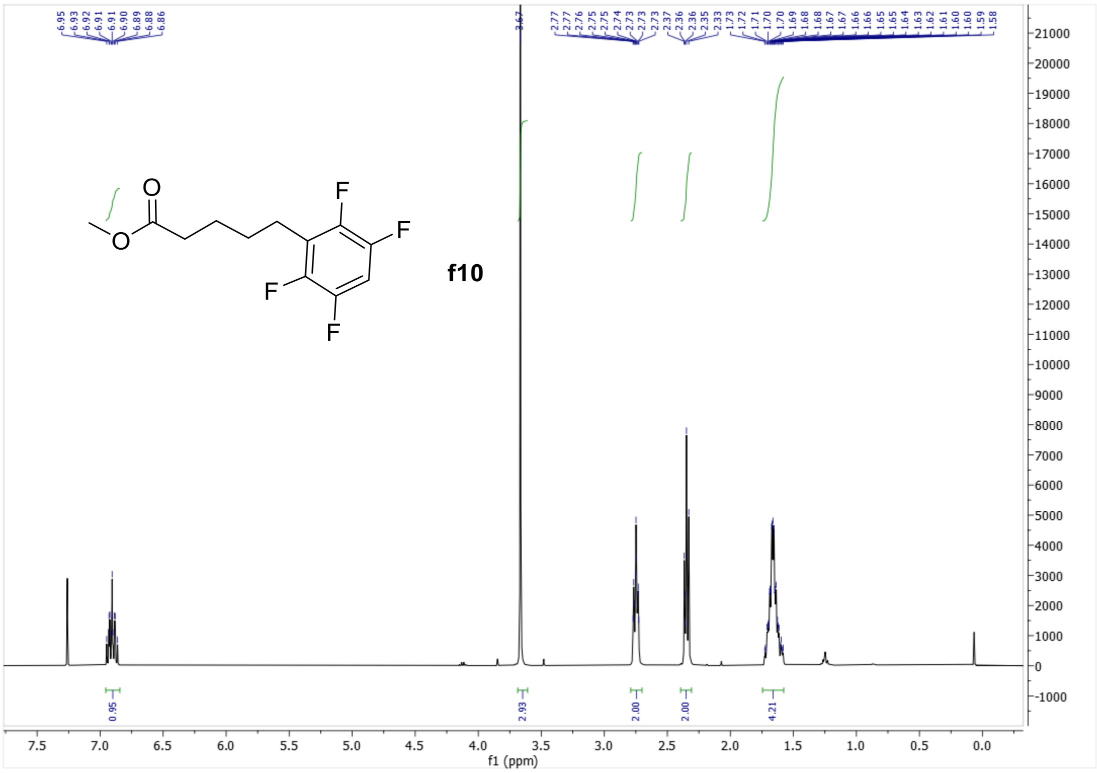

^{1}H-NMR spectrum of (**f10**) (400 MHz, $CDCl_3$)

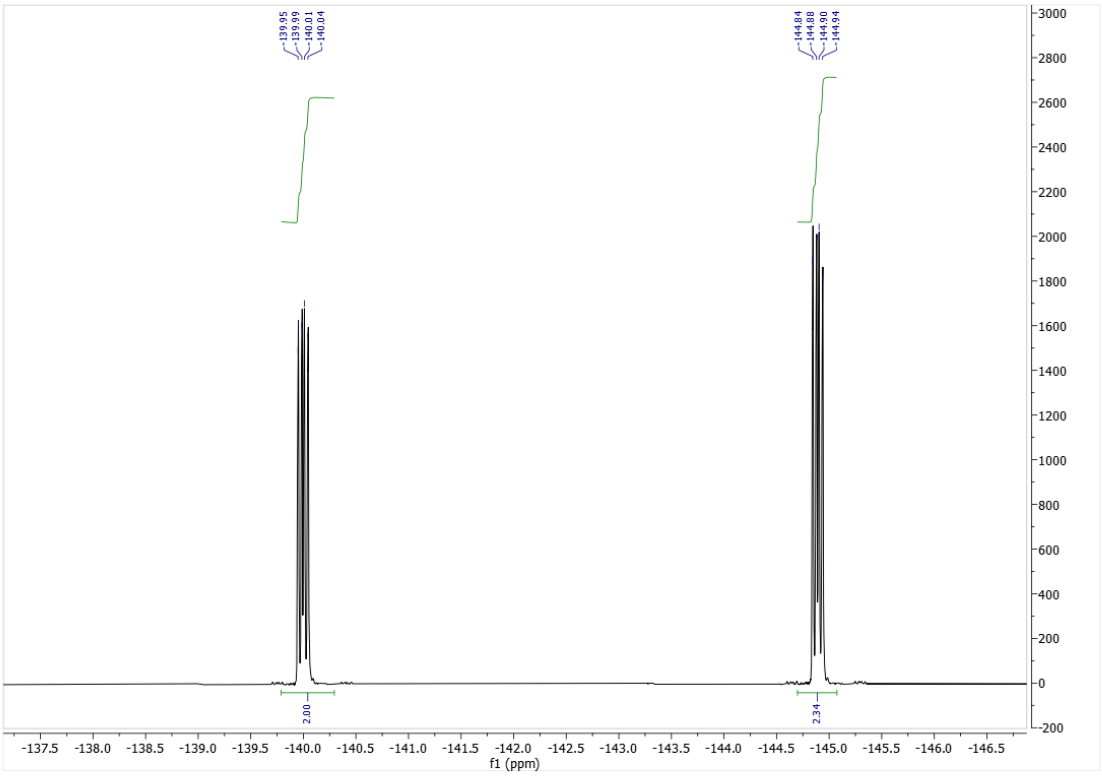

^{19}F-NMR spectrum of (**f10**) (400 MHz, $CDCl_3$)

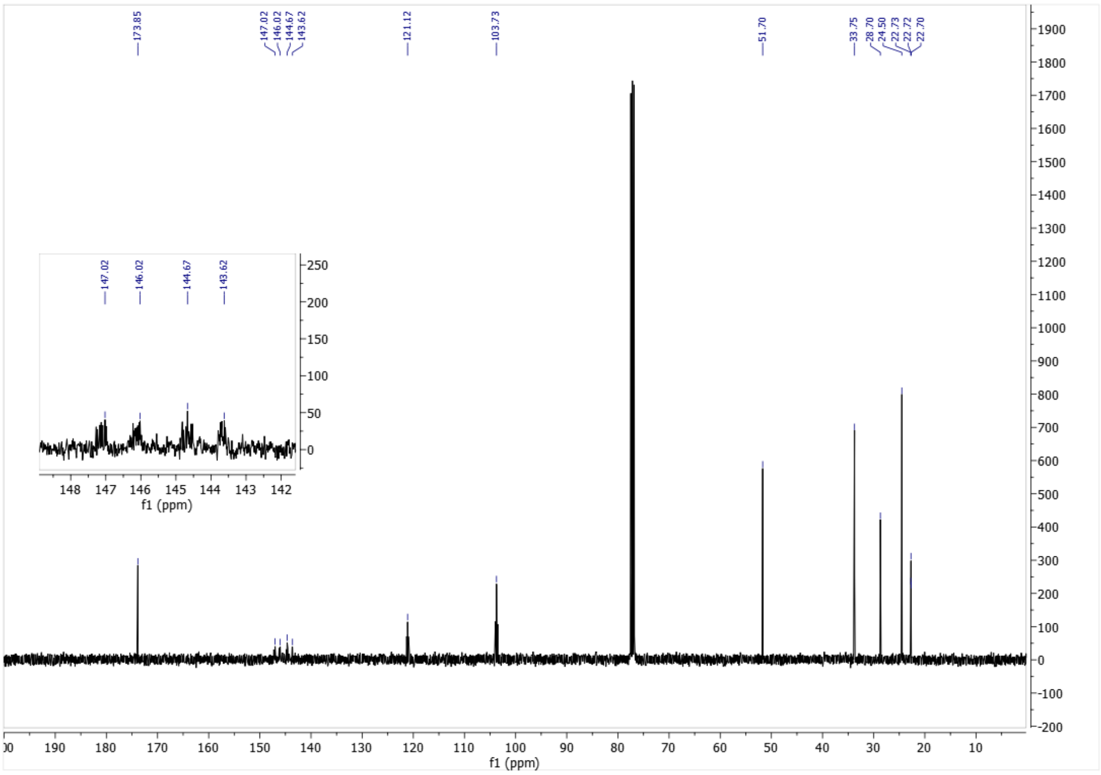

^{13}C-NMR spectrum of (**f10**) (400 MHz, $CDCl_3$)

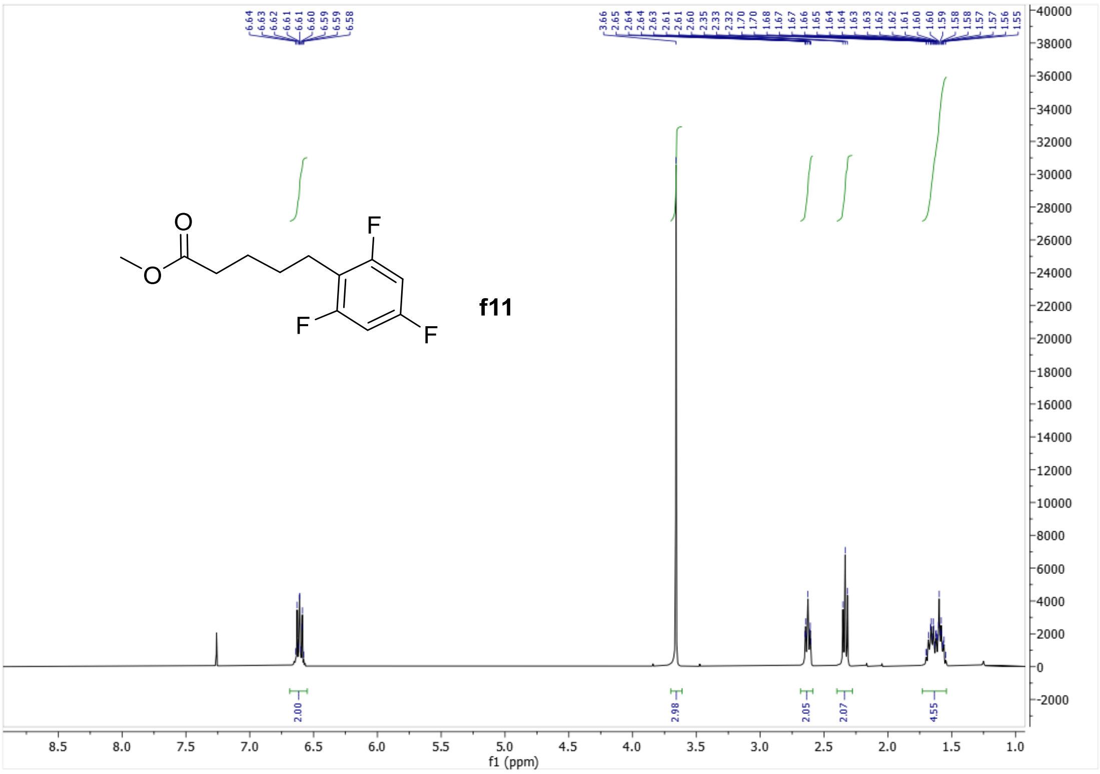

^{1}H-NMR spectrum of (**f11**) (400 MHz, $CDCl_3$)

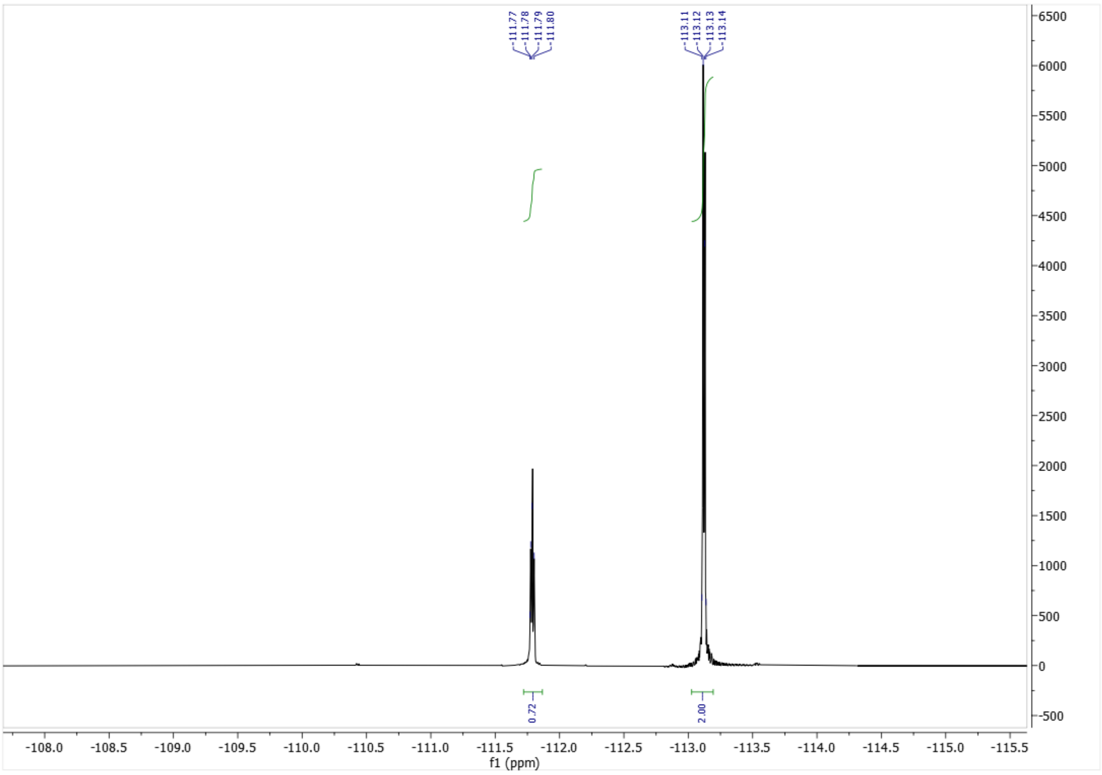

^{19}F-NMR spectrum of (**f11**) (400 MHz, $CDCl_3$)

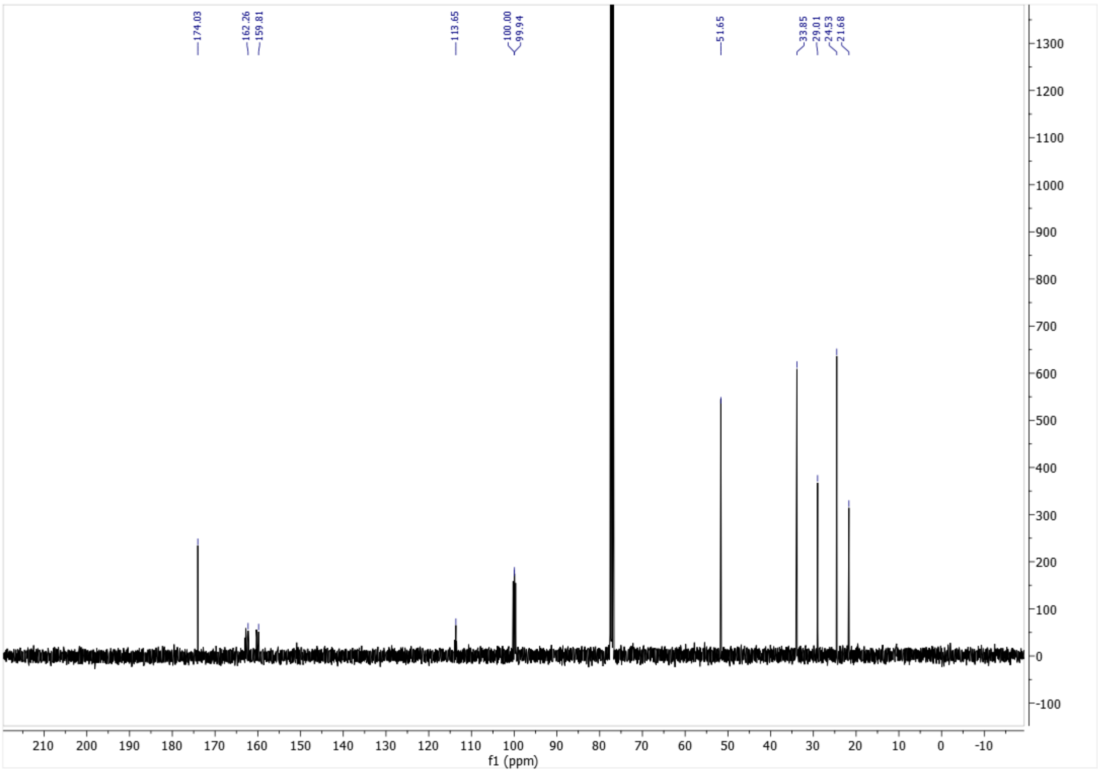

^{13}C-NMR spectrum of (**f11**) (400 MHz, $CDCl_3$)

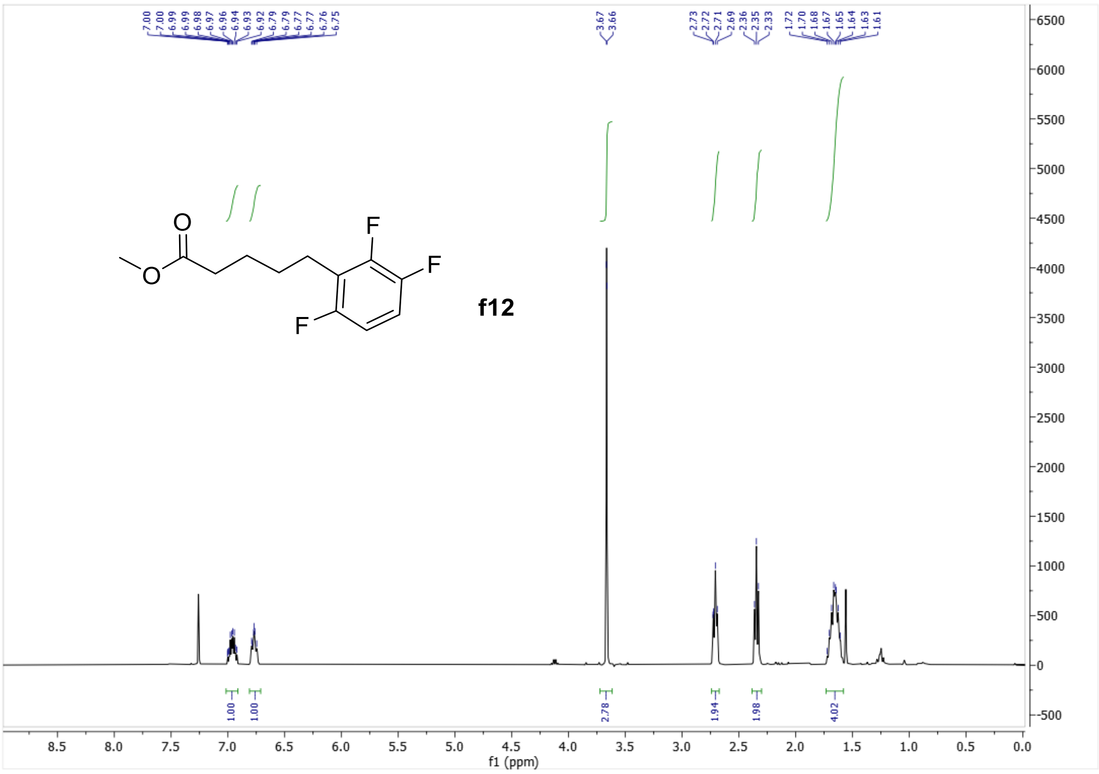

^{1}H-NMR spectrum of (**f12**) (400 MHz, $CDCl_3$)

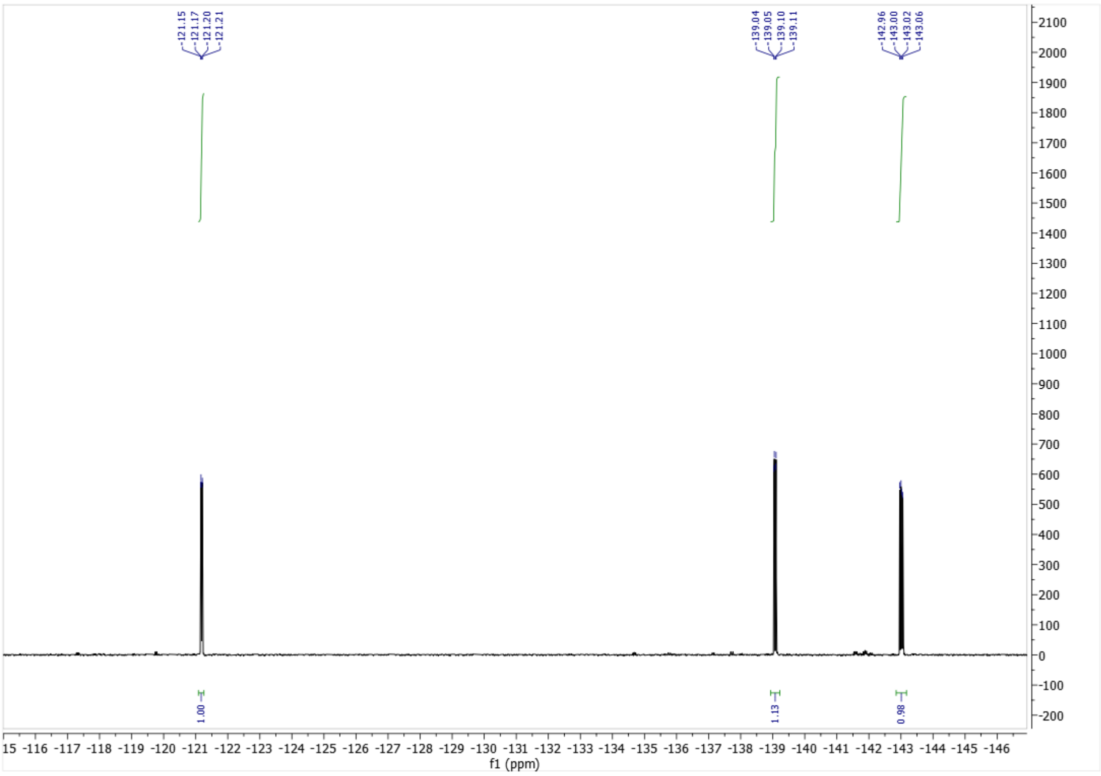

^{19}F-NMR spectrum of (**f12**) (400 MHz, $CDCl_3$)

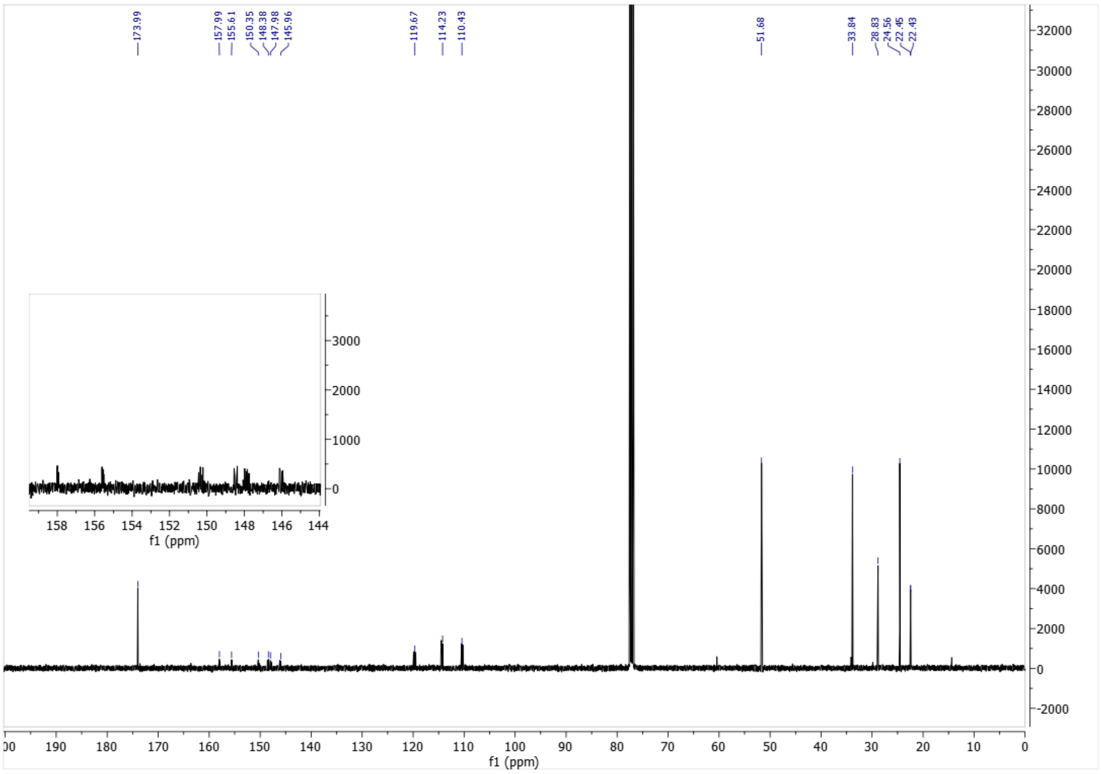

^{13}C-NMR spectrum of (**f12**) (400 MHz, $CDCl_3$)

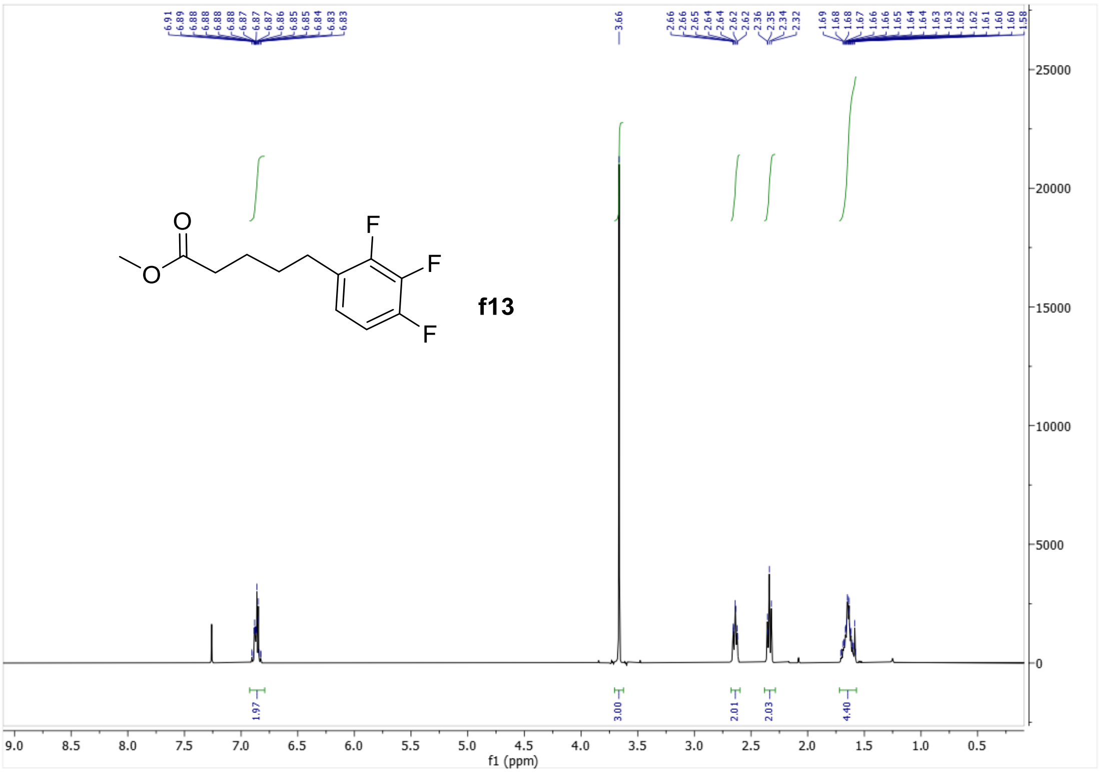

^{1}H-NMR spectrum of (**f13**) (400 MHz, $CDCl_3$)

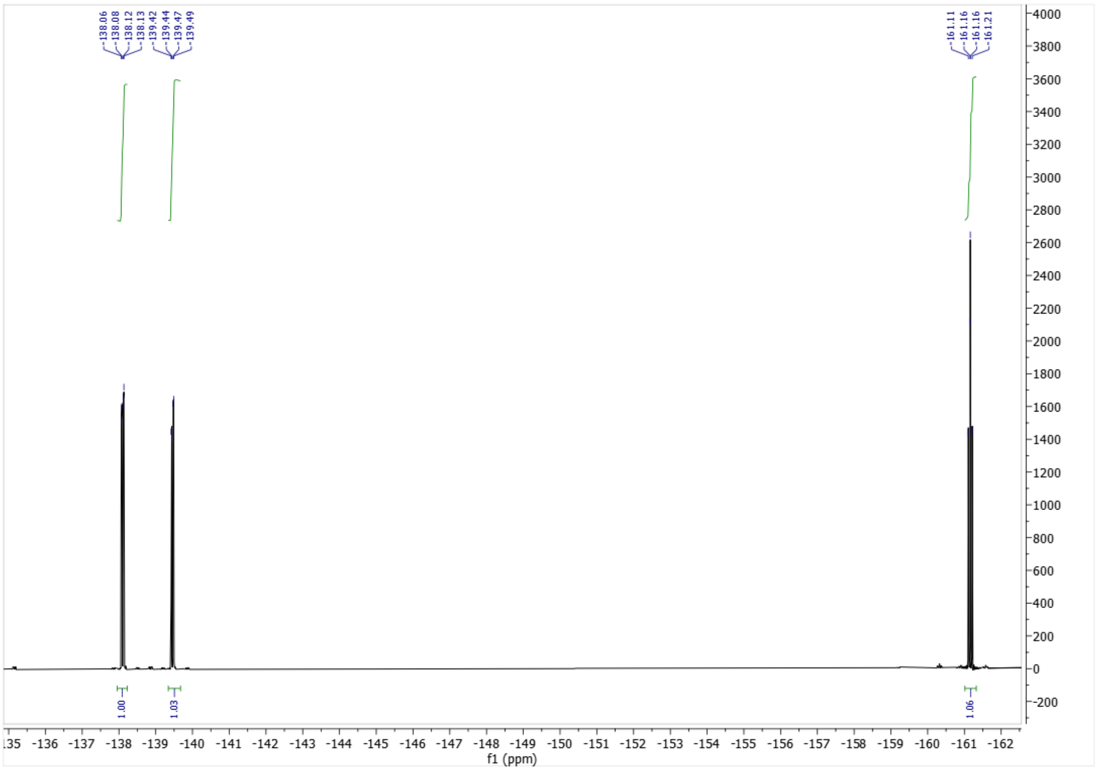

^{19}F-NMR spectrum of (**f13**) (400 MHz, $CDCl_3$)

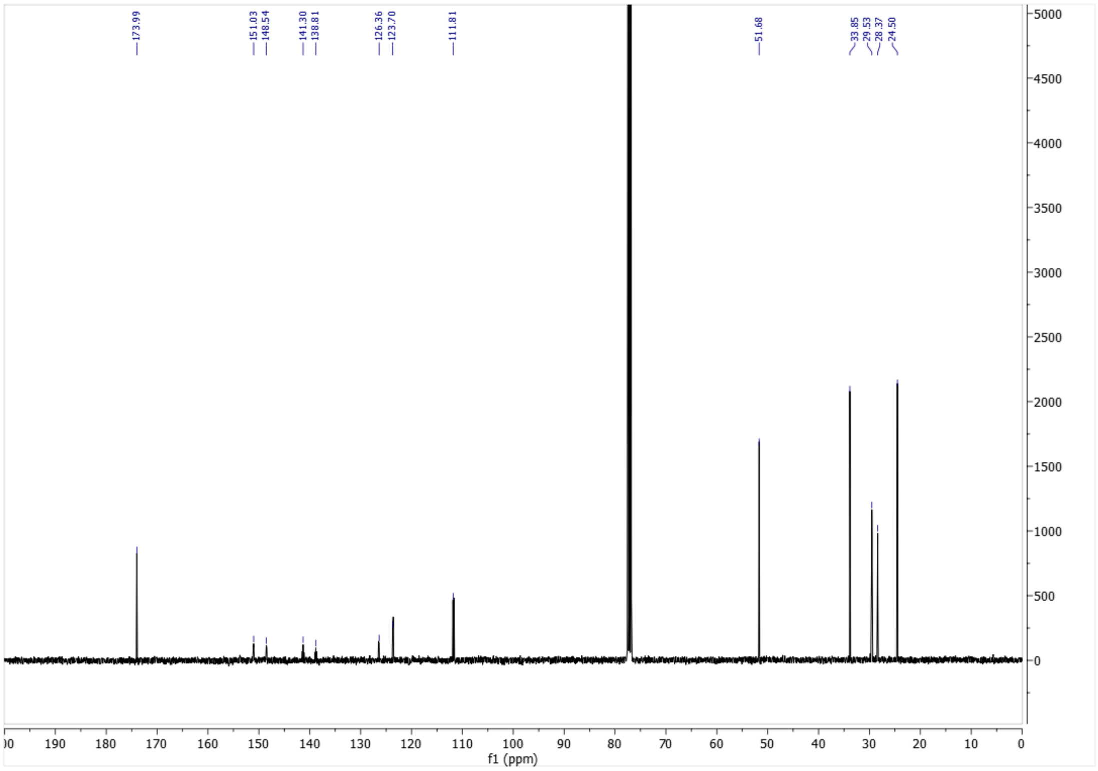

^{13}C-NMR spectrum of (**f13**) (400 MHz, $CDCl_3$)

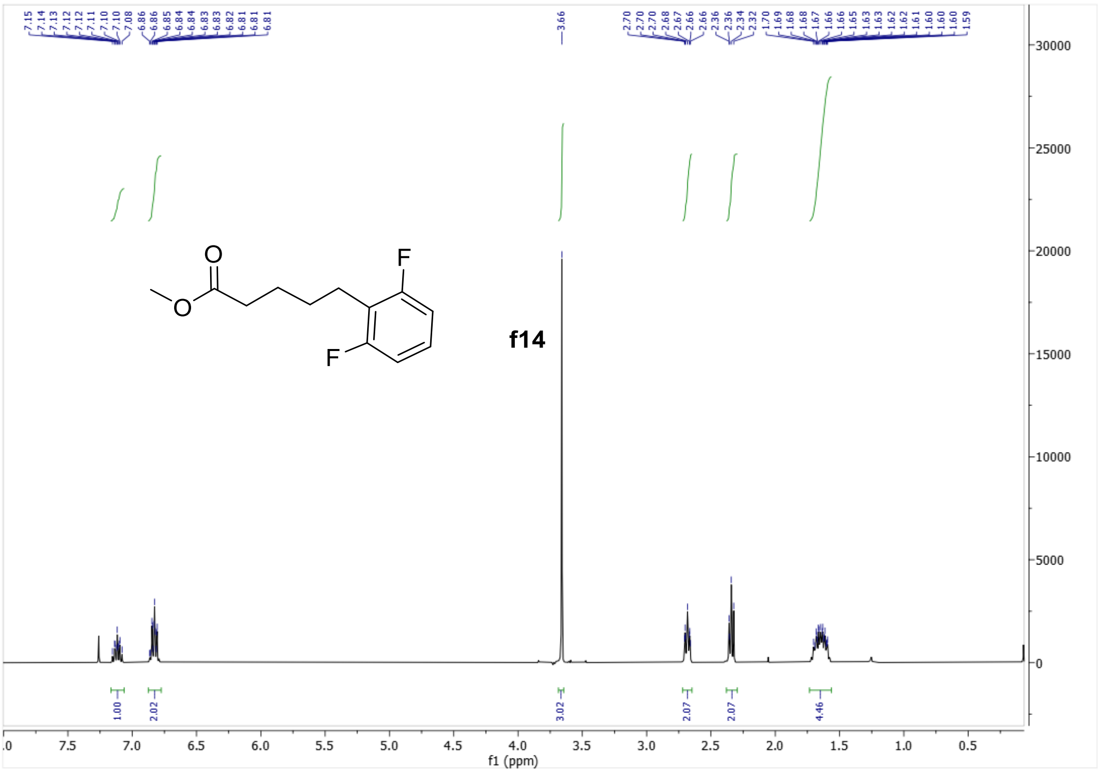

^{1}H-NMR spectrum of (**f14**) (400 MHz, $CDCl_3$)

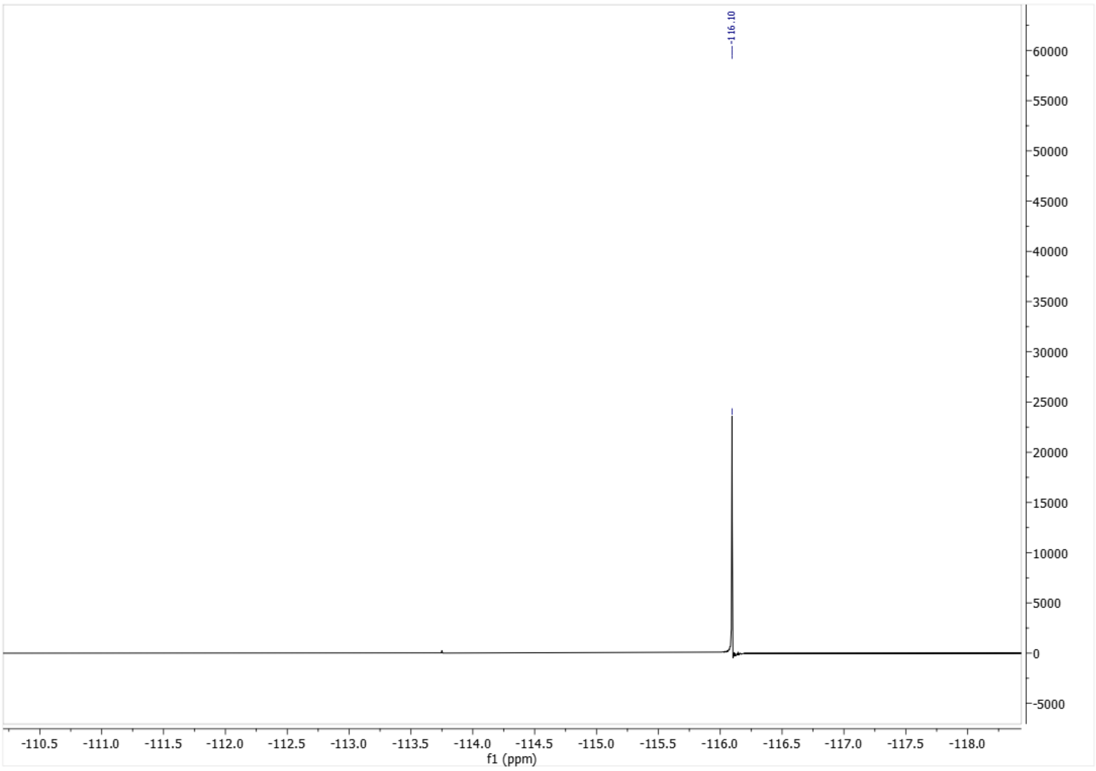

^{19}F-NMR spectrum of (**f14**) (400 MHz, $CDCl_3$)

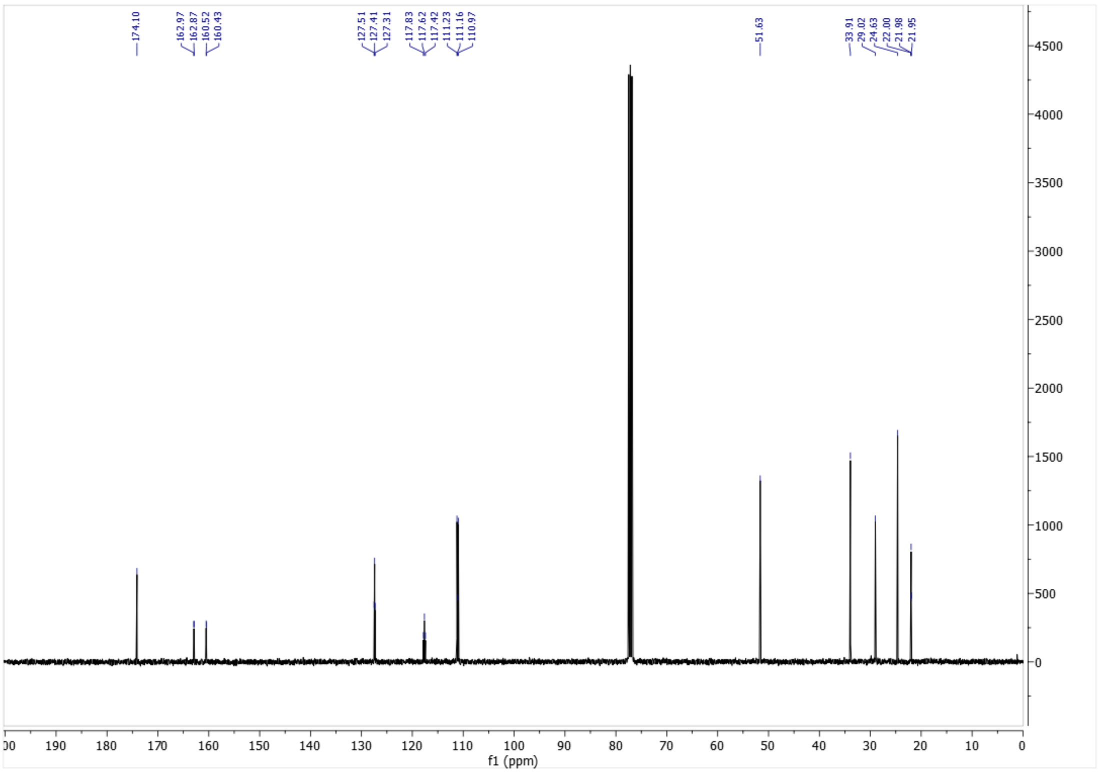

^{13}C-NMR spectrum of (**f14**) (400 MHz, $CDCl_3$)

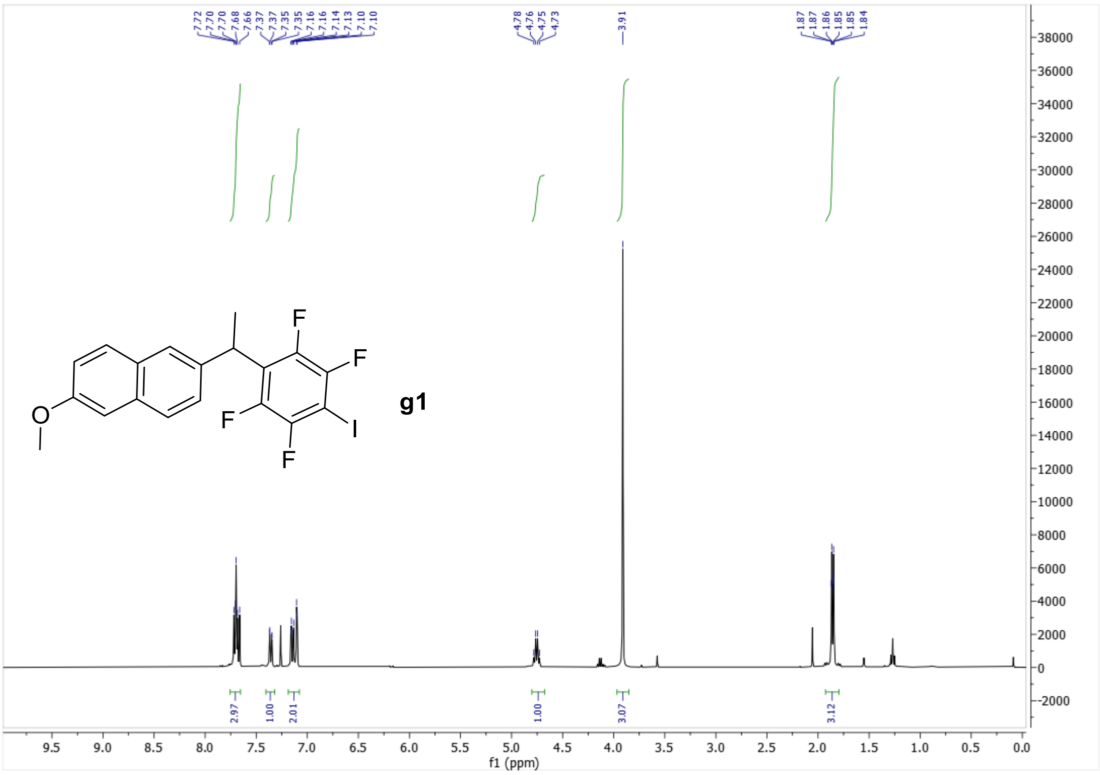

^{1}H-NMR spectrum of (**g1**) (400 MHz, $CDCl_3$)

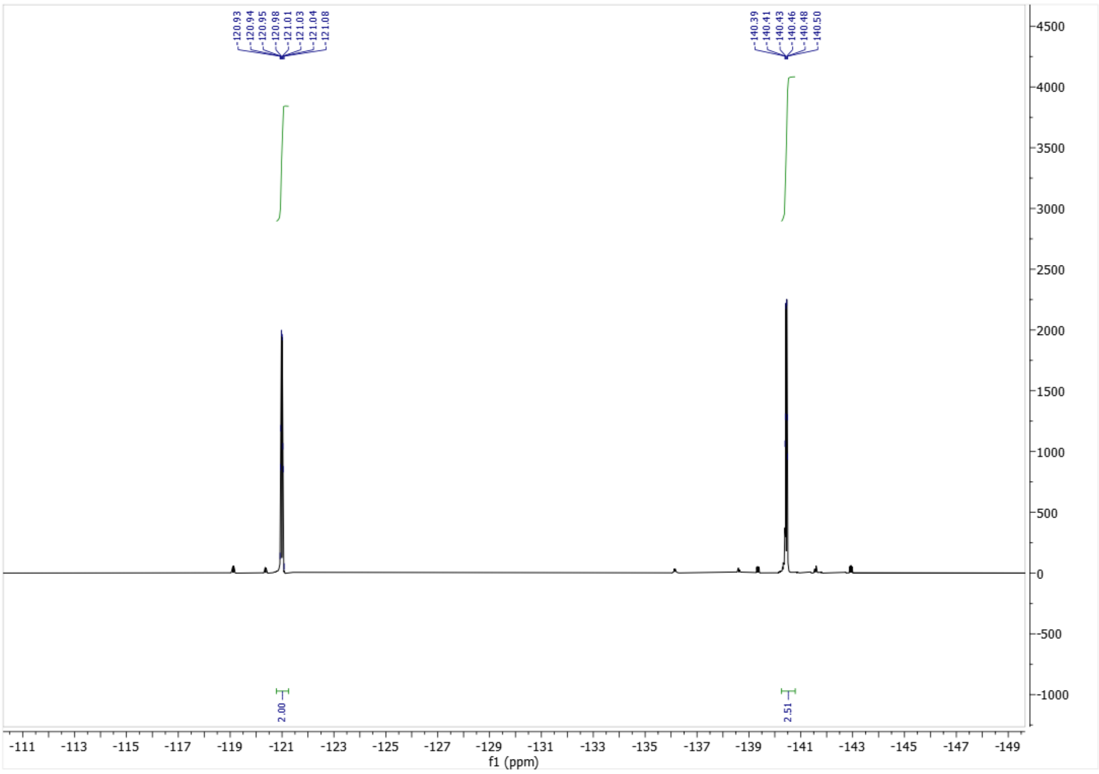

^{19}F-NMR spectrum of (**g1**) (400 MHz, $CDCl_3$)

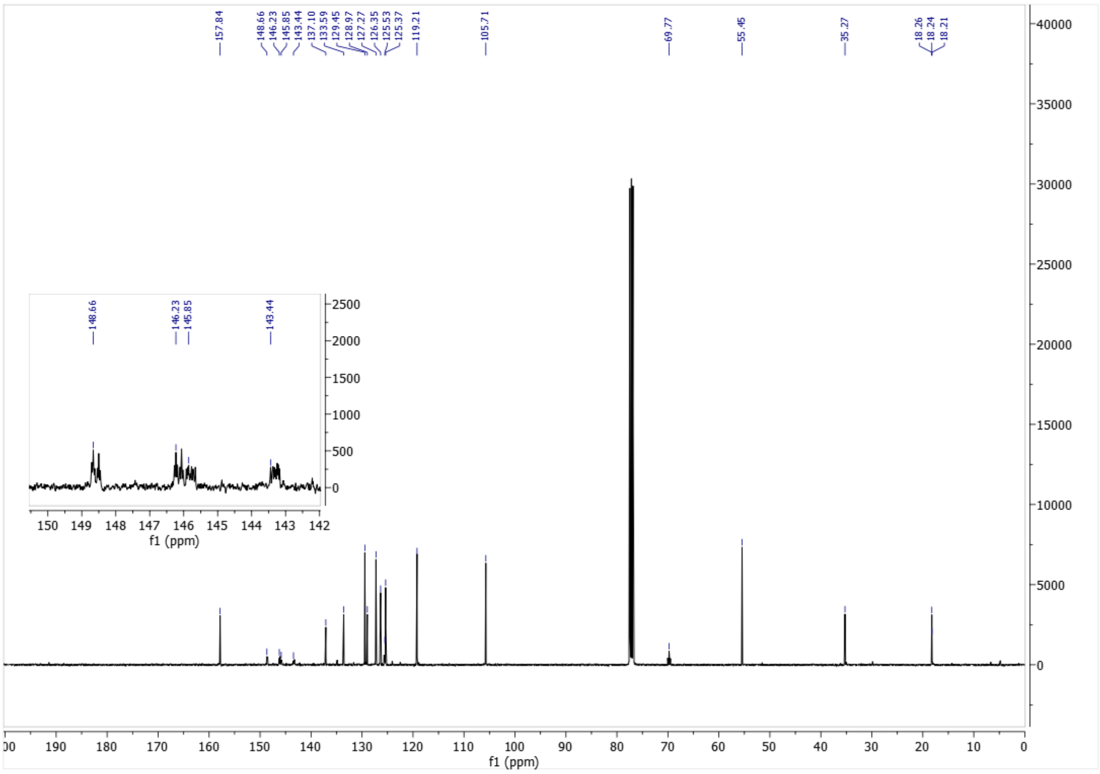

^{13}C-NMR spectrum of (**g1**) (400 MHz, $CDCl_3$)

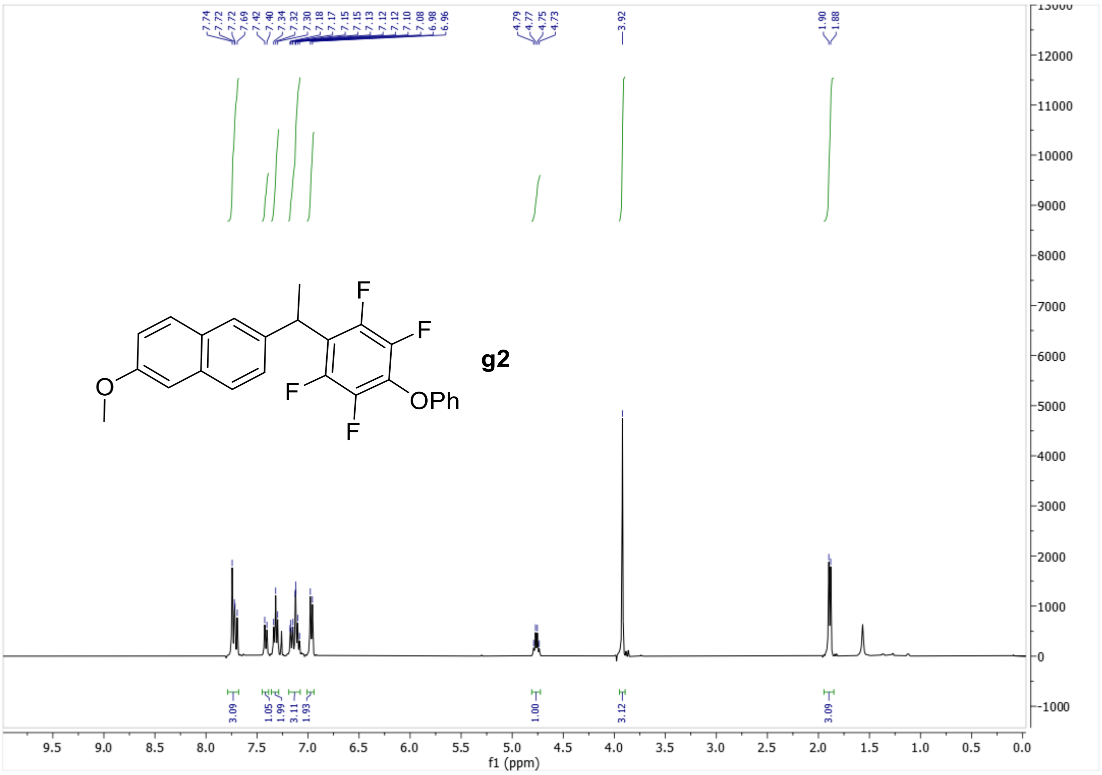

^{1}H-NMR spectrum of (**g2**) (400 MHz, $CDCl_3$)

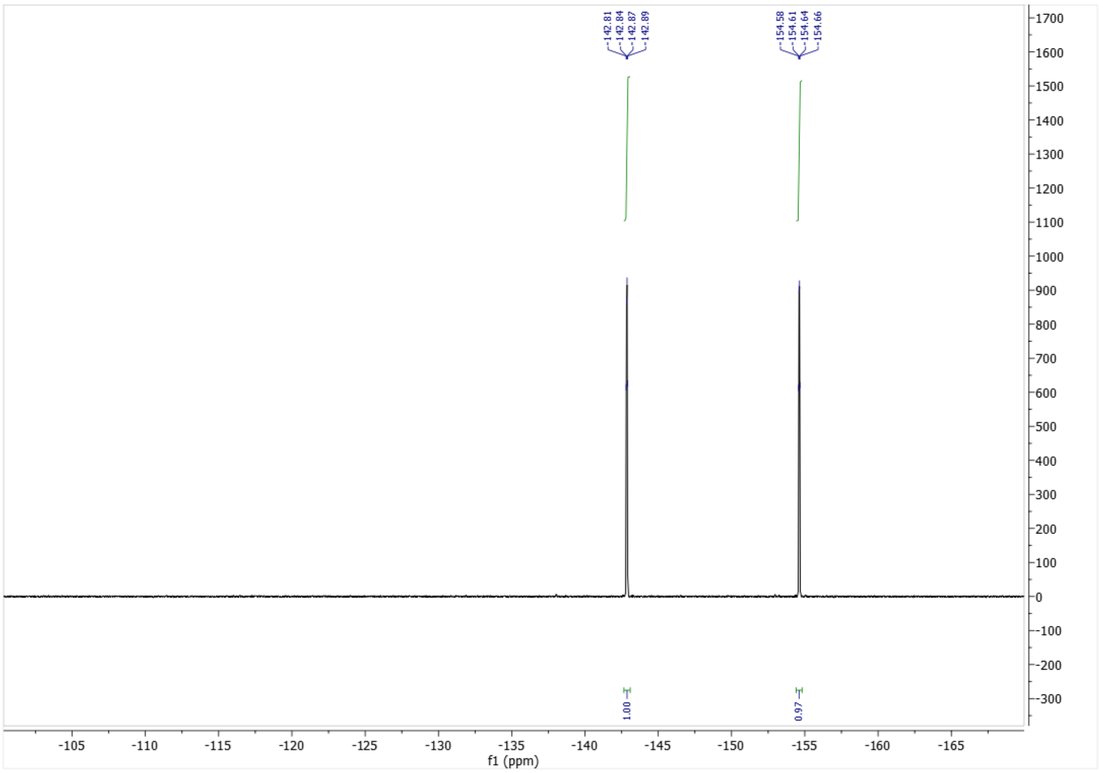

^{19}F-NMR spectrum of (**g2**) (400 MHz, $CDCl_3$)

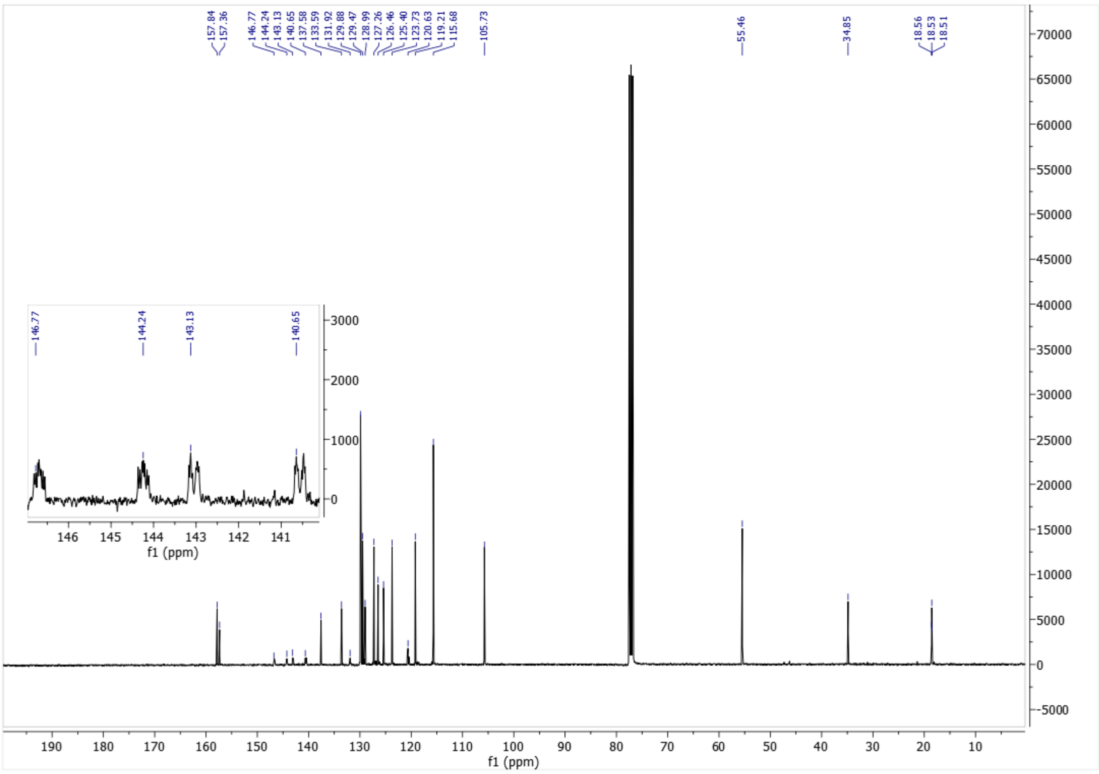

^{13}C-NMR spectrum of (**g2**) (400 MHz, $CDCl_3$)

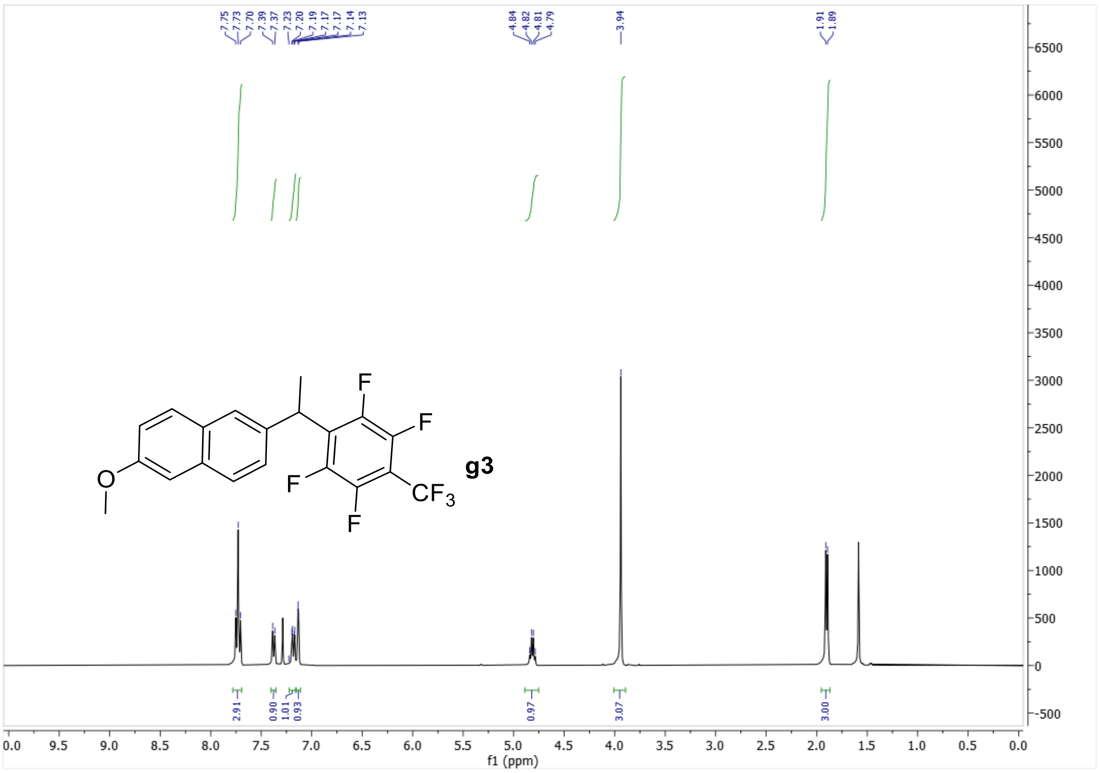

^{1}H-NMR spectrum of (**g3**) (400 MHz, $CDCl_3$)

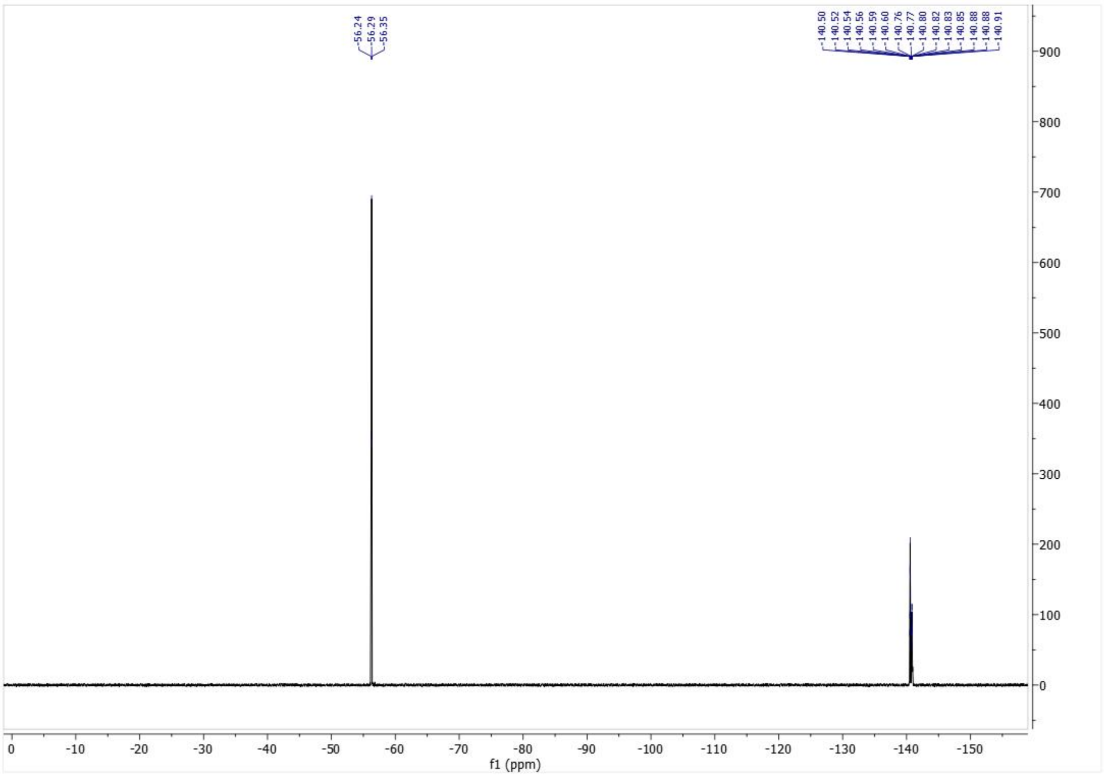

^{19}F-NMR spectrum of (**g3**) (400 MHz, $CDCl_3$)

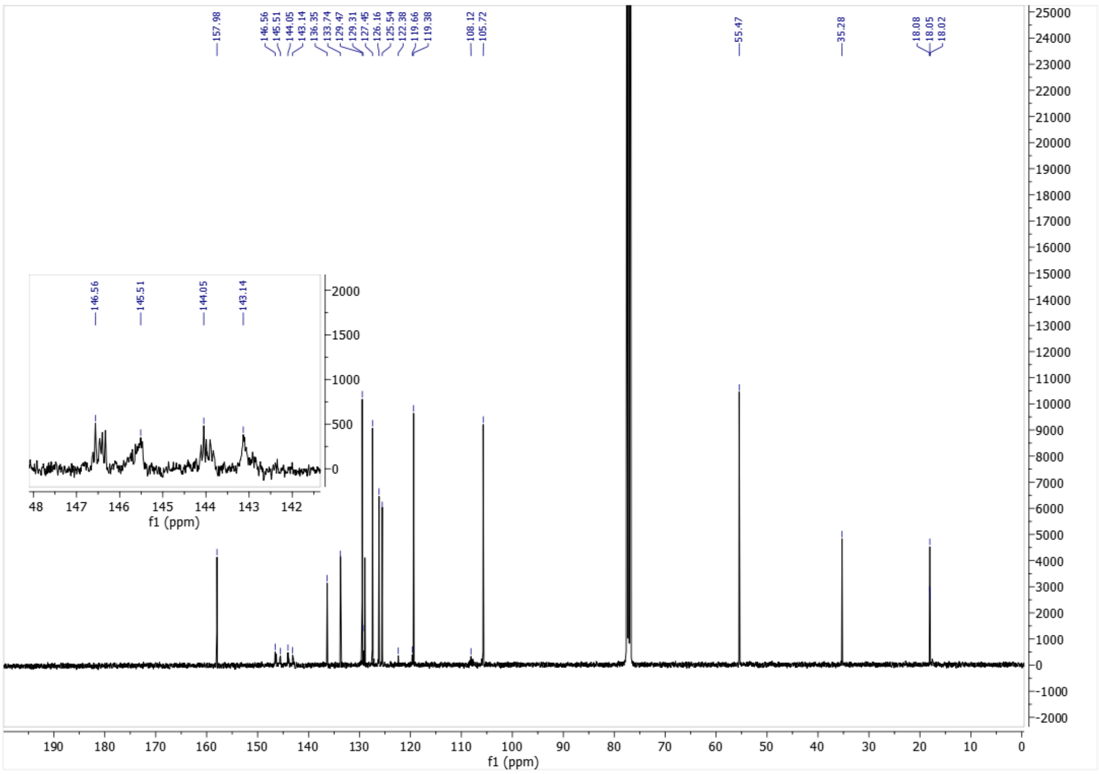

^{13}C-NMR spectrum of (**g3**) (400 MHz, $CDCl_3$)

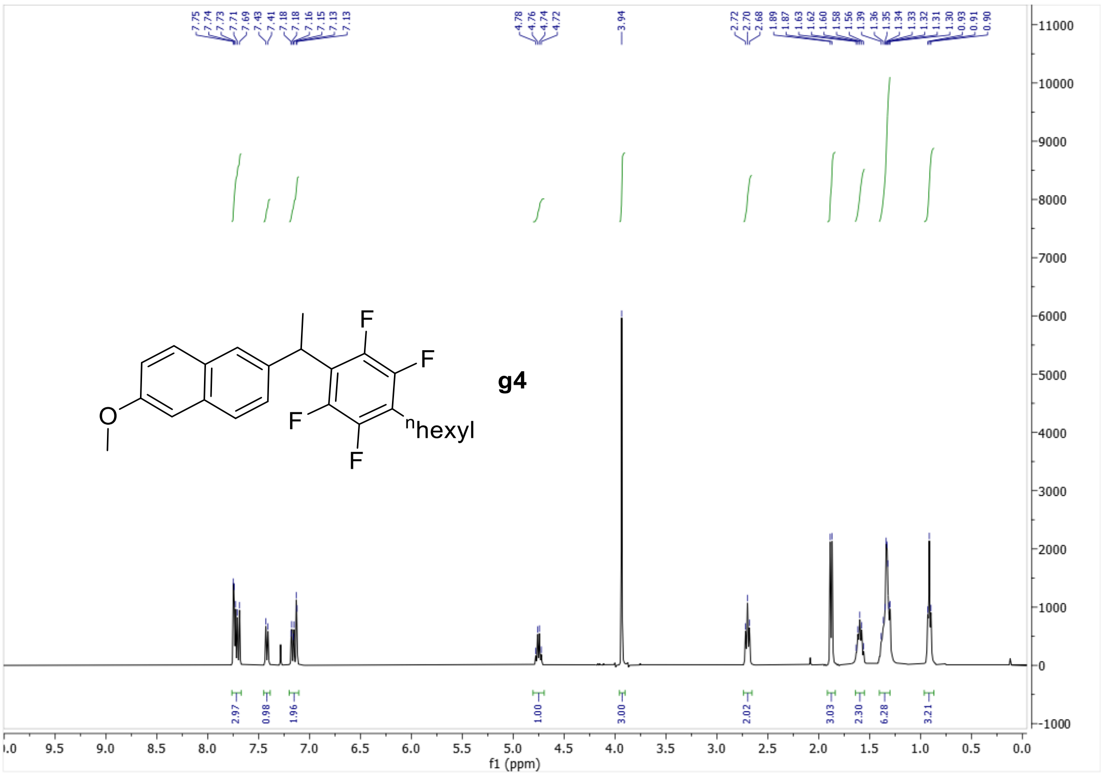

^{1}H-NMR spectrum of (**g4**) (400 MHz, $CDCl_3$)

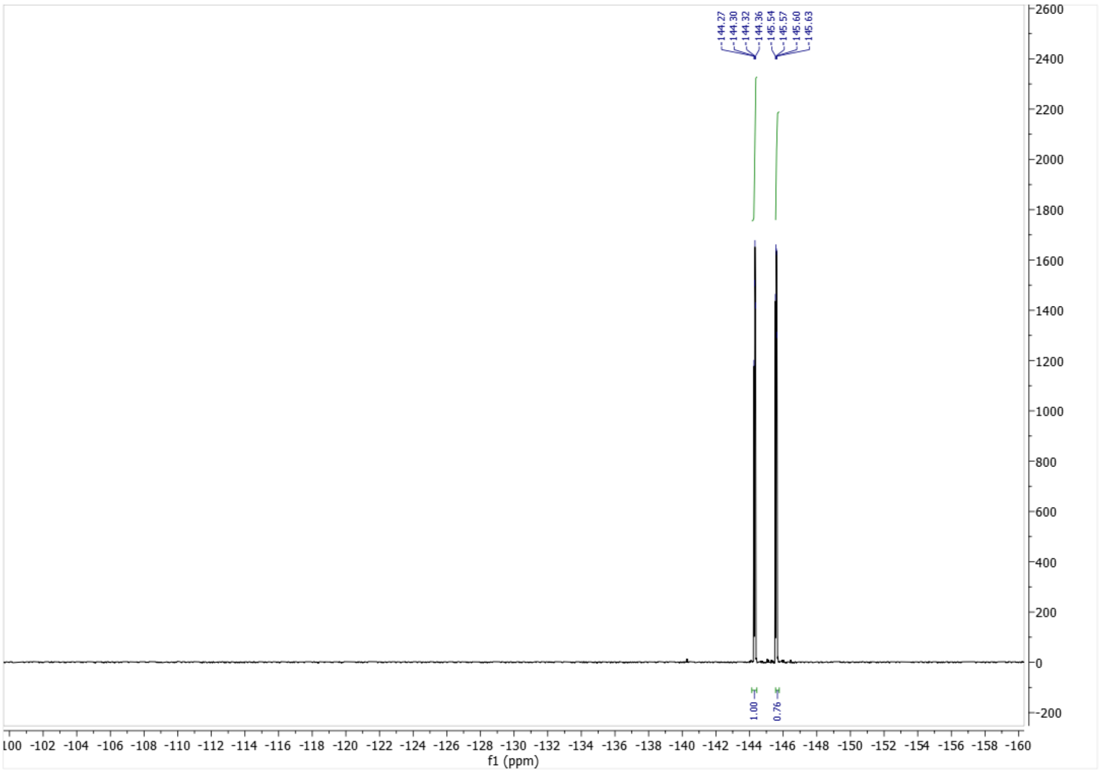

^{19}F-NMR spectrum of (**g4**) (400 MHz, $CDCl_3$)

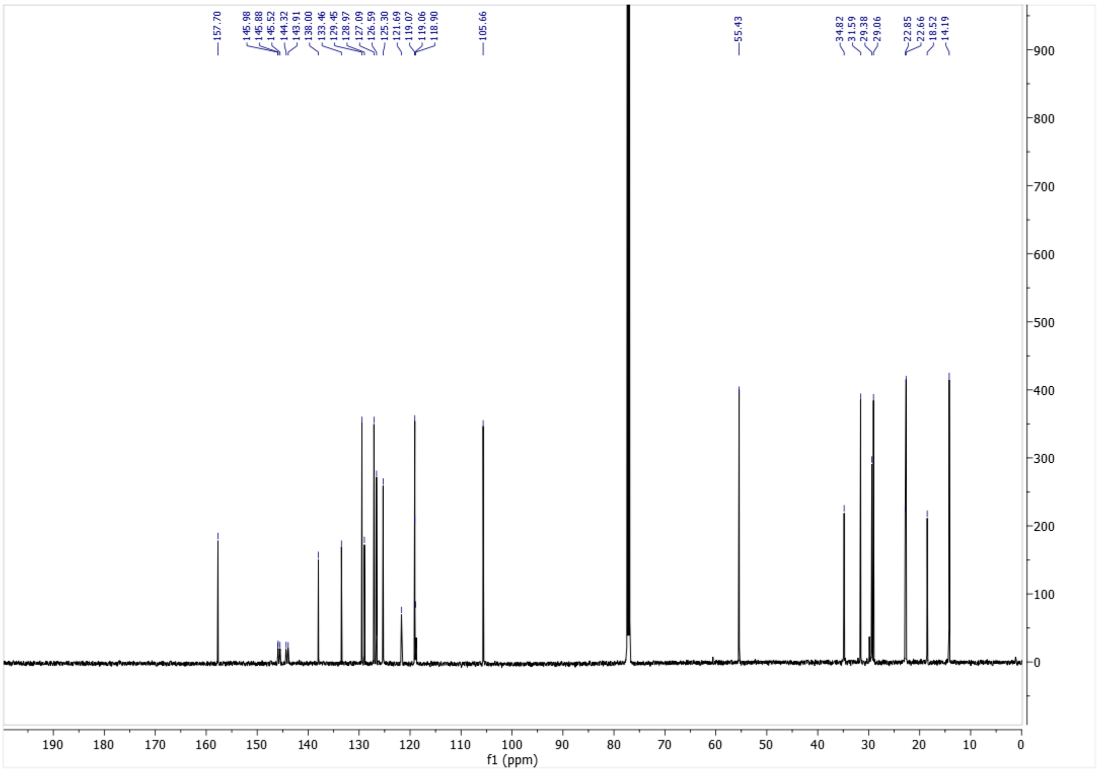

^{13}C-NMR spectrum of (**g4**) (400 MHz, $CDCl_3$)

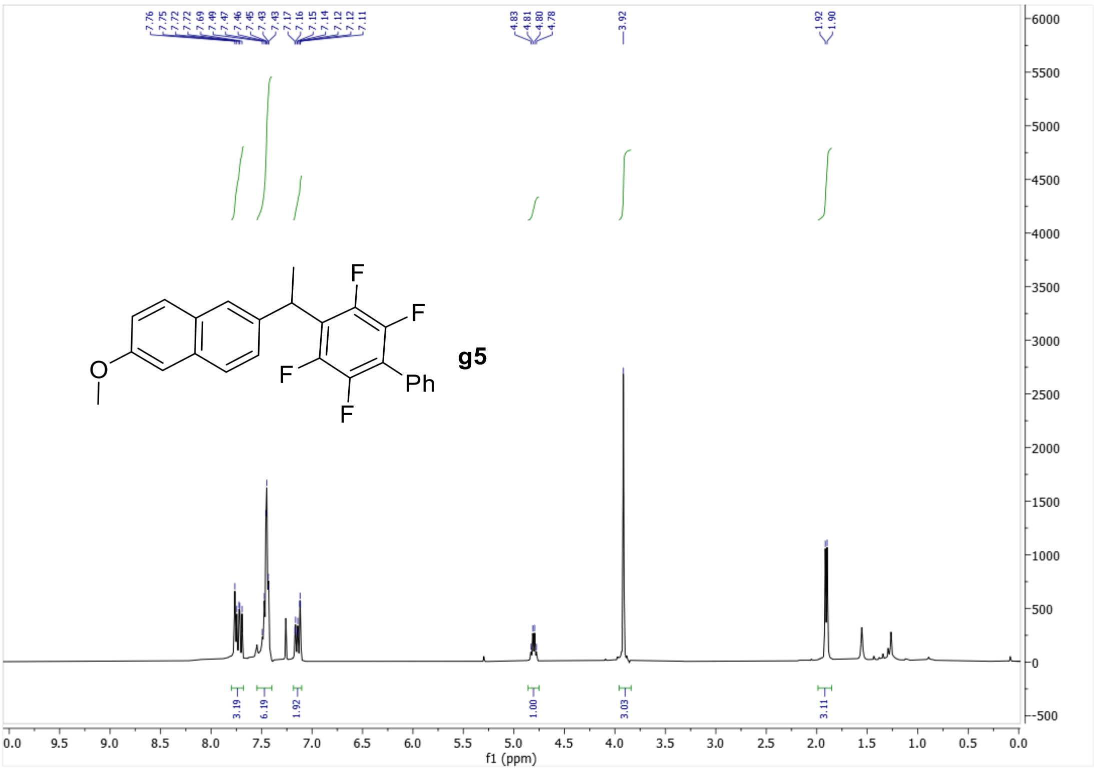

^{1}H-NMR spectrum of (**g5**) (400 MHz, $CDCl_3$)

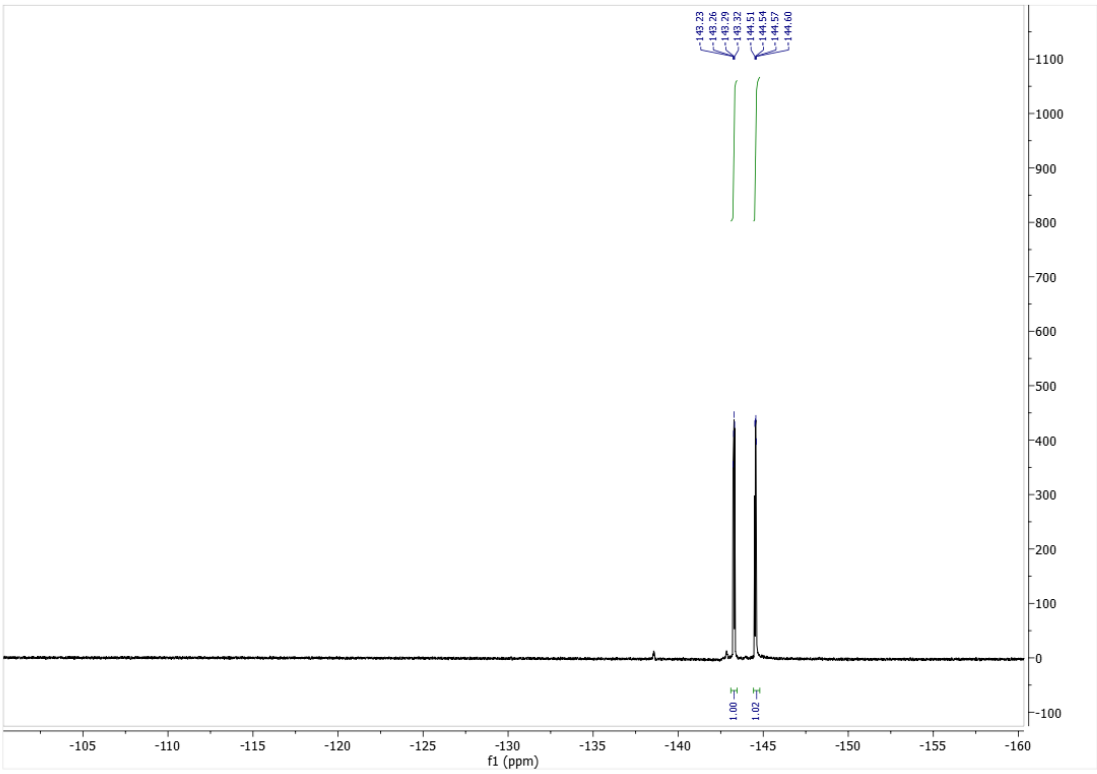

^{19}F-NMR spectrum of (**g5**) (400 MHz, $CDCl_3$)

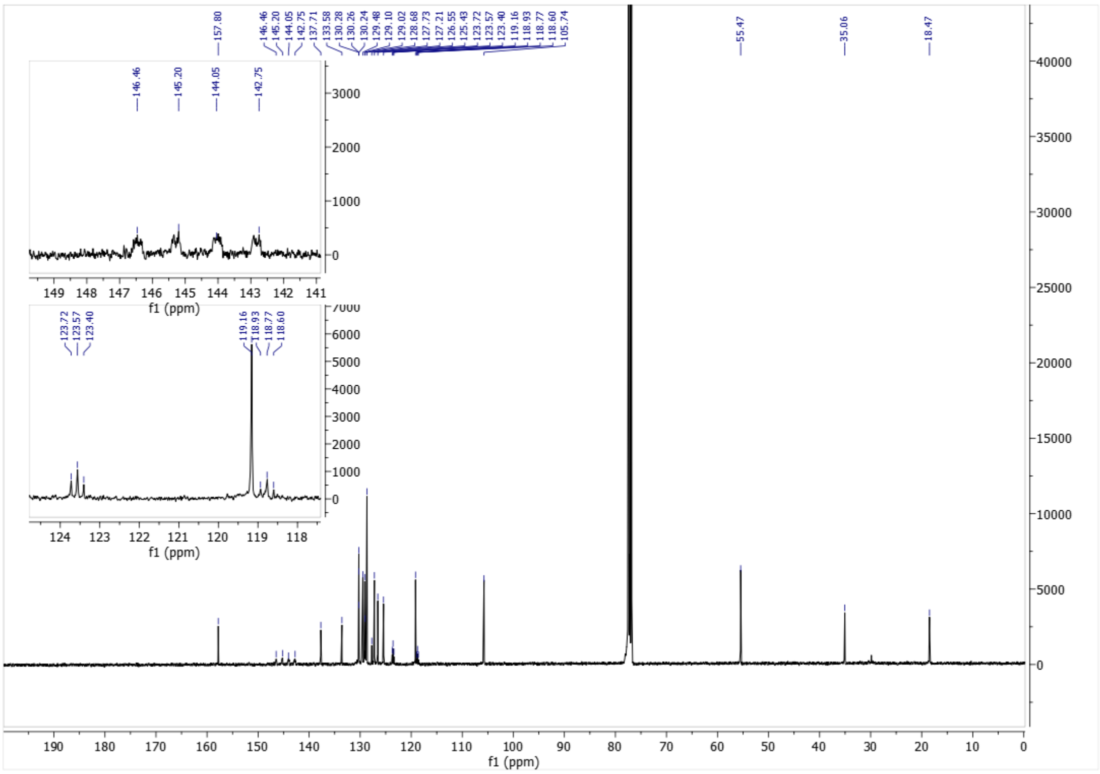

^{13}C-NMR spectrum of (**g5**) (400 MHz, $CDCl_3$)

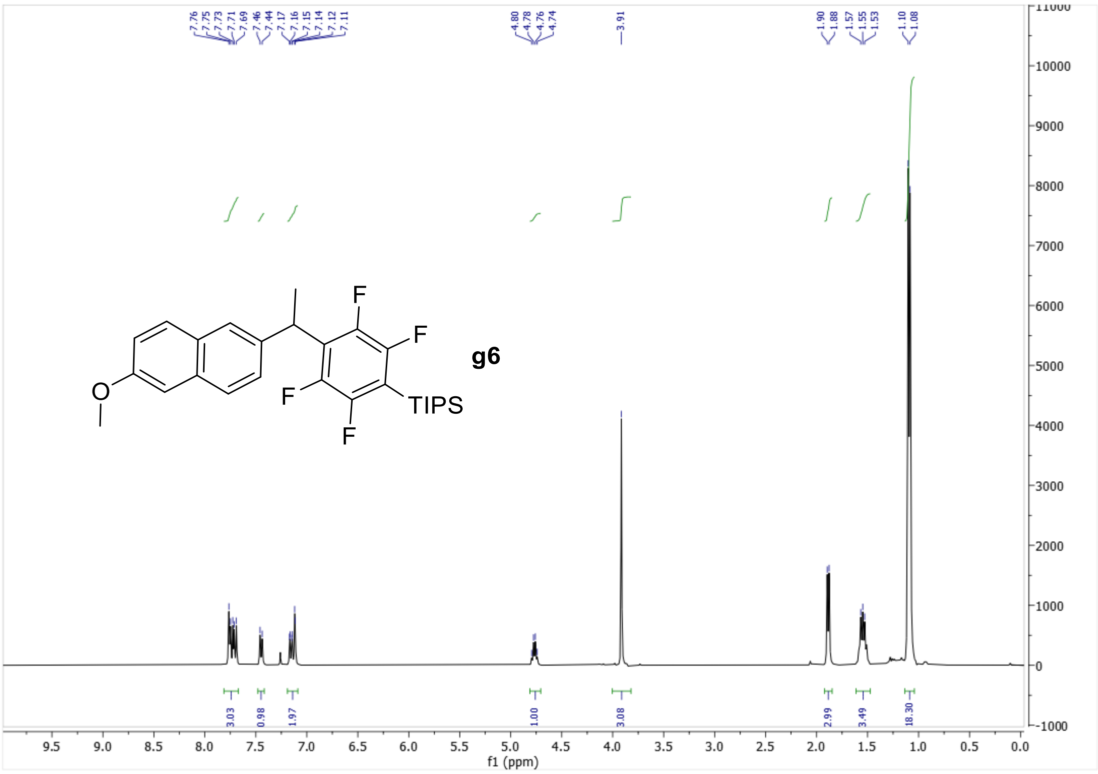

^{1}H-NMR spectrum of (**g6**) (400 MHz, $CDCl_3$)

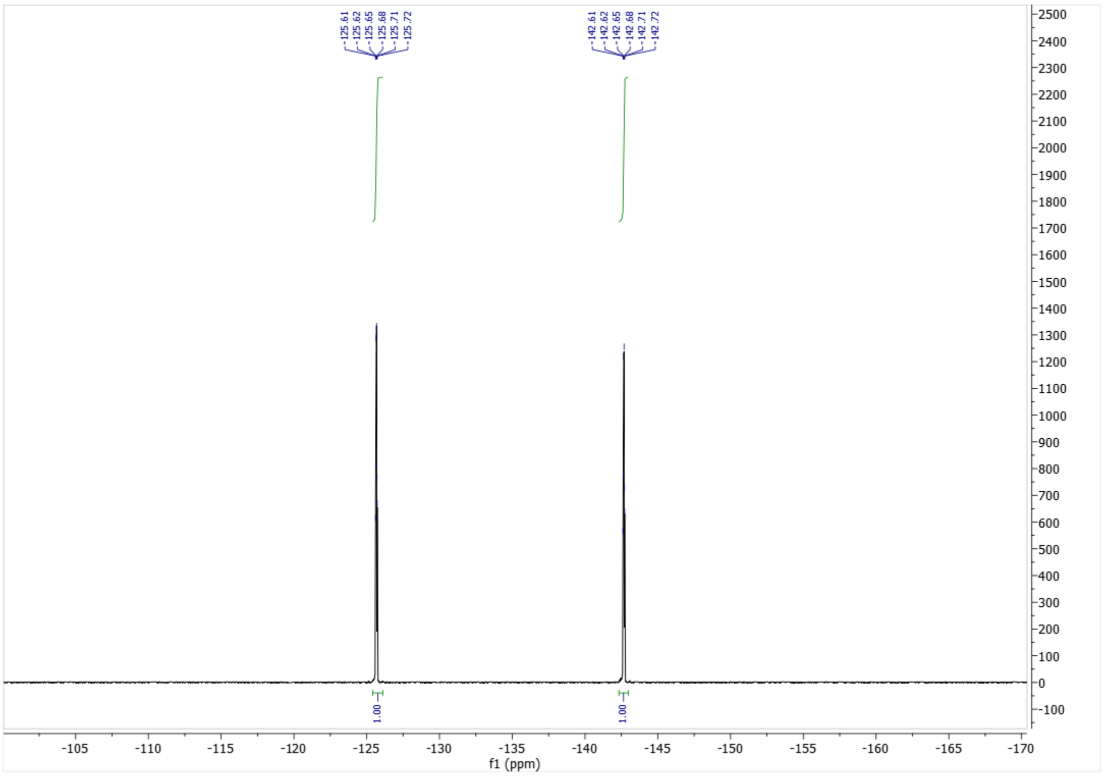

^{19}F-NMR spectrum of (**g6**) (400 MHz, $CDCl_3$)

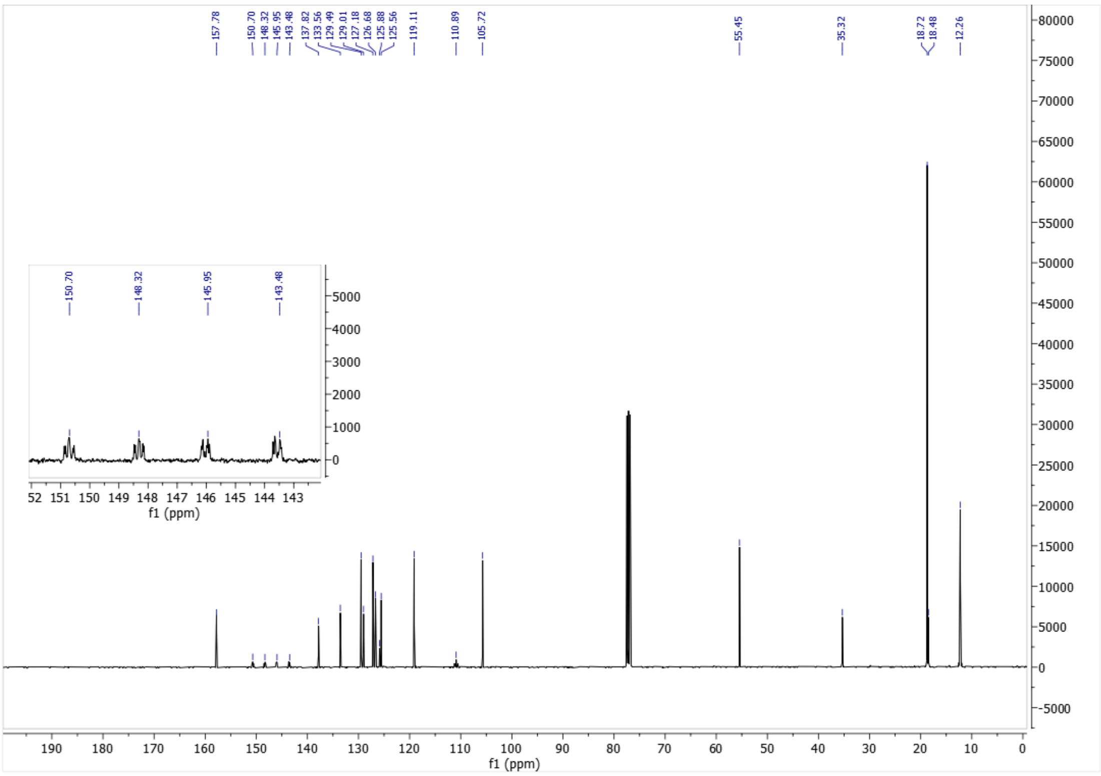

^{13}C-NMR spectrum of (**g6**) (400 MHz, $CDCl_3$)

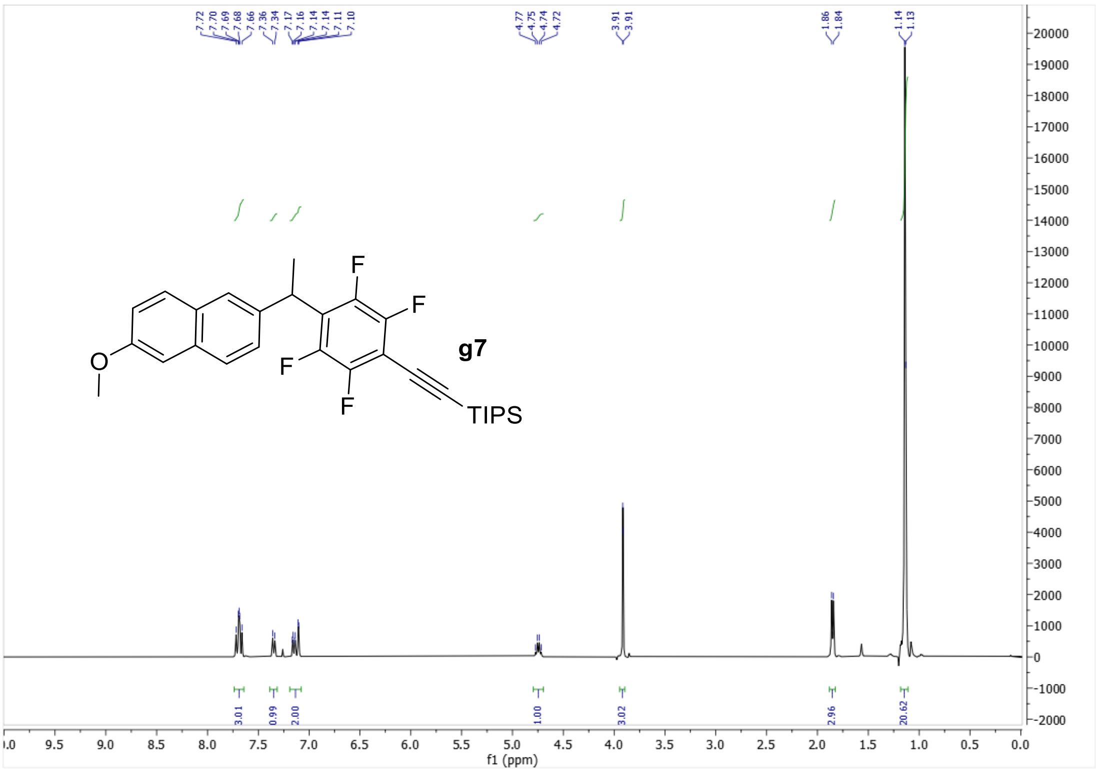

^{1}H-NMR spectrum of (**g7**) (400 MHz, CDCl$_3$)

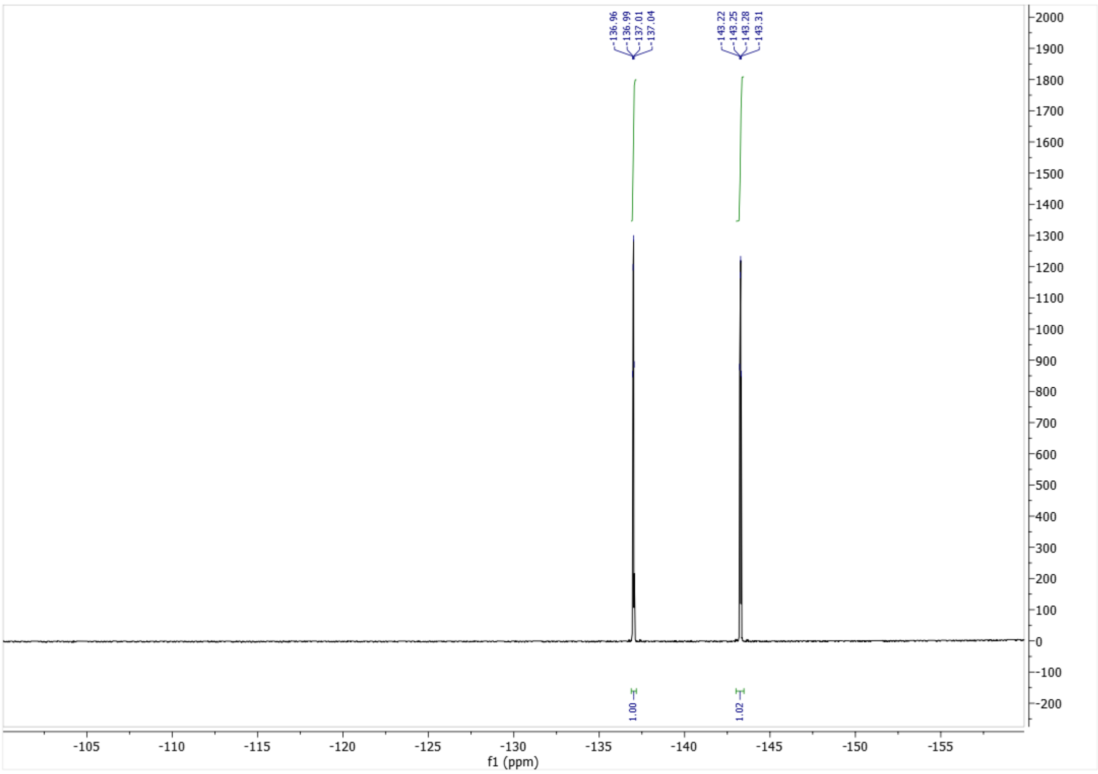

^{19}F-NMR spectrum of (**g7**) (400 MHz, CDCl$_3$)

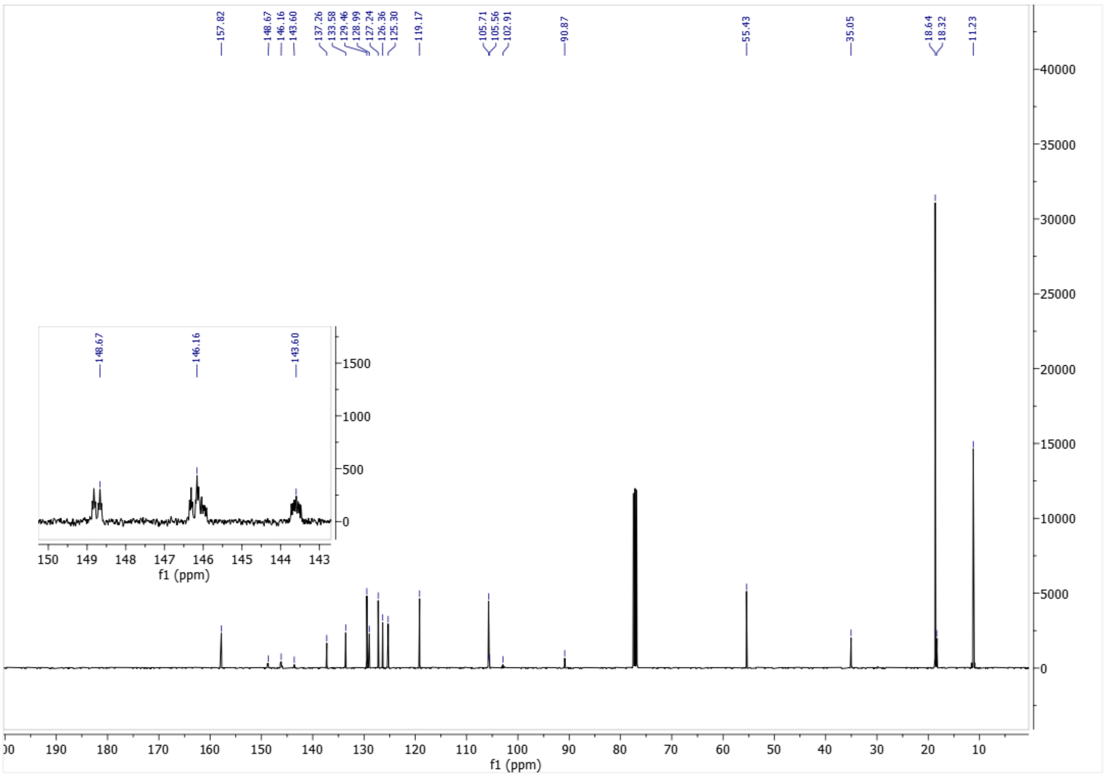

^{13}C-NMR spectrum of (**g7**) (400 MHz, $CDCl_3$)

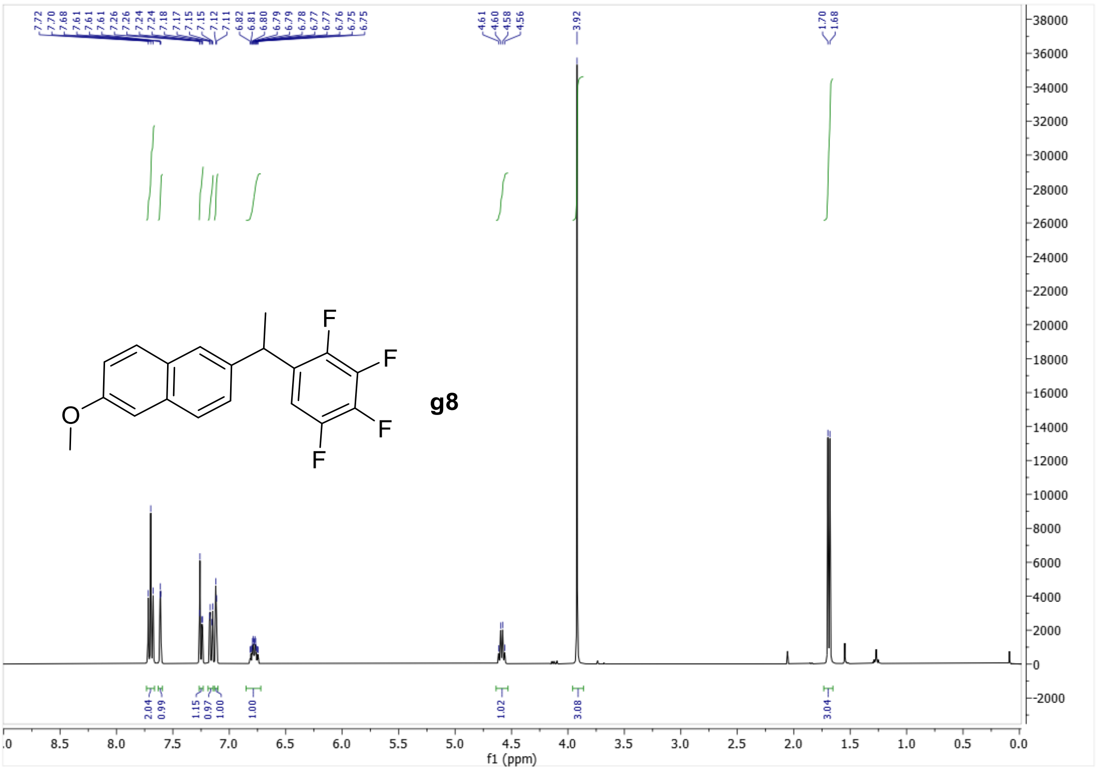

^{1}H-NMR spectrum of (**g8**) (400 MHz, $CDCl_3$)

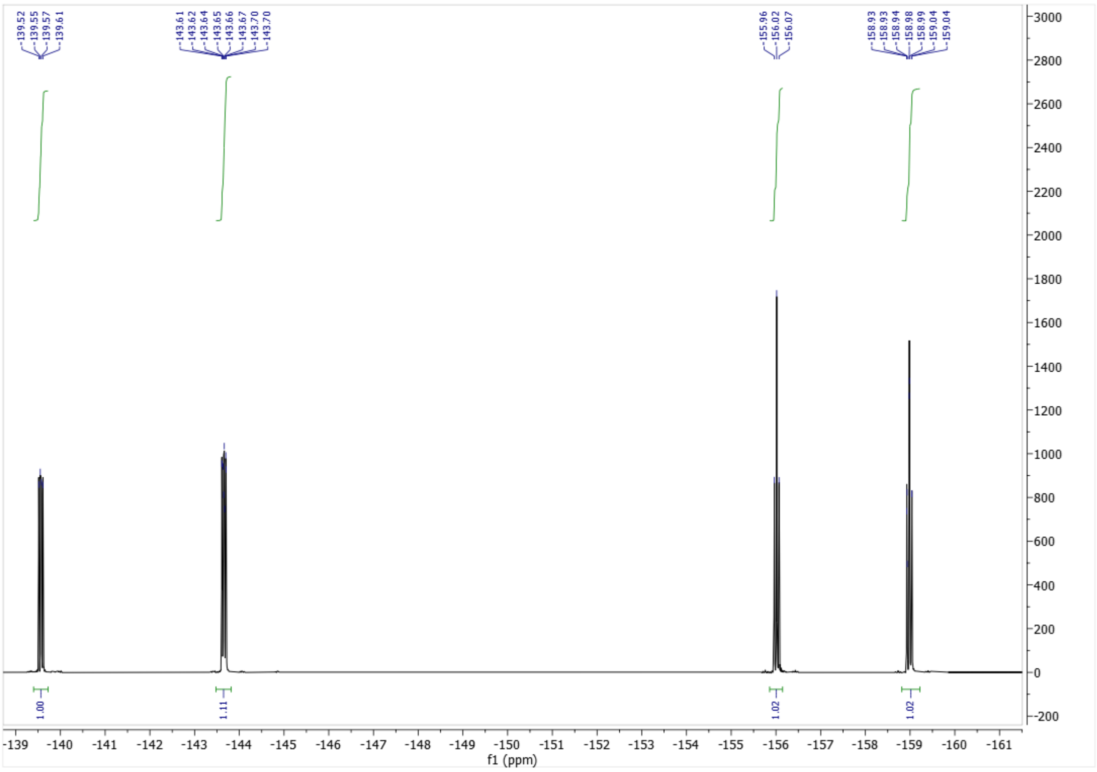

^{19}F-NMR spectrum of (**g8**) (400 MHz, $CDCl_3$)

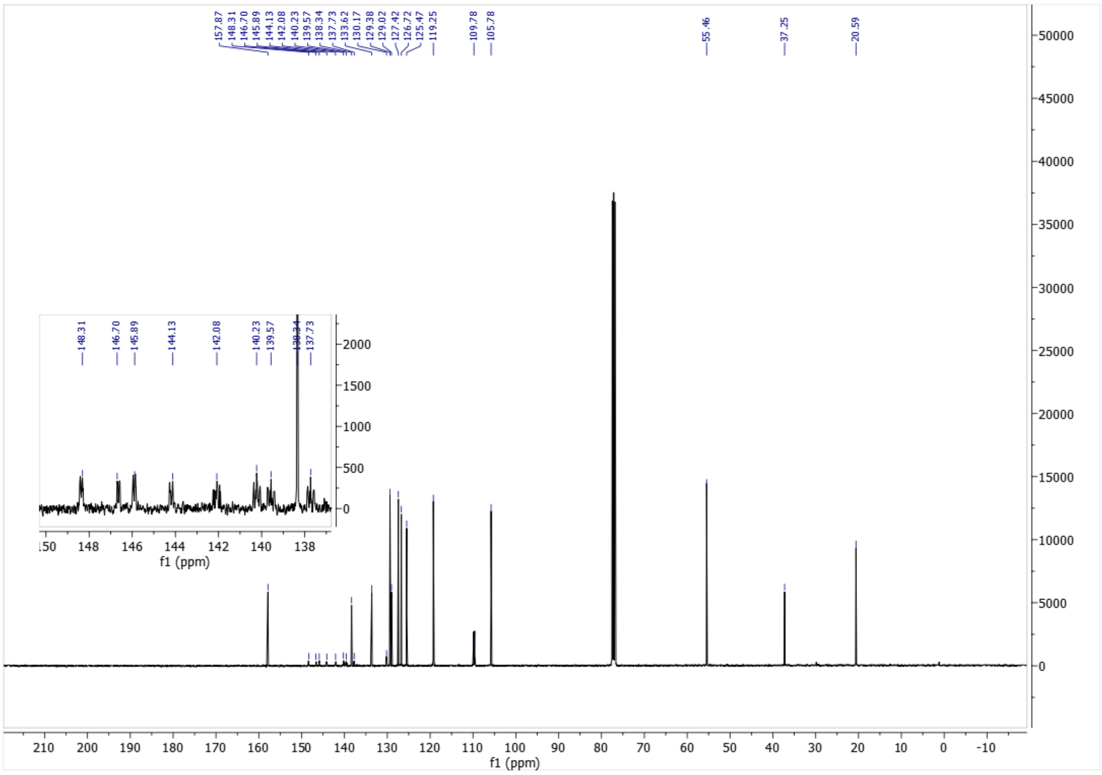

^{13}C-NMR spectrum of (**g8**) (400 MHz, $CDCl_3$)

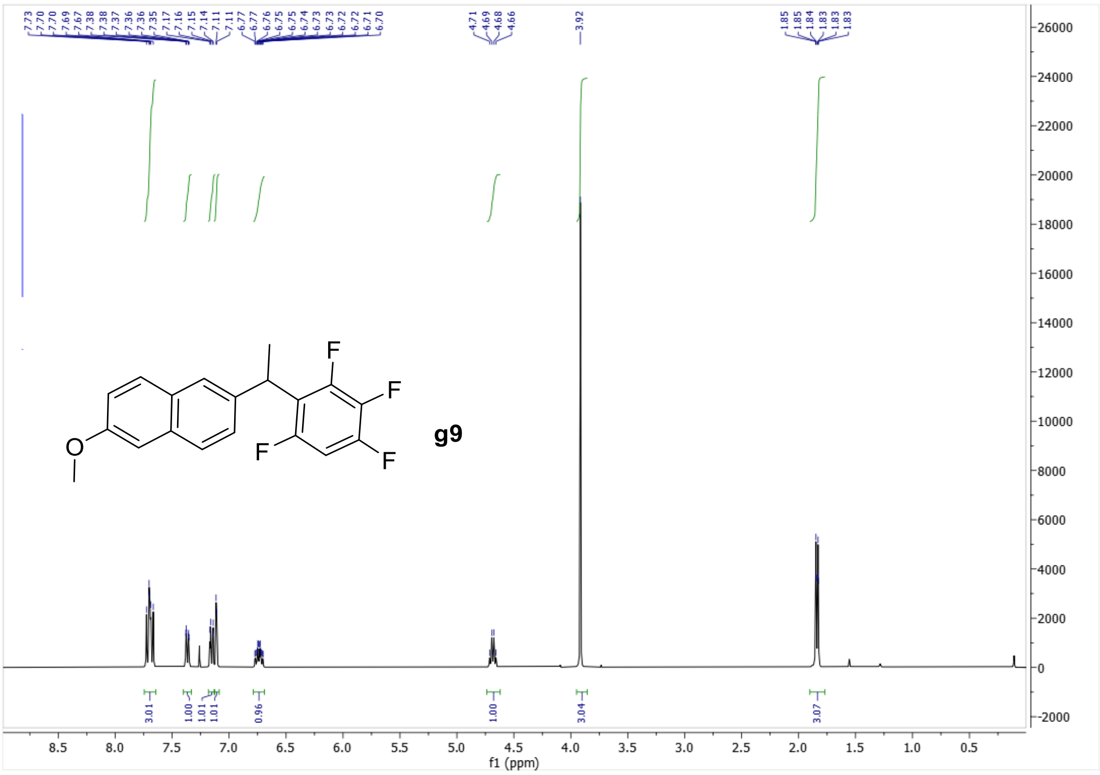

^{1}H-NMR spectrum of (**g9**) (400 MHz, $CDCl_3$)

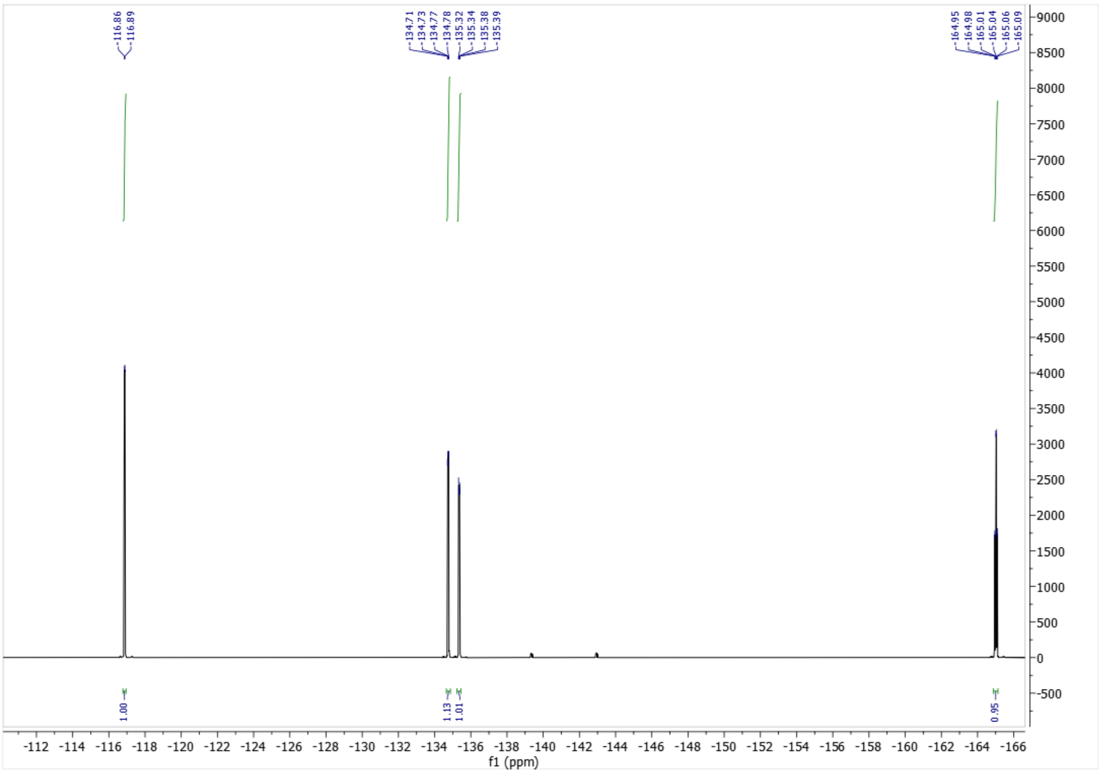

^{19}F-NMR spectrum of (**g9**) (400 MHz, $CDCl_3$)

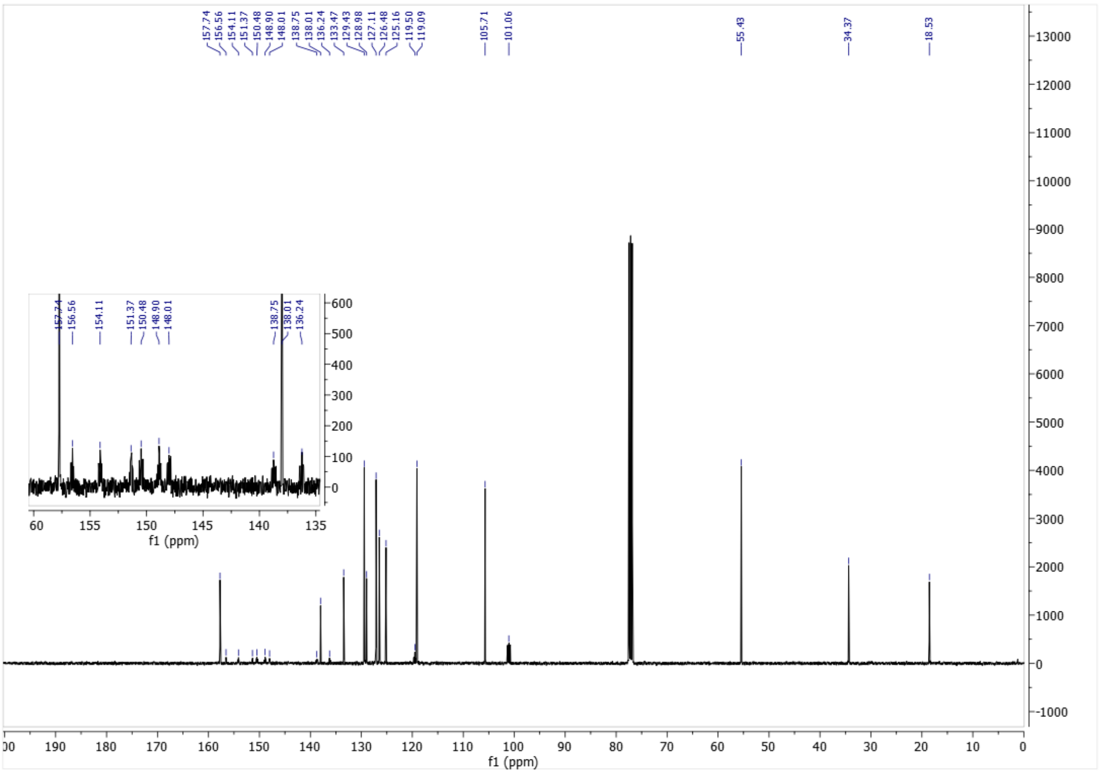

^{13}C-NMR spectrum of (**g9**) (400 MHz, $CDCl_3$)

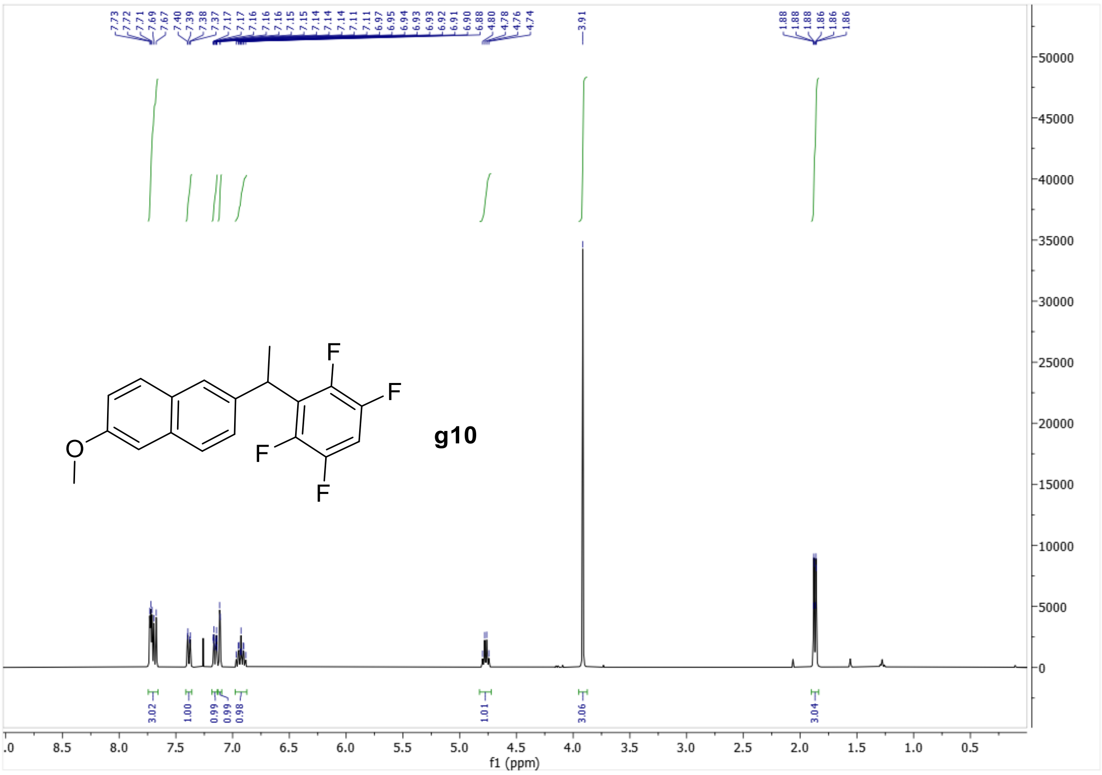

^{1}H-NMR spectrum of (**g10**) (400 MHz, $CDCl_3$)

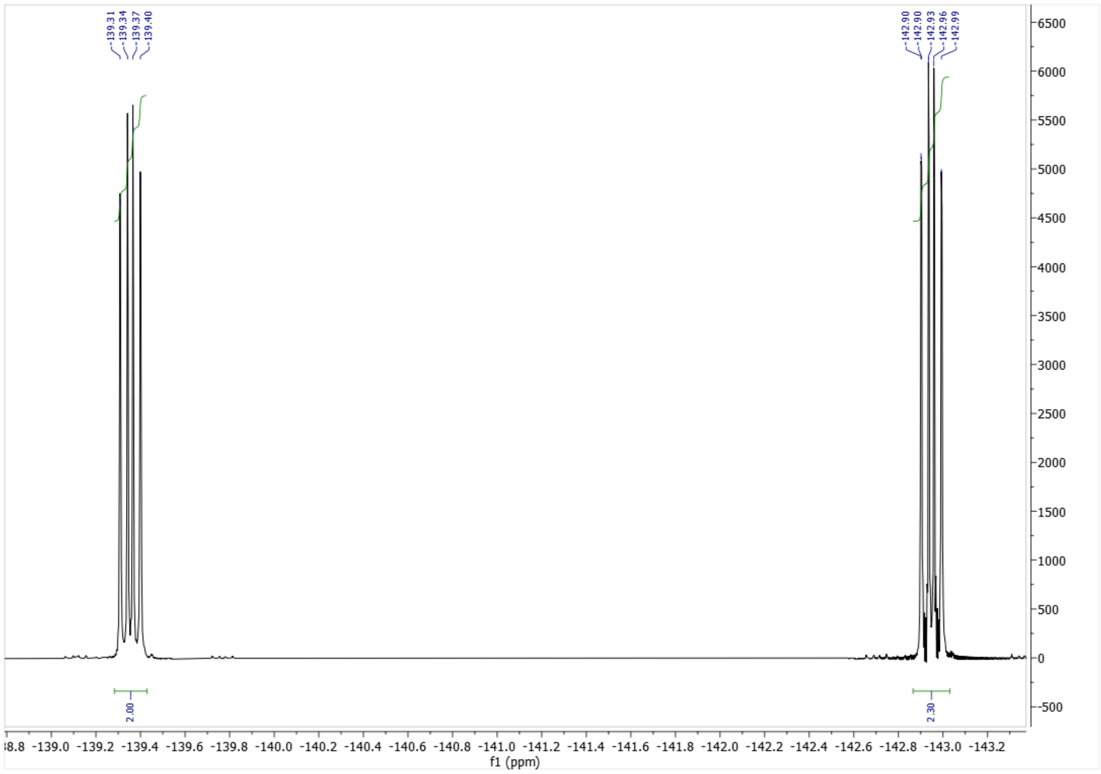

^{19}F-NMR spectrum of (**g10**) (400 MHz, $CDCl_3$)

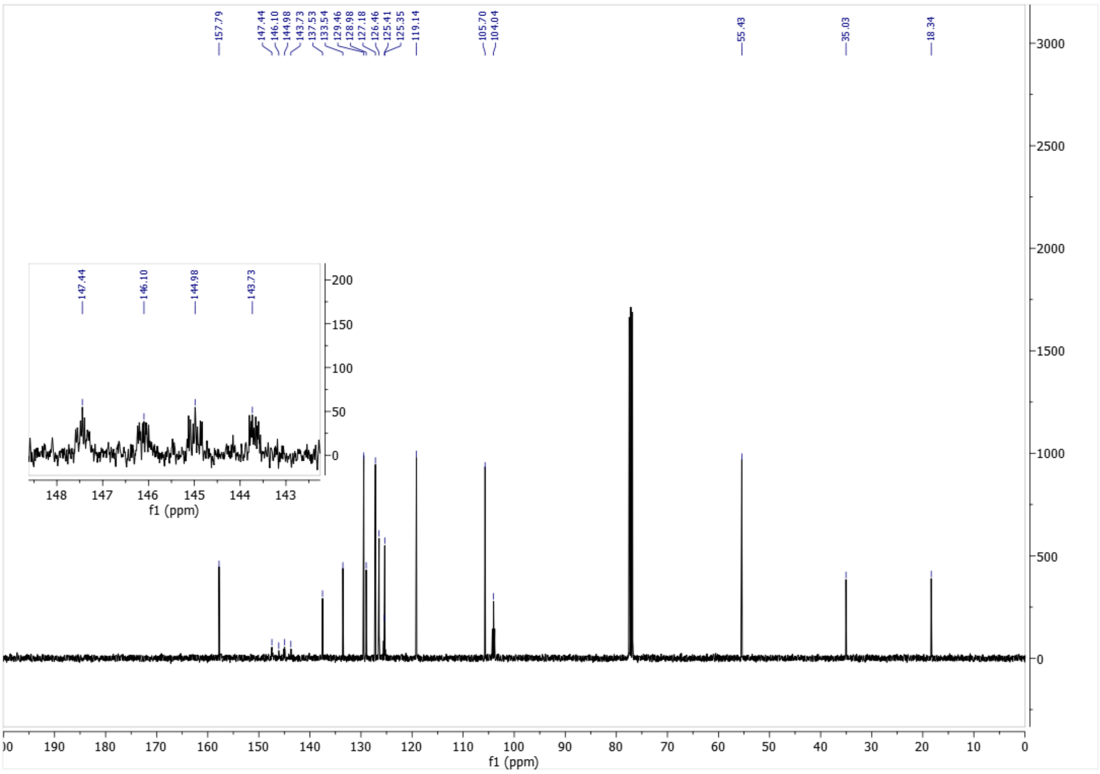

^{13}C-NMR spectrum of (**g10**) (400 MHz, $CDCl_3$)

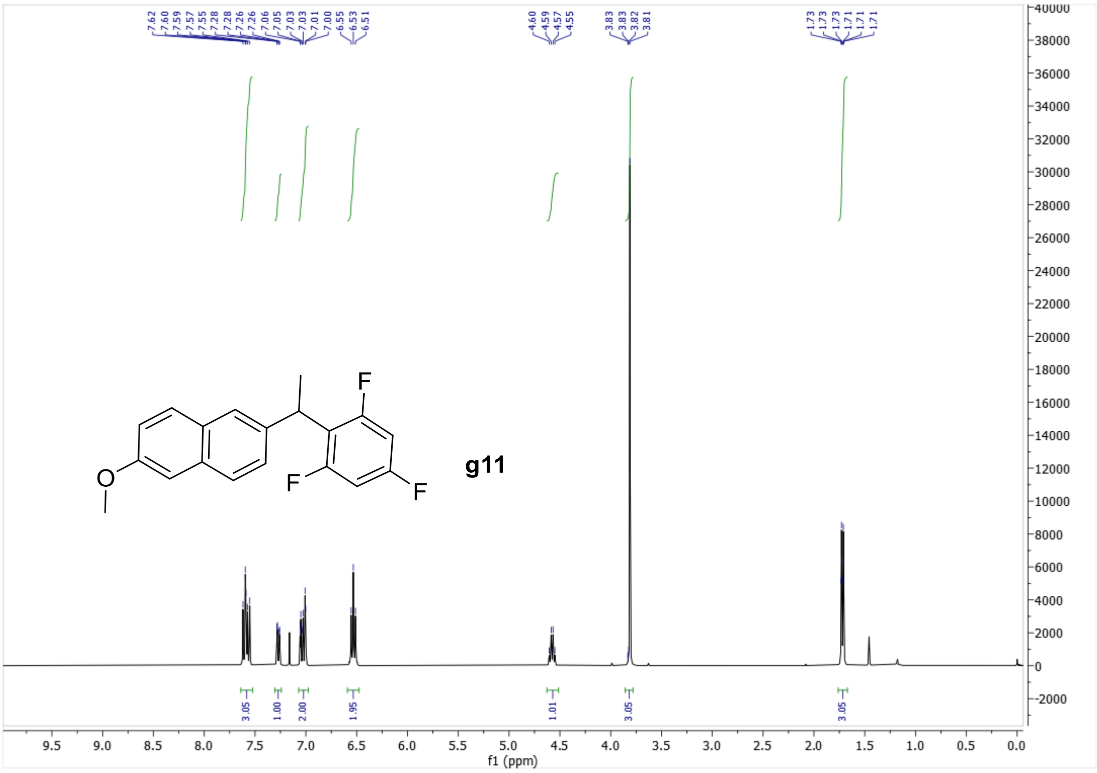

^{1}H-NMR spectrum of (**g11**) (400 MHz, $CDCl_3$)

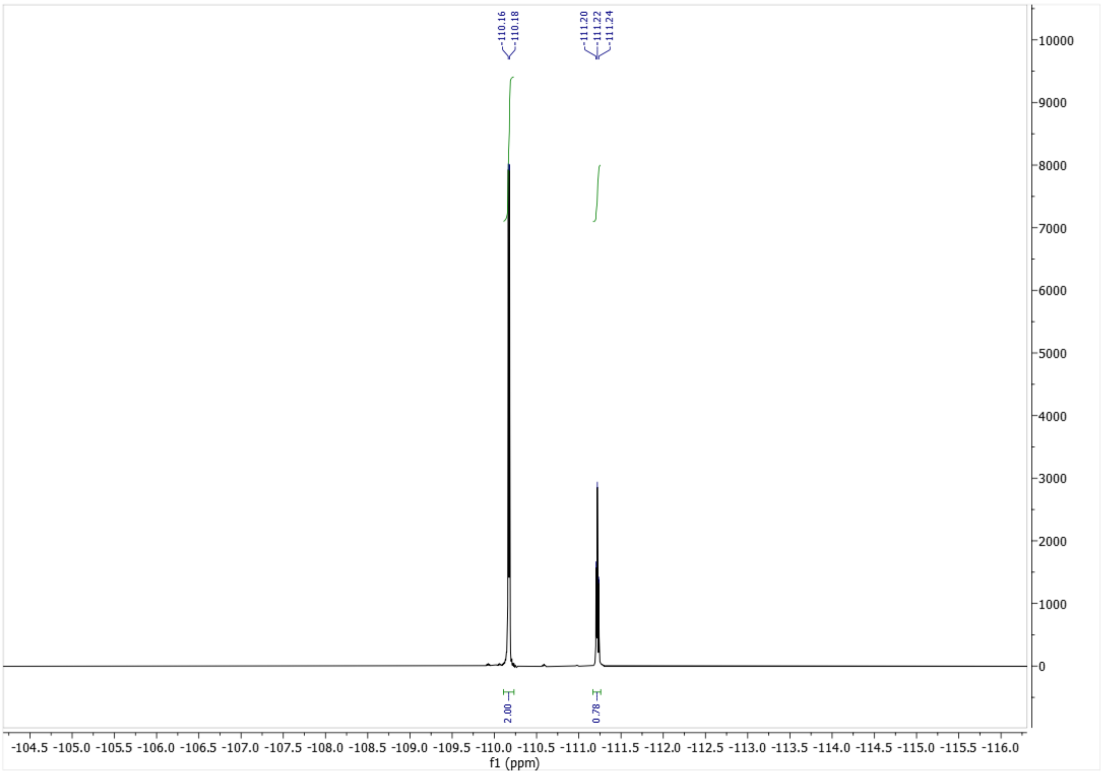

^{19}F-NMR spectrum of (**g11**) (400 MHz, $CDCl_3$)

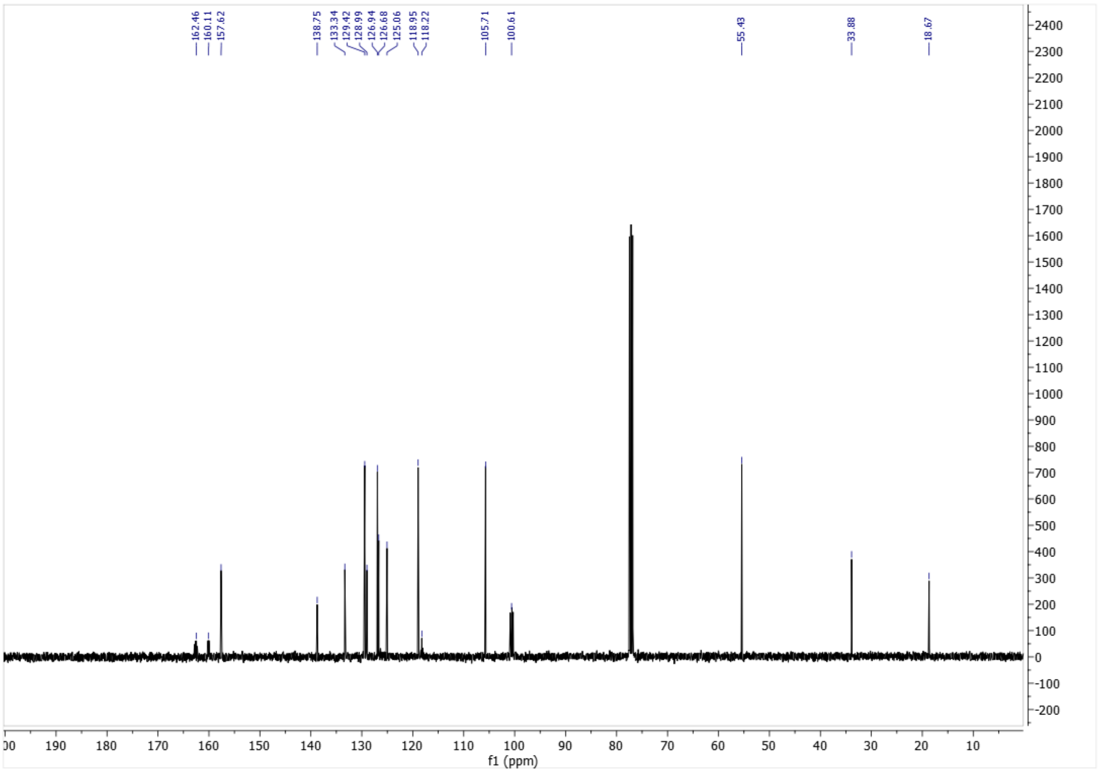

^{13}C-NMR spectrum of (**g11**) (400 MHz, $CDCl_3$)

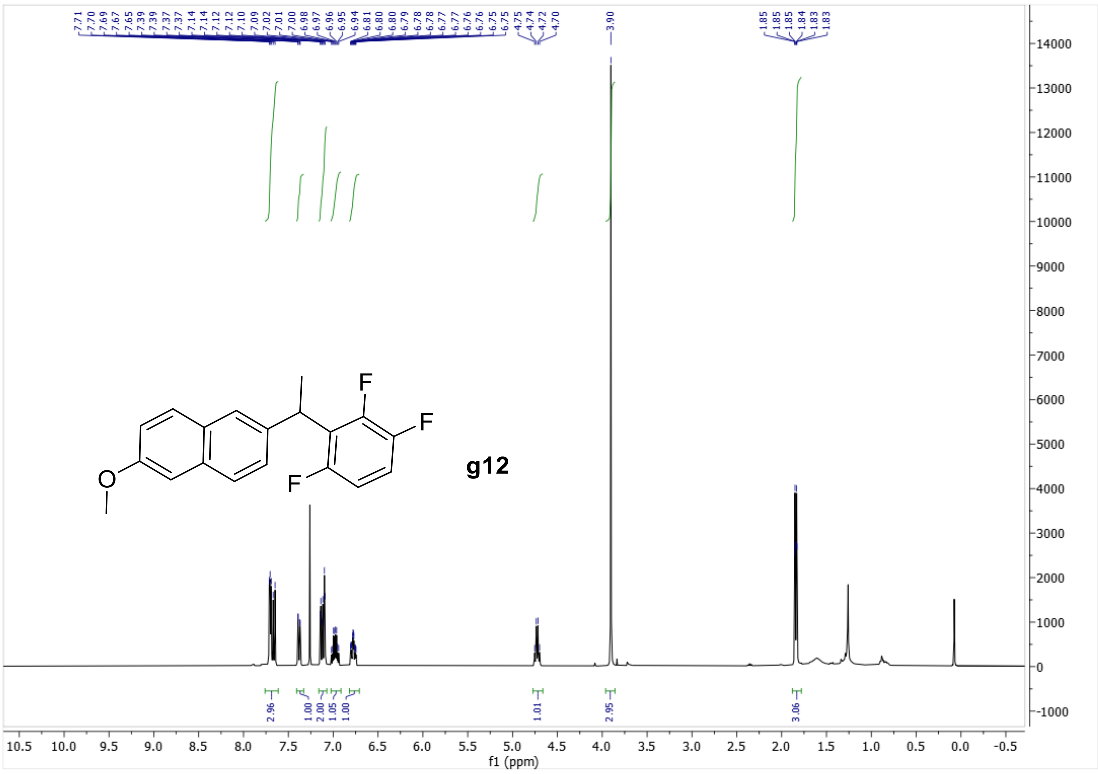

^{1}H-NMR spectrum of (**g12**) (400 MHz, $CDCl_3$)

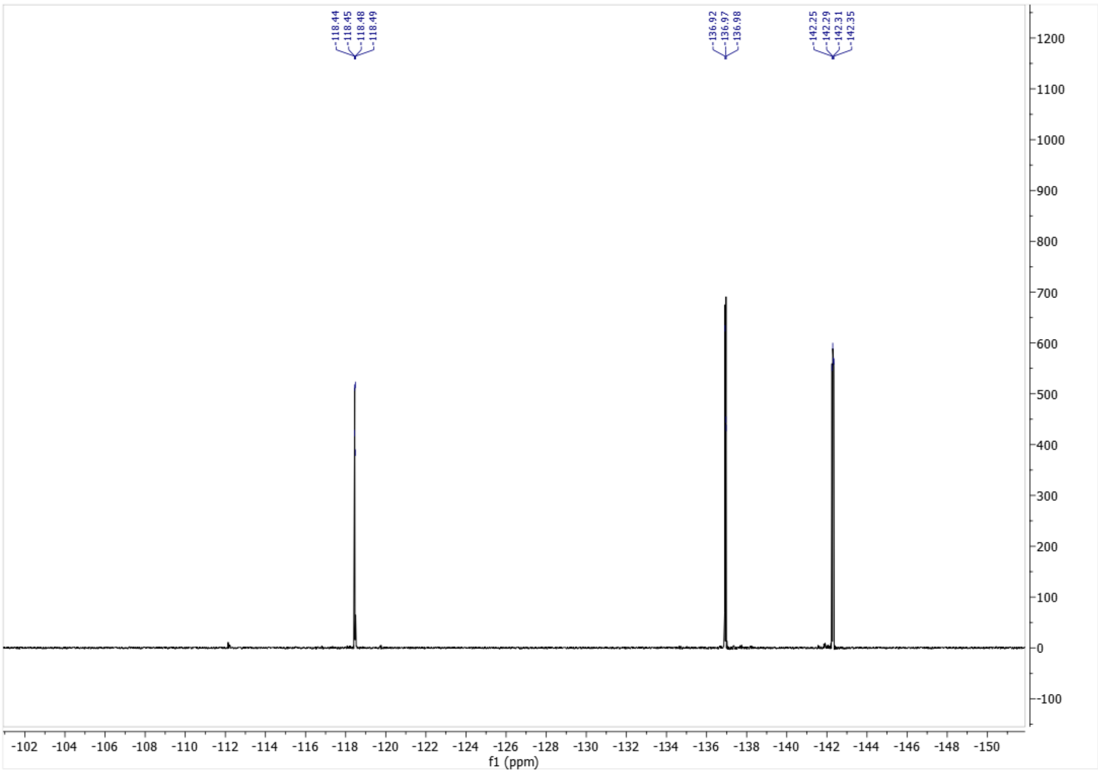

^{19}F-NMR spectrum of (**g12**) (400 MHz, $CDCl_3$)

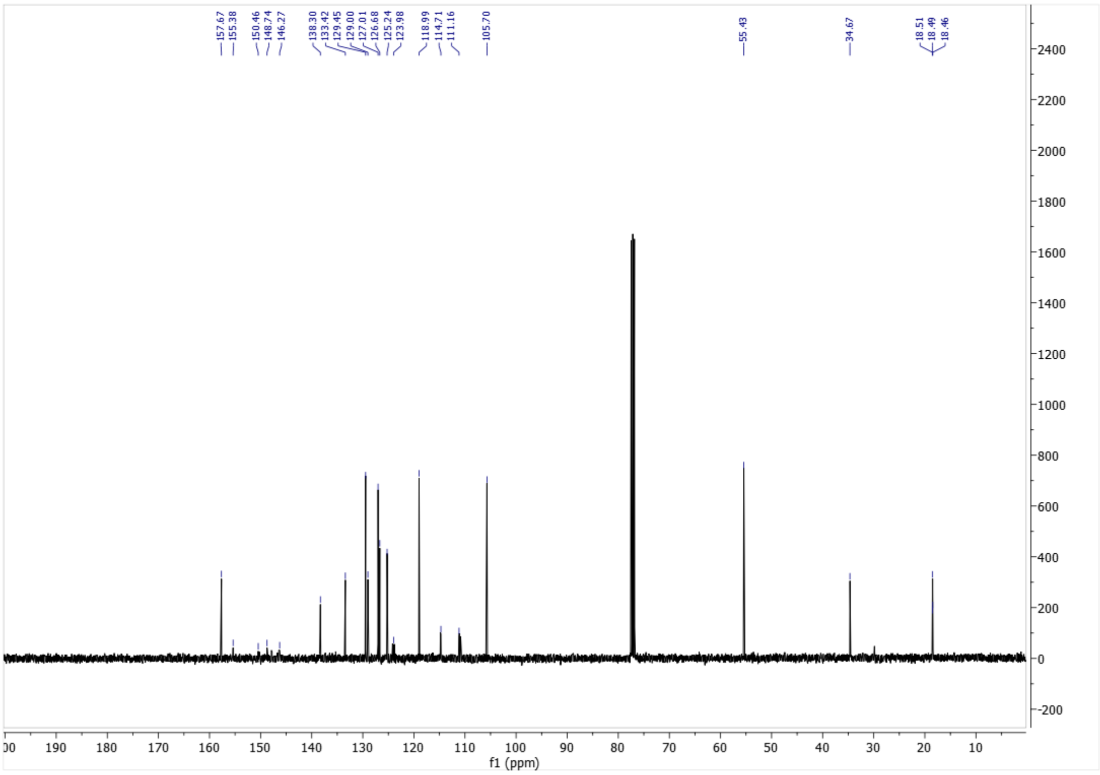

^{13}C-NMR spectrum of (**g12**) (400 MHz, $CDCl_3$)

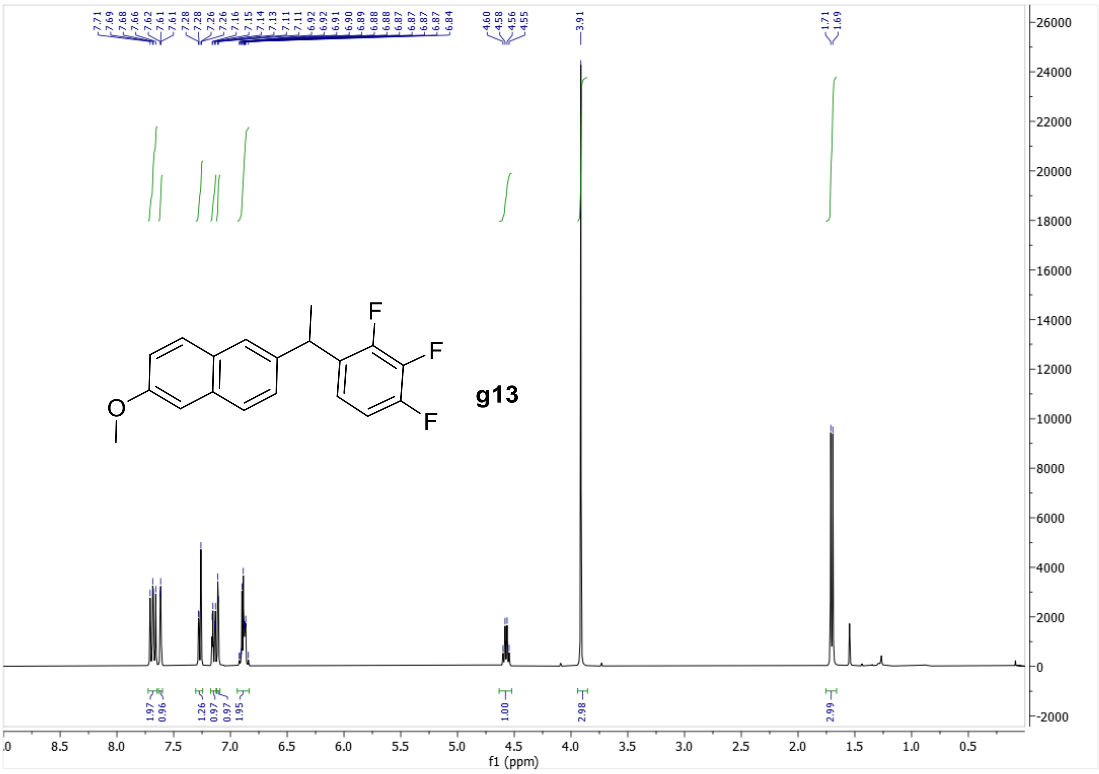

^{1}H-NMR spectrum of (**g13**) (400 MHz, $CDCl_3$)

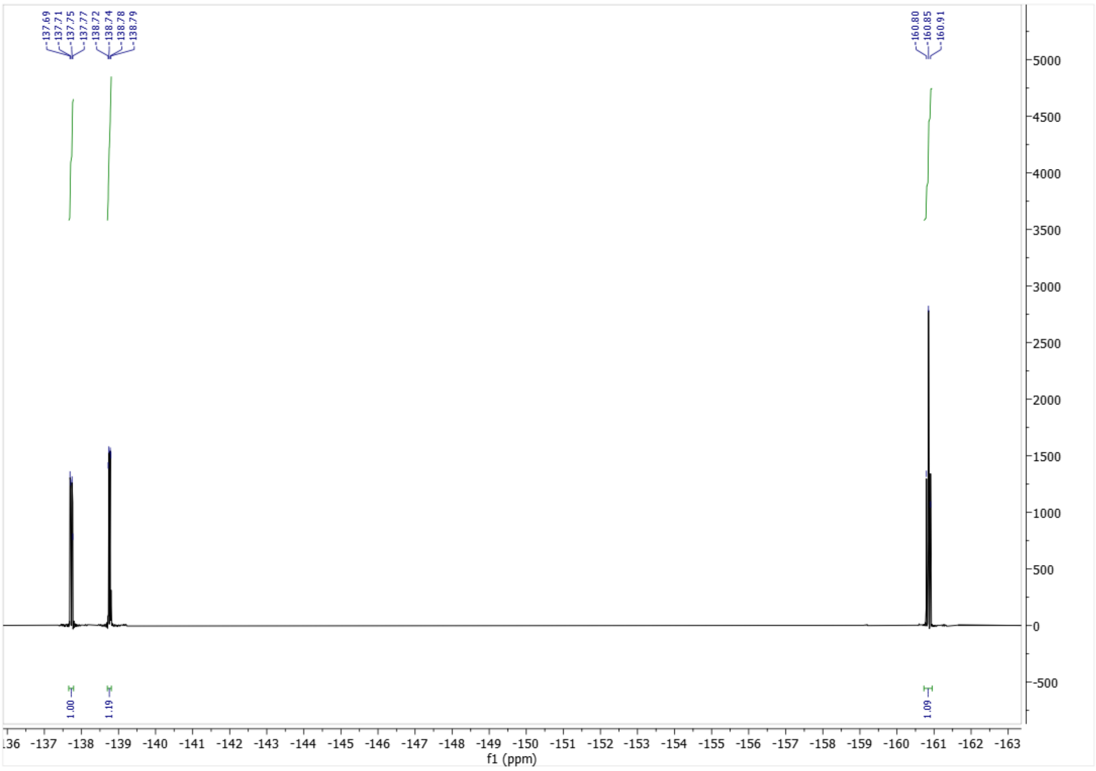

^{19}F-NMR spectrum of (**g13**) (400 MHz, $CDCl_3$)

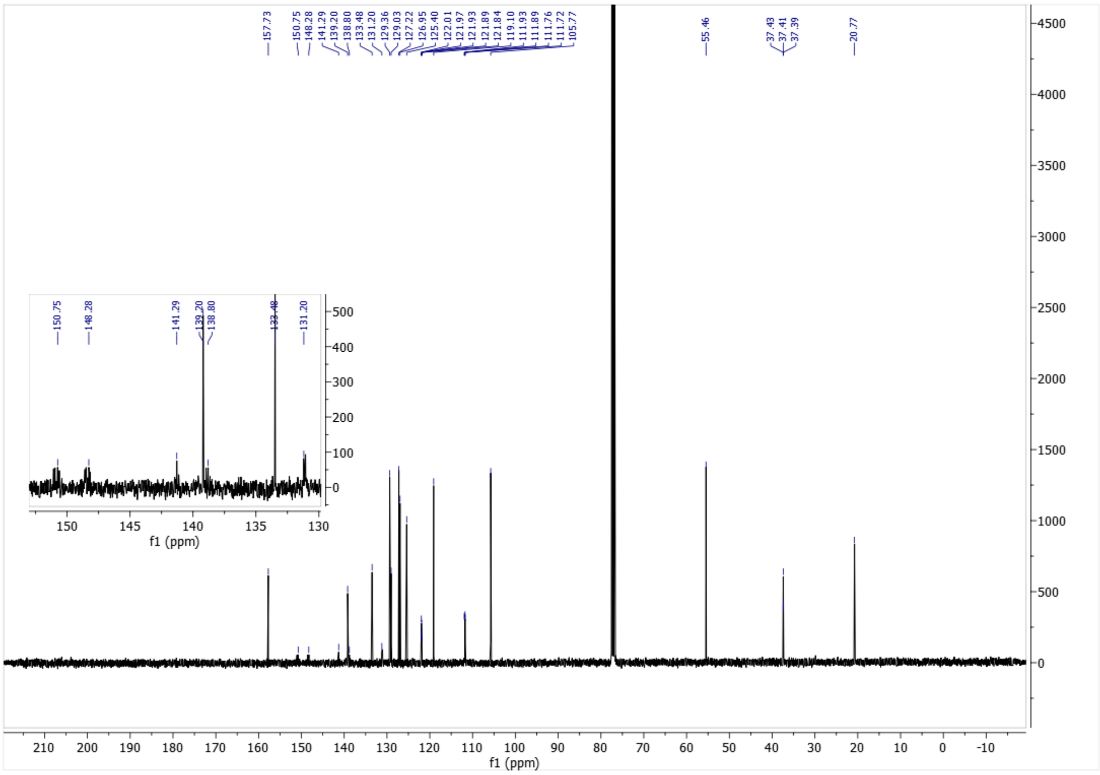

^{13}C-NMR spectrum of (**g13**) (400 MHz, $CDCl_3$)

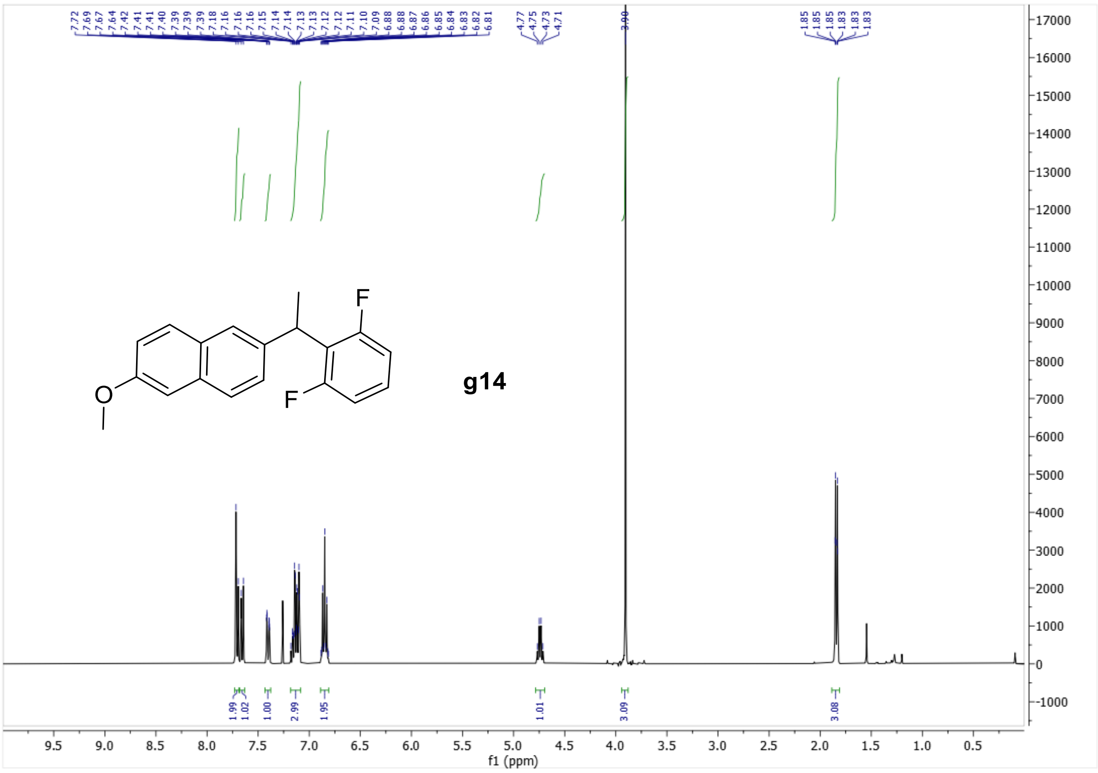

^{1}H-NMR spectrum of (**g14**) (400 MHz, $CDCl_3$)

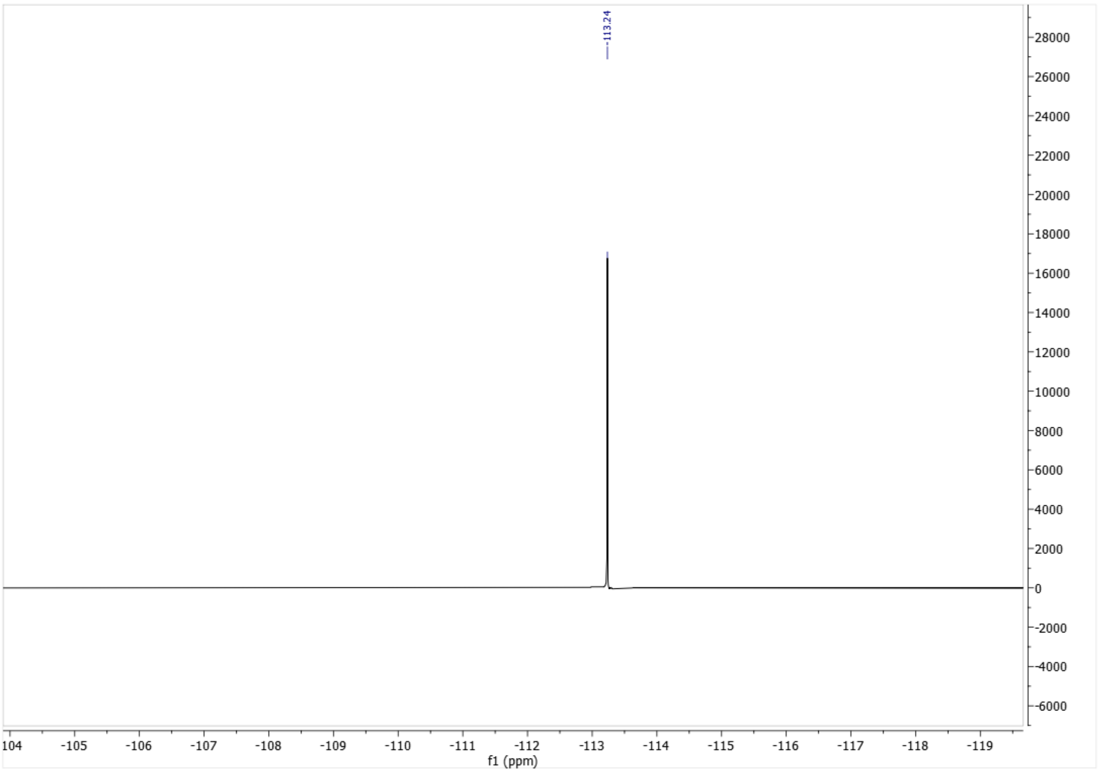

^{19}F-NMR spectrum of (**g14**) (400 MHz, $CDCl_3$)

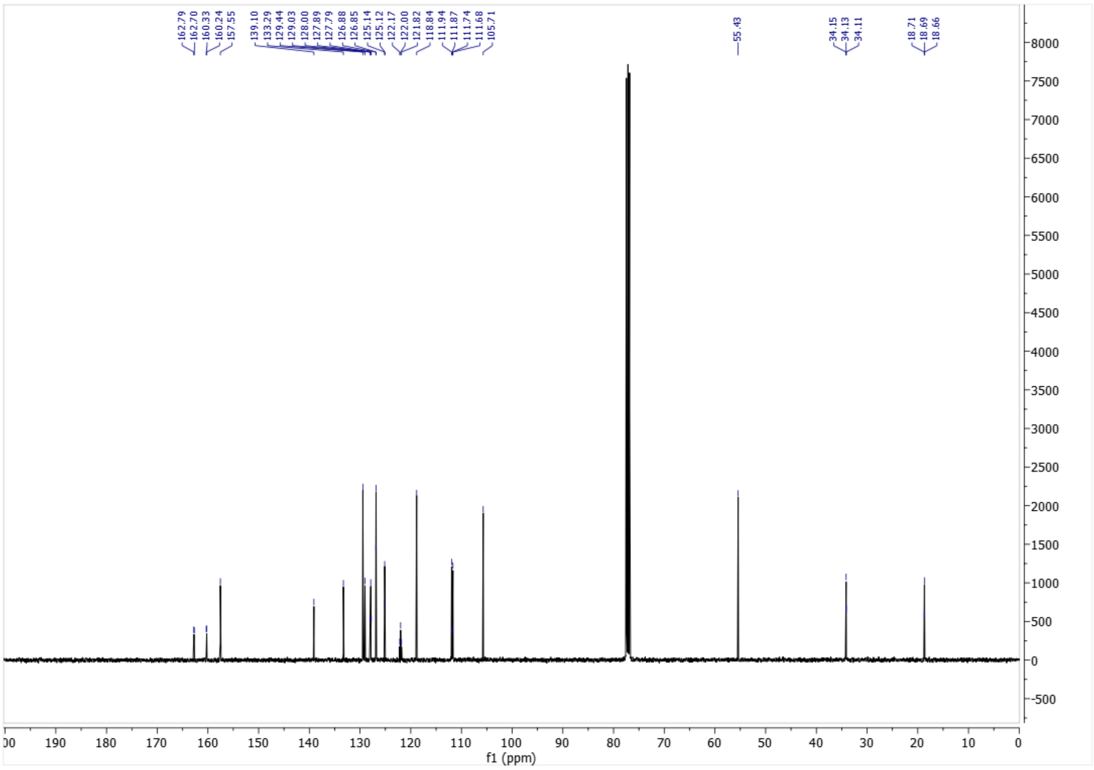

^{13}C-NMR spectrum of (**g14**) (400 MHz, $CDCl_3$)

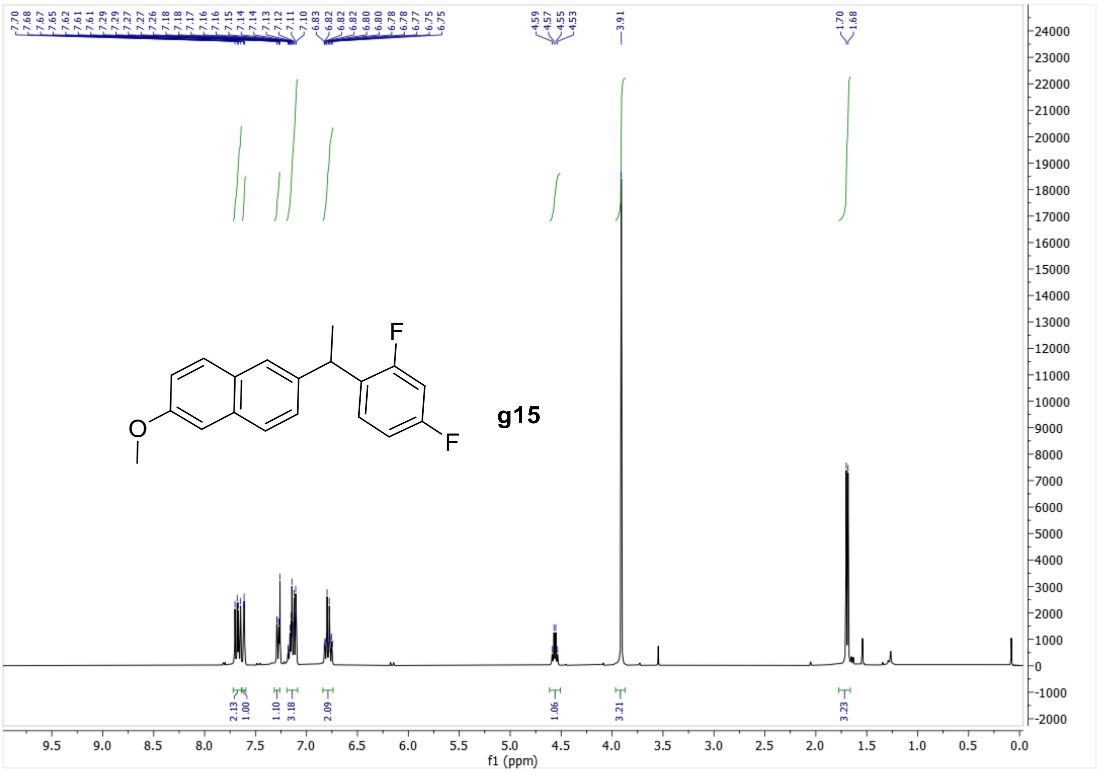

^{1}H-NMR spectrum of (**g15**) (400 MHz, $CDCl_3$)

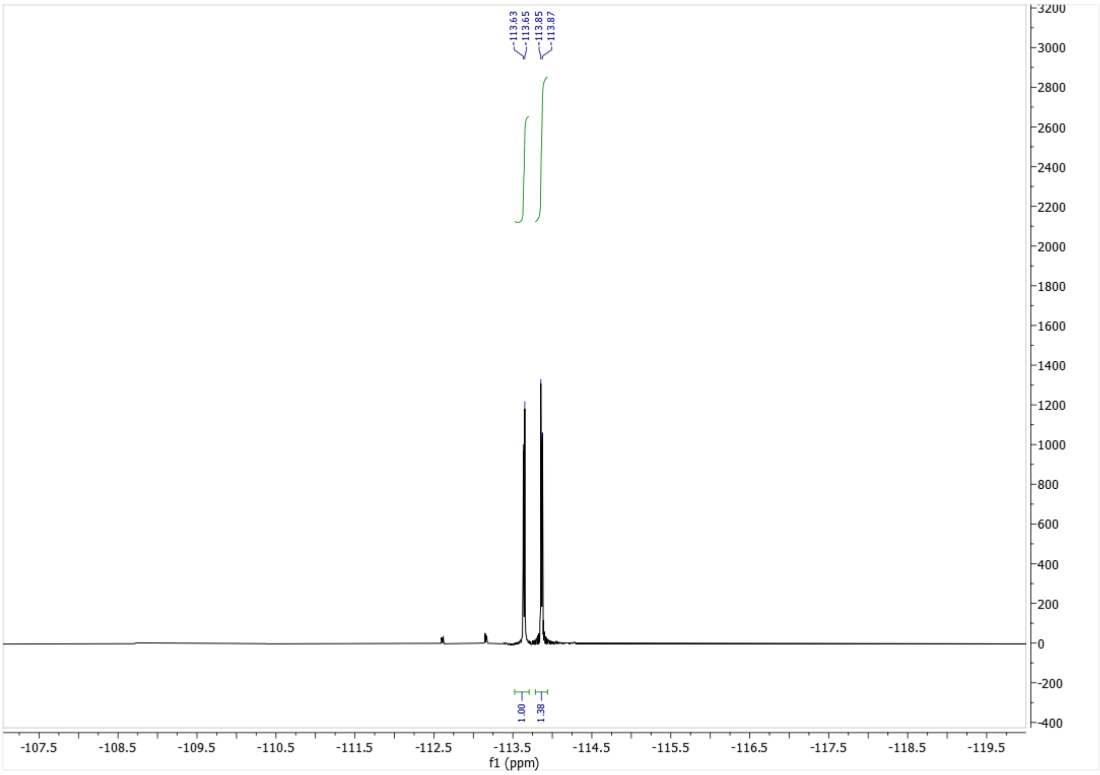

^{19}F-NMR spectrum of (**g15**) (400 MHz, $CDCl_3$)

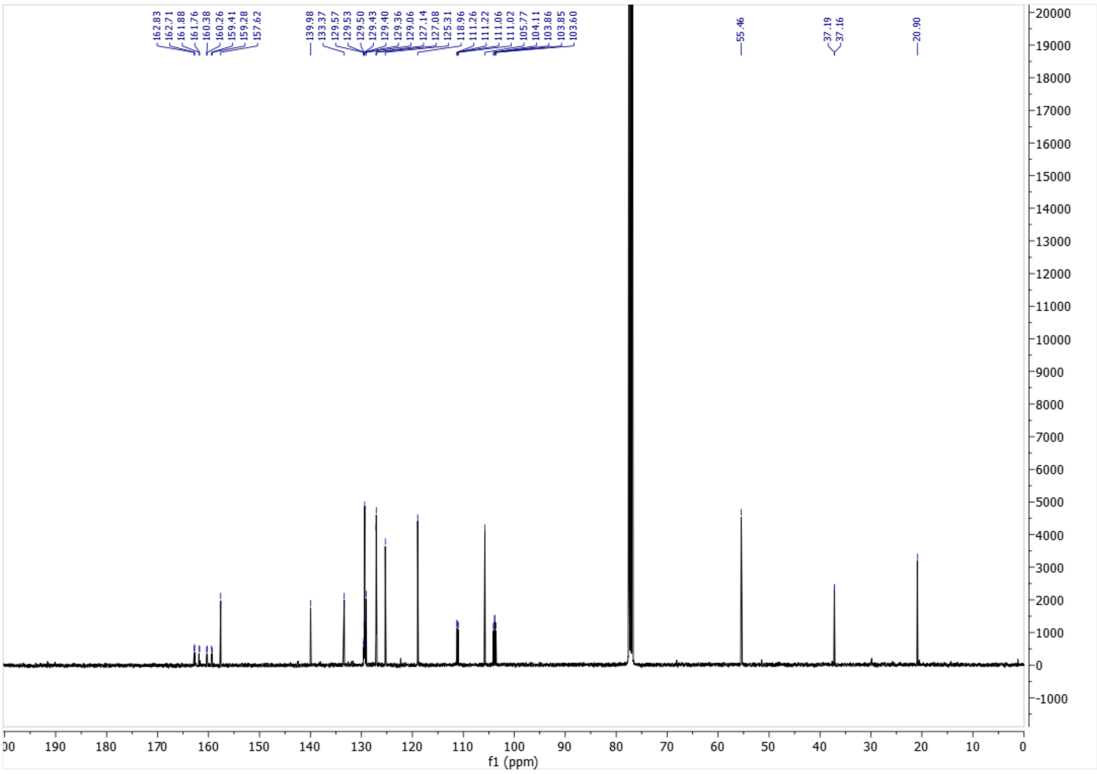

^{13}C-NMR spectrum of (**g15**) (400 MHz, $CDCl_3$)

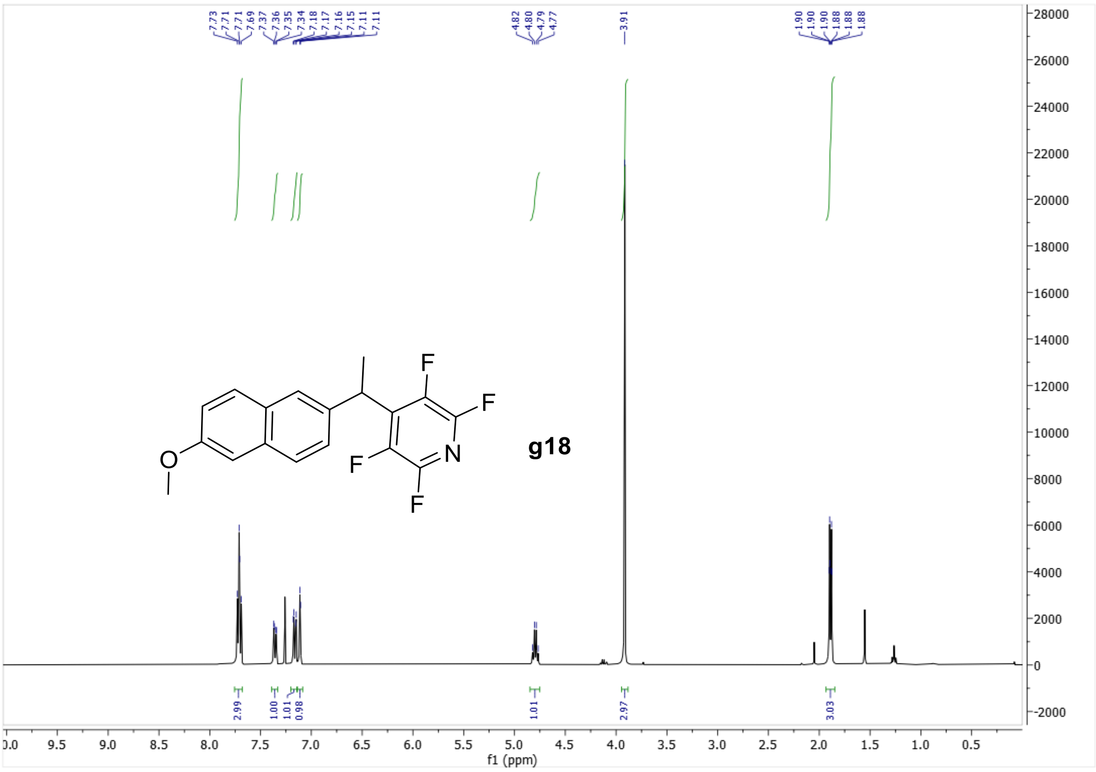

^{1}H-NMR spectrum of (**g18**) (400 MHz, $CDCl_3$)

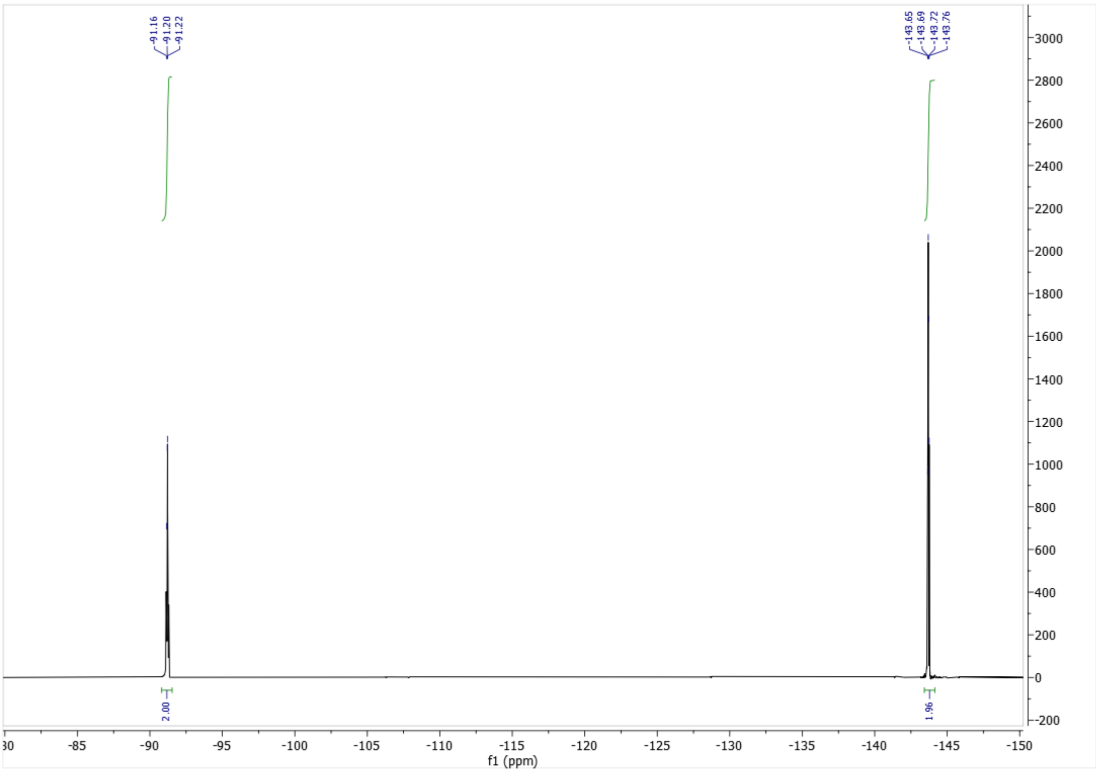

^{19}F-NMR spectrum of (**g18**) (400 MHz, $CDCl_3$)

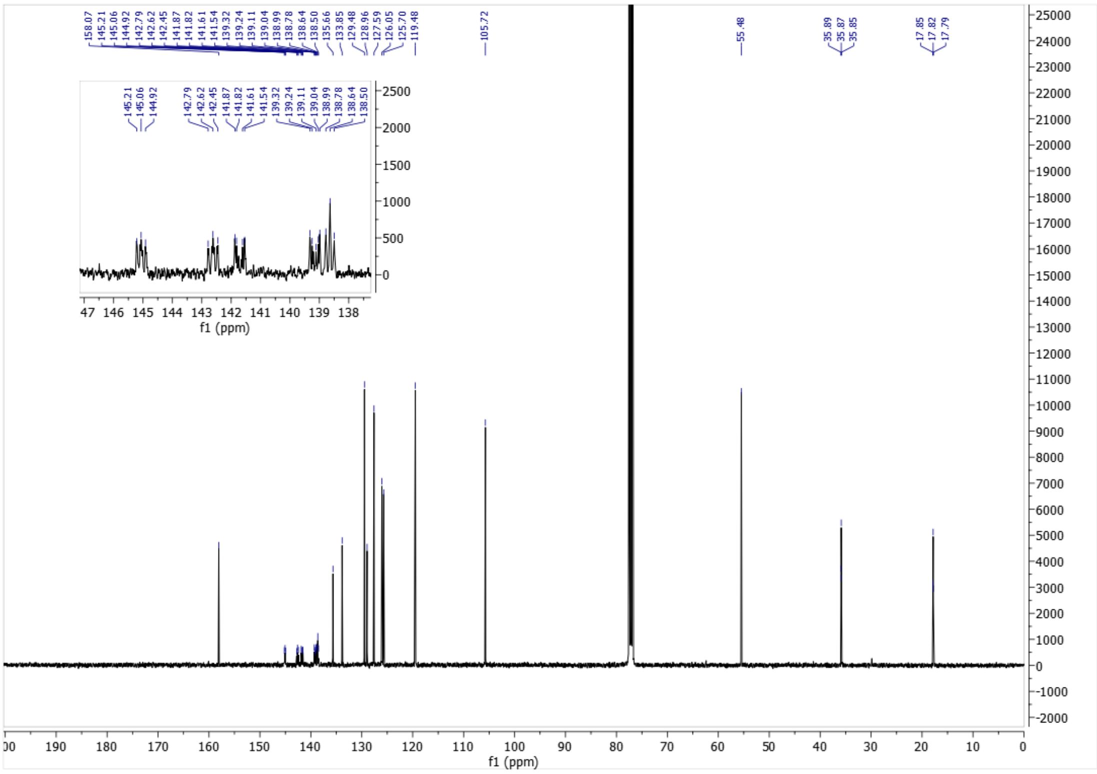

^{13}C-NMR spectrum of (**g18**) (400 MHz, $CDCl_3$)

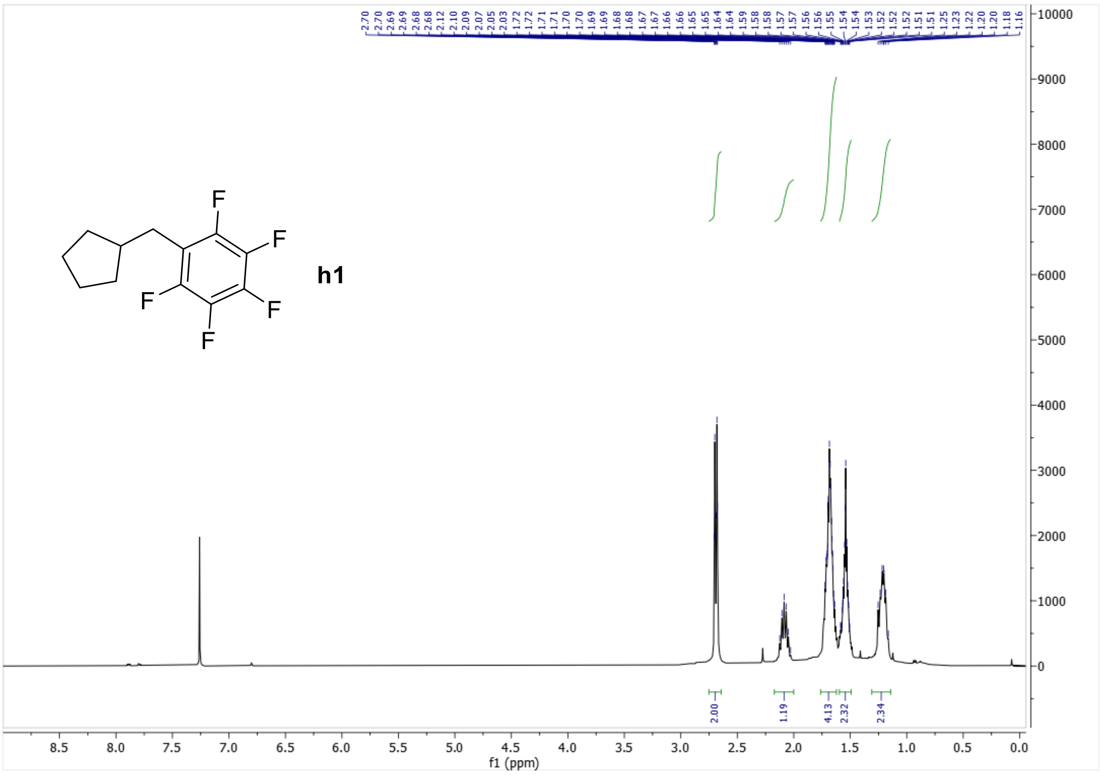

^{1}H-NMR spectrum of (**h1**) (400 MHz, $CDCl_3$)

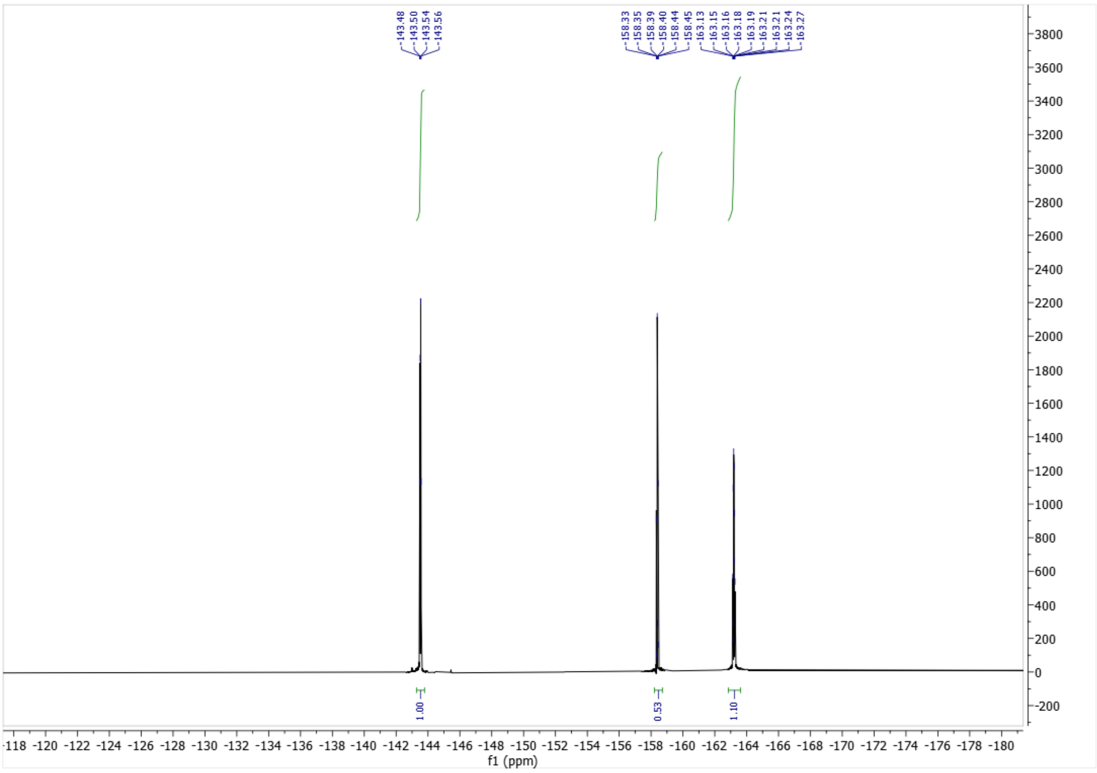

^{19}F-NMR spectrum of (**h1**) (400 MHz, $CDCl_3$)

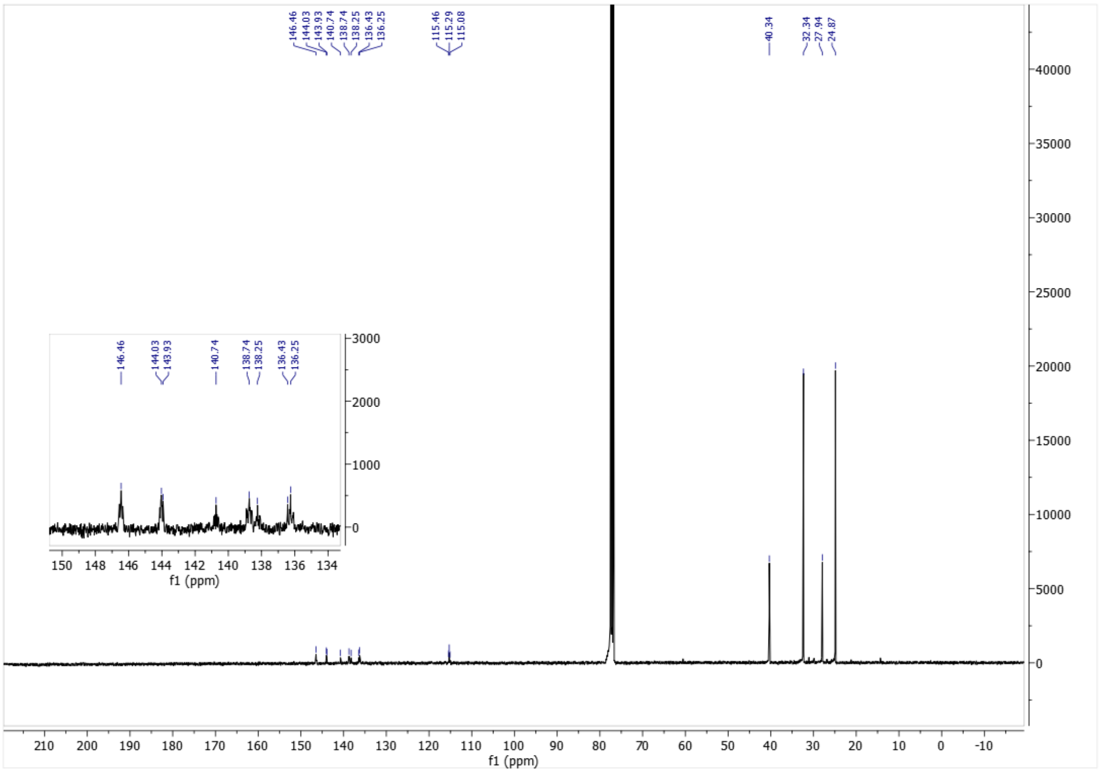

^{13}C-NMR spectrum of (**h1**) (400 MHz, $CDCl_3$)

References:

[1] Mao, R. Z.; Frey, A.; Balon, J.; Hu, X. L. Decarboxylative C(sp(3))-N cross-coupling via synergetic photoredox and copper catalysis. *Nat. Catal.* **2018**, *1*, 120-126.

[2] Xu, R. T.; Xu, T. X.; Yang, M. C.; Cao, T. P.; Liao, S. H. A rapid access to aliphatic sulfonyl fluorides. *Nat. Commun.* **2019**, *10*.

[3] Dong, Y.; Ji, P.; Zhang, Y.; Wang, C.; Meng, X.; Wang, W. Organophotoredox-Catalyzed Formation of Alkyl–Aryl and −Alkyl C–S/Se Bonds from Coupling of Redox-Active Esters with Thio/Selenosulfonates. *Org. Let.* **2020**, *22*, 9562-9567.

[4] Zhao, W.; Wurz, R. P.; Peters, J. C.; Fu, G. C. Photoinduced, Copper-Catalyzed Decarboxylative C-N Coupling to Generate Protected Amines: An Alternative to the Curtius Rearrangement.*J. Am. Chem. Soc.* **2017**, *139*, 12153-12156.

[5] Cornella, J.; Edwards, J. T.; Qin, T.; Kawamura, S.; Wang, J.; Pan, C. M.; Gianatassio, R.; Schmidt, M.; Eastgate, M. D.; Baran, P. S. Practical Ni-Catalyzed Aryl-Alkyl Cross-Coupling of Secondary Redox-Active Esters.*J. Am. Chem. Soc.* **2016**, *138*, 2174-2177.

[6] Doshi, A.; Sundararaman, A.; Venkatasubbaiah, K.; Zakharov, L. N.; Rheingold, A. L.; Myahkostupov, M.; Piotrowiak, P.; Jakle, F. Pentafluorophenyl Copper-Pyridine Complexes: Synthesis, Supramolecular Structures via Cuprophilic and pi-Stacking Interactions, and Solid-State Luminescence. *Organometallics* **2012**, *31*, 1546-1558.

[7] Lu, J. Z.; Khetrapal, N. S.; Johnson, J. A.; Zeng, X. C.; Zhang, J. "pi-Hole-pi" Interaction Promoted Photocatalytic Hydrodefluorination via Inner-Sphere Electron Transfer.*J. Am. Chem. Soc.* **2016**, *138*, 15805-15808.

[8] Xie, W.; Heo, J.; Kim, D.; Chang, S. Copper-Catalyzed Direct C-H Alkylation of Polyfluoroarenes by Using Hydrocarbons as an Alkylating Source.*J. Am. Chem. Soc.* **2020**, *142*, 7487-7496.
